# Chemical Diversity and Complexity of Scotch Whisky as Revealed by High-Resolution Mass Spectrometry

**DOI:** 10.1007/s13361-016-1513-y

**Published:** 2016-10-17

**Authors:** Will Kew, Ian Goodall, David Clarke, Dušan Uhrín

**Affiliations:** 1 EaStCHEM, School of Chemistry, University of Edinburgh, Joseph Black Building, Edinburgh, EH9 3FJ UK; 2 The Scotch Whisky Research Institute, The Robertson Trust Building, Research Avenue North, Riccarton, Edinburgh, EH14 4AP UK

**Keywords:** Scotch Whisky, FT-ICR MS, Complex mixture, CHOS species, Maturation, Multivariate analysis

## Abstract

Scotch Whisky is an important product, both culturally and economically. Chemically, Scotch Whisky is a complex mixture, which comprises thousands of compounds, the nature of which are largely unknown. Here, we present a thorough overview of the chemistry of Scotch Whisky as observed by Fourier transform ion cyclotron resonance mass spectrometry (FT-ICR MS). Eighty-five whiskies, representing the majority of Scotch Whisky produced and sold, were analyzed by untargeted high-resolution mass spectrometry. Thousands of chemical formulae were assigned for each sample based on parts-per-billion mass accuracy of FT-ICR MS spectra. For the first time, isotopic fine structure analysis was used to confirm the assignment of high molecular weight CHOS species in Scotch Whisky. The assigned spectra were compared using a number of visualization techniques, including van Krevelen diagrams, double bond equivalence (DBE) plots, as well as heteroatomic compound class distributions. Additionally, multivariate analysis, including PCA and OPLS-DA, was used to interpret the data, with key compounds identified for discriminating between types of whisky (blend or malt) or maturation wood type. FT-ICR MS analysis of Scotch Whisky was shown to be of significant potential in further understanding of the complexity of mature spirit drinks and as a tool for investigating the chemistry of the maturation processes.

**Electronic supplementary material:**

The online version of this article (doi:10.1007/s13361-016-1513-y) contains supplementary material, which is available to authorized users.

**Graphical Abstract:**
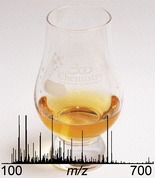
ᅟ

## Introduction

Scotch Whisky is a high value product, both commercially and culturally, generating £3.86bn in 2015 in UK exports to over 200 markets [1]. Chemically, Scotch Whisky is a complex mixture, which comprises several thousands of compounds of a largely unknown nature. From this point of view, it can be compared with other complex mixtures such as soil organic matter, dissolved organic matter, or organic aerosols. Natural organic matter (NOM) is a subject of intense research in developing methodology for its characterization at the molecular level [2–5]. Related to these efforts are studies of metabolites [6, 7], plant extracts [8], or indeed other foods or beverages [9–12]. This work thus contributes to achieving broader goals of analytical chemistry in the area of analysis of complex mixtures.

Routine analysis of Scotch Whisky involves separation techniques, such as gas or liquid chromatography, often coupled with commonly available detectors, including UV, flame ionization detector (FID), or mass spectrometry (MS) [13–18]. With these techniques, many of the major compounds in whisky have been characterized [13]. These analyses are highly targeted, and the determined concentrations of specific congeners are often used as a measure of the authenticity of whisky. For example, the concentrations of 2- and 3-methyl-butanol are associated with the relative amount of malt in a blended whisky product [19, 20]. This type of targeted approach is very useful, but has its limitations. More sensitive techniques demonstrate that whisky contains many hundreds or thousands of compounds [17, 21, 22], many of which have unknown structures. Therefore, in order to gain an understanding of the complete chemical make-up of whisky, non-targeted approaches are essential.

The resolution and sensitivity of FT-ICR MS enabled studies and characterization of a number of complex mixtures, e.g., natural organic matter (NOM) [5, 23, 24], and many -omics sciences have emerged such as metabolomics [25], petroleomics [26, 27], and even “foodomics” [28], looking at complex food products, including vegetable oil [29], beer [30], wine [12, 31–33], champagne [34, 35], and whisky [22]. A typical analysis by FT-ICR MS consists of acquiring electrospray ionization (ESI) or matrix-assisted laser desorption/ionization (MALDI) mass spectra, determination of the molecular formulae of the observed peaks—usually by Kendrick mass defect analysis [36–38]—and production of van Krevelen diagrams [37, 39, 40] to visualize the distribution of elemental compositions in the sample. Whilst such presentation of the MS data is informative, interpretation of the data towards developing understanding of the chemical complexity of samples is still a challenge. Multivariate statistical analysis has been used to examine differences between FT-ICR MS spectra of different samples [32, 41–44], typically by compiling an input matrix containing normalized signal intensities versus either exact *m/z* or assigned chemical formula for each sample. A detailed overview of multivariate analysis in –omics studies is presented by Wheelock et al. [45].

Before we present our results, we briefly outline the background to Scotch Whisky as a mature spirit drink. The production of whisky involves a number of key steps: malting of barley; fermentation of the cereals to an alcoholic wort; distillation; and maturation, where the distillate, termed *new-make spirit*, must spend at least 3 y in oak casks in Scotland to become Scotch Whisky. The chemistry of maturation is not fully understood [13, 46, 47]; however, during this stage, the interaction between spirit and cask produces a complex mixture containing thousands of compounds. Across Scotland, there are over 100 licenced distilleries producing whisky in a large number of styles [48]. The Scotch Whisky Regulations 2009 formally define five categories of Scotch Whisky – (1) *single malt*, (2) *single grain*, (3) *blended malt*, (4) *blended grain*, and (5) *blended Scotch Whisky* [49]. Blended Scotch Whisky represents the largest product category by volume of sales, but single malts are also significant, with many premium products on the market [50, 51].

Malt whiskies are produced solely from malted barley, whilst blended whisky and grain whiskies can include other cereal sources, such as wheat or maize [48]. A single malt, or single grain, is the product of a single distillery, whereas a blended malt, or blended grain, is the product of two or more distilleries. A blended Scotch Whisky is a blend of at least one single malt and one single grain whisky. Some producers infuse peat smoke into the barley at the kilning stage, producing a peated whisky. Distillation processes differ for malt and grain whisky, with grain whisky being distilled in a continuous process to a higher alcoholic strength (94% ABV) [52], whereas malt whisky is batch distilled to a lower strength (70% ABV) [53]. This has a significant effect on the character of the distillate, with grain new-make spirit having a lighter sensory profile and malt new-make spirit retaining more pre-distillation flavor compounds. After distillation, maturation occurs in an oak barrel [47]. Typically, the barrels have previously been used for maturation of Bourbon whiskey in the USA, or in the production of Sherry wine in Jerez, Spain. A whisky may be matured for several years in one cask before it spends a shorter period of time, typically 6 mo or less, in a different type of cask. This process is known as finishing the whisky [46]. Other factors in maturation of whisky include the cask size, age, number of refills, char or toast level of the barrel, previous fills, and oak species used [47]. Additionally, environmental variables such as temperature and humidity have an effect on the maturation of Scotch Whisky. Importantly, the final product, *even for a single malt*, is usually a blend of a number of different casks to produce a product that has the same sensory profile year on year [46]. The only ingredients allowed to be added to Scotch Whisky are water and E150a caramel coloring [49].

All of the variables involved in whisky production contribute to the overall chemical composition and sensory profile of the final product [47]; as a result, the analysis of whisky presents a highly challenging problem [13]. Additionally, the time and volume scales involved in whisky production make designing a sample set for analysis a non-trivial matter. In order to overcome these challenges, in this work a large number of samples of both blend and malt classifications were studied. The samples were selected to represent a broad range of the majority of Scotch Whisky produced and sold.

Previous use of high-resolution mass spectrometry for the analysis of Scotch Whisky is limited, with only a couple of studies in the literature. ESI-QTOF was used to compare a small number of Scotch and American whiskies [21], including several counterfeit samples. The technique was able to classify the samples according to country of origin and establish their authenticity or otherwise. To date, the authors could find only one report on the analysis of Scotch Whisky by ESI-FT-ICR MS [22], which examined a small number of real and counterfeit samples of blended whiskies. Here we show that through the direct and untargeted analysis of Scotch Whisky using high-resolution mass spectrometry, it is possible to observe and assign thousands of chemical compounds. The resulting spectra can provide a unique chemical fingerprint of each sample. Our analysis of a comprehensive sample set of 85 whiskies highlighted a number of key points, including product reproducibility, the use of isotopic fine structure analysis on small molecules for confident formula assignment, and the importance for multivariate analysis for sample discrimination, e.g., based on maturation wood types. These results shed some light on the little understood chemical processes and transformation that occur during the maturation process of Scotch Whisky.

## Experimental

### Sample Preparation

Scotch Whisky samples were provided by the Scotch Whisky Research Institute (SWRI). A total of 85 authentic Scotch Whisky samples were analyzed, consisting of a mixture of malts and blends. The main sample set was the SWRI standard sample set from 2014 (n_malts_ = 24, n_blends_ = 28). Further samples were sourced from subsets of previous years’ standard sample sets (i.e., 2010 (n_malts_ = 23, n_blends_ = 1) and 2012 (n_malts_ = 8, n_blends_ = 1). The sample sets were curated by the SWRI to represent the majority of Scotch Whisky sold as UK or export case sales; 2014 (73.15% of total sales), 2012 (71.2% of total sales), and 2010 (67.4% of total sales) [51, 54, 55]. In this work, malts refer to both single malt and blended malts, whilst blends refer to blended whisky. There are no single or blended grain whiskies in the sample set, as these represent a very small percentage of products on the market. A malt new-make spirit sample was also analyzed. Samples are referred to by an anonymized reference label of the format S*YY*-*XXXX*, where *YY* refers to the sample set year and XXXX is a unique identifier for the sample in that year. LC-MS grade methanol and water were purchased from Sigma Aldrich, Dorset, UK. Samples (30 μL) were diluted 1:10 in methanol:water (50:50) immediately prior to direct infusion into the ESI source. The dilution ratio was optimized to minimize potential carryover effects.

### ESI FT-ICR MS Analysis

The MS spectra were acquired on a 12 Tesla SolariX FT-ICR MS (Bruker Daltonics, Bremen, Germany) with an ESI source. Nebulizer gas flow was set to 1.8 bar, drying gas was 6 L/min at 180 °C. Broadband spectra were acquired with 200 summed scans between 98.3 *m/z* and 1000 *m/z* into a 4 MW FID of 1.1185 s. Time of flight was set to 0.6 ms with an ion accumulation time of 150 ms. The mass resolution achieved was over 300,000 at 400 *m/z*. For acquisition of narrow windows for isotopic pattern analysis, ions were filtered using the quadrupole, and the ion accumulation time was increased to 2000 ms. For fragmentation experiments, specific ions were isolated using the quadrupole and fragmented using CID in the collision cell (MS^2^) or source and collision cell (MS^3^). Solvent blanks were run after every 12 samples and at the start and end of each experimental session. Three samples were analyzed in replicate across different days to validate the instrumental reproducibility, which remained acceptable. Potential carryover effects were minimized by flushing the syringe, capilliary, and sprayer with methanol:water prior to infusion of the next sample. Spectra were acquired in either the negative- or positive-ion mode. However, only negative-ion data was used for data analysis. A preference for negative mode ESI analysis of complex mixtures has been widely reported elsewhere [12, 21, 22, 30, 56].

### Data Processing and Visualization

Spectra were calibrated and peak picked in Data Analysis 4.4 (Bruker Daltonics). The calibration list was based on a number of known compounds and formulae previously determined (Online Resource Supplementary Table S2), and the calibration function was quadratic. Spectra were peak picked with a SNR threshold of 4 and a minimum absolute intensity of 2 × 10^6^, a value based on visual inspection of the data. The peak lists were exported as text files to PetroOrg S-10.2 (The Florida State University) for assignment. Assignment in PetroOrg was set with the elemental limits C_0-100_ H_0-200_ O_1-20_ S_0-1_ with a maximum error threshold of 1 ppm and a minimum of 15 species per class. Only singly charged species were observed and assigned. Between 72% and 88% of peaks were assigned across the entire Scotch Whisky sample set, with an average assignment error of 99 ppb. Assignments were exported from PetroOrg for data analysis and visualization using in-house Python scripts, and for multivariate analysis.

Van Krevelen [39, 40] plots were produced using in-house Python scripts and Matplotlib [57]; each assigned monoisotopic formula was represented as a circle on a scatter graph based on the O/C ratio versus H/C ratio. The circles are sized according to normalized relative peak intensity, where the sum of intensities per spectrum is set to 1. The circles are colored according to mass. Double bond equivalent (DBE) versus carbon number plots were produced in a similar manner, with DBEs calculated by PetroOrg. The formula for DBE calculation is as follows; $DBE=1+\frac{1}{2}\left(2C-H+N+P\right)$. As our formula assignments included only CHOS, the DBE calculation is based solely on carbon and hydrogen content. The circles were again sized by relative peak intensity, but colored according to the oxygen number to compensate for the fact that the DBE parameter only reflects the elements CHNP [4].

### Multivariate Analyses

A data table was constructed for *n* samples (observations) against *m* variables (assigned molecular formulae) using peak intensities, where NA values (peaks not found in a given sample) were filled with a random value at the level of the noise. Thus, this results in no formulae being excluded from the model. Data were then normalized, per spectra, to a sum total intensity of 1. Data were then mean centered and scaled to unit variance prior to PCA or OPLS-DA model construction. PCA and OPLS-DA was performed with SIMCA 14.1 (Umetrics, MKS Data Analytics Solutions).

## Results and Discussion

### High-resolution MS reveals whisky’s chemical complexity, as well as the chemical diversity of whiskies from across Scotland

For this study, we curated samples to include a comprehensive cross-section of the diverse set of Scotch Whisky. In total, 85 authentic whisky samples were analyzed by FT-ICR MS (see Online Resource Supplementary Table S1 for full details of the sample set). Our sample set only included commercially available whiskies, and contains both malt (n = 55) and blended whisky (n = 30). For the malt whisky subset, samples were sourced from distilleries from all major regions of Scotch Whisky production (Lowland, Highland, Islay, Speyside). In addition, we had information on the provenance of our malt sample set (year of bottling, stated age, cask maturation wood type, and whether the whisky was peated).

**Figure 1:**
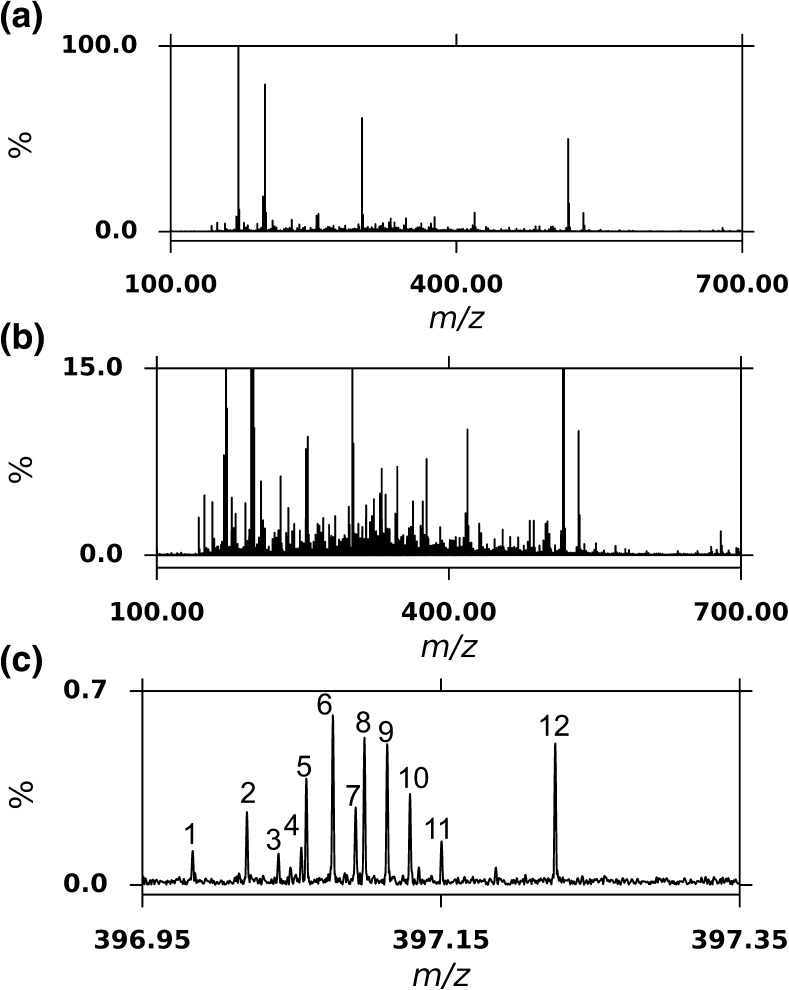
ESI(–)-FT-ICR MS spectra of sample S14-2373 showing **(a)** broadband mass spectrum between 100 and 700 *m/z*, **(b)** broadband mass spectrum between 100 and 700 *m/z* zoomed in to 15% relative abundance, **(c)** 12 peaks of the zoomed in region of 396.95– 397.35 *m/z*; identities are detailed in Table 1

An example spectrum acquired is displayed in Figure 1a–c, which was collected for sample S14-2373, a 10-y-old ex-Sherry cask matured Highland single malt whisky. The spectrum clearly demonstrates the chemical complexity within the sample with 3325 peaks observed [signal-to-noise ratio (SNR) > 4]. The spectrum was calibrated with a standard deviation of 214 ppb up to 577 *m/z*. Reflecting the complexity of this sample, many individual species were present at a single nominal *m/z* as exemplified in Figure 1c with a dozen peaks present within 0.3 *m/z* at 397 *m/z*. Eleven of these 12 peaks could be unambiguously assigned a chemical formula based on their accurate mass (Table 1). For this sample, of the 3325 peaks observed, 1902 could be assigned as monoisotopic formulae within 1 ppm error, with an average assignment error of 102.5 ± 118.3 (standard deviation) ppb and a median error of 34.5 ppb. Additionally, a further 571 peaks were assigned as ^13^C_1_ isotopologues, with an average assignment error of 93.2 ± 91.6 ppb and a median error of 67.1 ppb. Overall, in this sample we assigned 1813 CHO species and 89 CHOS species. Therefore, in total over 74% of peaks picked could be assigned, and it is likely that a significant number of the unassigned peaks were further isotopologues, include ^13^C_2_ and ^18^O_1_ species. Elemental limits for assignment were tested iteratively by including higher O and S limits with broader mass error thresholds. However, no confident assignments could be made for a species with a heteroatomic class larger than O_16_ or O_9_S in this sample, or O_19_ or O_12_S for any sample. Across the entire sample set (n = 85), there were 4271 unique formulae assigned, 82.2% were CHO species and 17.8% were CHOS species. Only 407 formulae were common to all samples, and 1201 formulae were present in over 75% of samples. In total, 44 samples identified sulfur containing formulae. Whilst CHO compounds and sulfur containing low molecular weight congeners have been identified by other techniques in Scotch Whisky [13, 17], a larger CHOS molecule, such as that assigned to peak 12 in Figure 1c as C_18_H_38_O_7_S has not been identified previously.

**Table 1:** Exact Masses and Assigned Molecular Formula with Associated Errors for Peaks Shown in Figure 1c

Number	*m/z* [M – H]^–^	Molecular Formulae	Error (ppb)
1	396.9837595	C_18_H_6_O_11_	62.7
2	397.0201095	C_19_H_10_O_10_	26.7
3	397.0412325	C_16_H_14_O_12_	42.6
4	397.0564975	C_20_H_14_O_9_	20.4
5	397.0598919	C_17_H_18_O_9_S	38.3
6	397.0776630	C_17_H_18_O_11_	70.8
7	397.0928843	C_21_H_18_O_8_	17.1
8	397.0987888	C_14_H_22_O_13_	61.7
9	397.1140314	C_18_H_22_O_10_	27.7
10	397.1292975	C_22_H_22_O_7_	52.6
11	397.1504005	C_19_H_26_O_9_	13.8
12	397.2265544	C_18_H_38_O_7_S	15.9

**Figure 2:**
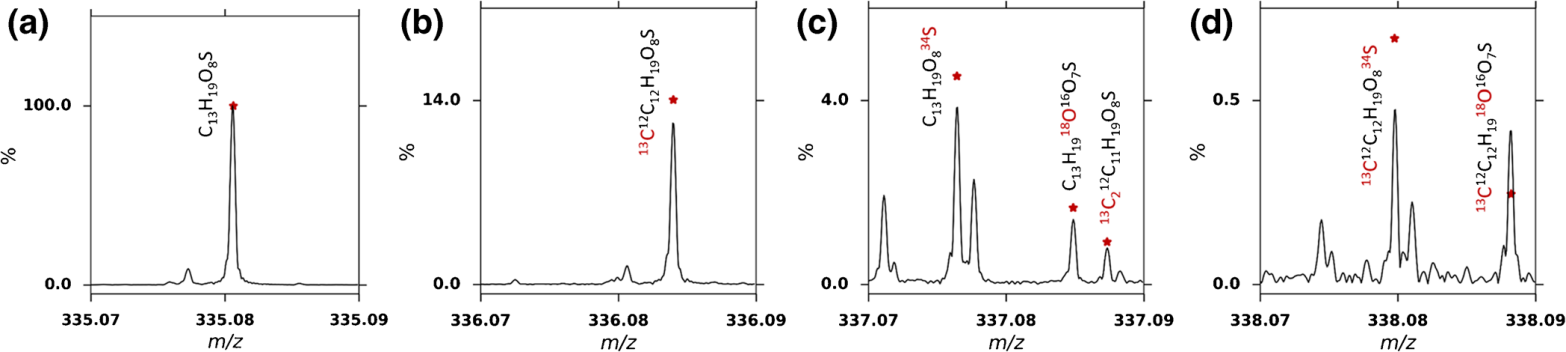
Expanded regions of a windowed mass spectrum of sample S14-2373 at the **(a)** first, **(b)** second, **(c)** third, and **(d)** fourth isotope of peak 335.08058 *m/z* with isotopic formulae labeled at red scatter points corresponding to their theoretical *m/z* and relative abundance

In order to validate and confirm the assignment of CHOS compounds, selected regions were re-examined by windowing spectral acquisition to improve the resolution and sensitivity as required for isotopic fine structure (IFS) analysis. IFS has previously been used to confirm assignments [8, 58, 59], including the use of selectively isolating ions of interest [60]. An example of this is shown in Figure 2 using the assigned formula at 335.08058 *m/z* of C_13_H_19_O_8_S identified in the S14-2373 sample. A 7 *m/z* window was isolated, a mass spectrum acquired, and calibrated against CHO species from the assignments of the broadband mass spectrum. The predicted isotopic pattern (C_13_H_19_O_8_S) was calculated and is shown as scatter points on the spectral data (Figure 2). The first isotope (Figure 2a) had an assignment error of 89 ppb, resolution of 760,500, and SNR of 13,935. This compares favorably with the original broadband spectrum parameters of 119 ppb (error), 375,000 (resolution), and 744 (SNR). Such increase in SNR allowed observation of isotopic ions at the second, third, and fourth isotope nominal *m/z*. For example, the final observed isotopologue (^13^C^12^C_12_H_19_^18^O^16^O_7_^32^S), with a natural abundance of only 0.23% of the monoisotopic form, was still observed with a resolution of 795,000 and SNR of 57. In contrast, in the broadband spectrum this peak was below the intensity threshold for peak picking. The increased resolution and SNR achieved using this technique allowed isotopologues with the same nominal mass to be clearly distinguished and to confirm the elemental composition of this molecule. This is demonstrated clearly in the third isotope signal (Figure 2c), where the intensity ratios of the O/S/C isotope peaks were compared with the predicted isotope pattern and confirm the overall elemental composition of this ion as C_13_H_19_O_8_S. This demonstrates the power and importance of windowing spectra for isotope fine structure assignments. This technique was repeated for C_30_H_46_O_7_, and two other formulae, C_18_H_29_O_3_S, and C_12_H_19_O_10_, in a different sample (S14-1908) (Online Resource Supplementary Figure S1).

### Visualizing the Complex Chemistry of Scotch Whisky

**Figure 3:**
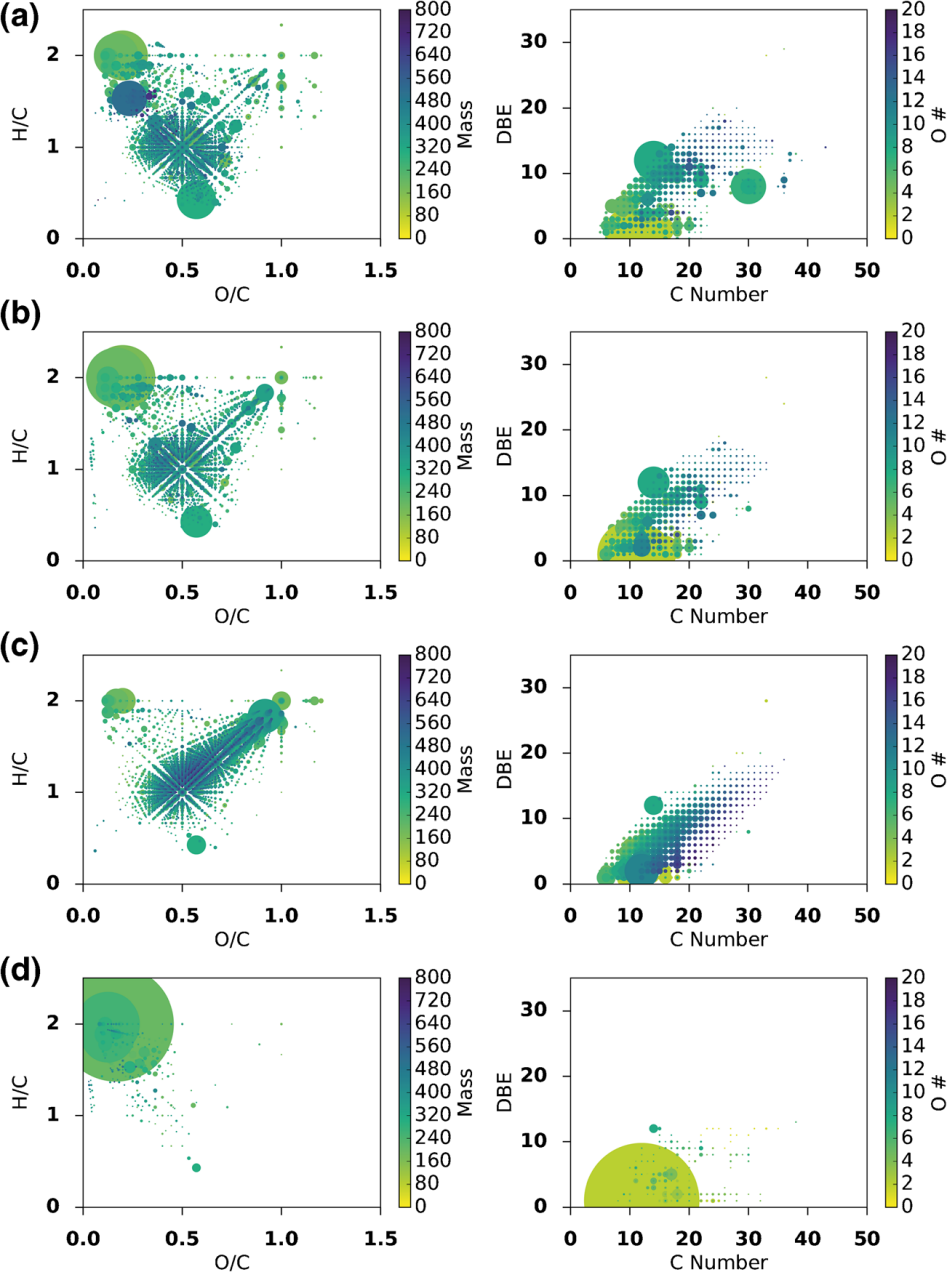
Van Krevelen (left) and DBE versus carbon number plots (right) for four different samples; (top to bottom) **(a)** S14-2373 an ex-Sherry cask matured Highland single malt, **(b)** S14-1948 an ex-Bourbon cask matured Highland single malt, **(c)** S14-1916 a blended whisky, **(d)** S11-0034, a new make spirit from a Speyside distillery. In the van Krevelen diagrams, points are colored according to mass and sized according to relative signal intensity. In the DBE versus carbon plots, points are colored according to oxygen number and sized according to relative signal intensity

The comprehensive assignment of chemical formulae in mass spectra of the studied whisky sample set (74% for sample S14-2373 and 72-88% across the entire sample set) allows the data to be visualized based on the atomic composition of the assigned species. Plots can be generated that offer a means to visually compare the chemical diversity of the studied samples. Towards this end, van Krevelen and DBE versus carbon number plots were produced as illustrated for four samples (S14-2373, S14-1948, S14-1916, and S11-0034) in Figure 3. Using the molecular formulae, the van Krevelen diagram (Figure 3 left column) sorts assigned species based on the hydrogen to carbon (H/C) versus the oxygen-to-carbon ratios (O/C). Additionally, in our representation, extra information is encoded by the size of the points reflecting the relative abundance of the original *m/z* signal and coloring the scatter plot by molecular mass of each species. It should be borne in mind that the higher relative abundance of any compound can be due to a naturally higher concentration in the sample, or a higher/lower ionization potential relative to the other components. This limitation is inherent to ESI-FT-ICR MS. In the resulting plot, major biogeochemical classes of compounds (such as lipids, carbohydrates, lignins, ellagitannins, etc.) cluster in specific characteristic regions (Online Resource Supplementary Figure S2). A comprehensive set of van Krevelen and DBE versus carbon number plots for all samples in this study can be found in the Electronic Supplementary Information.

The van Krevelen plots reveal several features that are common to all whisky samples analyzed. These include large signals for fatty acids or esters at O/C < 0.3 and H/C of 2, representing formulae including C_10_H_20_O_2_ and C_12_H_24_O_2_, compounds that may be formed during fermentation or esterification during maturation. From the central position of O/C 5 and H/C 1, there are apparent lines extending outwards in multiple directions. This region can be described as a “central star” region. In this van Krevelen, there is a significant central star region corresponding to aromatic molecules; lignin, and ellagitannin type compounds. At the center of the star are formulae such as C_8_H_8_O_4_, for example vanillic acid, a known maturation related compound. There is also a common region at O/C of 1 and H/C of 2 corresponding to carbohydrates. These peaks will represent compounds such as glucose and fructose, as well as related compounds extracted through maturation from cask heat treatment or carry over from previous fills.

Despite the similarities between the whisky samples as shown by the van Krevelen plots, it is clear that there is substantial chemical diversity amongst the samples. The van Krevelen plot for sample S14-2373 (Figure 3a), a 12-y-old ex-Sherry cask matured Highland single malt, shows a significant complexity within the central star region around O/C 0.5 and H/C 1.0. The higher abundance species towards the bottom at O/C of 0.6 and H/C of 0.4 corresponds to C_14_H_6_O_8_, an elemental formula which corresponds to ellagic acid, a known polyphenolic tannin-hydrolysis product present in Scotch Whisky [16, 61]. There is also a high abundance species at O/C of 0.2 and H/C of 1.5 that corresponds to C_30_H_46_O_7_, an unknown compound. The second sample, S14-1948, (Figure 3b) a 10-y-old ex-Bourbon cask matured Highland single malt, appears slightly less complex than the ex-Sherry cask matured sample. Interestingly, C_30_H_46_O_7_ is of significantly lower relative abundance in this sample. The third sample, S14-1916, (Figure 3c) a blended whisky, has a complex central star region. However, in this case, the complexity spreads towards H/C 2 and O/C 1. This stark difference compared with the malt samples shown may be due to the differences in production methods. Some of the major species in this region correspond to (from the top right to the center) C_6_H_12_O_6_, C_12_H_22_O_11_, C_12_H_20_O_10_, C_12_H_18_O_9_. These are likely carbohydrates or carbohydrate derivatives, possibly “caramel-type” compounds [62].

For comparison, a new-make spirit (S11-0034) was also analyzed using this methodology (Figure 2d). This sample is a non-peated new-make malt spirit from a Speyside distillery. This spectrum contained far fewer peaks than the spectra of the mature samples, with many nominal *m/z* not represented. In total, there were 1084 peaks detected, with 252 assigned monoisotopic formula and 49 isotopologues, indicating the lack of complexity. The major compounds present are fatty acid or ester type compounds, such as C_10_H_20_O_2_. These compounds will correspond to volatile congeners produced during fermentation and carried over through distillation. When compared to the three mature Scotch Whisky samples, it is abundantly clear that the complexity of Scotch Whisky reflected in their mass spectra arises through maturation.

Furthermore, as trend lines through data points on the van Krevelen plot represent the characteristic loss or gain of repeating chemical units, such as apparent methylation or oxidation, these plots have been used to visualize potential chemical transformations occurring in complex samples [39, 40]. The trend lines extending from the central star region represent chemical transformations; for example, vertical lines (e.g., H/C 2 to 1, O/C 0.5) correspond to (de)hydrogenation, diagonal lines (e.g., H/C 2 to 1, O/C 0 to 0.5) (de)methylation, and horizontal lines (e.g., H/C 1, O/C 0 to 1) oxidation/reduction. By examining a single sample, it is impossible to infer the direction of these trend lines, if indeed a direction exists. In our case, comparisons of matured whisky to the new-make spirit allow a general trend to be inferred. The trend lines correspond to an oxidative process, pointing towards the central star region, where the types of compounds present are increasingly aromatic, unsaturated, and oxygen-rich. This change in chemistry may be due to chemical transformations within the new-make spirit; however, it is likely that the majority of changes are due to extraction of additional compounds from the cask.

The chemical diversity can also be represented by assigning to each molecular formulae its DBE. The DBE versus carbon number plots (Figure 3 right column) demonstrate similar trends in the samples as observed with the van Krevelen plots. The ex-Sherry cask matured sample (Figure 3a right) and the ex-Bourbon matured sample (Figure 3b right) have largely similar DBE plots, with the relatively high abundance compound C_30_H_46_O_7_ being the most significant difference between them. As with the van Krevelen plot, the blended whisky (Figure 3c right) has a distinctly different DBE plot to the other samples. The DBE plot of the new-make spirit is sparse, with the major components having a very low DBE and carbon number of around 10 to 15.

### Heteroatomic Class Distributions

The translation of molecular formulae alone into chemical structures is not possible, as any given formula can correspond to many possible structures. Using the S14-2373 sample as an example, its spectrum had 1902 unique formulae assigned. These formulae were cross-referenced against the ChemSpider (The Royal Society of Chemistry) database and it was found that 1735 formula had at least one known structure in the database and, in total, over 320,000 possible known structures were found. With many of the higher mass compounds having more theoretically possible combinations, this number is likely a significant underestimate [63]. That being said, based on a priori knowledge of compounds present in Scotch Whisky, it is possible to propose identities for some peaks in the spectrum; for example, a large peak at 300.99897 *m/z* corresponds to C_14_H_5_O_8_ ([M – H]^–^), which is likely ellagic acid. Further identifications are proposed in Table 2, with molecular formulae assignments also made by Garcia et al. highlighted with an asterisk [22]. Note that all but one of the ESI-(–) assignments by Garcia et al. could be found here in a single sample.

**Table 2:** Subset of Molecular Formulae Assigned in Sample S14-2373 Along with Associated Absolute Errors of Assignment in ppb and Possible Compound Identification Based on Known Chemistry of Scotch Whisky

*m/z* [M – H]^–^	Error (ppb)	Molecular formula	Possible compound ID
171.1390419	67.3	C_10_H_20_O_2_	Ethyl octanoate / decanoic acid*
199.1703420	58.0	C_12_H_24_O_2_	Ethyl decanoate / dodecanoic acid*
300.9989723	61.0	C_14_H_6_O_8_	Ellagic Acid*
197.0455386	42.7	C_9_H_10_O_5_	Syringic Acid*
169.0142383	50.3	C_7_H_6_O_5_	Gallic Acid
227.2016473	28.3	C_14_H_28_O_2_	Ethyl Dodecanoate*
207.0662751	35.2	C_11_H_12_O_4_	Sinapaldehyde
181.0506241	45.5	C_9_H_10_O_4_	Synringaldehyde
283.2642638	34.7	C_18_H_36_O_2_	Ethyl Hexadecanoate
179.0561001	64.5	C_6_H_12_O_6_	Monosaccharide
177.0557077	56.8	C_10_H_10_O_3_	Coniferaldehyde
191.0349739	43.7	C_10_H_8_O_4_	Scopeletin
281.2486228	67.4	C_18_H_34_O_2_	Ethyl-9-Hexadecenoate
167.0349732	54.3	C_8_H_8_O_4_	Vanillic Acid
341.1089493	41.8	C_12_H_22_O_11_	Disaccharide*
151.0400608	45.6	C_8_H_8_O_3_	Vanillin
155.1077472	39.1	C_9_H_16_O_2_	Whisky Lactone

*Asterisks indicate assignments also observed in Garcia et al. [22]

**Figure 4:**
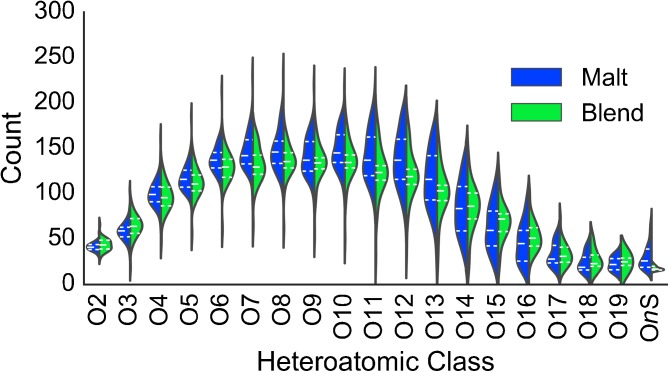
Violin plot for heteroatomic class distributions for the entire sample set. Malts are in blue and blends are in green. The shapes of the plots represent the kernel density estimate of the distribution of counts across the sample set; quartiles are represented with white dotted lines within the plots. Each violin has been scaled to the same width. Sulfur containing heteroatomic classes are summarized in the single OnS class

In the absence of a rigorous automated and practical procedure for identifying structures associated with individual molecular formulate, it is still possible to examine the makeup of a complex mixture as a whole, for example by inspecting the heteroatomic class distribution within a sample or set of samples. Figure 4 shows the heteroatomic class distribution calculated for the entire set of Scotch Whiskies, comparing the chemistry of malt and blend whisky. Here the data are represented as a violin plot, which shows the distribution of the counts of heteroatomic classes across the type of samples, e.g., the number of compounds with the heteroatomic class O2 within the malt whisky type. The distributions were calculated as a kernel density estimate using the Silverman kernel bandwidth rule of thumb.

Overall, the general trend is a unimodal distribution centered at around O_9_ for blends, with the malts’ distribution centered around O_10_. It is evident that generally, the malt whiskies have a higher count at each heteroatomic class than the blend whiskies. Additionally, there is a larger spread of counts around the middle heteroatom classes, with the upper and lower extremes having narrower distributions. Within each heteroatomic class, the blends generally have a narrower unimodal distribution, whereas the malts have a larger distribution. Furthermore, in several cases, this distribution is (at least) bimodal, for example for O_17_. Interestingly, the O*n*S class is distinctly different between malts and blends, with a much narrower and smaller distribution in blends than in malts. The total counts for this class are also low relative to other heteroatomic classes. The distribution of the individual CHOS classes across the sample set is shown in Supplementary Figures S3–S4. The heteroatomic class distribution violin plot shows that it is possible to differentiate between types and suggests that the classification of whisky may be possible with statistical analysis of the mass spectral data.

### Multivariate statistical analysis of the FT-ICR MS data of whisky can discriminate malts and blends and highlights the consistency of the blending process year on year

**Figure 5:**
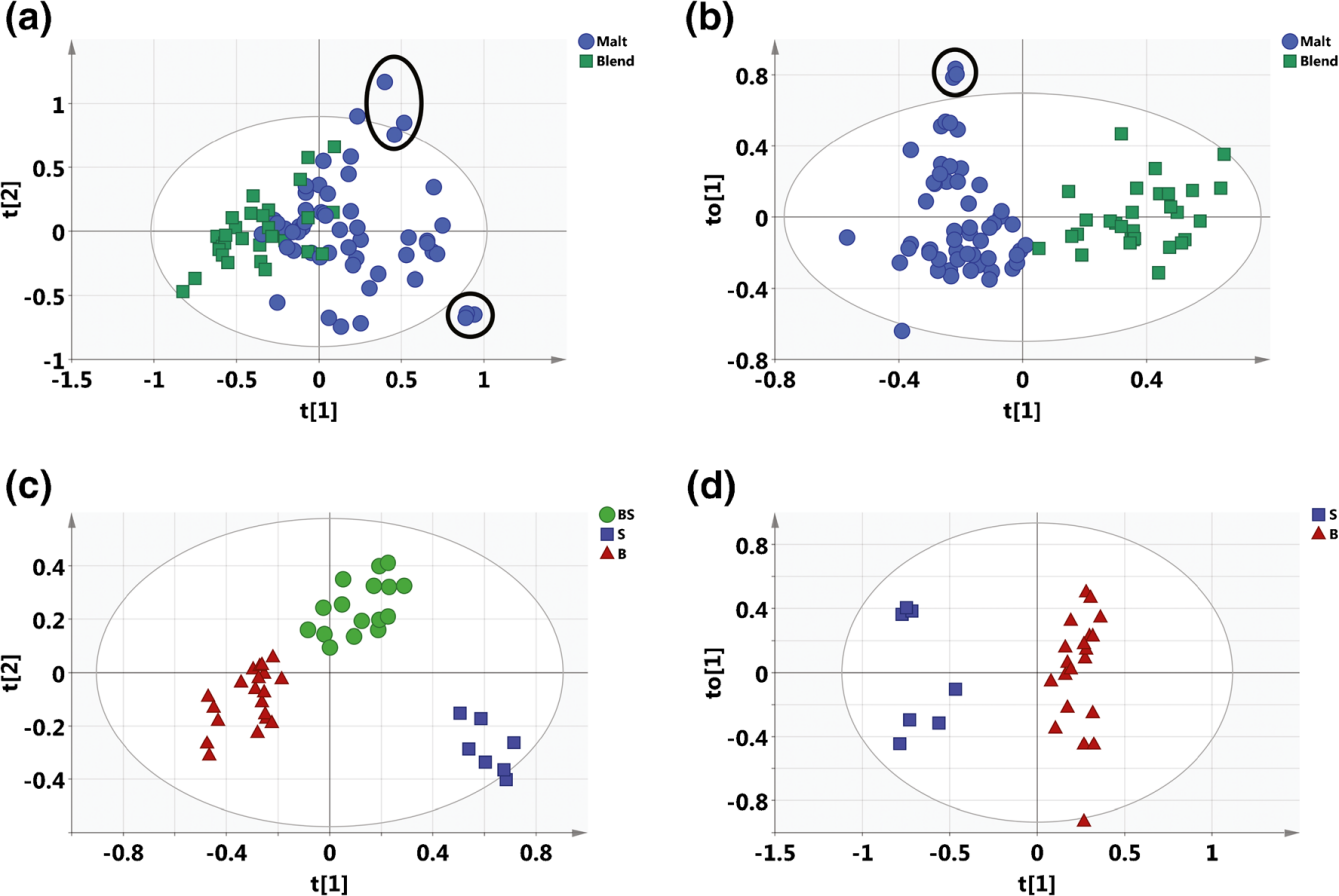
Scores plots for statistical models built around samples. **(a)** PCA scores plot for all Scotch Whisky samples analyzed (n = 85) colored according to malt (blue) or blend (green). Two examples of samples of the same whisky product from differing production years are circled. The bottom circle represents samples discussed in Figure 6, and the top circle represents samples shown in Online Resource Supplementary Figure S5. **(b)** OPLS-DA Scores plot for the Malt (blue) versus Blend (green) (n = 85) model. Three samples of the same whisky product from different production years are circled. **(c)** OPLS-DA scores plot for a model based on malts with known maturation wood type (n = 43). BS (green) = ex-Bourbon and ex-Sherry, S (red) = ex-Sherry, B (blue) = ex-Bourbon. **(d)** OPLS-DA model for malts matured in ex-Sherry only or ex-Bourbon only casks (n = 27), S (red) = ex-Sherry, B (blue) = ex-Bourbon

A PCA model (Figure 5a) was constructed from 85 samples across 2010, 2012, and 2014 production years. Using the method as described in the Experimental section, this model was constructed with nine principal components describing 85% of the data, with the first two representing 50% of the variance in the data. The blends appear more closely clustered compared with the malts, which are more disparate. Several of the malts are outliers, with the three outliers in the top right being peated Islay single malts, two of which were the same product from 2 y (2012 and 2014), and the third was a single malt from an unknown distillery on Islay. The bottom right three outliers were all the same product, from 3 different y (S10-1218, S12-1293, S14-1941); an ex-Sherry cask matured Highland single malt from 2010, 2012, 2014. We attribute the larger spread of malts compared with blends to the greater variety within malt products than blended products. This observation is consistent with the heteroatomic class distribution analysis, which showed greater diversity in the malt sample type. The lack of distinct clustering, however, demonstrates that there are more variables in whisky production and type than just classification as malt and blend that are affecting the mass spectra. The loadings scatter plot is included in the Supplementary Information (Online Resource Supplementary Figure S7), and highlights a number of carbohydrate type formulae for blends and cask extractive type compounds for malts. The relative abundance of these compounds may be a measure of whisky classification. Furthermore, from the loadings plot it can be calculated that the first principal component separation is based on H/C ratio going from high (2) to low (1), where the values quoted in brackets are approximate averages; at the same time, the O/C ratio remains high (1), whereas positive positions on the second principal component are due to formula with high H/C (2) and low O/C (0.2) ratios.

An OPLS-DA model was required to better distinguish between the malts and blends. The malt versus blend model successfully distinguished these two whisky types, with a scores plot of the predictive component versus the first orthogonal component shown in Figure 5b. This model was built with four orthogonal components with an R^2^X of 0.64, R^2^Y of 0.83, with a cumulative Q^2^ of 0.61. The S-plot (Online Resource Supplementary Figure S8) highlights several formulae significant to discriminating between malt and blended whiskies; these are listed in Table 3. These blends are discriminated by having a higher relative abundance of a number of carbohydrate-type species, whereas the malts have a higher relative abundance of cask extractive compounds. The underlying differences between blends and malts are multiple; grain whisky is distilled to a higher alcohol strength, producing a lighter spirit; malt whiskies are a premium product and may be matured for longer in a more active cask. Interestingly, this model placed one blended whisky (S14-2858) very near the malts. Upon further analysis, this blended sample was found to represent a premium blended whisky with a high malt content. The three closely positioned single malt outliers (Figure 5b, top left, circled) represent the same samples grouped as the bottom right outliers in the PCA plot (Figure 5a).

**Table 3:** Key Variables (Formulae) Discriminating Between Malts and Blends for the OPLS-DA Model. “Carbohydrate” Includes Derivative or Related Compounds

Variable ID	Possible identity	Discriminates type
C_9_H_10_O_5_	Syringic Acid	Malt
C_14_H_6_O_8_	Ellagic Acid	Malt
C_30_H_46_O_7_	Unknown	Malt
C_7_H_6_O_5_	Gallic Acid	Malt
C_12_H_20_O_10_	Carbohydrate	Blend
C_12_H_22_O_11_	Carbohydrate	Blend
C_9_H_16_O_8_	Carbohydrate	Blend
C_12_H_18_O_9_	Carbohydrate	Blend

**Figure 6:**
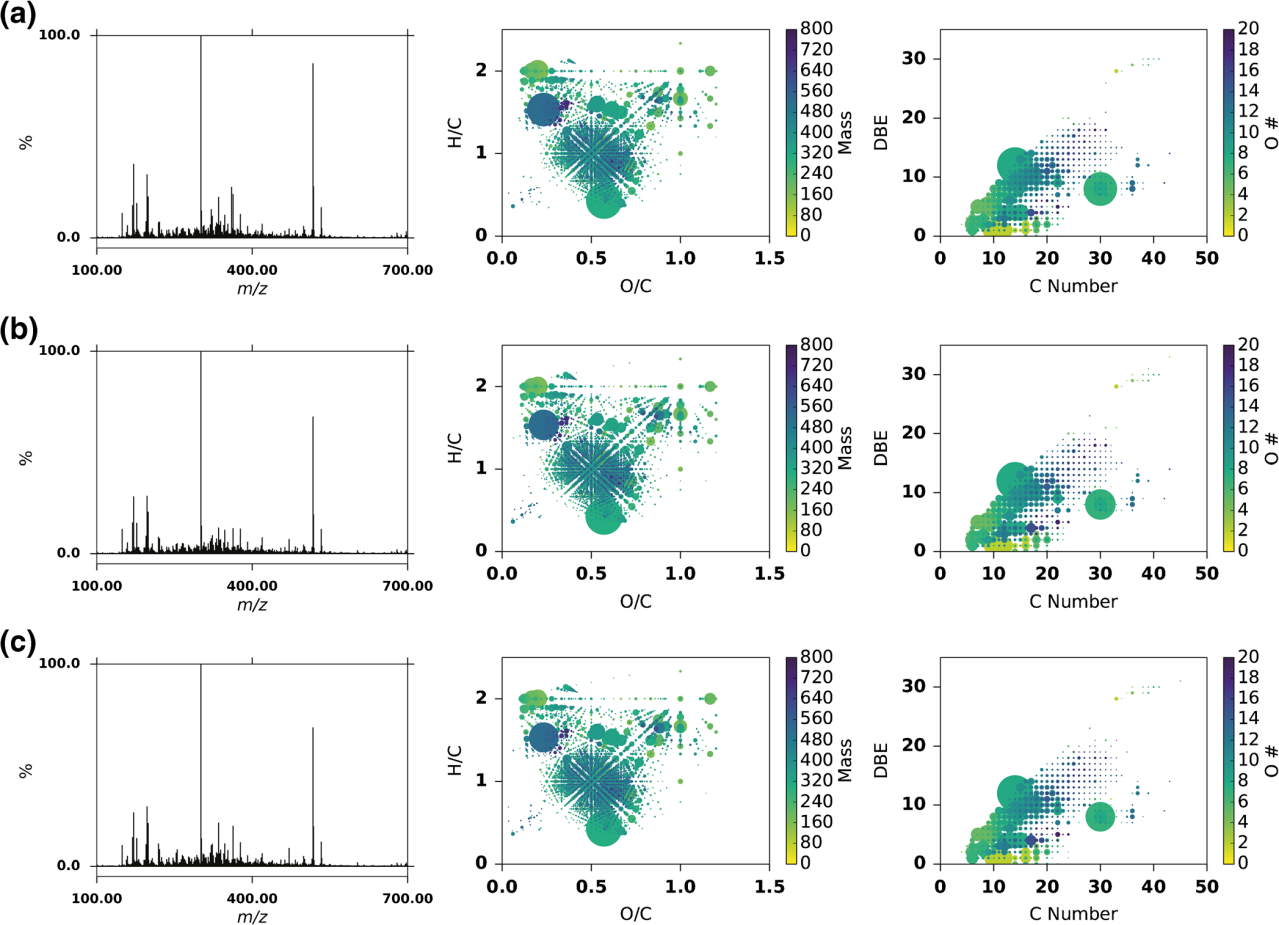
Samples of the same Scotch Whisky product from 3 y S10-1218 **(a)**, S12-1293 **(b)**, S14-1941 **(c)**, a 12-y-old ex-Sherry cask matured Highland single malt. The left column shows the broadband mass spectra for each sample, the middle column shows a van Krevelen diagram colored according to mass and sized according to normalized relative abundance, the right column shows a plot of carbon number versus double bond equivalent colored according to oxygen number and sized according to normalized relative abundance

These three well-clustered outliers were examined further to investigate how consistent the production of these whiskies was over the 3 y and their mass spectra, van Krevelen and DBE plots were directly compared (Figure 6). It is clear that the mass spectral data are strikingly similar, with the same dominant peaks corresponding to C_14_H_6_O_8_ and C_30_H_46_O_7._ Additionally, two species C_10_H_20_O_2_ and C_12_H_24_O_2_ occur with similar relative intensity and above average intensity. This product similarity is also highlighted by the van Krevelen and DBE versus C# plots, which are also superficially identical. In order to quantify this similarity, the standard deviation (population) was calculated for the normalized relative abundance of each formula across the three samples. The average standard deviation was 0.003% of the total normalized relative intensity, with a maximum standard deviation of 0.45% of the total normalized relative intensity. For comparison, the same values calculated across all 85 samples were 0.015% and 3.13%, respectively. Furthermore, the three products had 2826 unique formula across all three samples with 2208 (78%) of those common to all three, whereas with the entire sample set there were 4271 unique formulae across the samples with only 407 (9.5%) common to all. Therefore, it can be seen that the ESI-FT-ICR MS profile of this product remains consistent across 3 production y. Our sample set contained two more products produced across 3 y. Again, these samples displayed a high degree of similarity (Online Resource Supplementary Figures S5 and S6).

These observations highlight the consistency of the blending process undertaken during whisky production. Whisky is blended, based on its sensory profile, to achieve a consistent product year on year; however, the similarity of the ESI-FT-ICR MS profiles of these samples suggest that a high degree of chemical consistency is also achieved by master blenders. This in itself is not surprising, as the sensory profile is inherently chemically based; however, such outcome in whiskies is thought to be related to around 80 volatile aroma compounds [64, 65]. Here we observed similarities across hundreds or thousands of compounds, suggesting a correlation between sensory profile and a far broader range of compounds than previously known.

### Multivariate Statistical Analysis of the FT-ICR MS Data of Scotch Whisky can Reflect the Casks Used in Maturation

Based on our analysis thus far, we hypothesised that it was possible to model some of the variables that contribute to the sensory profile such as maturation wood type against the mass spectra. OPLS-DA models were thus constructed to attempt to model a number of provenance variables.

#### Regions, Peating, and Age

An OPLS-DA model was built for samples of known geographical region (n = 54) (Online Resource Supplementary Figure S9); however, there was limited predictive power with a Q^2^ of only 0.20. This is to be expected, given the limited impact the geographical location will have on the whisky production, as there is no geographical bias for wood types, or other dominant production style. The exception may perhaps be the peated whiskies with a dominant flavor profile, traditionally associated with Islay whiskies. However, peating malt for whisky production is not strictly limited to this region of Scotland; in fact, the Islay region produces some non-peated whiskies, and several malts from the Highland region are peated.

Next, an OPLS-DA model was constructed for peated and non-peated samples from the 2014 standard sample set (n_peated_ = 13, n_non-peated_ = 18) (Online Resource Supplementary Figure S10). The predictive power of this model was also limited, with a Q^2^ of 0.14.

A model was also constructed for age statements; however, it is of limited use as the final product age statement reflects only the youngest component in the bottle, and only two age statements with enough samples to model were present in the sample set (10- and 12-y-old) (Online Resource Supplementary Figure S11).

#### Wood Types

An OPLS-DA model was built for the malt samples across all years with known maturation wood type (n = 43) (Figure 5c). Three classes were considered— whiskies that had been matured in ex-Bourbon casks only (B), ex-Sherry casks only (S), or a mixture of ex-Bourbon and ex-Sherry casks (BS). This model had two predictive components and four orthogonal components with an R^2^X of 0.73, R^2^Y of 0.86, and Q^2^ of 0.61. As can be seen in the scores plot, the first predictive component (x-axis) comprehensively separates class S from B, and the second predictive component (y-axis) separates the S and BS classes. The separation of B and BS classes is smaller, possibly due to some of BS samples only being finished in an ex-Sherry cask, rather than matured more evenly (over time) between wood types.

To understand the discrimination between ex-Sherry and ex-Bourbon cask maturation, a further model was built using just the B and S classes only (n = 27) (Figure 5d). This model had one predictive component and two orthogonal components with an R^2^X of 0.65, R^2^Y of 0.96, and a Q^2^ of 0.80. Despite the small S class sample size, the model clearly discriminated between the two classes. Additionally, the ex-Sherry cask matured samples are clustered into two groups on the first orthogonal component (y-axis); the top cluster represents the same product from different years, the bottom four are two different products from 2 y. The ex-Bourbon cask matured samples are more spread across this component, representing larger variation found in the larger sample size.

To identify the variables (formula) discriminating between the B and S classes, an S-plot was produced (Online Resource Supplementary Figure S12), which shows major contributors and their formulae highlighted. These variables are summarized in Table 4.

**Table 4:** Summary of Key Variables Discriminating ex-Bourbon and ex-Sherry Cask Matured Scotch Whiskies Based on an OPLS-DA S-Plot (Online Resource Fig. S12)

Variable ID	Possible identity	Discriminates wood type
C_30_H_46_O_7_	Unknown	Sherry
C_14_H_6_O_8_	Ellagic Acid	Sherry
C_13_H_20_O_8_S	Unknown	Sherry
C_6_H_10_O_6_	Glucono delta-lactone	Sherry
C_15_H_24_O_8_S	Unknown	Sherry
C_30_H_46_O_8_	Unknown	Sherry
C_7_H_6_O_5_	Gallic Acid	Sherry
C_9_H_10_O_5_	Syringic Acid	Sherry
C_10_H_20_O_2_	Decanoic Acid	Bourbon
C_12_H_24_O_2_	Dodecanoic Acid	Bourbon
C_12_H_22_O_11_	Disaccharide	Bourbon
C_16_H_32_O_2_	Hexadecanoic Acid	Bourbon
C_16_H_30_O_2_	Hexadec-9-enoic acid	Bourbon
C_12_H_20_O_10_	Carbohydrate	Bourbon
C_14_H_28_O_2_	Tetradecanoic acid	Bourbon

**Figure 7:**
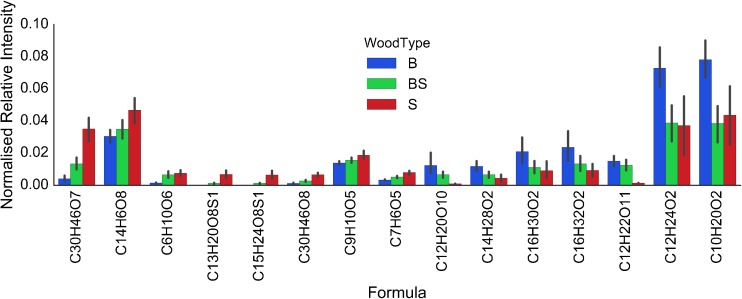
Bar plot of discriminating formula against normalized relative intensity within three classes of samples—ex-Bourbon cask only matured (B, blue), ex-Bourbon and ex-Sherry cask matured (BS, green), and ex-Sherry cask only matured (S, red). Black lines indicate 95% confidence intervals. Formulae are ordered according to their location on the OPLS-DA S-Plot for B versus S class (Online Resource Supplementary Figure S12)

A bar plot for the relative abundances of the formulae summarized in Table 4 is shown in Figure 7, classified per maturation wood type, including the BS class. From this bar plot, it is seen that the samples matured in both ex-Bourbon and ex-Sherry casks generally show abundances of these compounds in between that of ex-Bourbon or ex-Sherry cask only matured whiskies. Based on known chemistry of Scotch Whisky, possible identities of some of the major discriminating formulae include mono- and disaccharides (C_6_H_10_O_6,_ C_12_H_22_O_11_, C_12_H_20_O_10_), and fatty acids, or esters (C_10_H_20_O_2_, C_12_H_24_O_2_). Identities of C_13_H_20_O_8_S, C_15_H_24_O_8_S, and C_30_H_46_O_7_ are not known. Strikingly, C_12_H_22_O_11_ appears to be nearly absent in ex-Sherry cask matured whiskies, whilst noticeable in ex-Bourbon cask matured samples. This formula corresponds to a disaccharide composed of two hexoses. Sulfur containing species, such as C_13_H_20_O_8_S, are also strong discriminating factors with this compound not found in any ex-Bourbon cask only matured whiskies. In contrast, B class samples have a higher relative abundance of several O2 class compounds, likely fatty acids or esters. C_30_H_46_O_7_ is the highest relative abundance compound that clearly discriminates ex-Sherry from ex-Bourbon cask matured whiskies; however, its structure is not known, and its presence has not been reported in Scotch Whisky before. As such, this became a target for further investigation.

### Fragmentation of C_30_H_46_O_7_

The observation of a species with formula C_30_H_46_O_7_ has been reported previously, during an LC-MS study of Irish Whiskey [66], with the chromatography suggesting that two structural isomers were present. MacNamara et al. [66] performed MS/MS analysis, and several major fragments were observed; however, no structure was proposed. Owing to the evident interest in this compound, its fragmentation was repeated in this study.

The species of 517 *m/z* in sample S14-1941 were isolated using the quadrupole and fragmented by CID in the collision cell of the FT-ICR MS; the fragmentation spectra are presented in Online Resource Supplementary Figure S13. The major fragmentation peaks observed are summarized in Table 5 and are consistent with those observed by MacNamara et al. [66].

**Table 5:** Fragmentation Peaks Observed for Molecular Ion C_30_H_45_O_7_. Note That Mass Spectra Were Not Calibrated Post-Acquisition, Which Accounts for the Larger Errors

MS^n^	*m/z*	Ion formula [M-H]^-^	Fragmentation	Error (ppm)
MS	517.31708	C_30_H_45_O_7_	M-H	1.43
MS^2^ (517)	499.30591	C_30_H_43_O_6_	M-H-H_2_O	1.21
	473.32671	C_29_H_45_O_5_	M-H-CO_2_	1.14
	455.31610	C_29_H_43_O_4_	M-H-CO_2_-H_2_O	1.28
	437.30559	C_29_H_41_O_3_	M-H-CO_2_-H_2_O-H2O	1.21
MS^3^ (517, 455)	455.31608	C_29_H_41_O_3_	M-H-CO_2_-H_2_O	1.32
	437.30612	C_29_H_41_O_3_	M-H-CO_2_-H_2_O-H_2_O	1.25
	407.29504	C_28_H_39_O_2_	M-H-CO_2_-H_2_O-H_2_O-H_2_O	1.27
	393.27939	C_27_H_37_O_2_	M-H-CO_2_-H_2_O-H_2_O-H_2_O-CH_2_	1.31
	379.30014	C_27_H_39_O	M-H-CO_2_-H_2_O-H_2_O-H_2_O-CH_2_-H_2_O	1.32

The fragments observed showed loss of H_2_O and CO_2_ units, with some CH_2_ loss in the MS^3^ experiment. This pattern suggests a number of cleavable hydroxyl and carboxyl groups, along with terminal methyl functionality. Unfortunately, full structural elucidation was not possible; however, cross-referencing the ChemSpider database suggests several triterpenoid derivatives. Based on our MS^n^ data of this species and structures in the literature for the same or similar formula [67, 68], we suggest that this compound is an oleanane-type triterpenoid.

Further examination of the mass spectrum (S14-1941) reveals additional compounds, including C_30_H_46_O_8_, a hydroxylated equivalent, and C_36_H_56_O_12_, a glucosylated equivalent of C_30_H_46_O_7_. Compounds such as glucosylated bartogenic acid (Glu-BA, C_36_H_56_O_12_) have been shown to exist in *Quercus robur*, a white oak species [69], and bartogenic acid (BA) itself has been found in oak wood extracts. Therefore, the identity of this unknown compound is likely bartogenic acid or a structurally similar isomer. Future work could include fragmentation by different means, including infrared multiphoton dissociation (IRMPD) or in-cell fragmentation [70]; alternative confirmation of this identity could be confirmed with isolation of the compound using preparative HPLC and structure elucidation by nuclear magnetic resonance (NMR).

## Conclusions

High-resolution mass spectrometry has revealed the chemical complexity of Scotch Whisky, allowing observation of thousands, rather than hundreds, of compounds as traditionally associated with this product. We show that this complexity arises through cask-maturation, as demonstrated by comparison of mature samples to a new-make spirit. The exact chemical processes that take place during maturation remain unknown, but the results obtained here present a route towards developing an understanding of the maturation chemistry. Potential chemical markers for production methods have been discovered, including those reflecting the maturation wood types. The origin of many of the identified formulae remains unclear, and future work will investigate time series of maturation cask samples to further understand processes in maturation, including their kinetics. FT-ICR MS is shown to be a powerful instrument for the analysis of Scotch Whisky, and this technique could be of great use in the investigation of other mature spirit drinks and other complex mixtures. Raw and processed FT-ICR MS data for the 85 Scotch Whisky samples is available online at doi:10.7488/ds/1486.

## Electronic Supplementary Material

Below is the link to the electronic supplementary material.

ESM 1 (DOCX 2066 kb)

ESM 2 (PDF 13513 kb)

ESM 3 (PDF 13686 kb)
