# Supplementary material for: Chemical Diversity and Complexity of Scotch Whisky as Revealed by High-Resolution Mass Spectrometry
Source: J Am Soc Mass Spectrom. 2016 Oct 17;28(1):200–13. doi: 10.1007/s13361-016-1513-y (PMC5174148; doi:10.1007/s13361-016-1513-y)
Supplement: Supplementary file 1 — (DOCX 2066 kb) [file 13361_2016_1513_MOESM1_ESM.docx]

Chemical Diversity and Complexity of Scotch Whisky as Revealed by High-Resolution Mass Spectrometry

W. Kew, I. Goodall, D. Clarke and D. Uhrín

Journal of the American Society for Mass Spectrometry

Corresponding Authors:

- Dr David Clarke, EaStCHEM, School of Chemistry, Joseph Black Building, University of Edinburgh, Edinburgh, UK, EH9 3FJ, Tel: +44(0)131 650 4808, email: david.clarke@ed.ac.uk
- Dr Dušan Uhrín, EaStCHEM, School of Chemistry, Joseph Black Building, University of Edinburgh, Edinburgh, UK, EH9 3FJ, Tel: +44(0)131 650 4742, email: dusan.uhrin@ed.ac.uk

Supplementary Information

Contents

[Table S1 - Sample List 2](#_Toc453594901)

[Table S2 - Calibration List 3](#_Toc453594902)

[Isotope Fine Structure (IFS) Analysis for Three Formulae 4](#_Toc453594903)

[Van Krevelen Map 5](#_Toc453594904)

[Heteroatomic Class Distributions 6](#_Toc453594905)

[Reproducibility in Scotch Whisky Production, Year on Year 8](#_Toc453594906)

[Multivariate Analysis 10](#_Toc453594907)

[PCA Loadings Plot 10](#_Toc453594908)

[OPLS-DA Model for Malt vs Blend Classification 11](#_Toc453594909)

[OPLS-DA Model for Regional Classification 12](#_Toc453594910)

[OPLS-DA Model for Peated vs Unpeated Classification 13](#_Toc453594911)

[OPLS-DA Model for Age Statements of Scotch Whisky 14](#_Toc453594912)

[OPLS-DA Model for Wood Types During Maturation 15](#_Toc453594913)

[Fragmentation Study of Species C_30_H_46_O_7_ 16](#_Toc453594914)

# Table S1 - Sample List

Table S1 - List of samples analysed and information known, Total Wood indicates wood type for maturation and or finishing, if known. BS = Bourbon and Sherry, S = Sherry only, B = Bourbon only.

| **Sample** | **Class** | **Region** | **Peated** | **Total**  **Wood** | **Sample** | **Class** | **Region** | **Peated** | **Total**  **Wood** |
| --- | --- | --- | --- | --- | --- | --- | --- | --- | --- |
| S10-1016 | Malt | Speyside |  |  | S14-1944 | Blend |  |  |  |
| S10-1017 | Malt | Islay | peated | BS | S14-2079 | Blend |  |  |  |
| S10-1019 | Malt | Highland |  | S | S14-2080 | Blend |  |  |  |
| S10-1020 | Malt | Islay | peated |  | S14-2082 | Blend |  |  |  |
| S10-1022 | Malt | Highland |  |  | S14-2086 | Blend |  |  | B |
| S10-1023 | Malt | Highland |  | B | S14-2087 | Blend |  |  |  |
| S10-1127 | Malt | Islay | peated | B | S14-2088 | Blend |  |  |  |
| S10-1131 | Malt | Highland | peated | S | S14-2195 | Blend |  |  |  |
| S10-1133 | Blend |  |  |  | S14-2335 | Blend |  |  |  |
| S10-1180 | Malt | Highland |  | B | S14-2336 | Blend |  |  |  |
| S10-1183 | Malt | Highland |  |  | S14-2338 | Blend |  |  |  |
| S10-1218 | Malt | Highland |  | S | S14-2372 | Blend |  |  |  |
| S10-1306 | Malt | Highland |  |  | S14-2375 | Blend |  |  |  |
| S10-1313 | Malt | Lowland |  | BS | S14-2816 | Blend |  |  |  |
| S10-1314 | Malt | Lowland |  | B | S14-2817 | Blend |  |  |  |
| S10-1315 | Malt | Islay | peated |  | S14-2818 | Blend |  |  |  |
| S10-1408 | Malt | Highland |  | B | S14-2857 | Blend |  |  |  |
| S10-1509 | Malt | Speyside |  | BS | S14-2858 | Blend |  |  |  |
| S10-1510 | Malt | Speyside |  | BS | S14-1906 | Malt | Islay | peated | B |
| S10-1849 | Malt | Islay | peated | B | S14-1908 | Malt | Speyside |  |  |
| S10-1850 | Malt | Highland |  | B | S14-1913 | Malt | Islay |  | BS |
| S10-1851 | Malt | Highland |  | BS | S14-1920 | Malt | Speyside |  | B |
| S10-2055 | Malt | Speyside |  | B | S14-1941 | Malt | Highland |  | S |
| S10-2058 | Malt | Speyside |  | BS | S14-1942 | Malt | Highland | peated | S |
| S12-0275 | Malt | Speyside |  |  | S14-1947 | Malt | Islay | peated | B |
| S12-1147 | Malt | Islay |  | BS | S14-1948 | Malt | Highland |  | B |
| S12-1240 | Malt | Speyside |  | BS | S14-1962 | Malt | Islay | peated |  |
| S12-1292 | Blend |  |  |  | S14-1963 | Malt | Lowland |  | B |
| S12-1293 | Malt | Highland |  | S | S14-1964 | Malt | Lowland |  | BS |
| S12-1485 | Malt | Islay | peated | B | S14-1972 | Malt | Highland |  | BP |
| S12-2514 | Malt | Highland |  |  | S14-2081 | Malt | Highland |  | B |
| S13-0090 | Malt | Lowland |  | BS | S14-2083 | Malt | Islay | peated | B |
| S13-0091 | Malt | Lowland |  | B | S14-2085 | Malt |  |  |  |
| S14-1907 | Blend |  |  |  | S14-2089 | Malt | Speyside |  | BS |
| S14-1909 | Blend |  |  |  | S14-2090 | Malt | Speyside |  | B |
| S14-1911 | Blend |  |  |  | S14-2196 | Malt | Highland |  | B |
| S14-1914 | Blend |  |  |  | S14-2319 | Malt | Speyside |  | BS |
| S14-1915 | Blend |  |  |  | S14-2337 | Malt | Highland |  | B |
| S14-1916 | Blend |  |  |  | S14-2373 | Malt | Highland |  | S |
| S14-1919 | Blend |  |  |  | S14-2374 | Malt | Islay | peated |  |
| S14-1939 | Blend |  |  |  | S14-2815 | Malt | Speyside |  | B |
| S14-1940 | Blend |  |  |  | S14-2856 | Malt | Speyside |  | BS |
| S14-1943 | Blend |  |  |  |  |  |  |  |  |

# Table S2 - Calibration List

Table S2 - Calibration List for Negative ESI

| **[M-H]^-^** | ***m/z*** | **z** |
| --- | --- | --- |
| C10H19O2 | 171.139050 | -1 |
| C12H23O2 | 199.170350 | -1 |
| C9H17O9 | 269.087810 | -1 |
| C14H5O8 | 300.998990 | -1 |
| C18H35O4 | 315.254080 | -1 |
| C14H27O8 | 323.171140 | -1 |
| C16H31O8 | 351.202440 | -1 |
| C18H35O8 | 379.233740 | -1 |
| C24H47O4 | 399.347980 | -1 |
| C15H27O14 | 431.140630 | -1 |
| C18H29O15 | 485.151190 | -1 |
| C30H45O7 | 517.317080 | -1 |
| C26H29O14 | 565.156280 | -1 |
| C27H29O14 | 577.156280 | -1 |
| C27H33O16 | 613.177410 | -1 |

# Isotope Fine Structure (IFS) Analysis for Three Formulae

Figure S1 - IFS for C_30_H_45_O_7_ (Sample S14-2373), C_18_H_29_O_3_S and C_12_H_19_O_10_ (Sample S14-1908). Red scatter point marks indicate location of theoretical isotopologue peaks at predicted relative abundances. Monoisotopic peaks are shown on the far left, followed by the second, third, and fourth isotope regions.


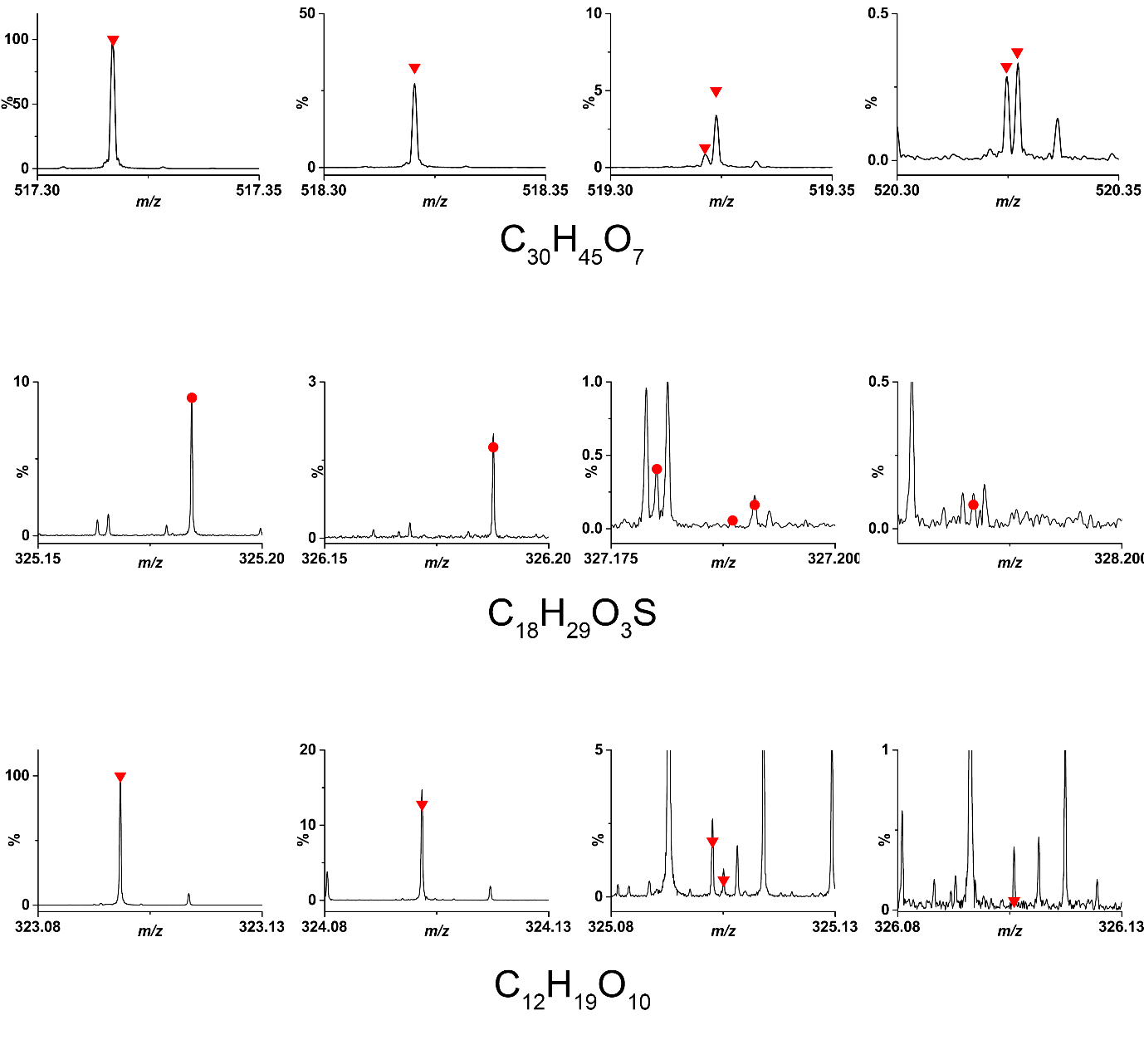


# **
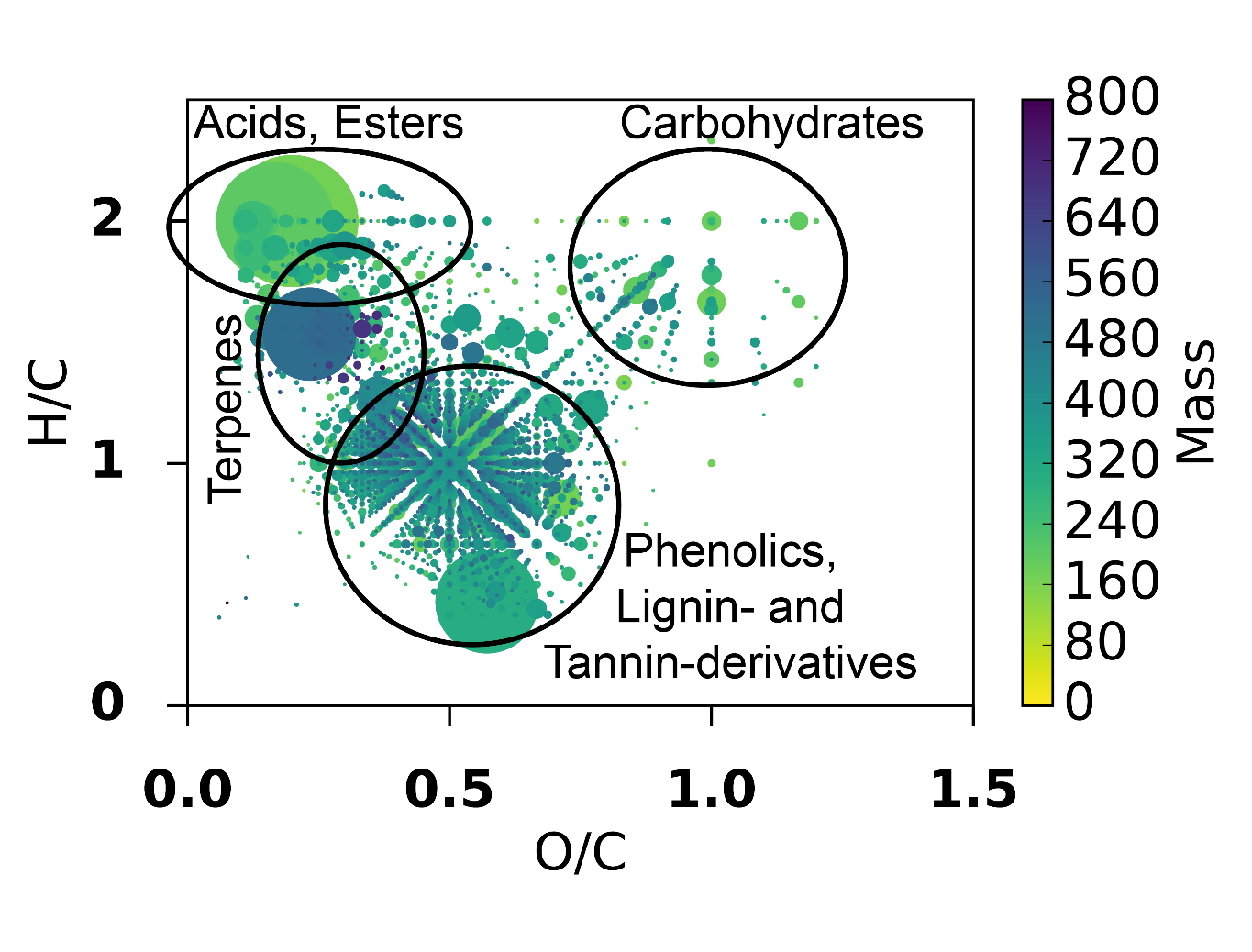
**Van Krevelen Map

Figure S2 - Van Krevelen Plot with regions highlighted and labelled. Van Krevelen is based on sample S14-2373. Broad regions are highlighted to aid in interpretation, though these are not absolute or definitive.

# Heteroatomic Class Distributions


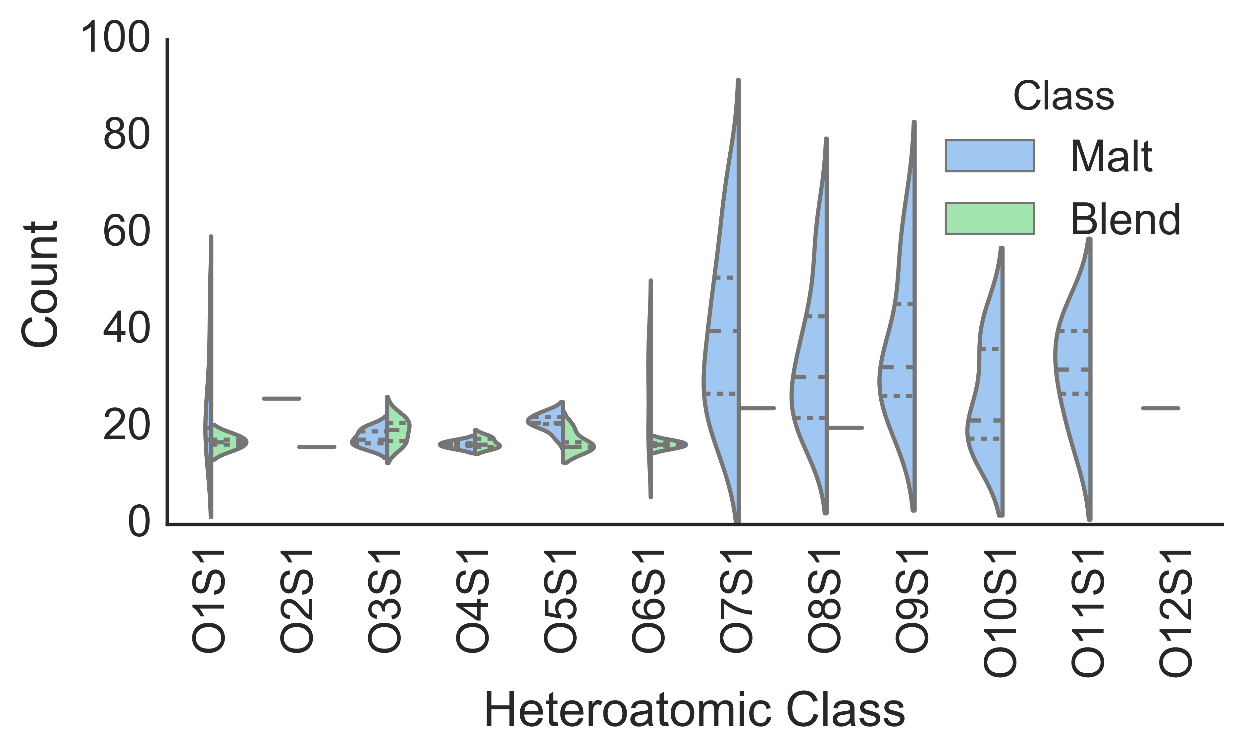


Figure S3 - OS class species violin plot, coloured according to class of whisky (Malt or Blend). Inner quartiles are shown with dotted lines. Note that the Malt class has more samples with sulfur containing species, especially at the higher oxygen number.


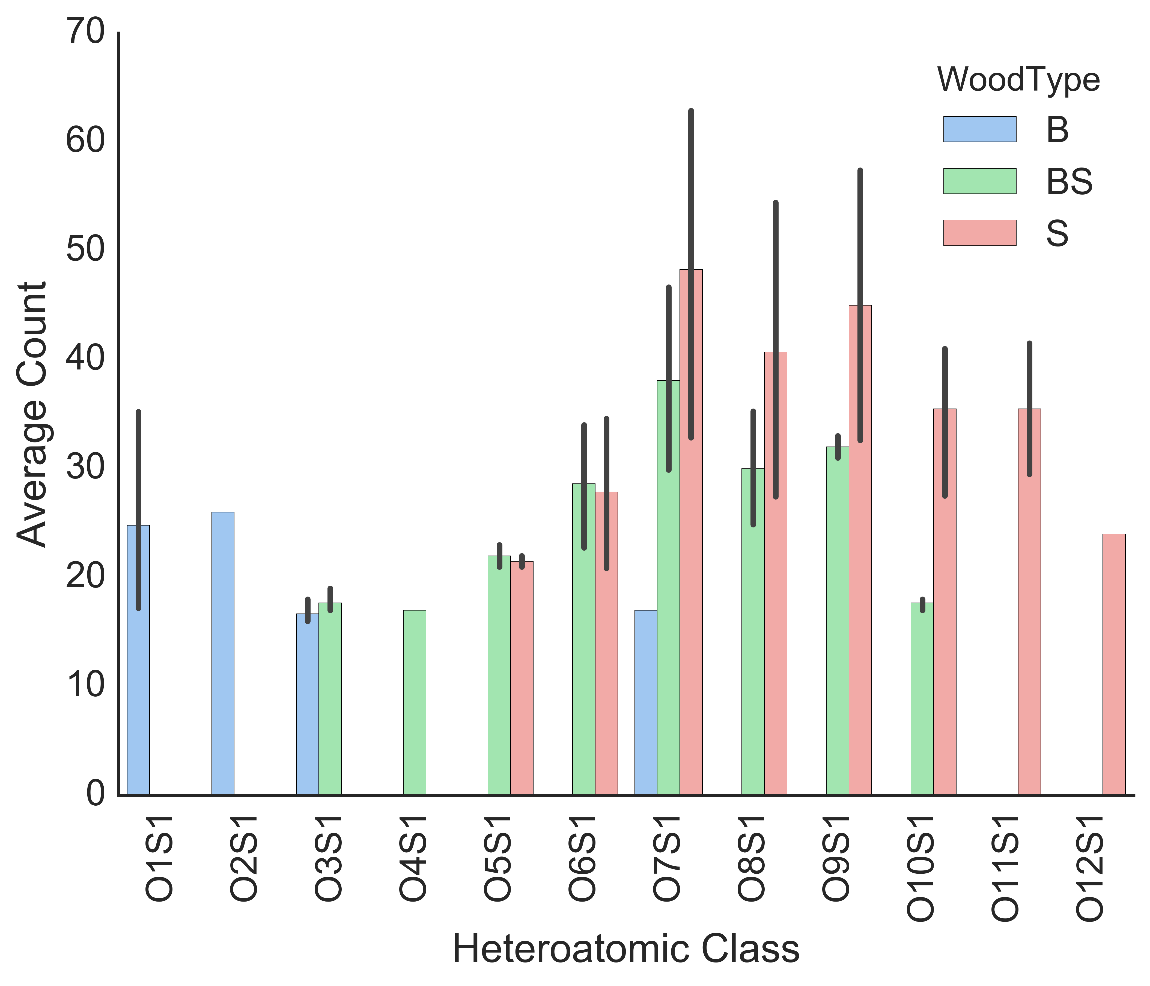


Figure S4 - OS class species average count bar plot by wood type, B = Bourbon only, S = Sherry only, BS = Bourbon and Sherry. Black lines indicated 95% confidence interval. Note that sherry cask matured whisky has higher OS classes present and a higher average count of their occurrence.

# Reproducibility in Scotch Whisky Production, Year on Year


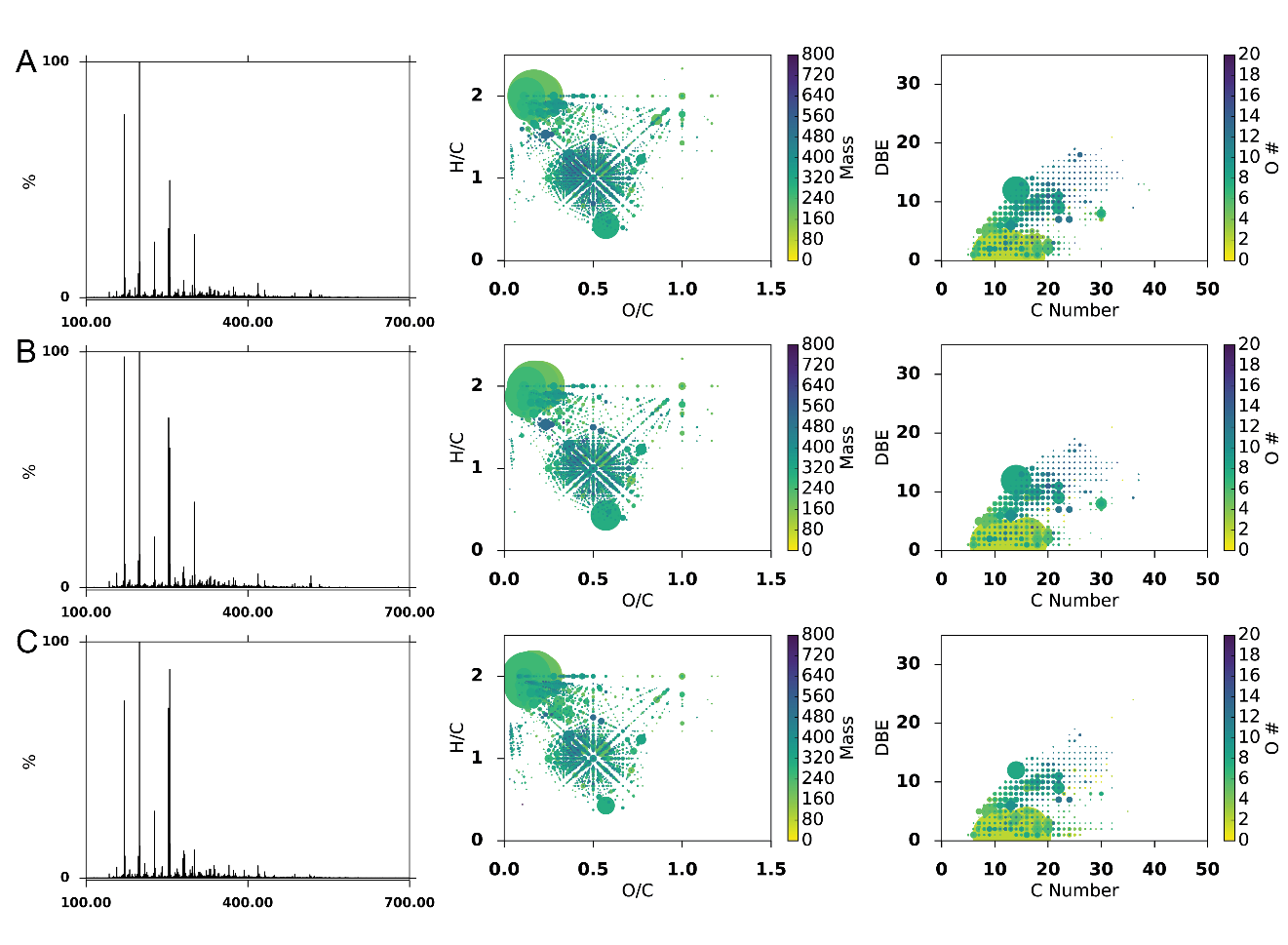


Figure S5 - Samples of the same Scotch Whisky product from three years, (A) S10-1849, (B) S12-1485, (C) S14-1947, a 10-year old ex-Bourbon cask matured peated Islay single malt. The left column shows the broadband mass spectra for each sample, the middle column shows a van Krevelen diagram coloured according to mass and sized according to normalised relative abundance, the right column shows a plot of carbon number versus double bond equivalent coloured according to oxygen number and sized according to normalised relative abundance.


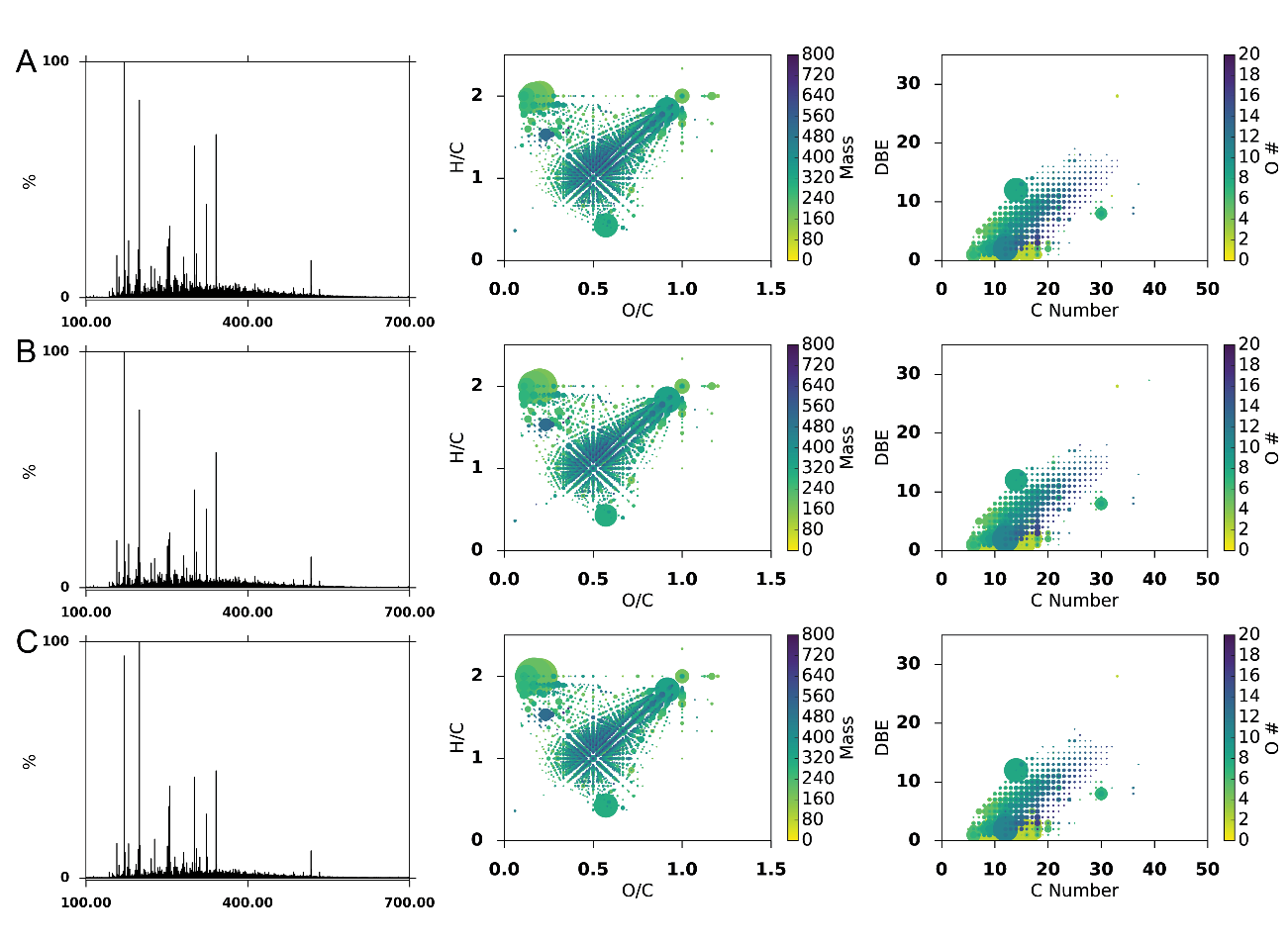


Figure S6 - Samples of the same Scotch Whisky product from three years, (A) S10-1133, (B) S12-1292, (C) S14-1944, a Blended Scotch Whisky. The left column shows the broadband mass spectra for each sample, the middle column shows a van Krevelen diagram coloured according to mass and sized according to normalised relative abundance, the right column shows a plot of carbon number versus double bond equivalent coloured according to oxygen number and sized according to normalised relative abundance.

# Multivariate Analysis

## PCA Loadings Plot


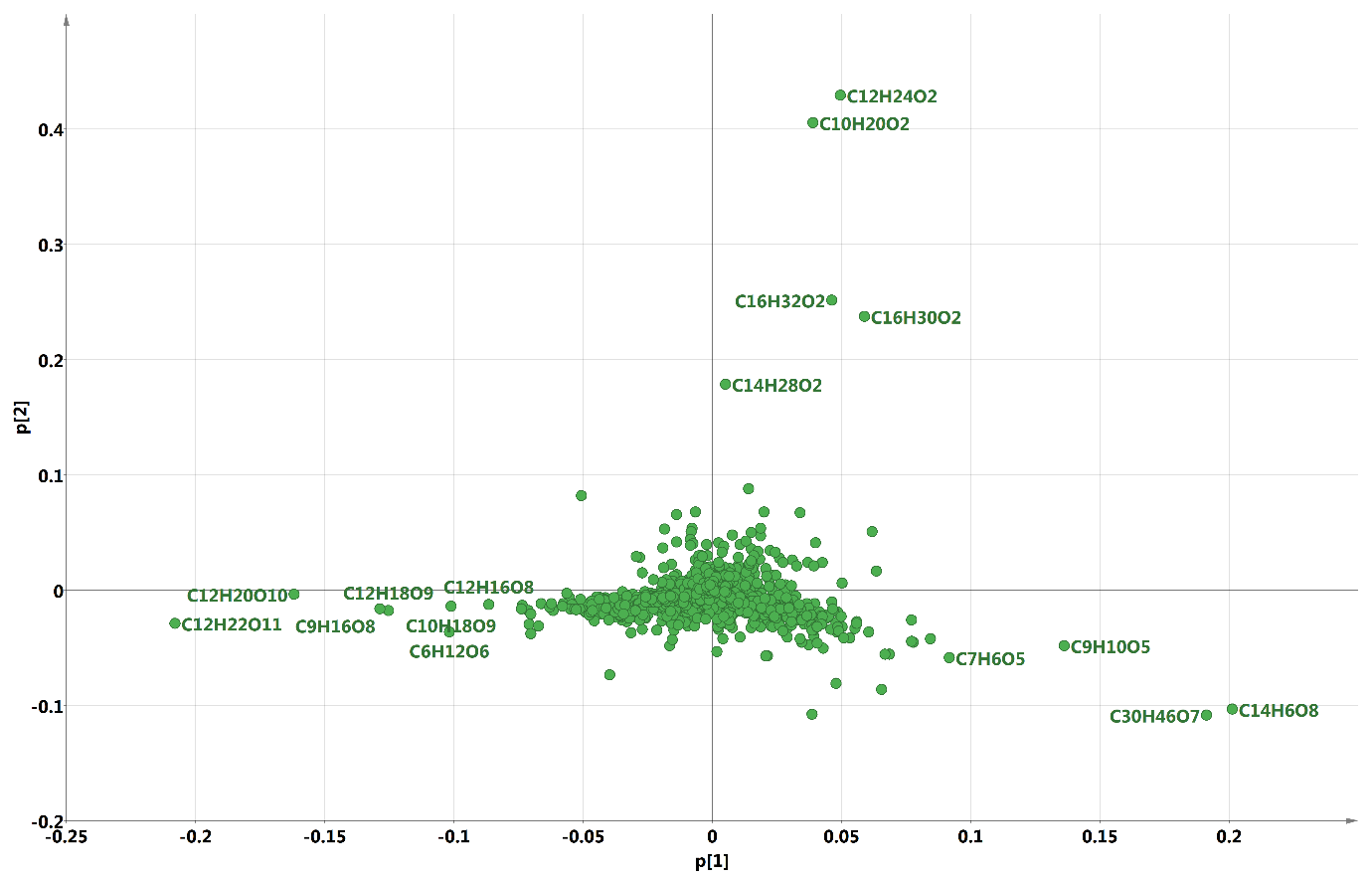


Figure S7 – Loadings plot for PCA model built using 9 principal components and unit variance scaling. Only the first two principal component loadings are shown. Key formulae are labelled. The scores plot (Fig. 5A) places the malts to the right and blends to the left of PC[1], as such the p[1] axis can be interpreted to highlight which formulae are related to these classifications.

## OPLS-DA Model for Malt vs Blend Classification


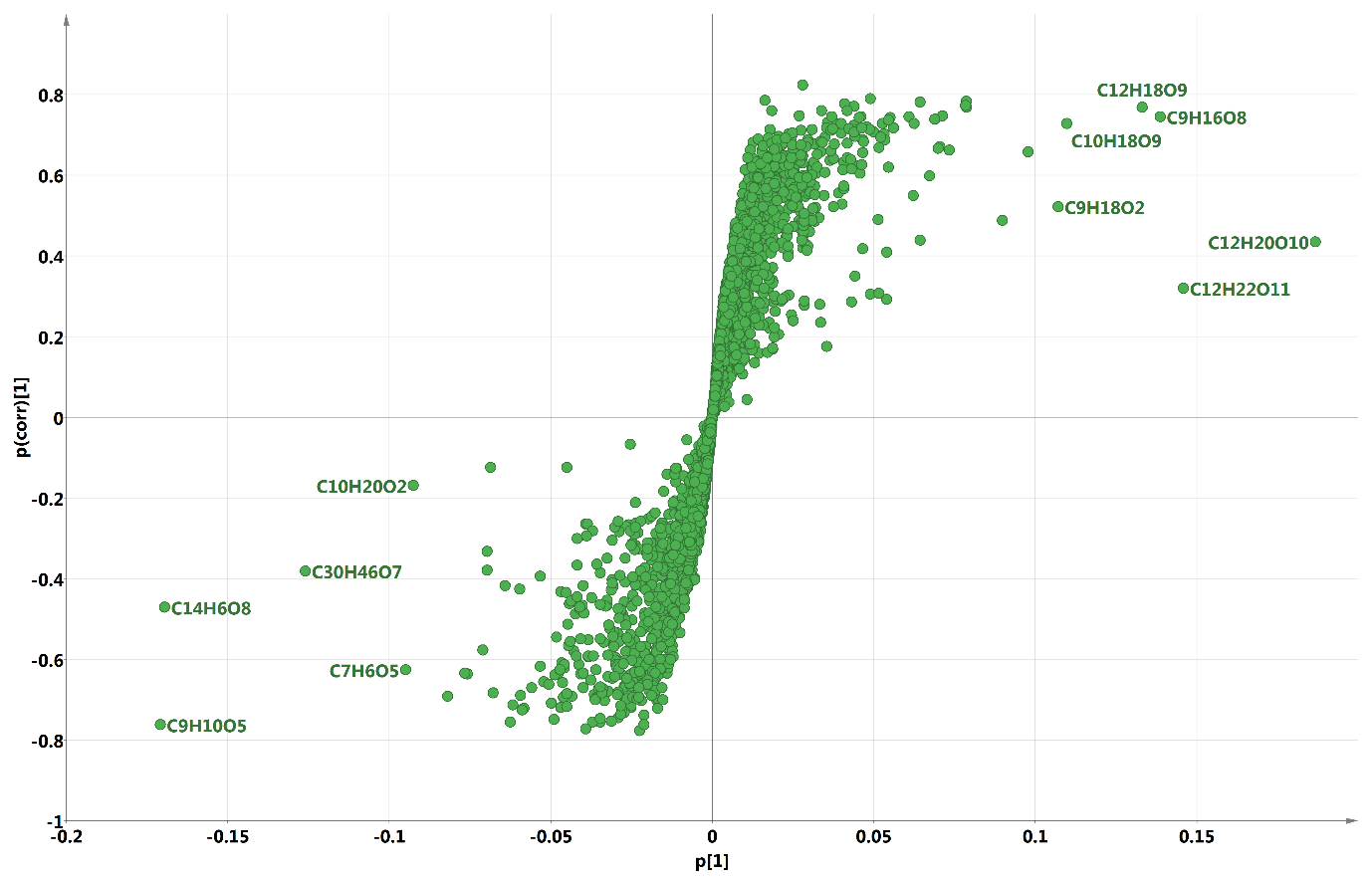


Figure S8 - OPLS-DA S-Plot for Malt versus Blend model (n=85 samples). Model constructed with 1 predicted and 4 orthogonal components. Shown are p[1] (loadings for predictive component) and p(corr)[1] (loadings scaled to correlation of variables and classes).

## OPLS-DA Model for Regional Classification


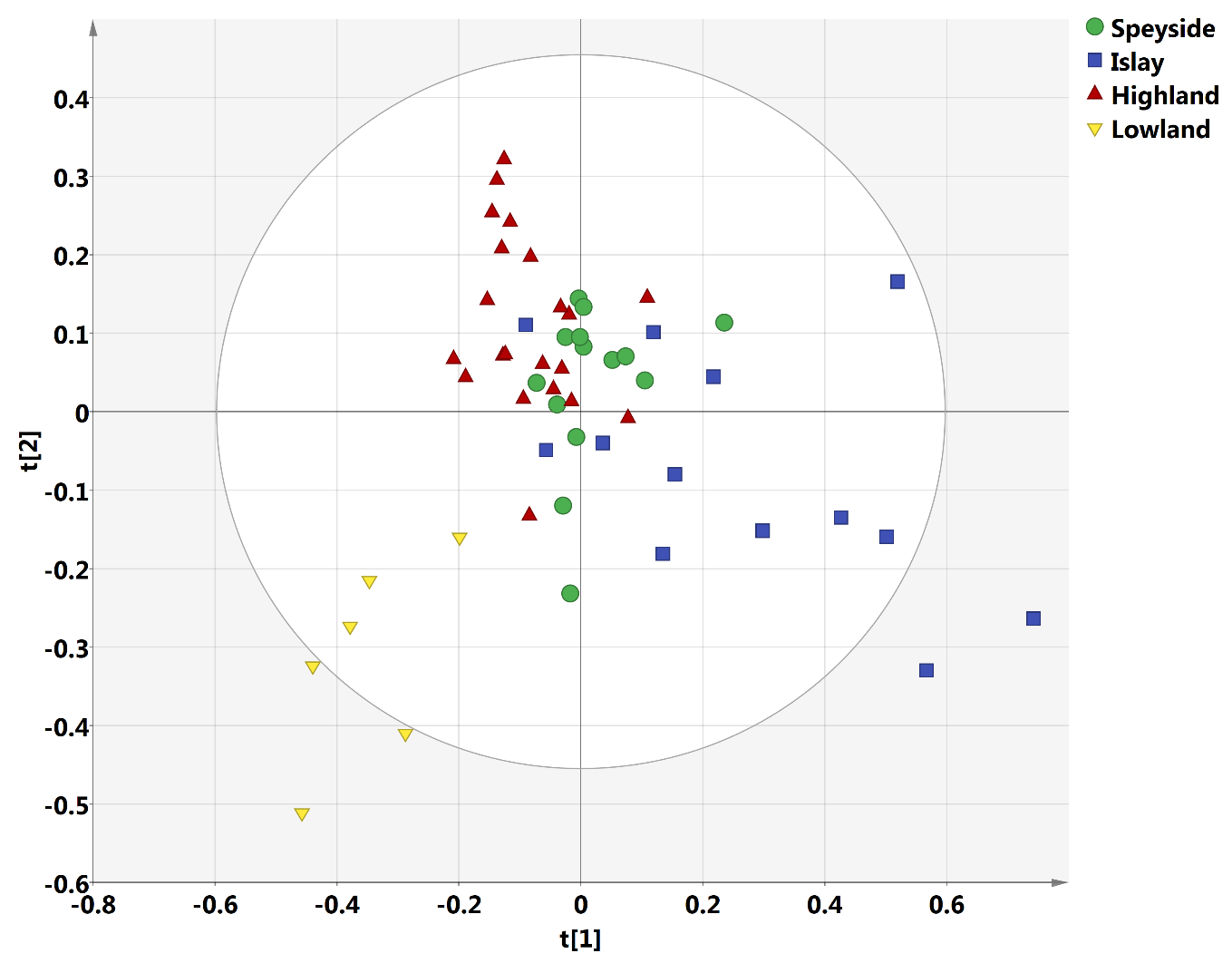


Figure S91 - OPLS-DA Scores plot for regions of Scotch Whisky production, n=54. Model constructed with 3 predictive components and 2 orthogonal components. Scores plot shows first and second predictive components. R^2^X=0.65, Q^2^=0.199

## OPLS-DA Model for Peated vs Unpeated Classification


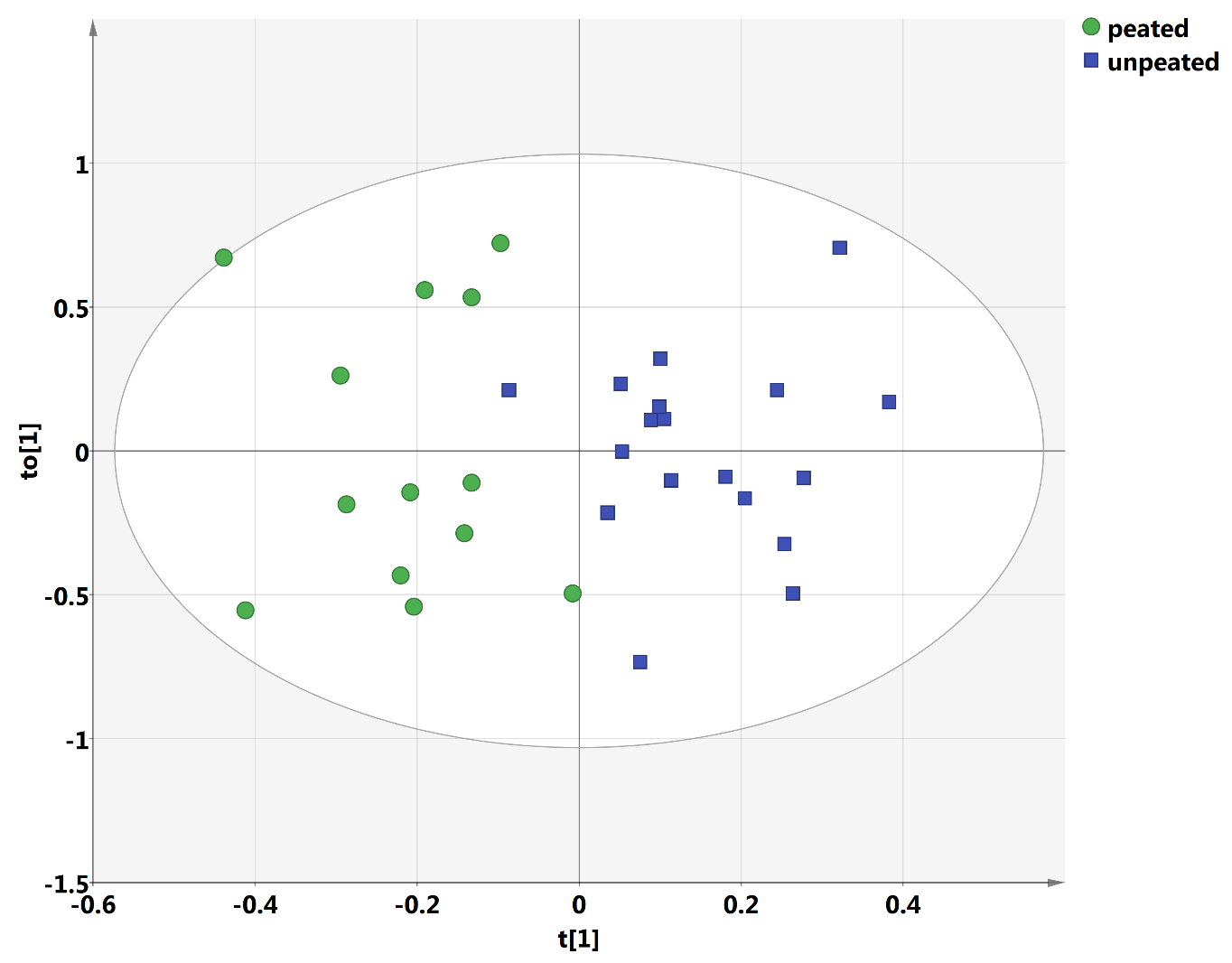


Figure S10 - OPLS-DA Scores plot for peated versus non-peated Scotch Whisky, n=36. Model constructed with 1 predictive component and 2 orthogonal components. R^2^X=0.594, Q^2^=0.094. Scores plot shows predictive component versus first orthogonal component.

## OPLS-DA Model for Age Statements of Scotch Whisky


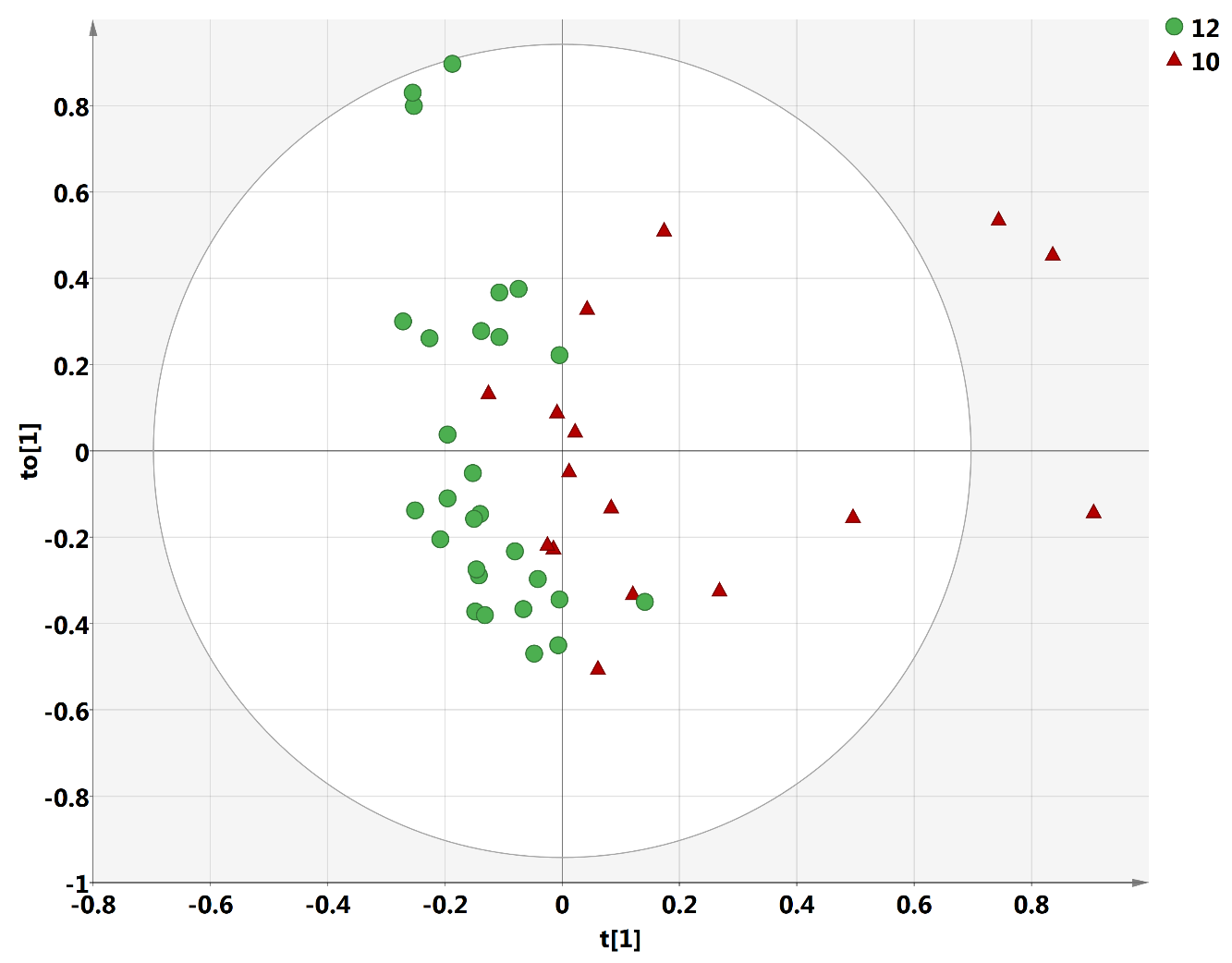


Figure S21 - OPLS-DA Scores plot for ages 10 and 12 years, n=44. Model constructed with 1 predictive and 1 orthogonal component. R^2^X=0.381, Q^2^=-0.099.

## OPLS-DA Model for Wood Types During Maturation


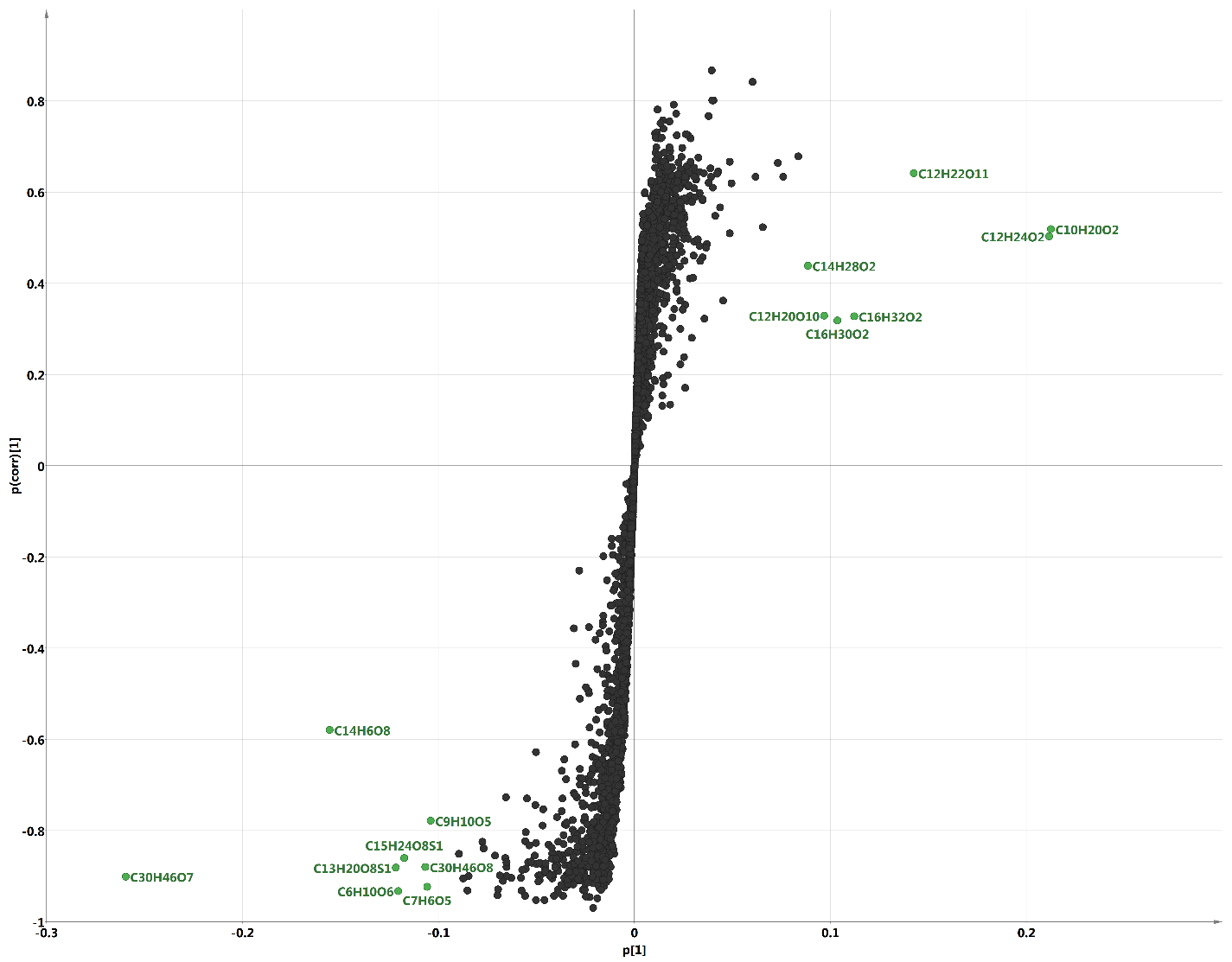


*Figure S12 - OPLS-DA S-Plot for model built for malts matured in ex-Bourbon or ex-Sherry casks only. Major discriminating variables are highlighted and labelled. Axes show p[1] (loadings) and p(corr)[1] (loadings scaled to correlation of variables and class).*

# Fragmentation Study of Species C_30_H_46_O_7_


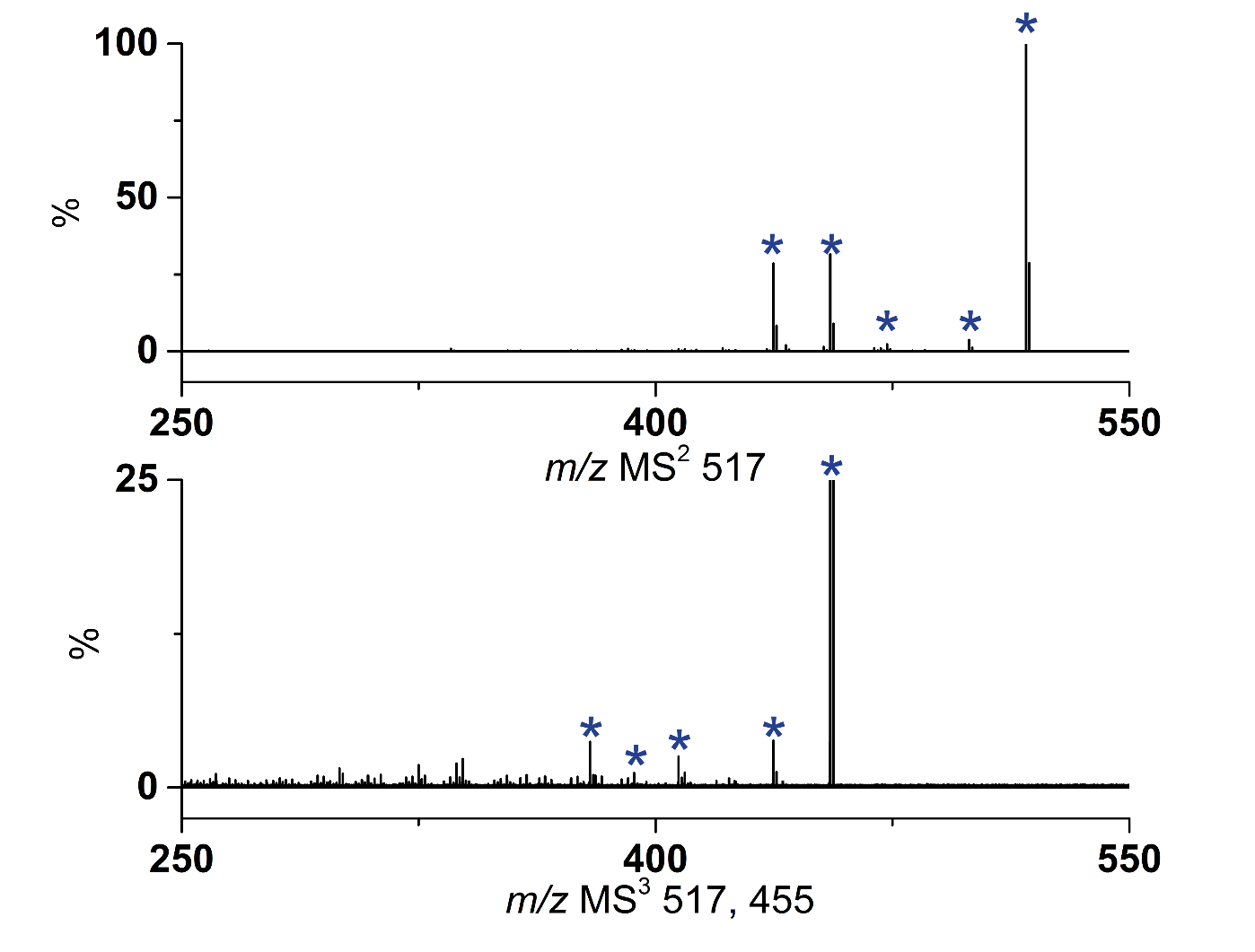


Figure S13 – S14-1941 Fragmentation spectra; (top) MS^2^ 517 and (bottom) MS^3^ 517-455. Key peaks which are listed in Table 5 are highlighted here.

Table 5 from the main text is reproduced here for reference.

Fragmentation peaks observed for molecular ion C_30_H_45_O_7_. Note that mass spectra were not calibrated post-acquisition, which accounts for the larger errors.

| MS^n^ | m/z | Ion Formula [M-H]^-^ | Fragmentation | Error (ppm) |
| --- | --- | --- | --- | --- |
| MS | 517.31708 | C_30_H_45_O_7_ | M-H | 1.43 |
| MS^2^ (517) | 499.30591 | C_30_H_43_O_6_ | M-H-H_2_O | 1.21 |
|  | 473.32671 | C_29_H_45_O_5_ | M-H-CO_2_ | 1.14 |
|  | 455.31610 | C_29_H_43_O_4_ | M-H-CO_2_-H_2_O | 1.28 |
|  | 437.30559 | C_29_H_41_O_3_ | M-H-CO_2_-H_2_O-H2O | 1.21 |
| MS^3^ (517, 455) | 455.31608 | C_29_H_41_O_3_ | M-H-CO_2_-H_2_O | 1.32 |
|  | 437.30612 | C_29_H_41_O_3_ | M-H-CO_2_-H_2_O-H_2_O | 1.25 |
|  | 407.29504 | C_28_H_39_O_2_ | M-H-CO_2_-H_2_O-H_2_O-H_2_O | 1.27 |
|  | 393.27939 | C_27_H_37_O_2_ | M-H-CO_2_-H_2_O-H_2_O-H_2_O-CH_2_ | 1.31 |
|  | 379.30014 | C_27_H_39_O | M-H-CO_2_-H_2_O-H_2_O-H_2_O-CH_2_-H_2_O | 1.32 |
