# Supplementary material for: Chemical Diversity and Complexity of Scotch Whisky as Revealed by High-Resolution Mass Spectrometry
Source: J Am Soc Mass Spectrom. 2016 Oct 17;28(1):200–13. doi: 10.1007/s13361-016-1513-y (PMC5174148; doi:10.1007/s13361-016-1513-y)
Supplement: Supplementary file 2 — (PDF 13513 kb) [file 13361_2016_1513_MOESM2_ESM.pdf]

Chemical Diversity and Complexity of Scotch Whisky as Revealed by High-Resolution Mass Spectrometry

W. Kew, I. Goodall, D. Clarke and D. Uhrín

Journal of the American Society for Mass Spectrometry

Corresponding Authors:

- Dr David Clarke, EaStCHEM, School of Chemistry, Joseph Black Building, University of Edinburgh, Edinburgh, UK, EH9 3FJ, Tel: +44(0)131 650 4808, email: david.clarke@ed.ac.uk
- Dr Dušan Uhrín, EaStCHEM, School of Chemistry, Joseph Black Building, University of Edinburgh, Edinburgh, UK, EH9 3FJ, Tel: +44(0)131 650 4742, email: dusan.uhrin@ed.ac.uk

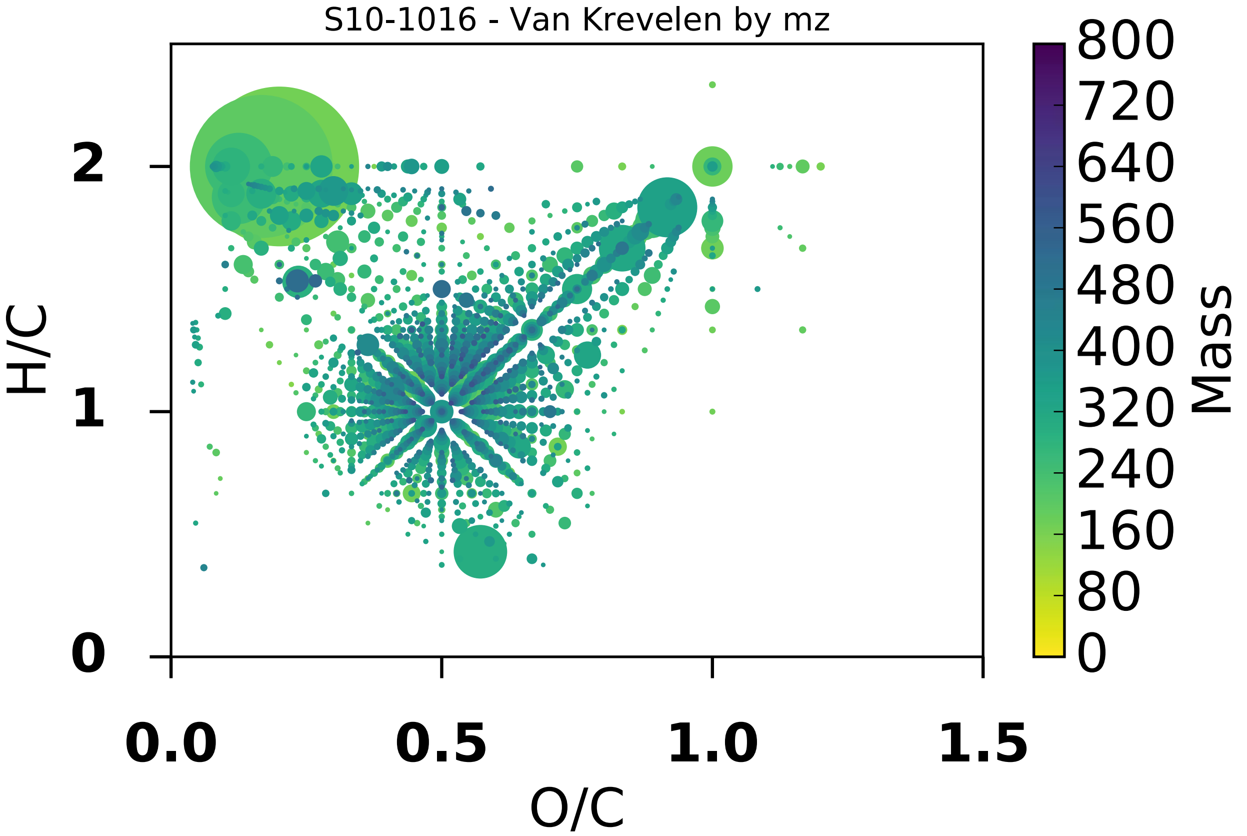

S10-1016 - Van Krevelen by mz
H/C
2
1
0
0.0
0.5
1.0
1.5
O/C
800
720
640
560
480
400
320
240
160
80
0
Mass

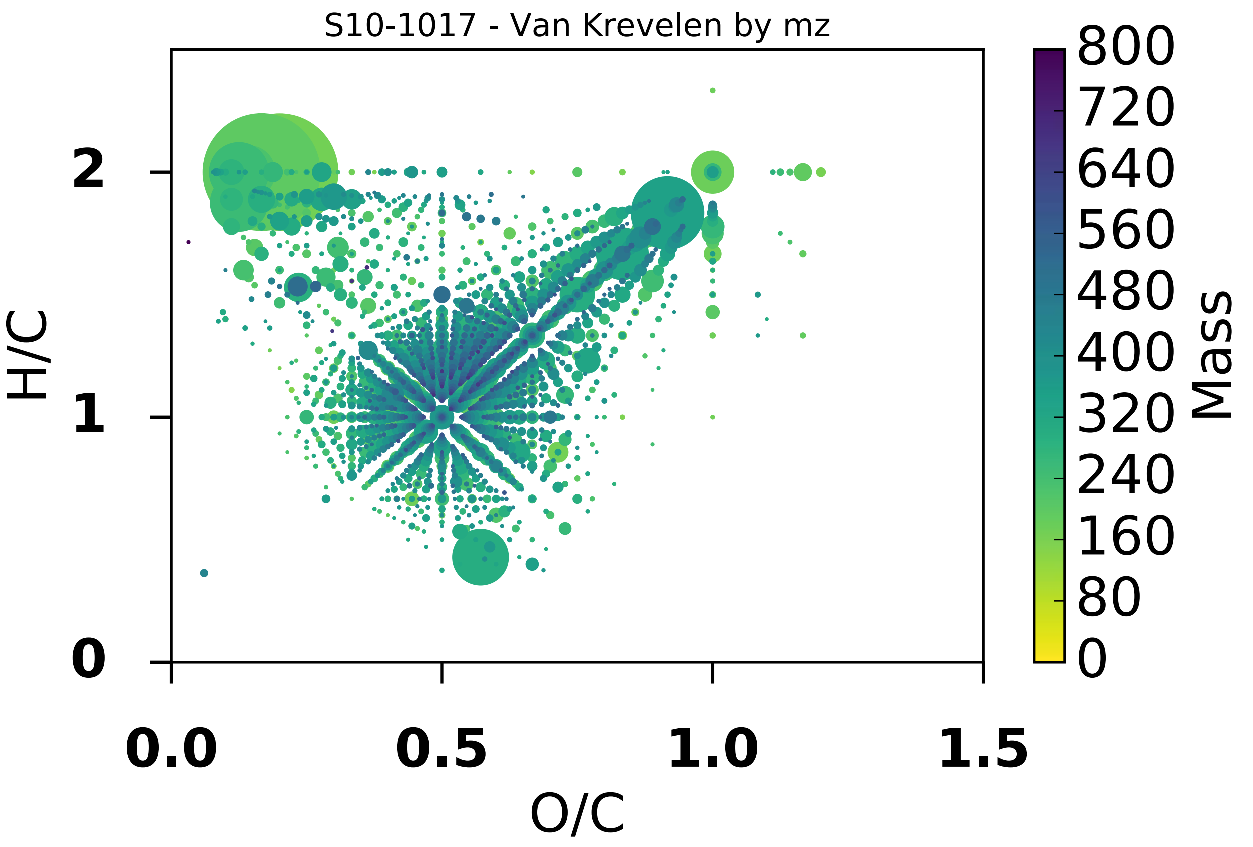

S10-1017 - Van Krevelen by mz
H/C
O/C
0.0
0.5
1.0
1.5
0
1
2
Mass
0
80
160
240
320
400
480
560
640
720
800

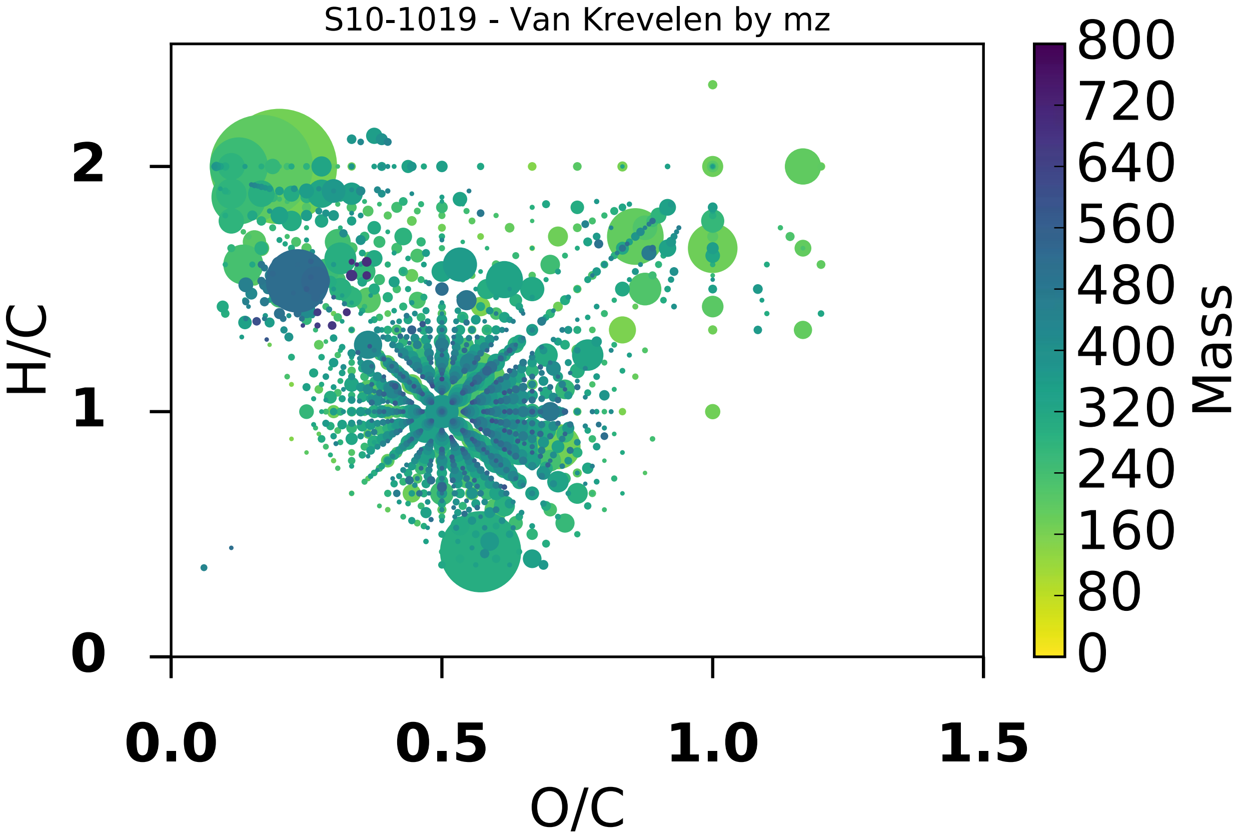

S10-1019 - Van Krevelen by mz
H/C
0
1
2
0.0
0.5
1.0
1.5
O/C
Mass
0
80
160
240
320
400
480
560
640
720
800

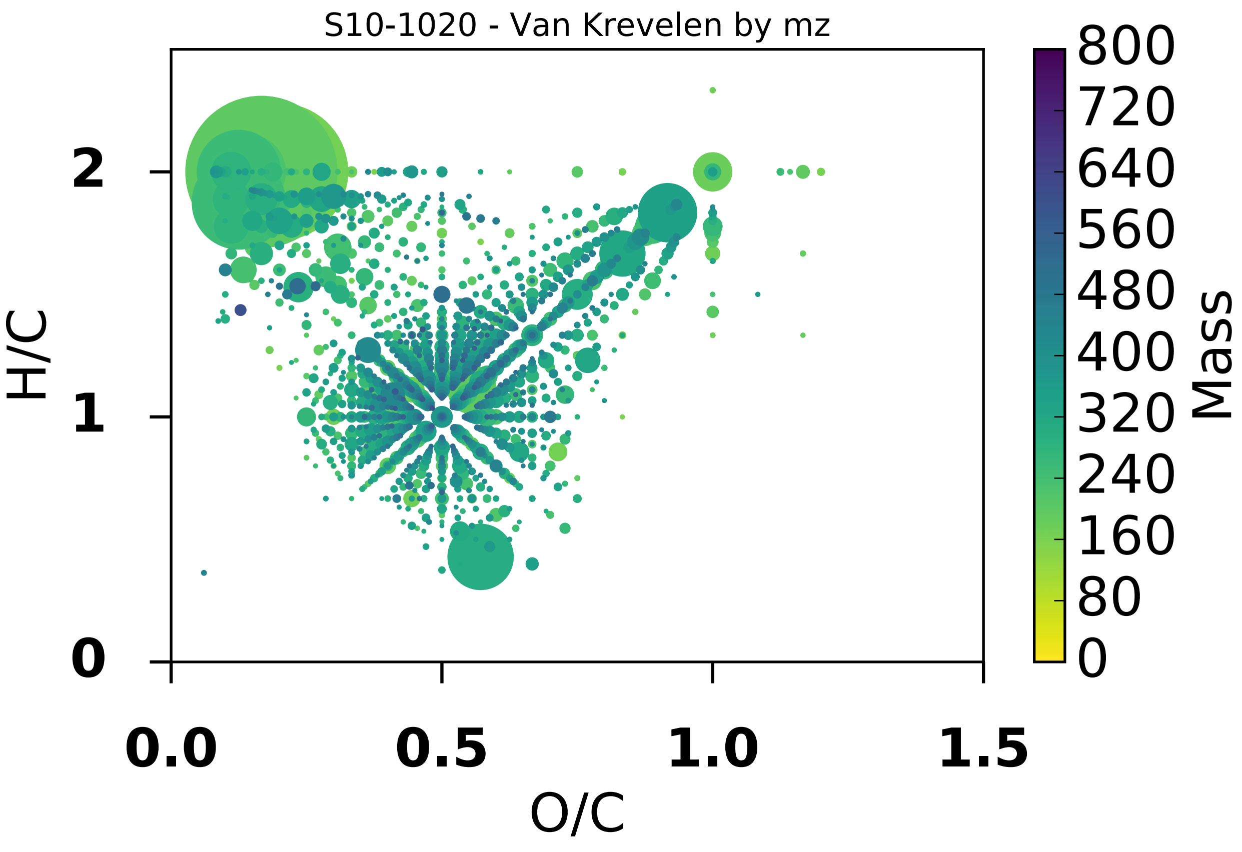

S10-1020 - Van Krevelen by mz
H/C
O/C
Mass
0
1
2
0.0
0.5
1.0
1.5
0
80
160
240
320
400
480
560
640
720
800

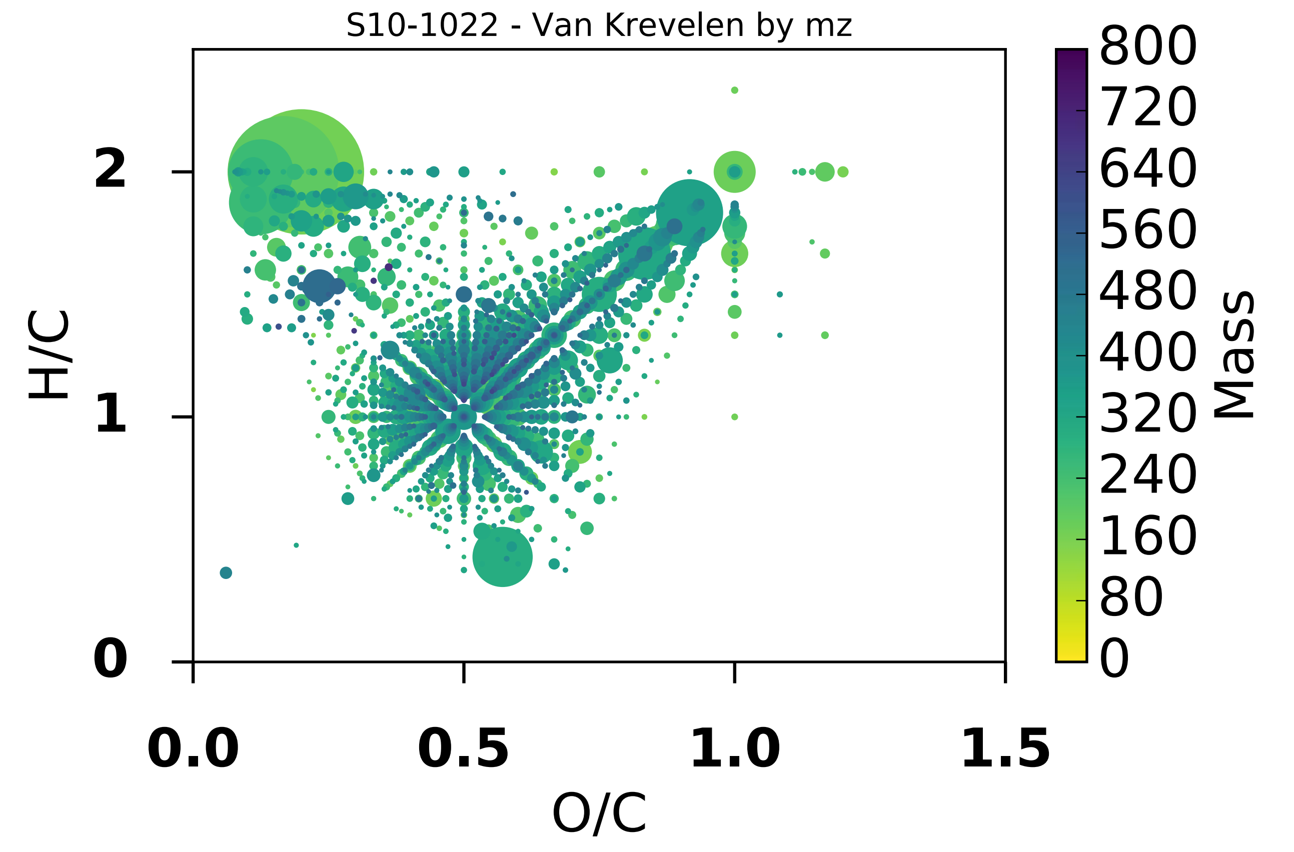
S10-1022 - Van Krevelen by mz
H/C
2
1
0
O/C
0.0
0.5
1.0
1.5
Mass
800
720
640
560
480
400
320
240
160
80
0

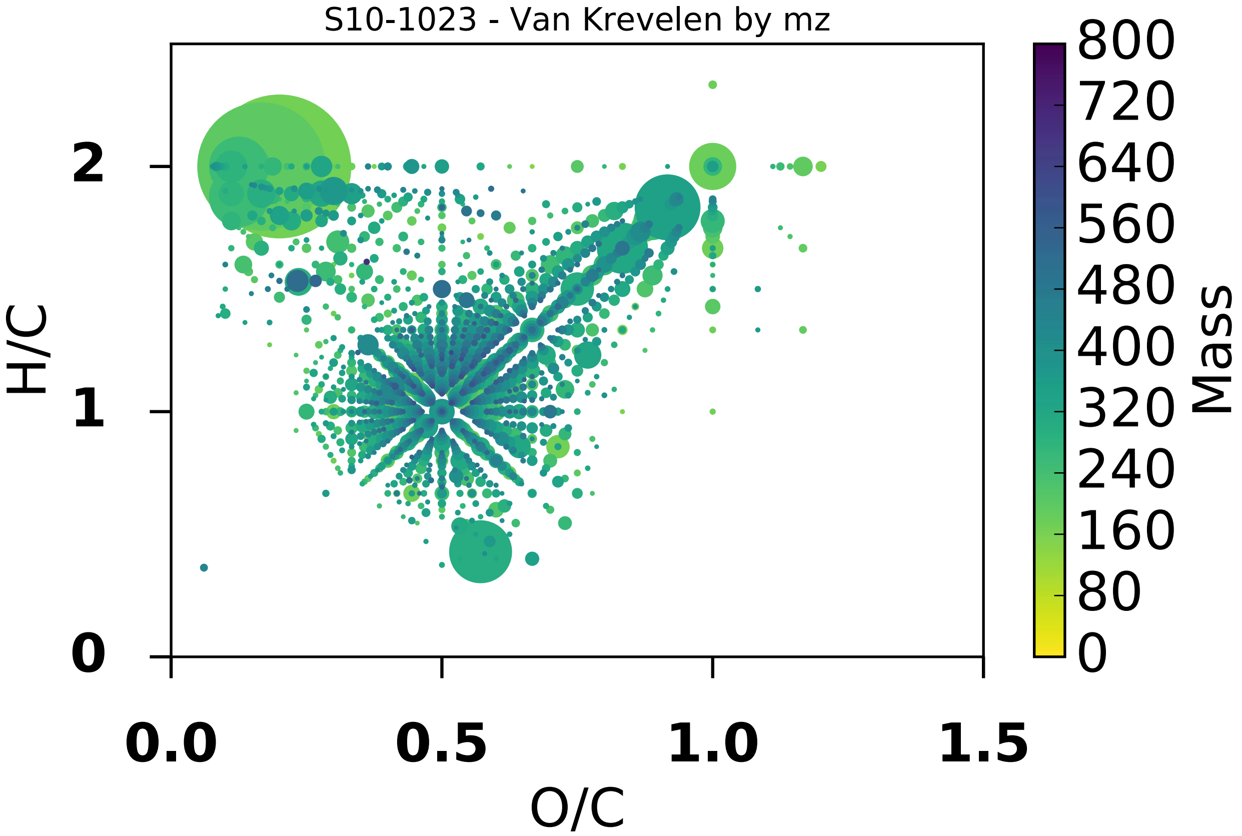

S10-1023 - Van Krevelen by mz
H/C
O/C
Mass
0
1
2
0.0
0.5
1.0
1.5
800
720
640
560
480
400
320
240
160
80
0

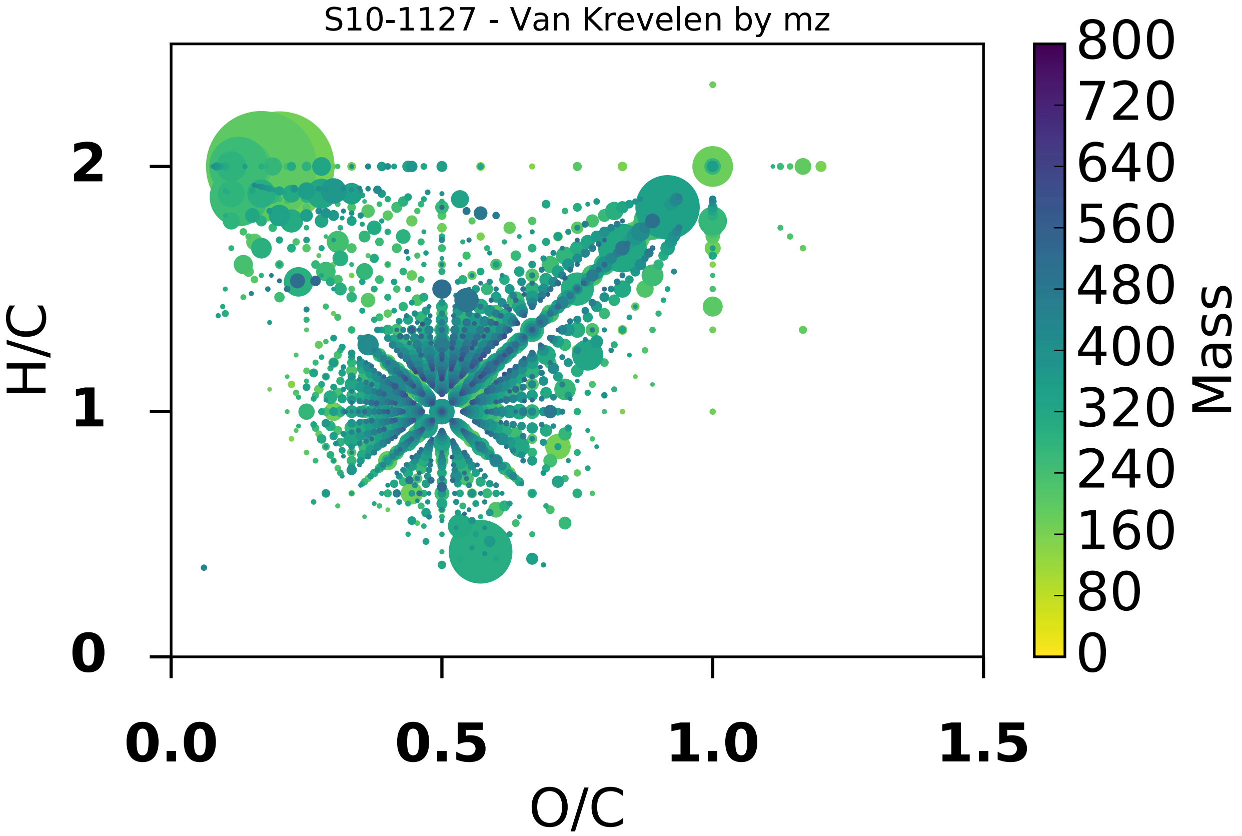
S10-1127 - Van Krevelen by mz
H/C
2
1
0
0.0
0.5
1.0
1.5
O/C
800
720
640
560
480
400
320
240
160
80
0
Mass

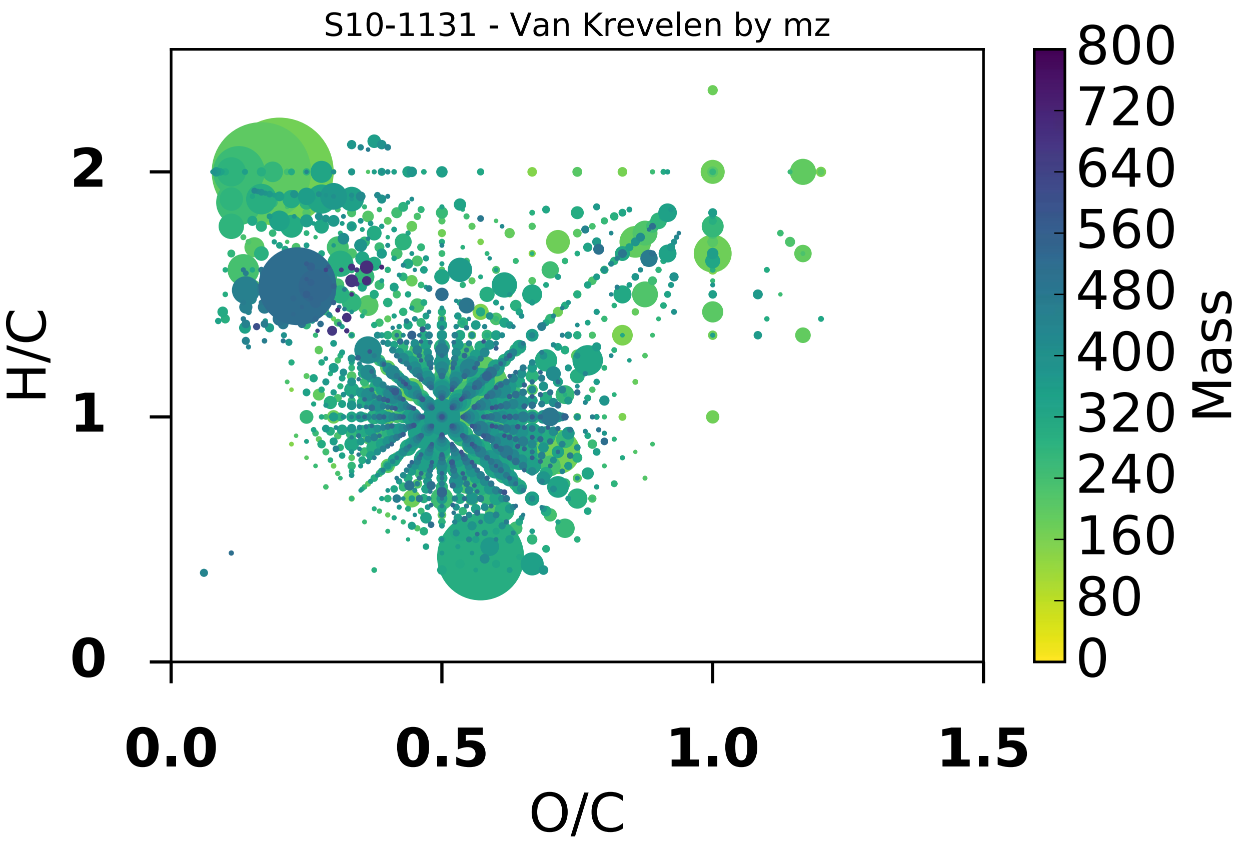
S10-1131 - Van Krevelen by mz
H/C
O/C
0.0
0.5
1.0
1.5
0
1
2
Mass
800
720
640
560
480
400
320
240
160
80
0

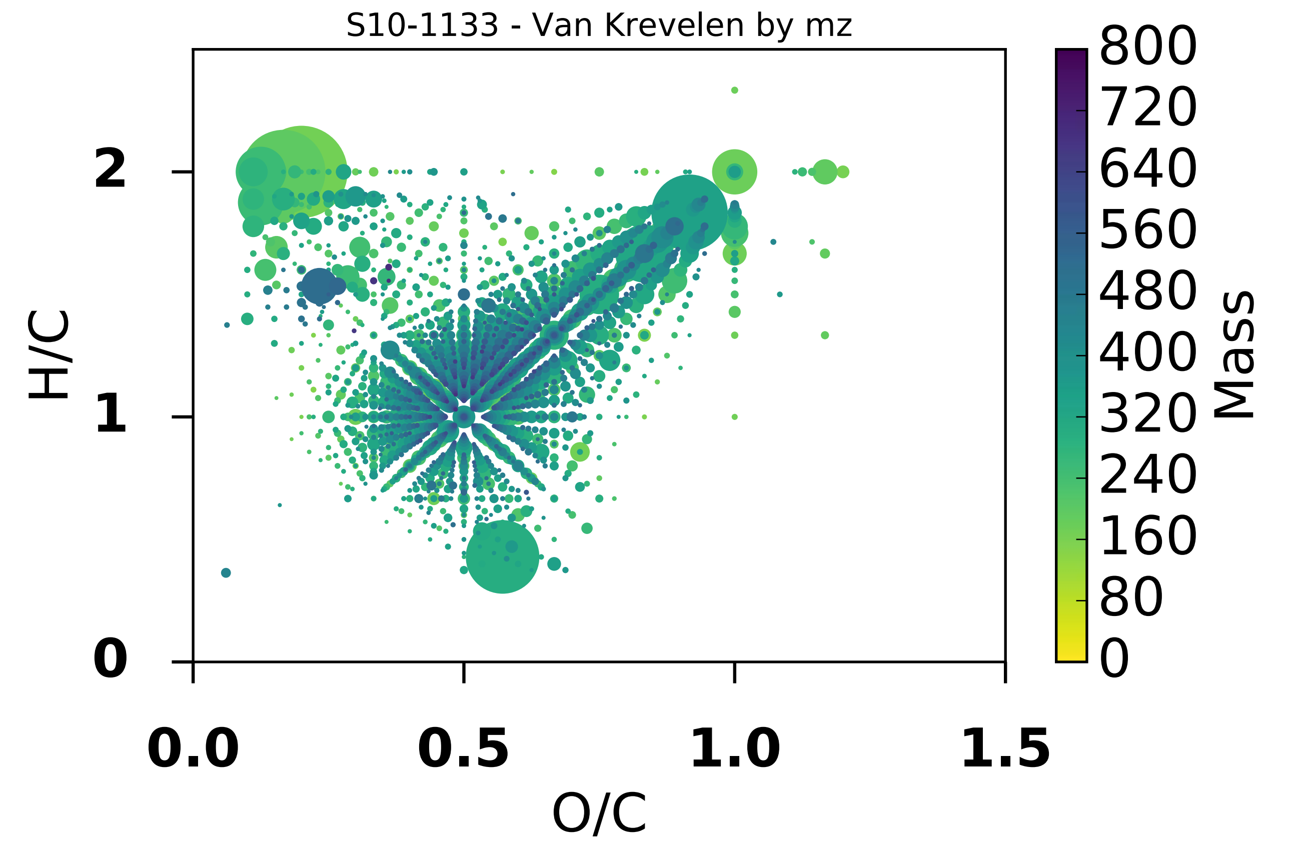

S10-1133 - Van Krevelen by mz
H/C
O/C
0.0
0.5
1.0
1.5
0
1
2
Mass
800
720
640
560
480
400
320
240
160
80
0

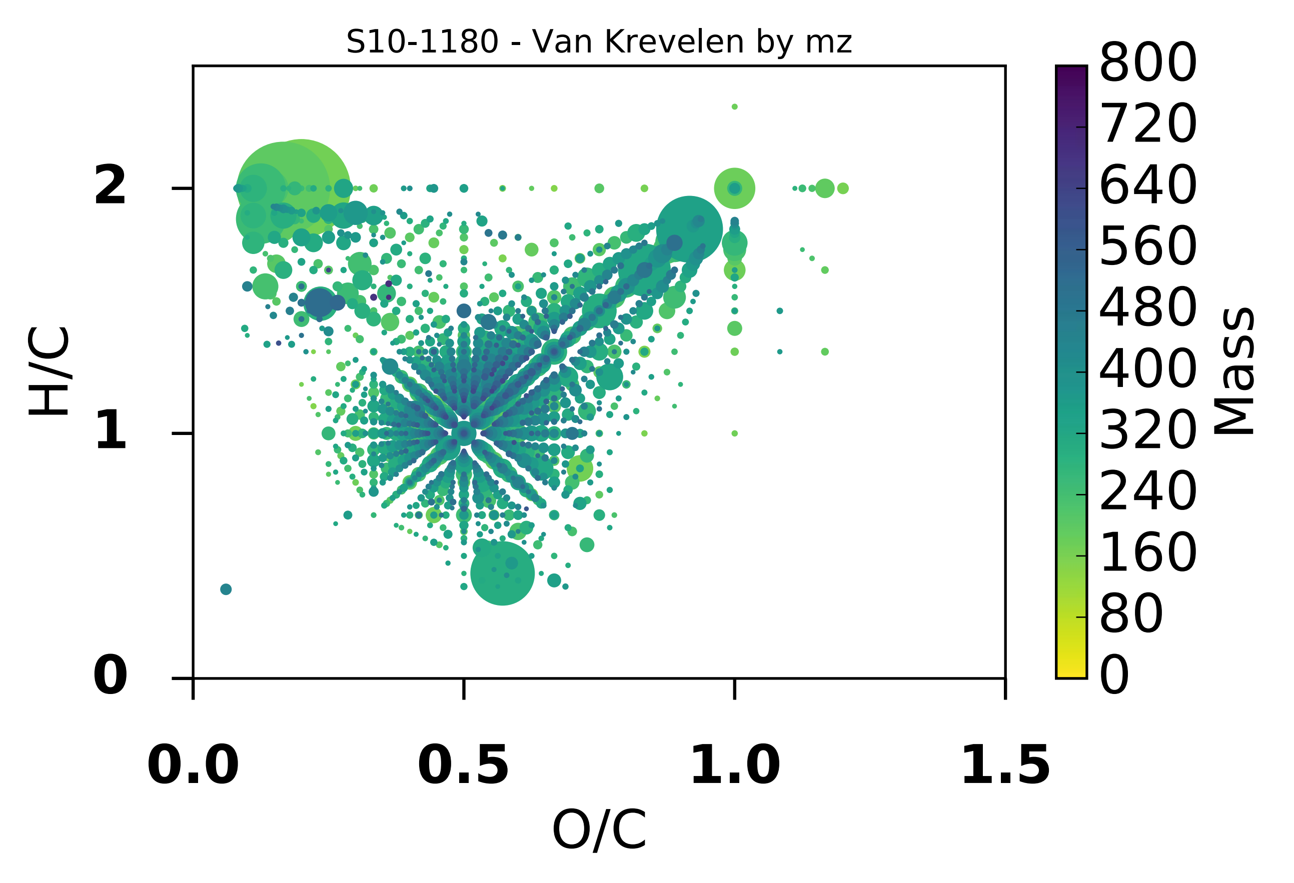

S10-1180 - Van Krevelen by mz
H/C
O/C
Mass
0
1
2
0.0
0.5
1.0
1.5
800
720
640
560
480
400
320
240
160
80
0

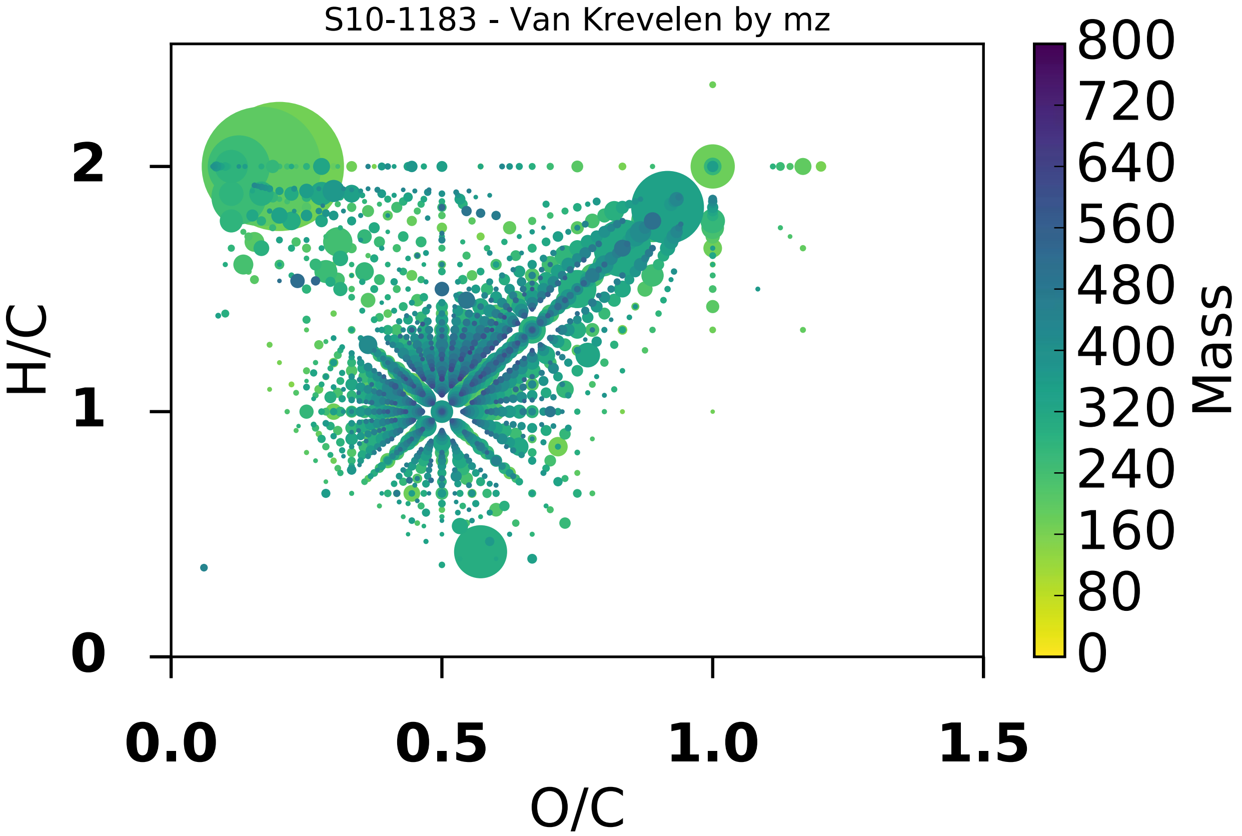

S10-1183 - Van Krevelen by mz
H/C
O/C
0
1
2
0.0
0.5
1.0
1.5
Mass
0
80
160
240
320
400
480
560
640
720
800

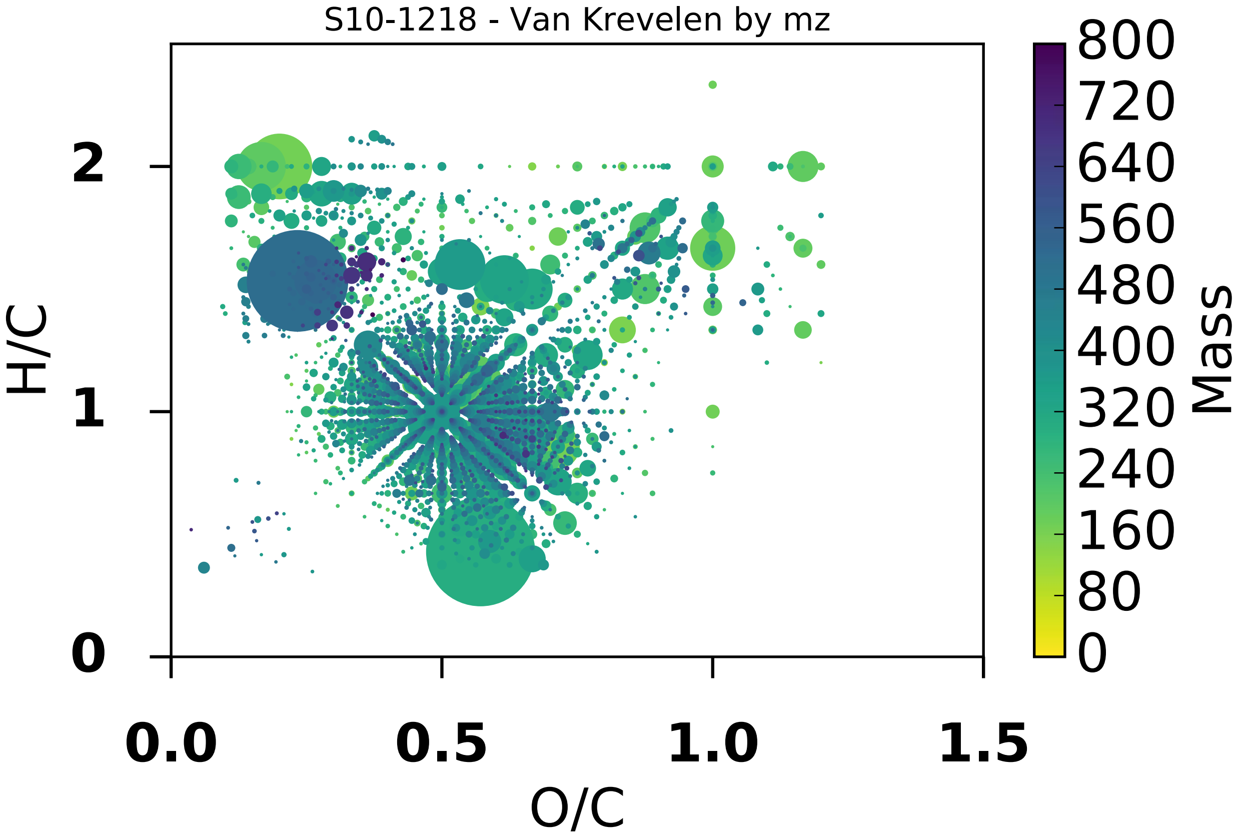
S10-1218 - Van Krevelen by mz
H/C
O/C
Mass
2
1
0
0.0
0.5
1.0
1.5
800
720
640
560
480
400
320
240
160
80
0

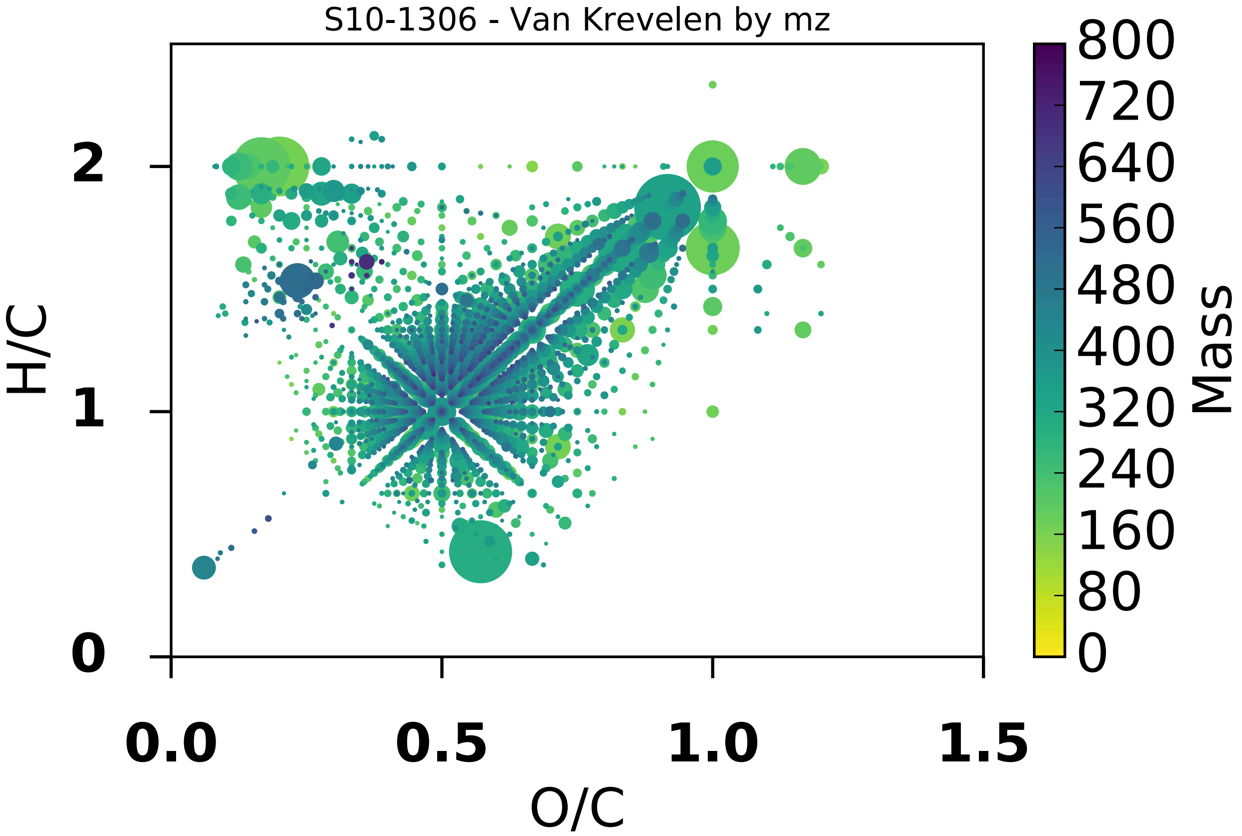
S10-1306 - Van Krevelen by mz
H/C
2
1
0
0.0
0.5
1.0
1.5
O/C
800
720
640
560
480
400
320
240
160
80
0
Mass

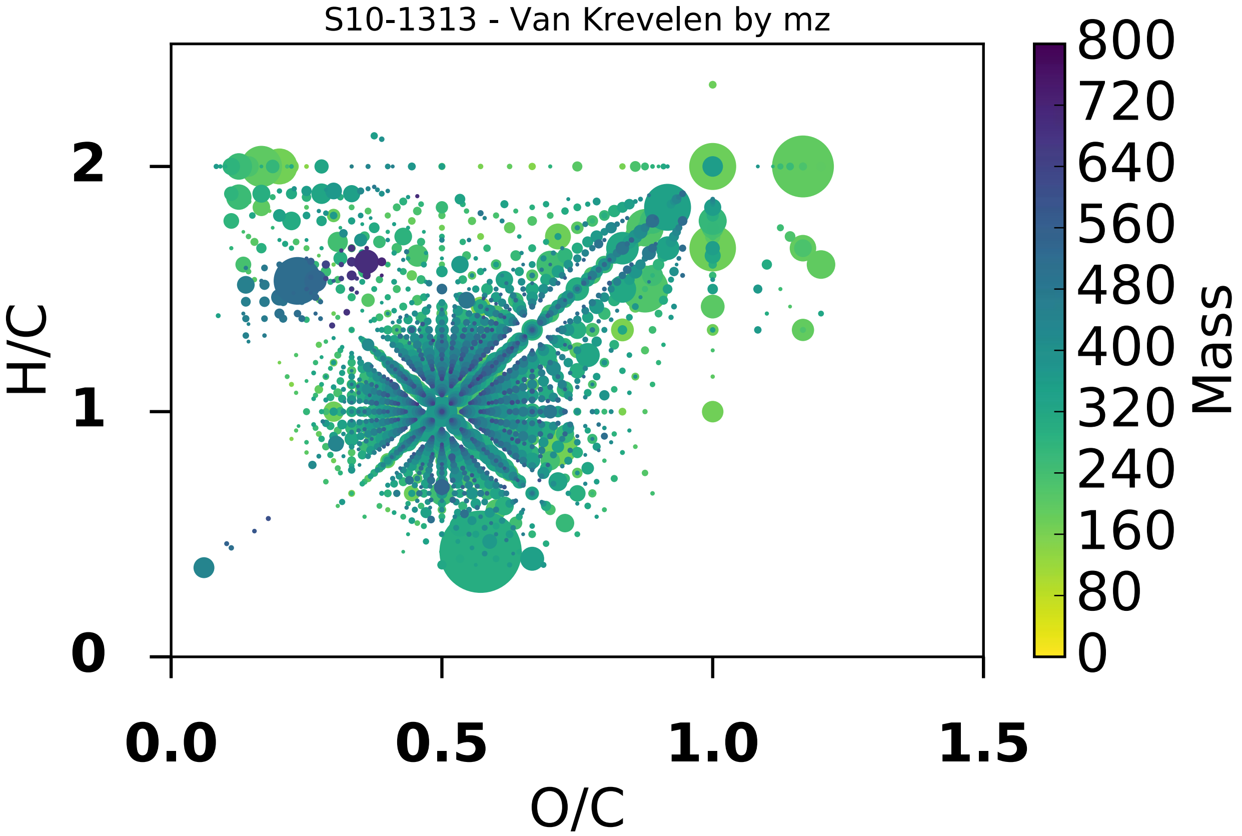
S10-1313 - Van Krevelen by mz
H/C
2
1
0
0.0
0.5
1.0
1.5
O/C
800
720
640
560
480
400
320
240
160
80
0
Mass

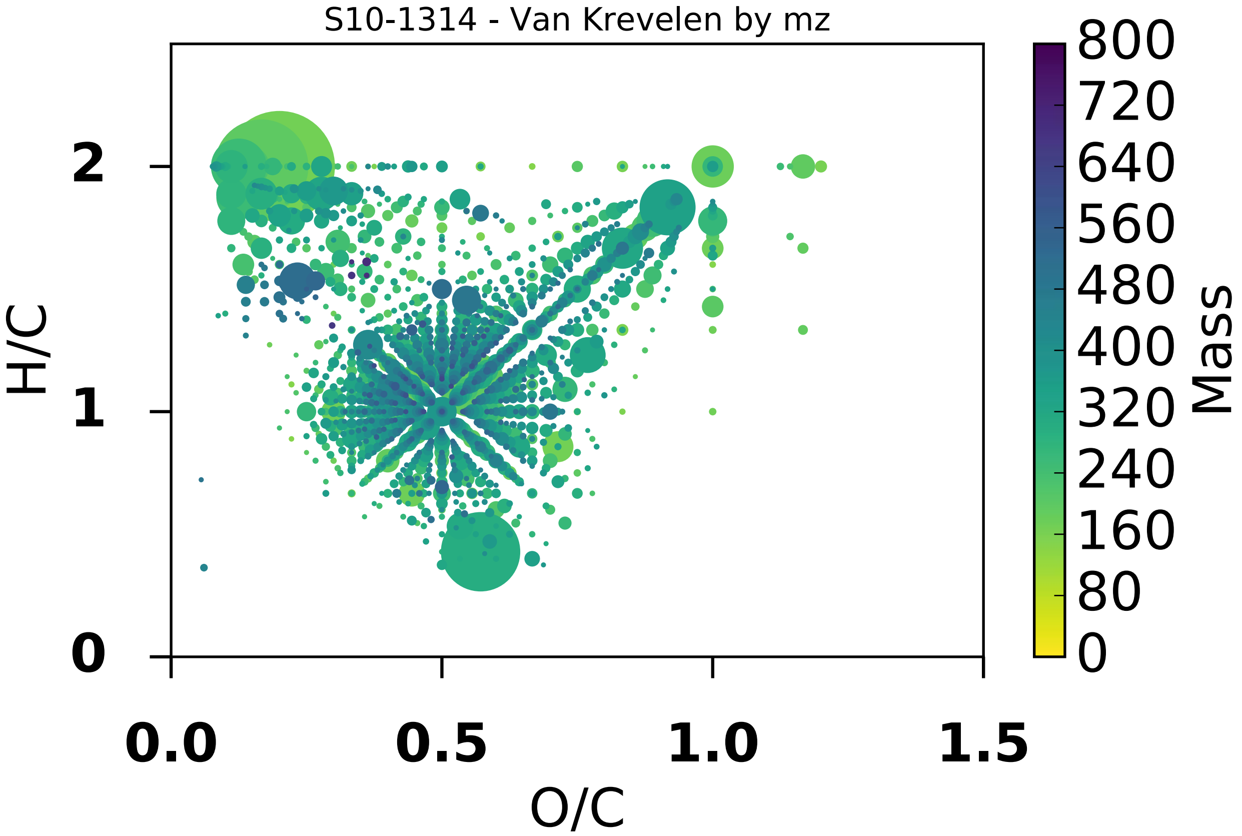

S10-1314 - Van Krevelen by mz
H/C
2
1
0
0.0
0.5
1.0
1.5
O/C
800
720
640
560
480
400
320
240
160
80
0
Mass

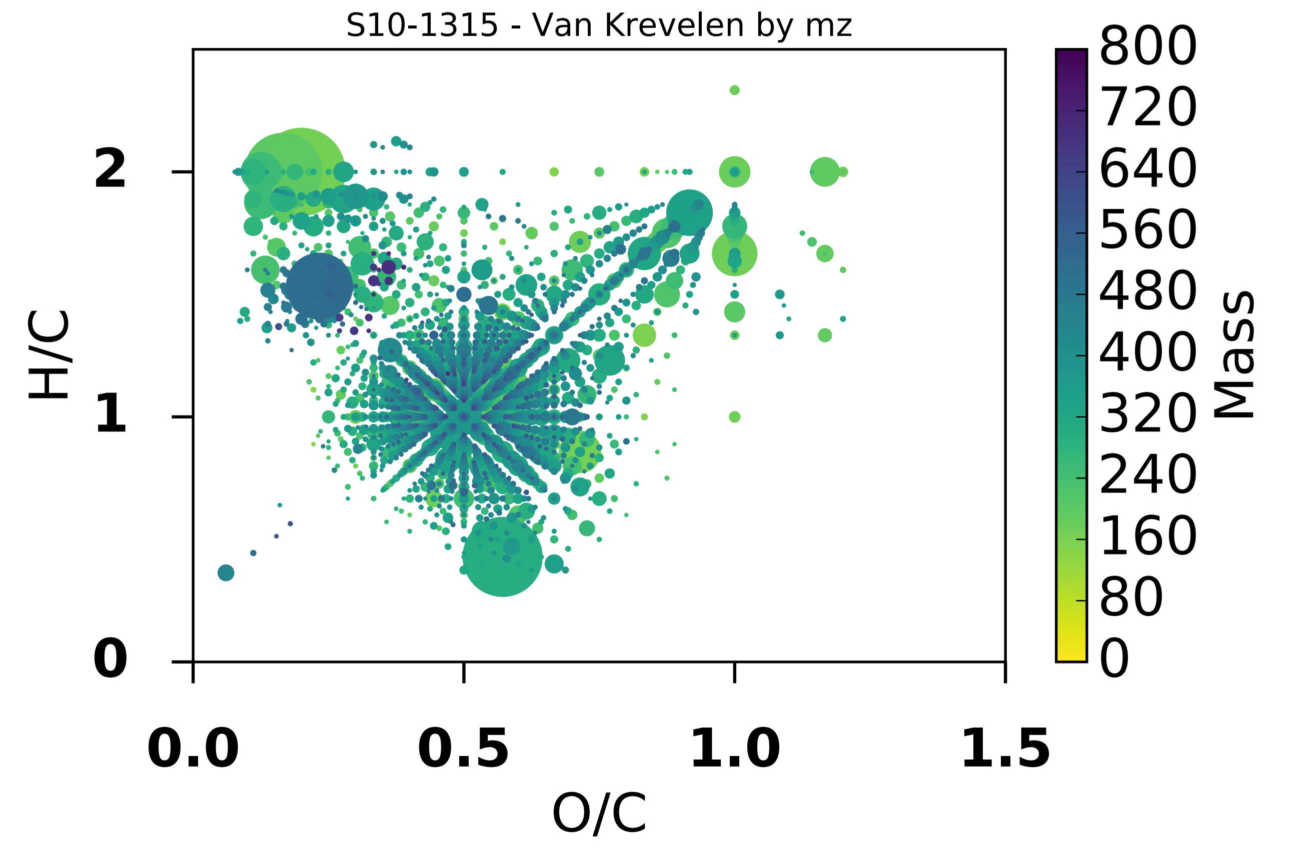

S10-1315 - Van Krevelen by mz
H/C
O/C
0
1
2
0.0
0.5
1.0
1.5
Mass
0
80
160
240
320
400
480
560
640
720
800

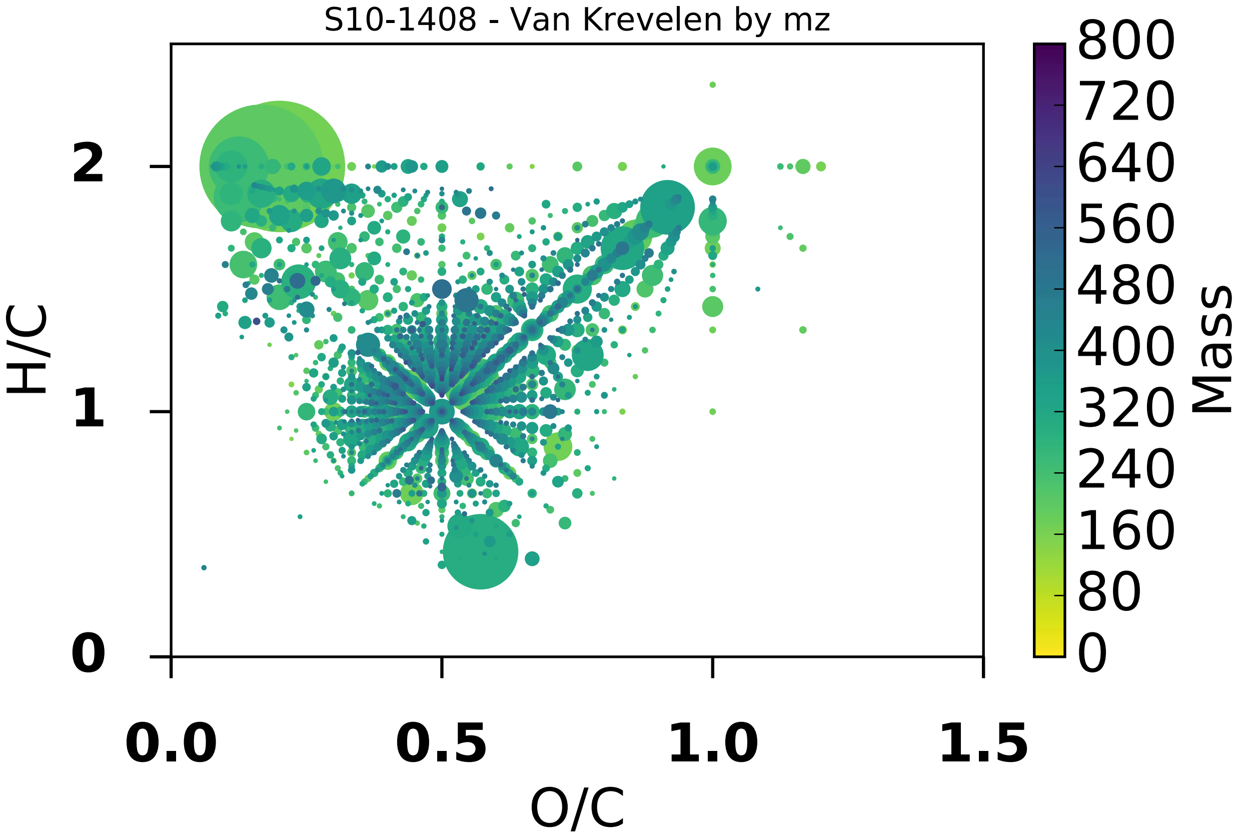
S10-1408 - Van Krevelen by mz
H/C
O/C
0
1
2
0.0
0.5
1.0
1.5
Mass
800
720
640
560
480
400
320
240
160
80
0

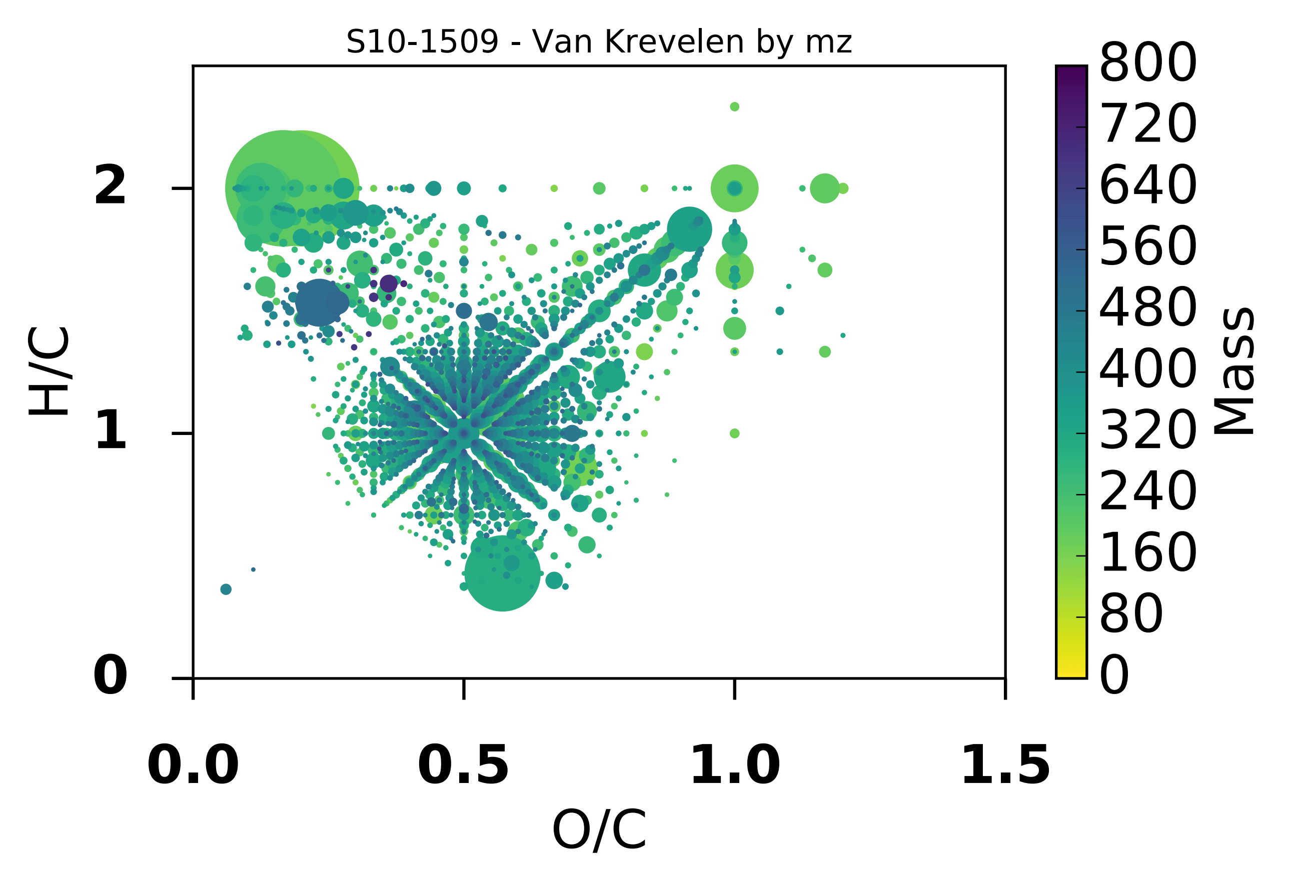
S10-1509 - Van Krevelen by mz
H/C
O/C
0
1
2
0.0
0.5
1.0
1.5
Mass
0
80
160
240
320
400
480
560
640
720
800

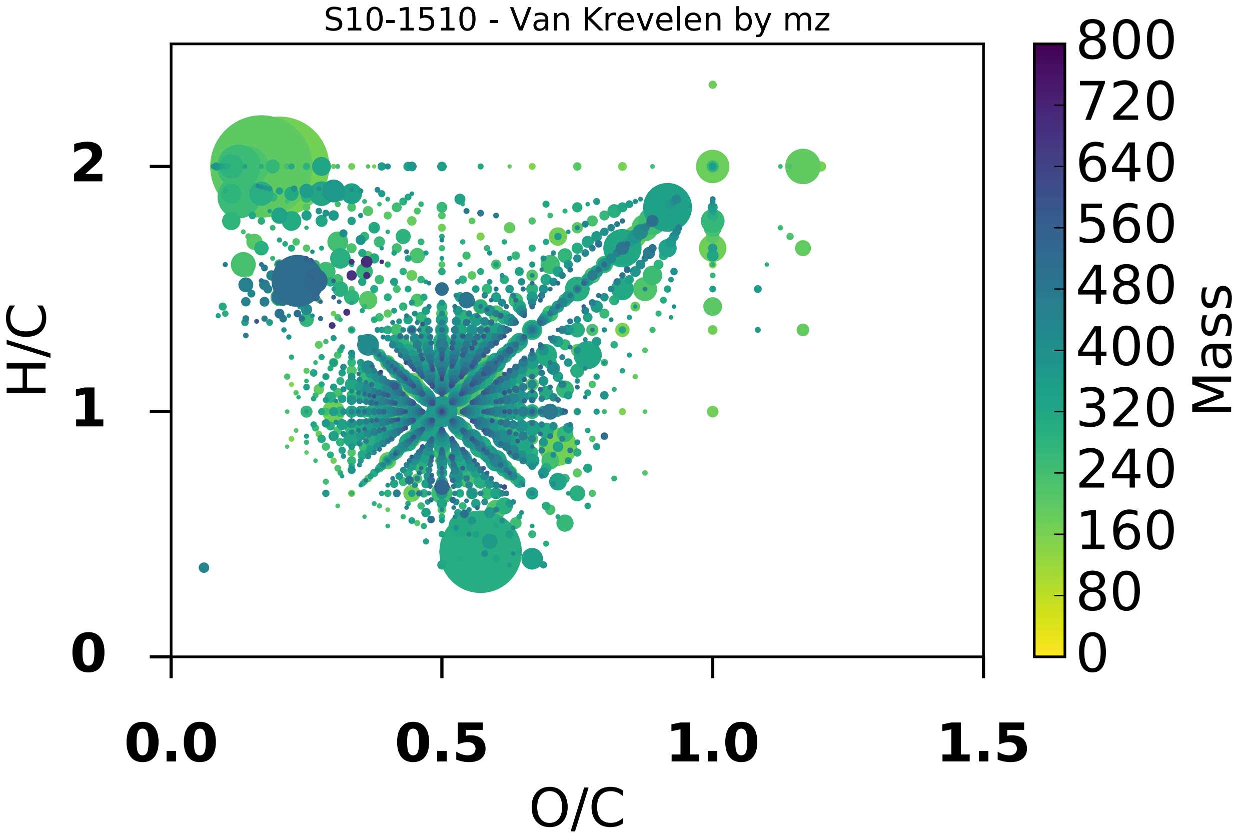
S10-1510 - Van Krevelen by mz
H/C
2
1
0
0.0
0.5
1.0
1.5
O/C
800
720
640
560
480
400
320
240
160
80
0
Mass

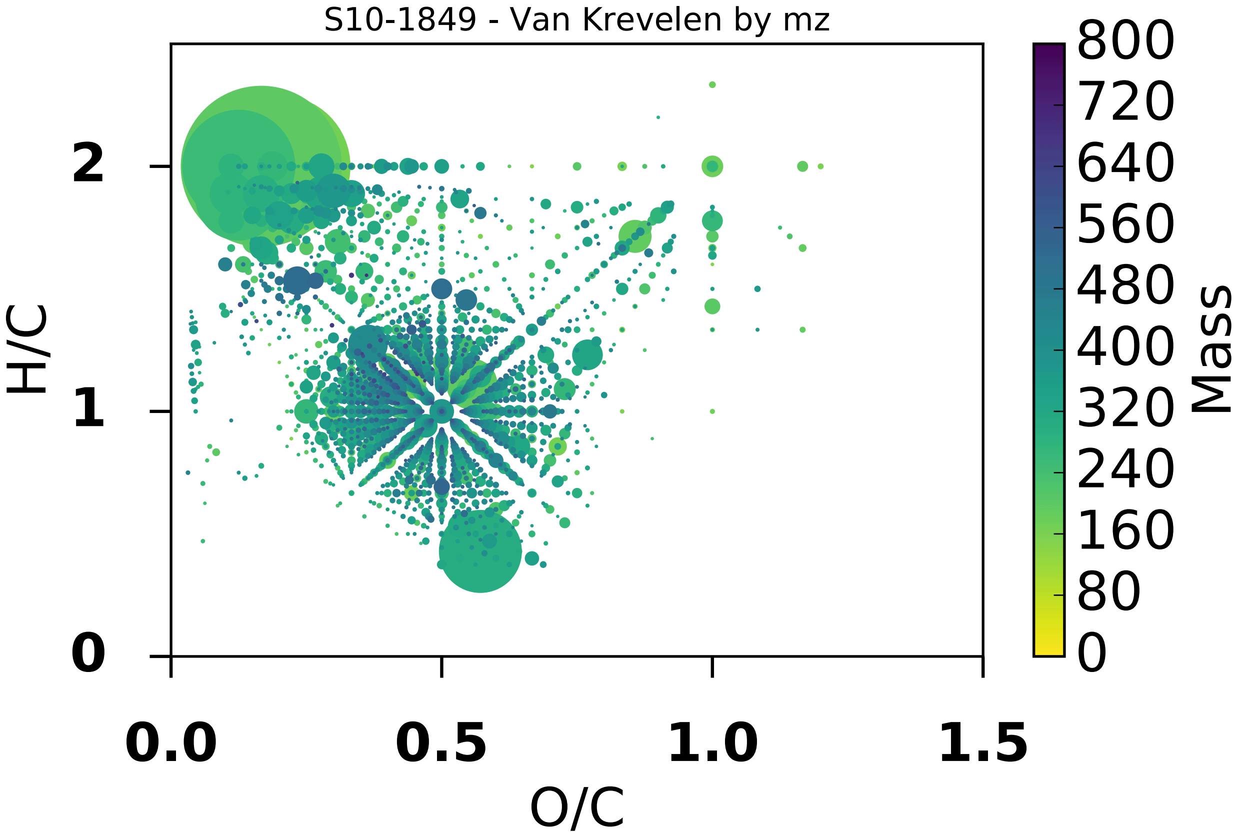

S10-1849 - Van Krevelen by mz
H/C
O/C
Mass
0
1
2
0.0
0.5
1.0
1.5
0
80
160
240
320
400
480
560
640
720
800

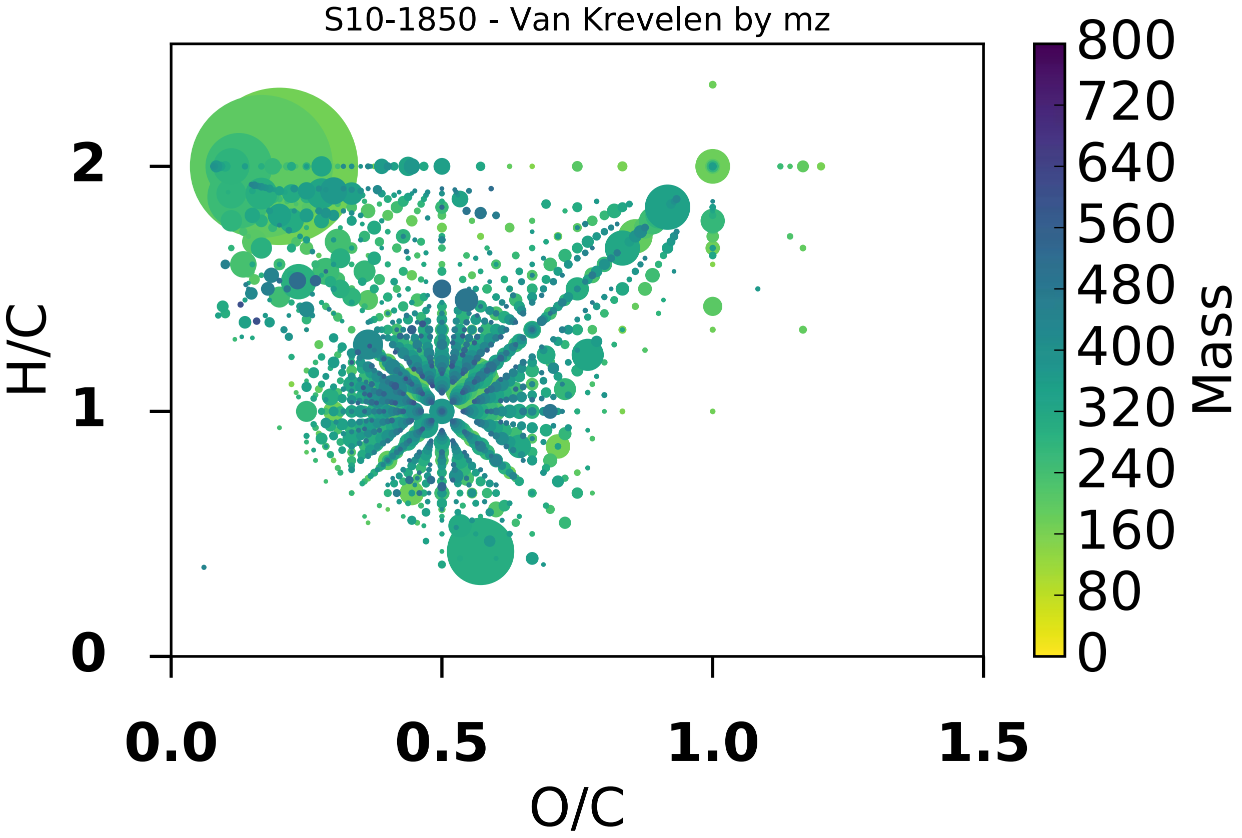
S10-1850 - Van Krevelen by mz
H/C
2
1
0
0.0
0.5
1.0
1.5
O/C
800
720
640
560
480
400
320
240
160
80
0
Mass

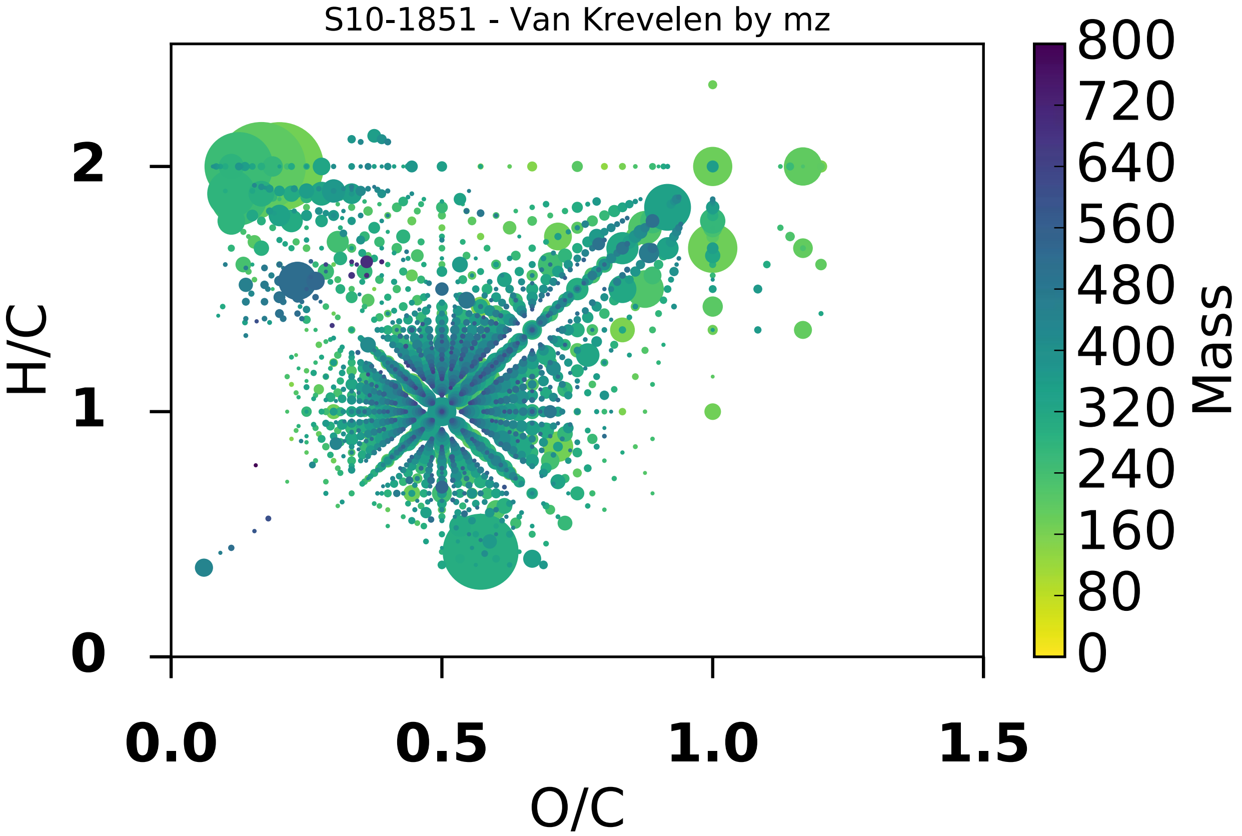

S10-1851 - Van Krevelen by mz
H/C
0
1
2
0.0
0.5
1.0
1.5
O/C
800
720
640
560
480
400
320
240
160
80
0
Mass

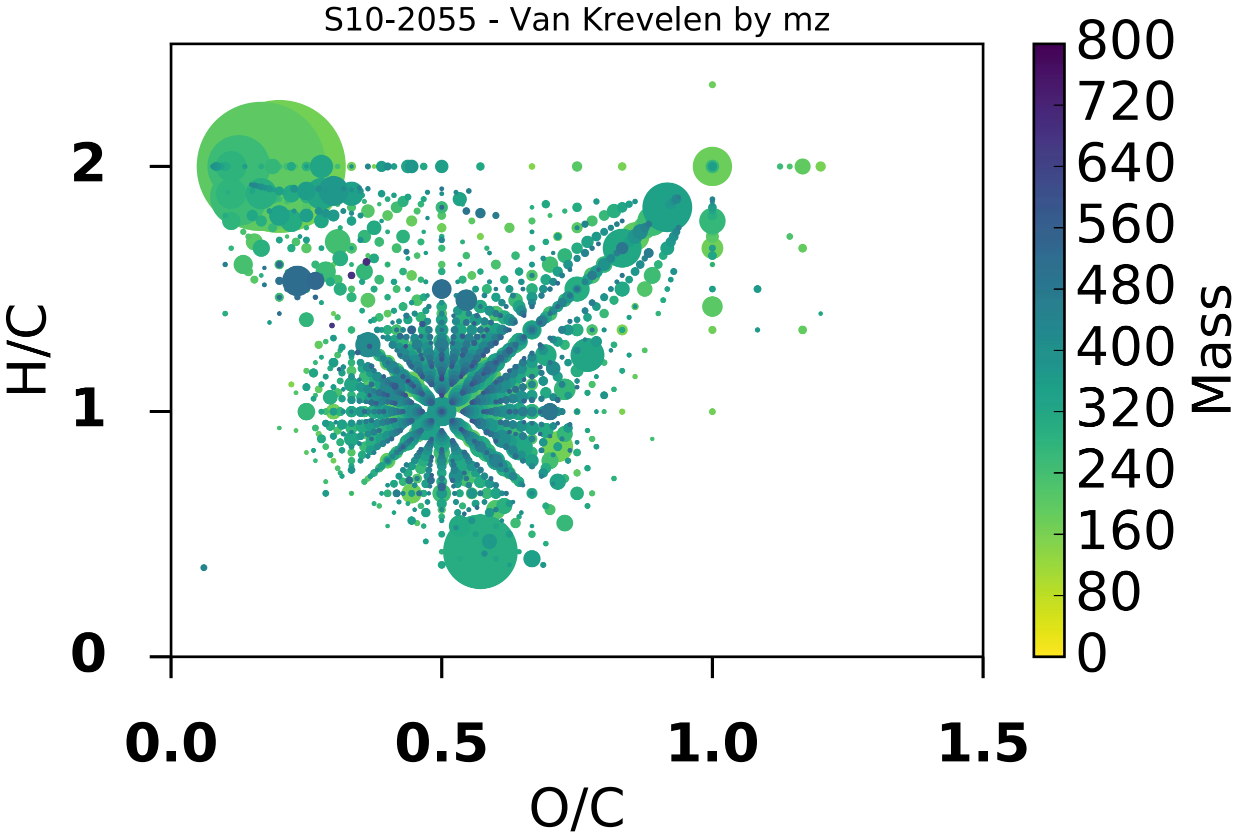

S10-2055 - Van Krevelen by mz
H/C
2
1
0
0.0
0.5
1.0
1.5
O/C
800
720
640
560
480
400
320
240
160
80
0
Mass

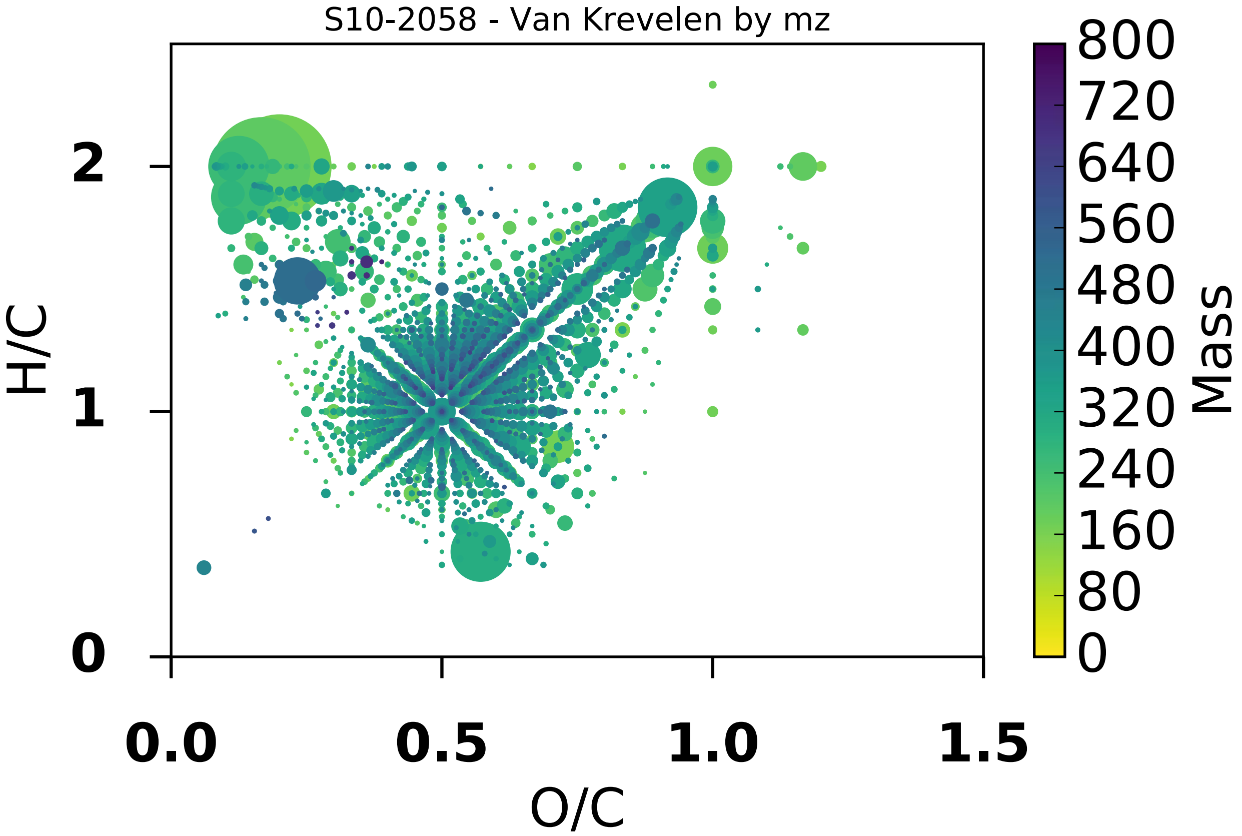

S10-2058 - Van Krevelen by mz
H/C
2
1
0
0.0
0.5
1.0
1.5
O/C
800
720
640
560
480
400
320
240
160
80
0
Mass

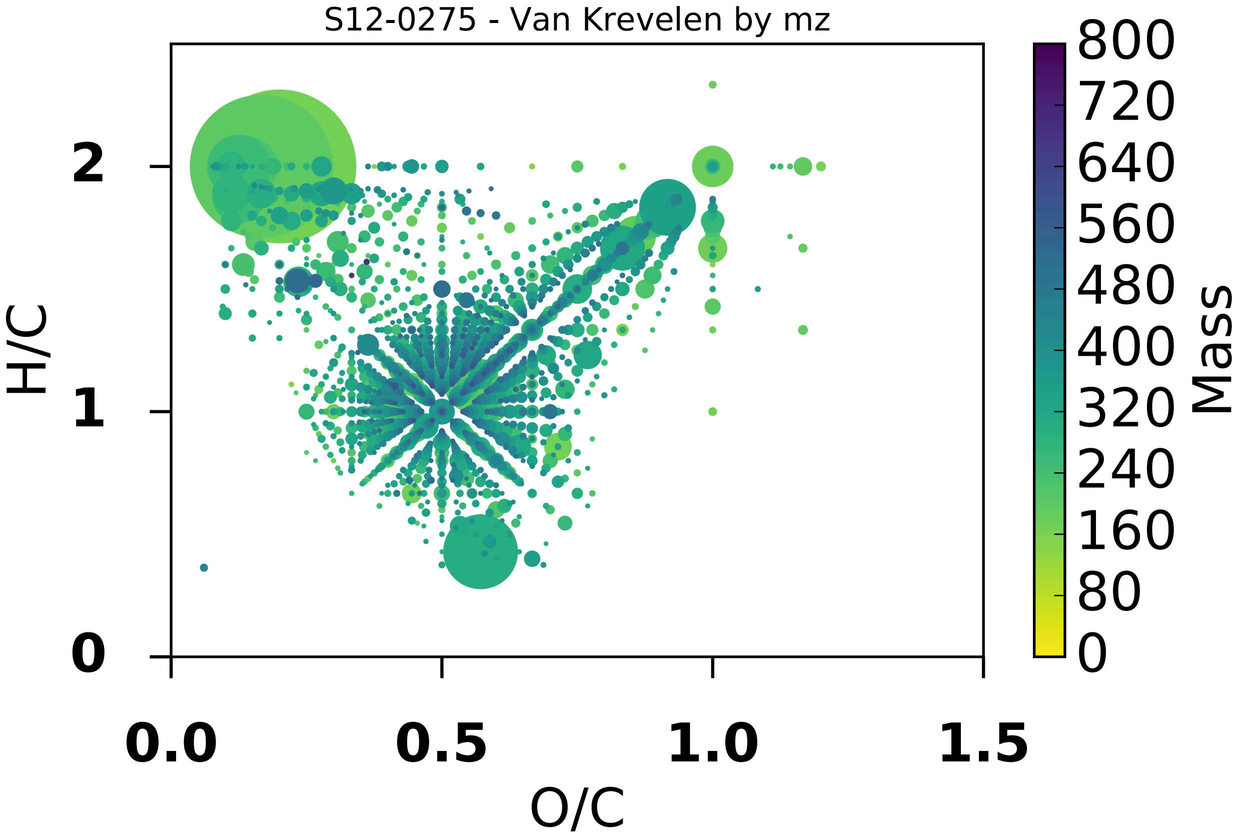

S12-0275 - Van Krevelen by mz
H/C
0
1
2
0.0
0.5
1.0
1.5
O/C
Mass
0
80
160
240
320
400
480
560
640
720
800

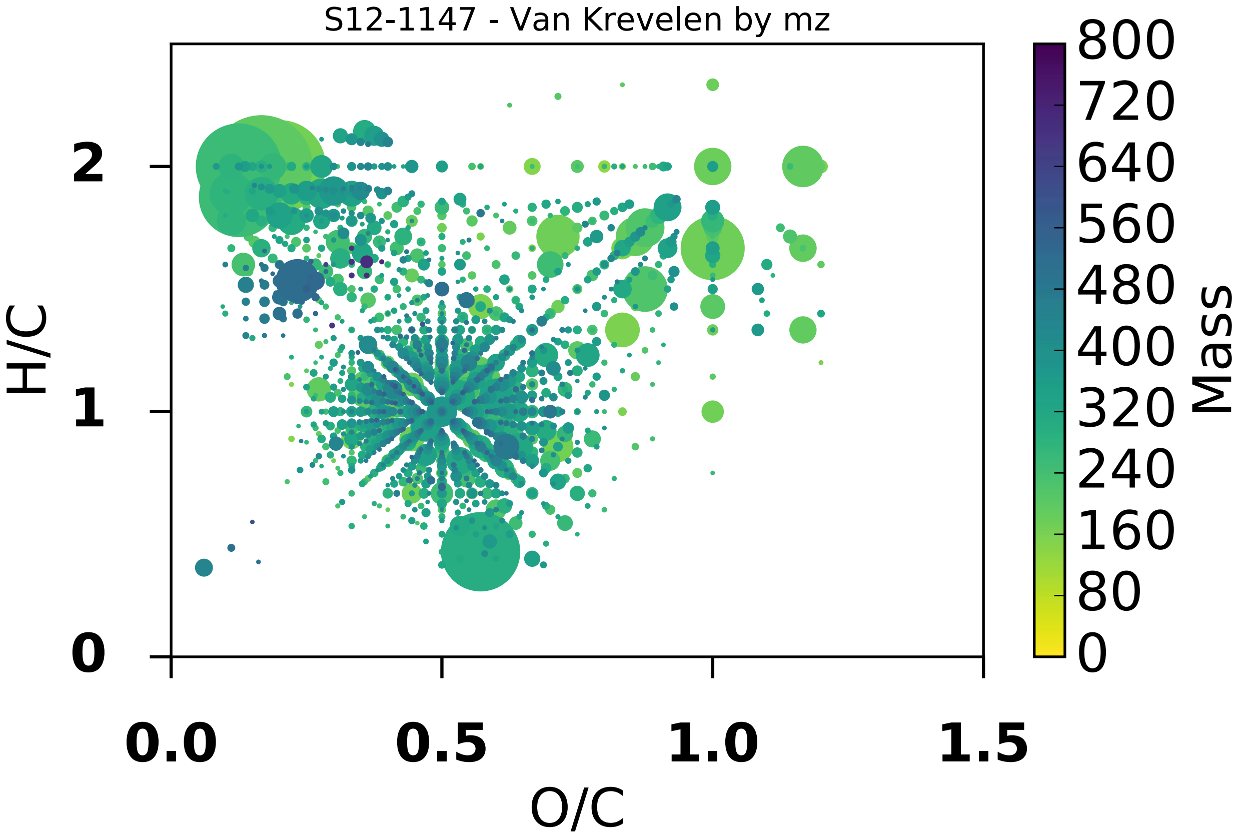

S12-1147 - Van Krevelen by mz
H/C
O/C
0
1
2
0.0
0.5
1.0
1.5
Mass
0
80
160
240
320
400
480
560
640
720
800

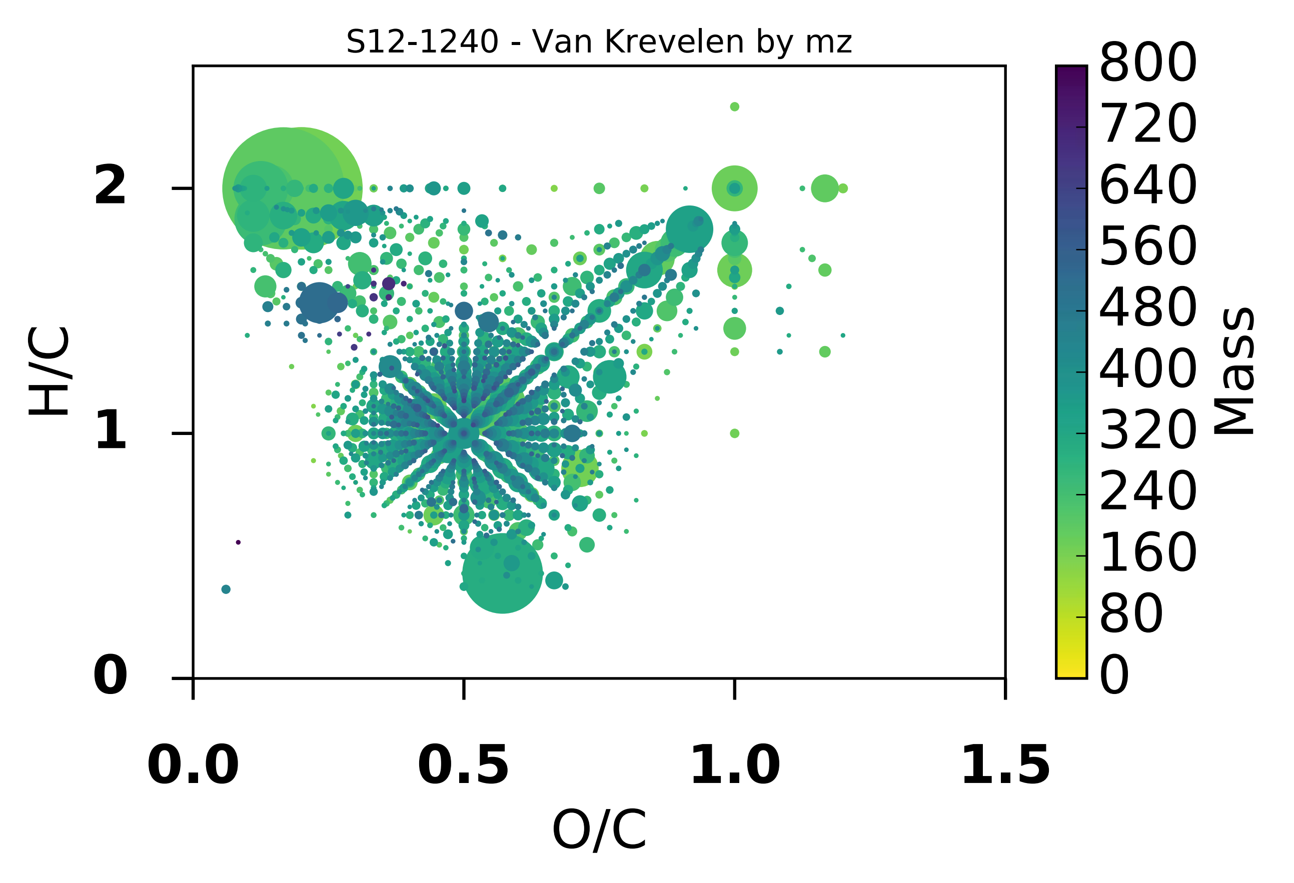

S12-1240 - Van Krevelen by mz
H/C
0
1
2
O/C
0.0
0.5
1.0
1.5
Mass
0
80
160
240
320
400
480
560
640
720
800

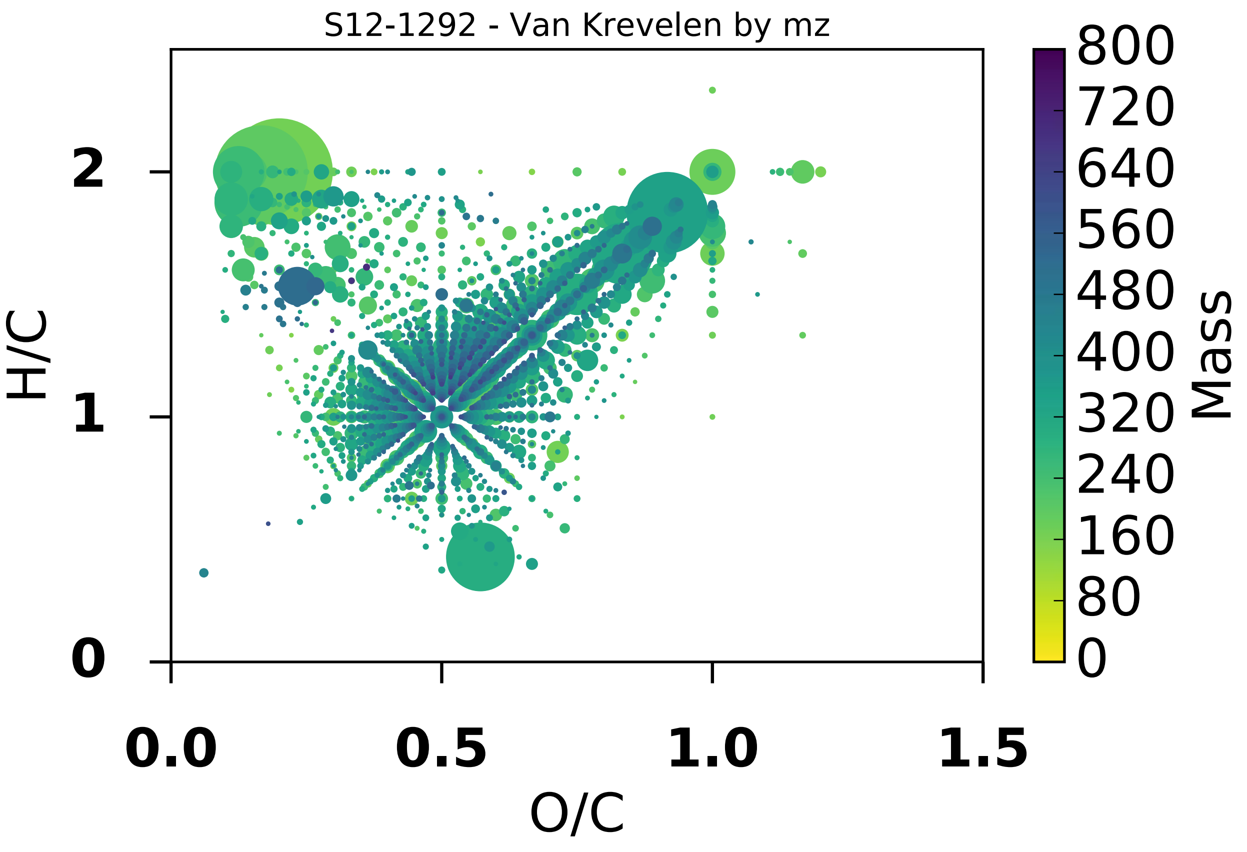

S12-1292 - Van Krevelen by mz
H/C
O/C
2
1
0
0.0
0.5
1.0
1.5
Mass
800
720
640
560
480
400
320
240
160
80
0

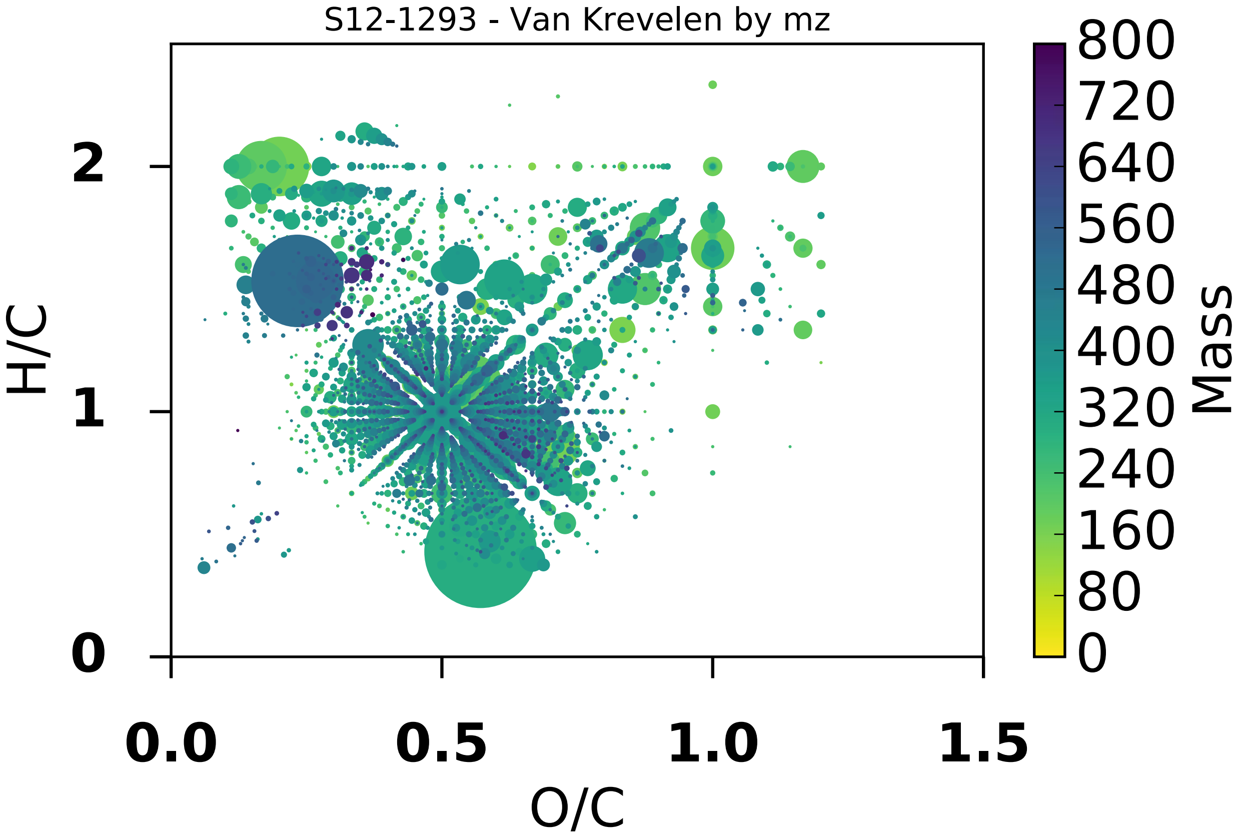
S12-1293 - Van Krevelen by mz
H/C
2
1
0
0.0
0.5
1.0
1.5
O/C
800
720
640
560
480
400
320
240
160
80
0
Mass

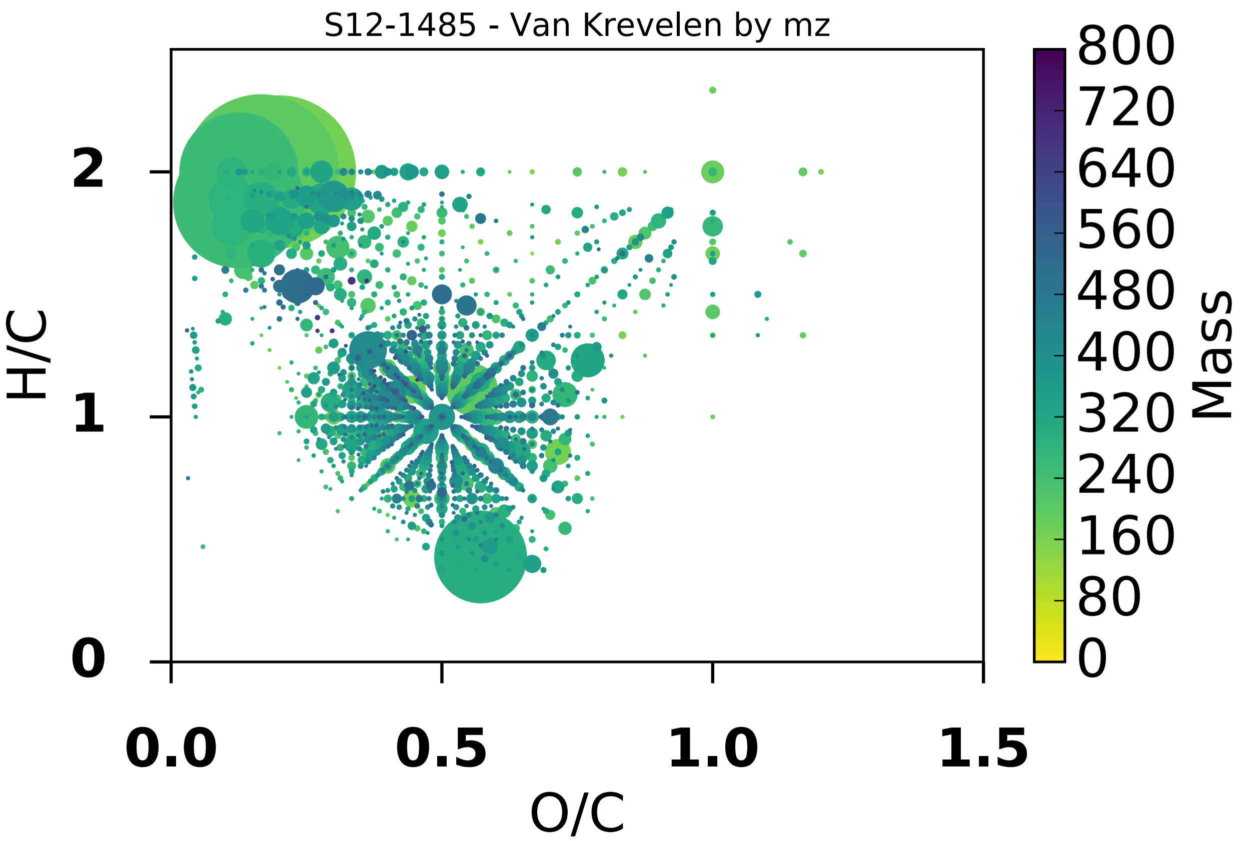

S12-1485 - Van Krevelen by mz
H/C
O/C
0.0
0.5
1.0
1.5
0
1
2
Mass
800
720
640
560
480
400
320
240
160
80
0

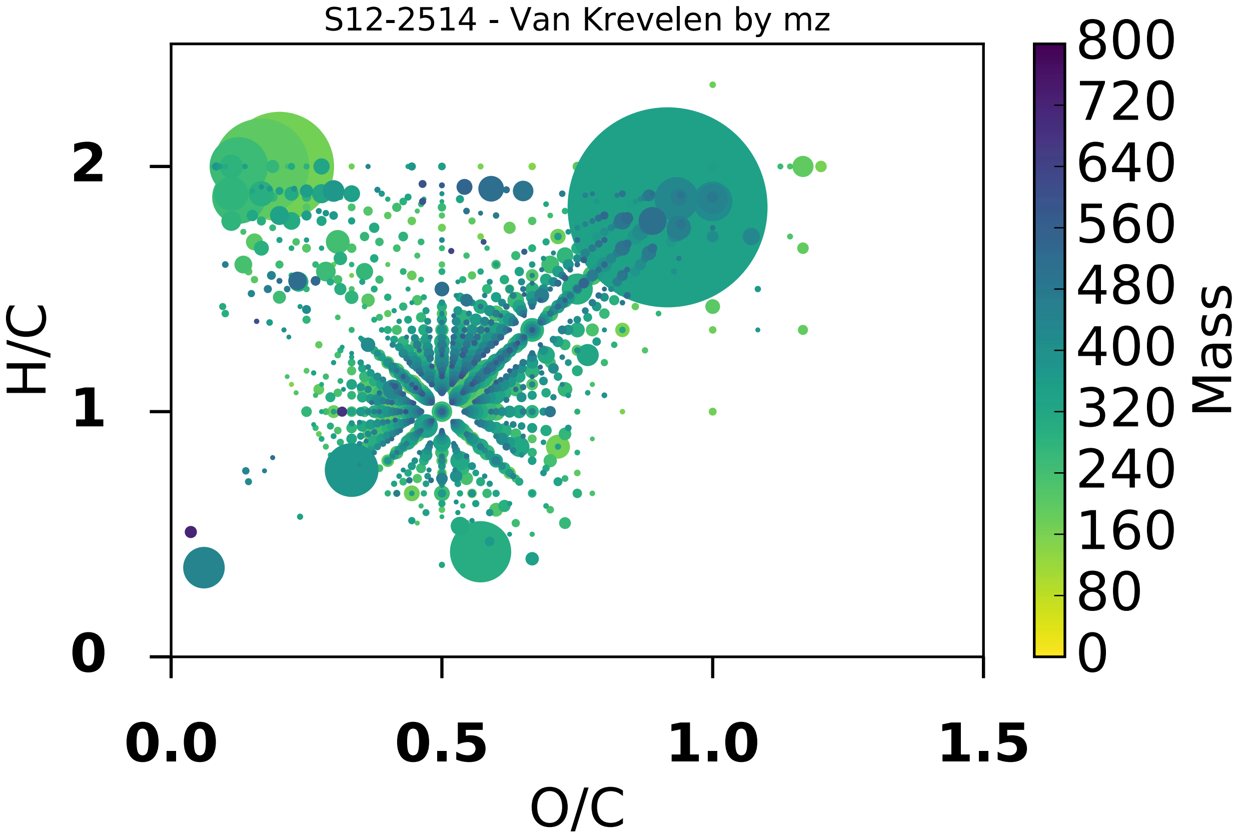

S12-2514 - Van Krevelen by mz
H/C
O/C
Mass
0
1
2
0.0
0.5
1.0
1.5
800
720
640
560
480
400
320
240
160
80
0

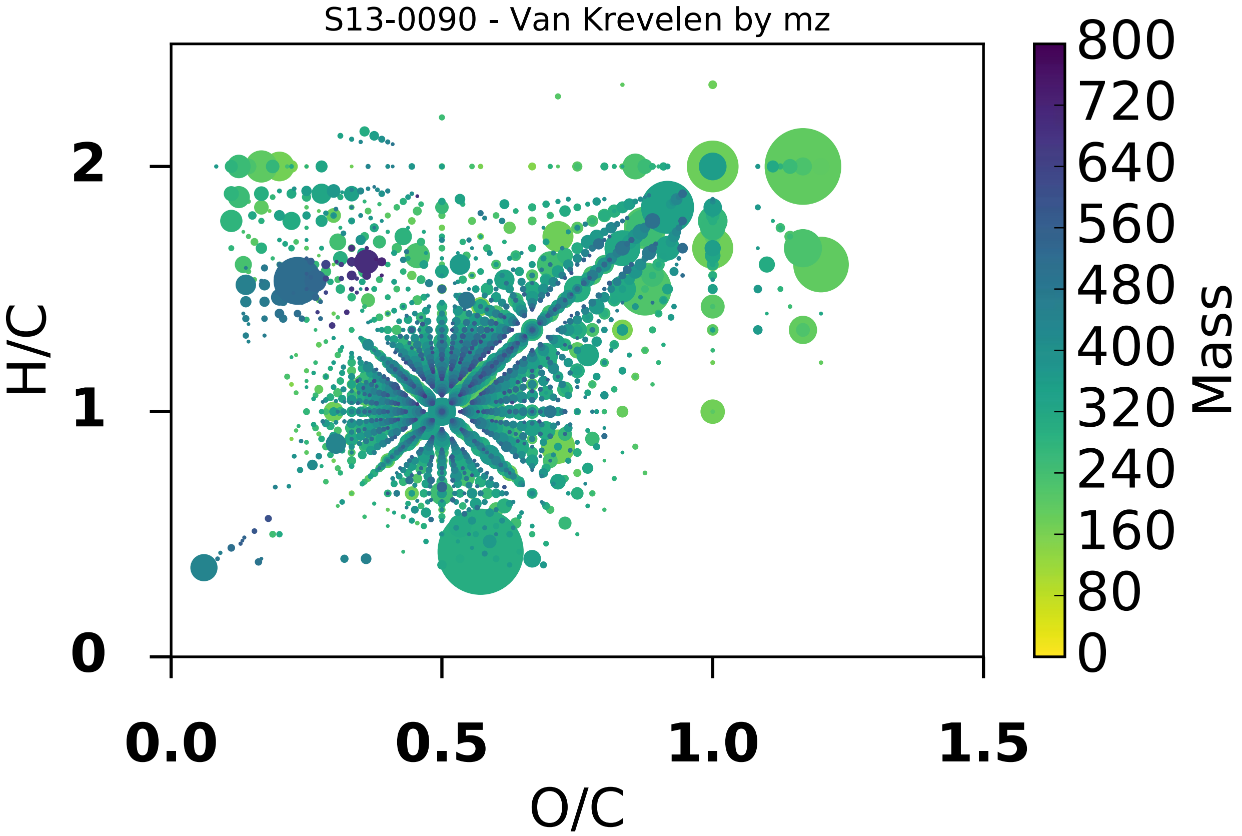

S13-0090 - Van Krevelen by mz
H/C
2
1
0
0.0
0.5
1.0
1.5
O/C
800
720
640
560
480
400
320
240
160
80
0
Mass

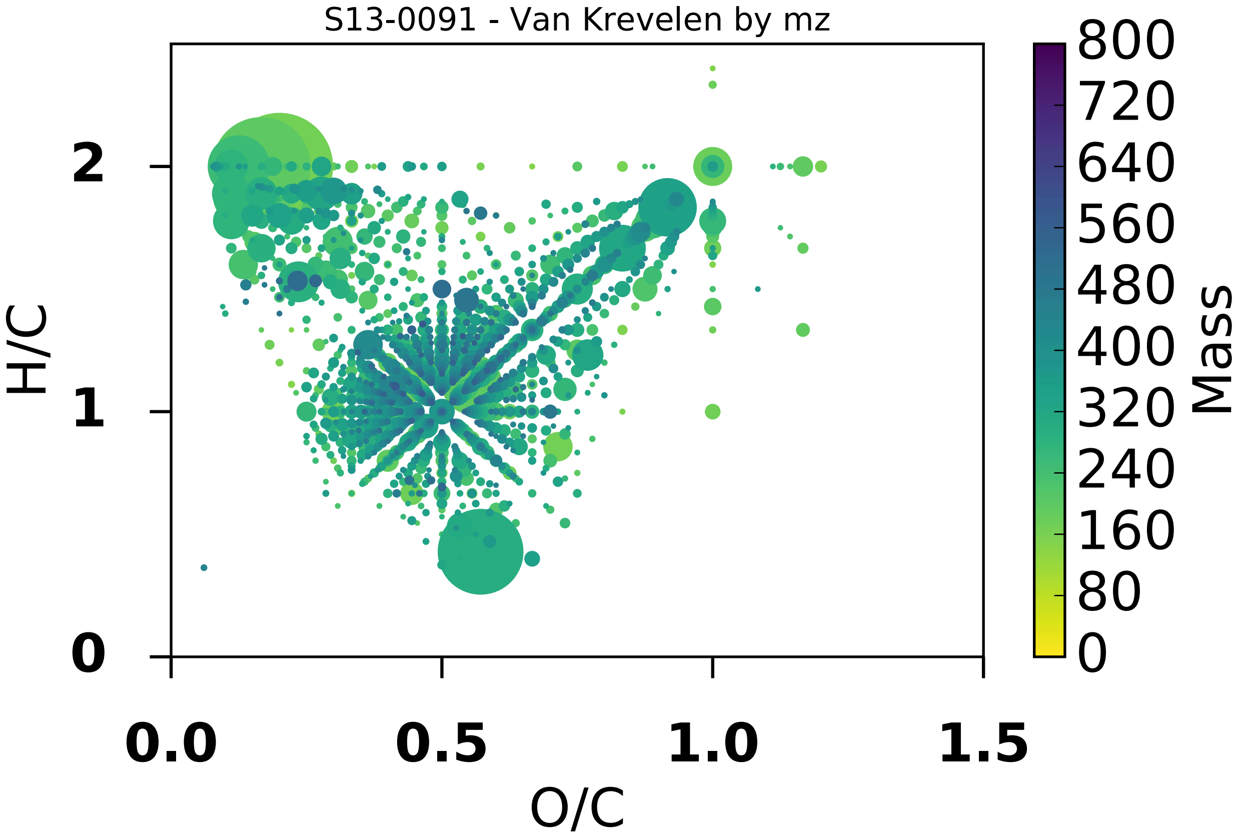
S13-0091 - Van Krevelen by mz
H/C
2
1
0
0.0
0.5
1.0
1.5
O/C
800
720
640
560
480
400
320
240
160
80
0
Mass

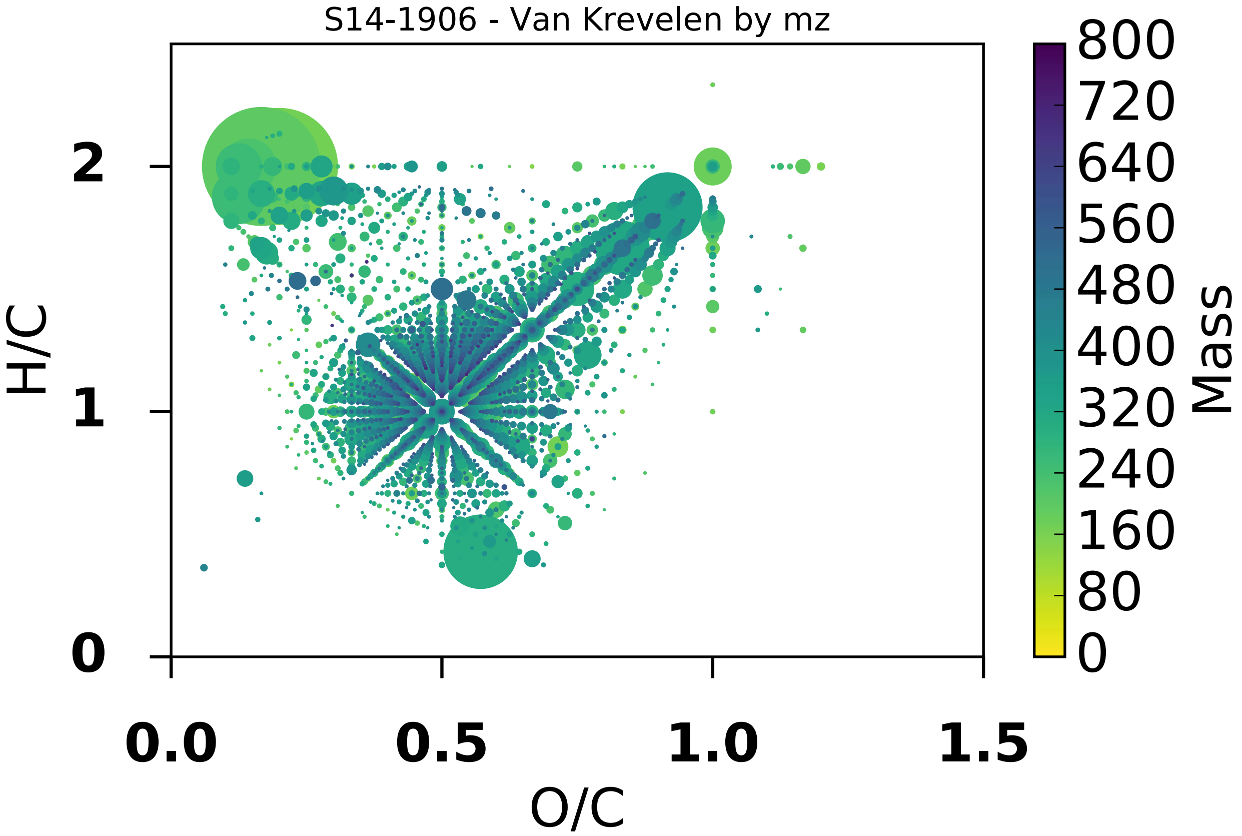
S14-1906 - Van Krevelen by mz
H/C
O/C
Mass
0
1
2
0.0
0.5
1.0
1.5
800
720
640
560
480
400
320
240
160
80
0

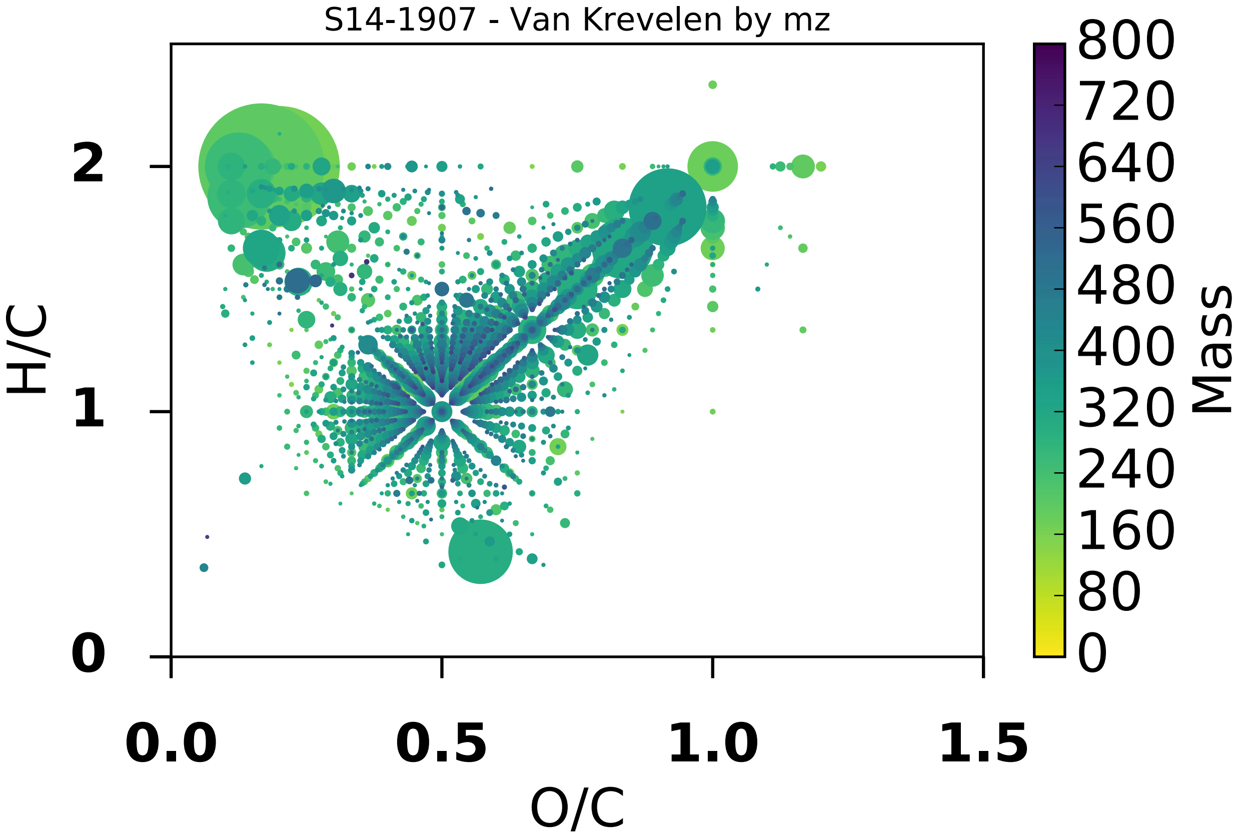
S14-1907 - Van Krevelen by mz
H/C
2
1
0
0.0
0.5
1.0
1.5
O/C
800
720
640
560
480
400
320
240
160
80
0
Mass

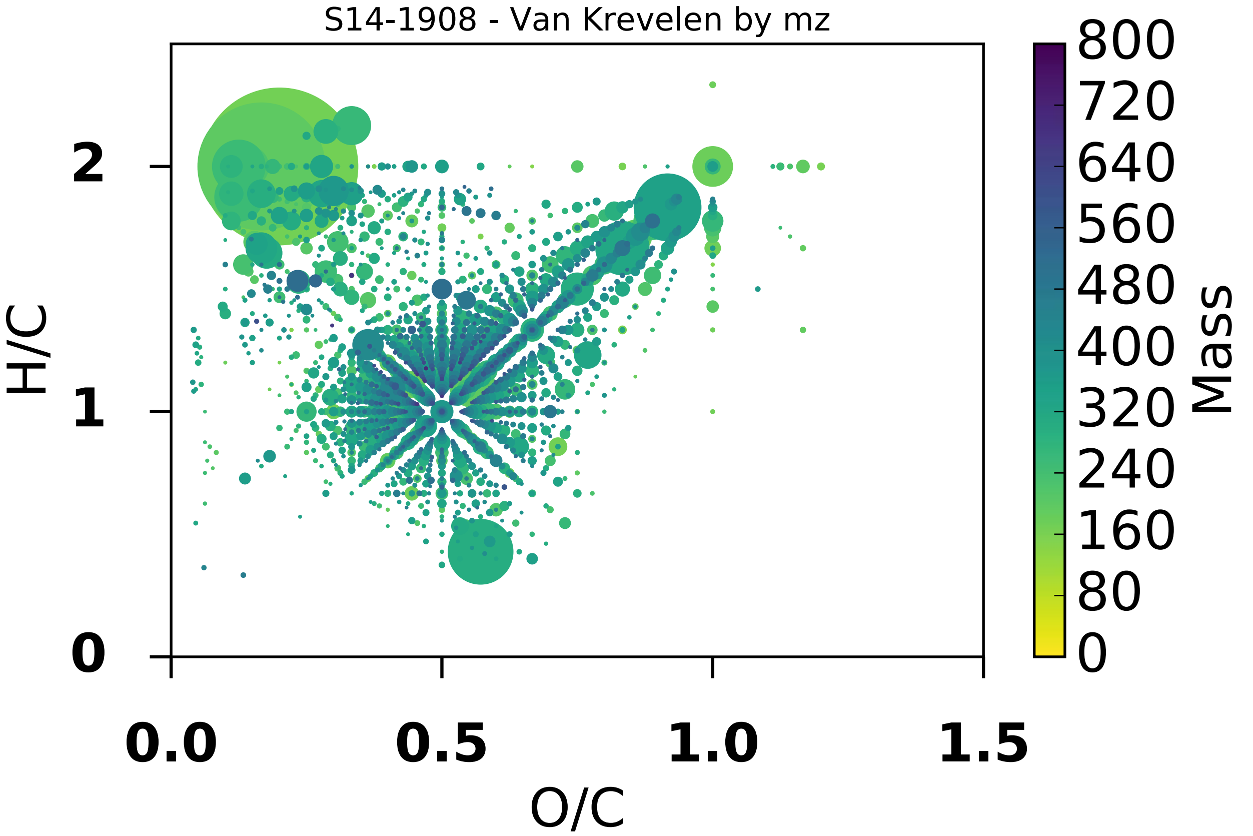

S14-1908 - Van Krevelen by mz
H/C
2
1
0
0.0
0.5
1.0
1.5
O/C
800
720
640
560
480
400
320
240
160
80
0
Mass

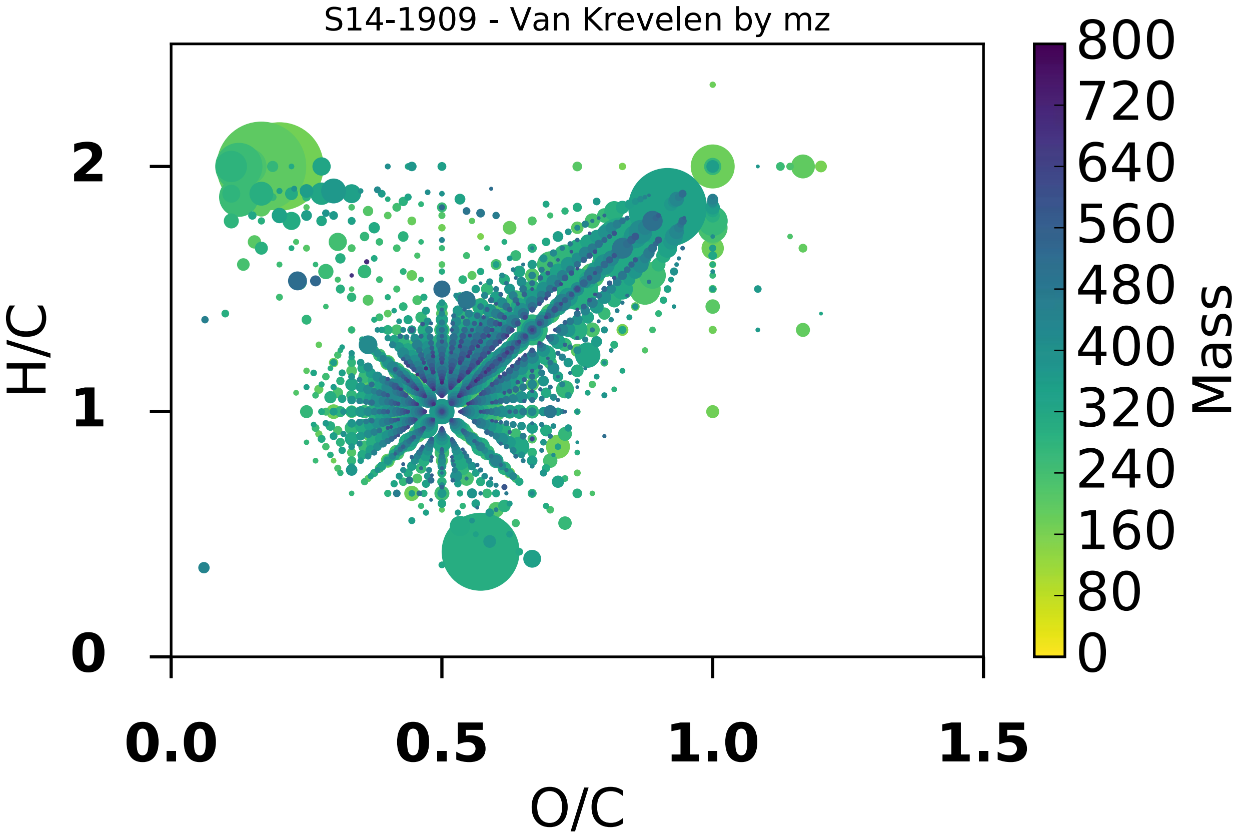

S14-1909 - Van Krevelen by mz
H/C
O/C
0
1
2
0.0
0.5
1.0
1.5
Mass
0
80
160
240
320
400
480
560
640
720
800

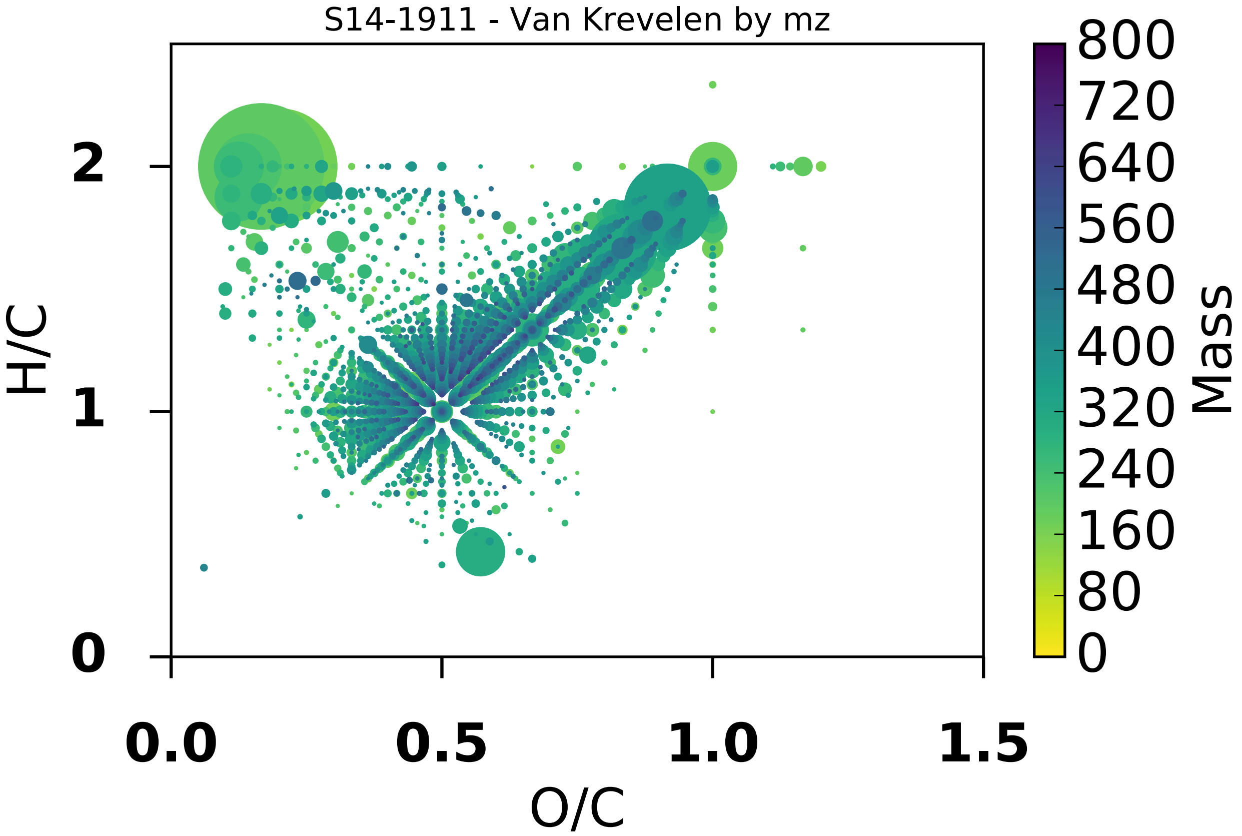

S14-1911 - Van Krevelen by mz
H/C
2
1
0
0.0
0.5
1.0
1.5
O/C
800
720
640
560
480
400
320
240
160
80
0
Mass

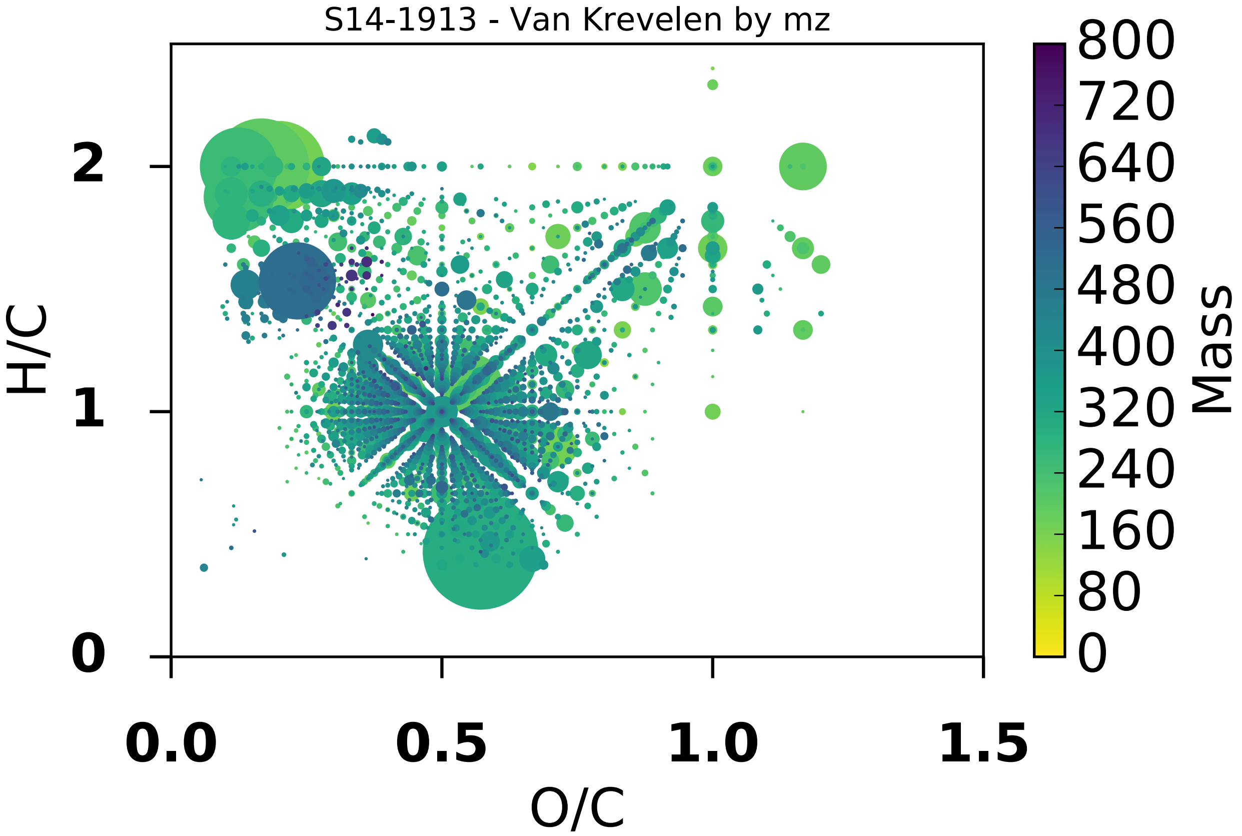
S14-1913 - Van Krevelen by mz
H/C
O/C
0
1
2
0.0
0.5
1.0
1.5
Mass
0
80
160
240
320
400
480
560
640
720
800

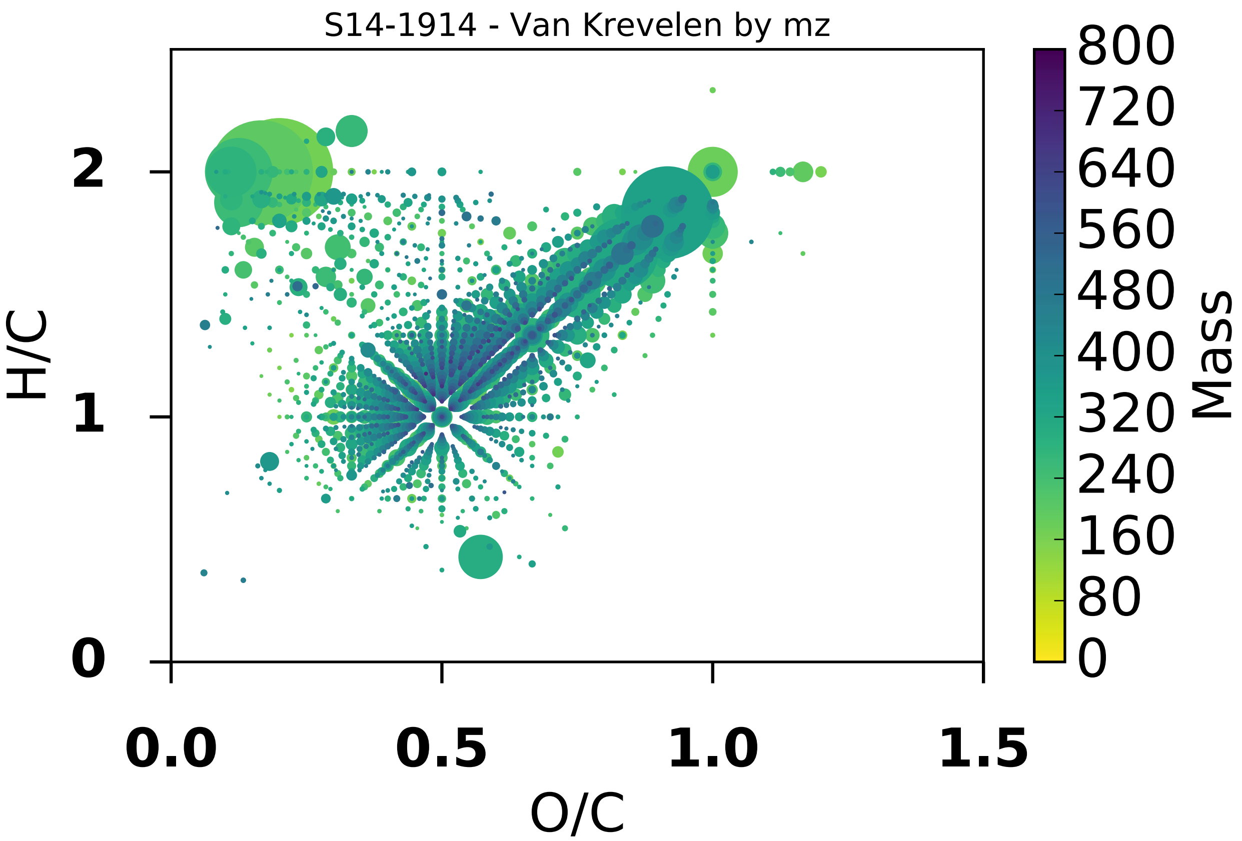
S14-1914 - Van Krevelen by mz
H/C
2
1
0
0.0
0.5
1.0
1.5
O/C
800
720
640
560
480
400
320
240
160
80
0
Mass

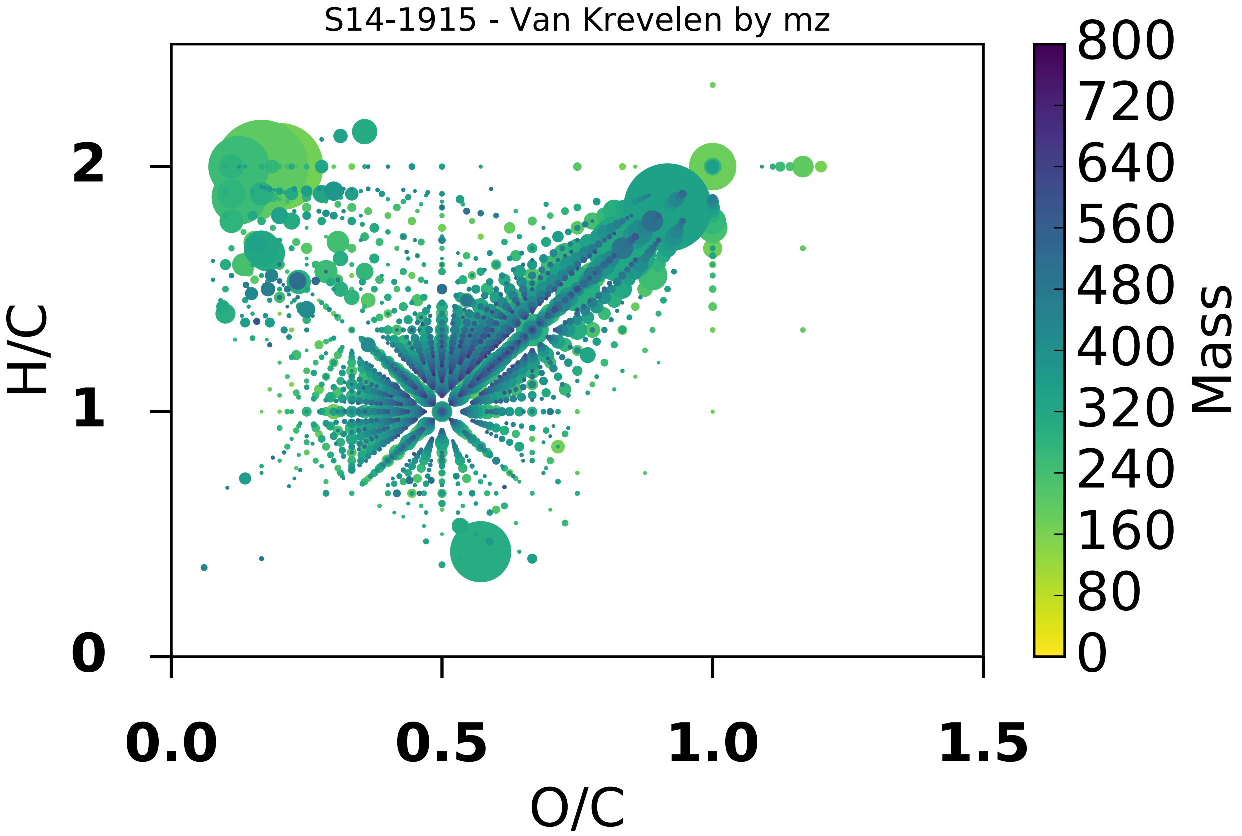
S14-1915 - Van Krevelen by mz
H/C
2
1
0
0.0
0.5
1.0
1.5
O/C
800
720
640
560
480
400
320
240
160
80
0
Mass

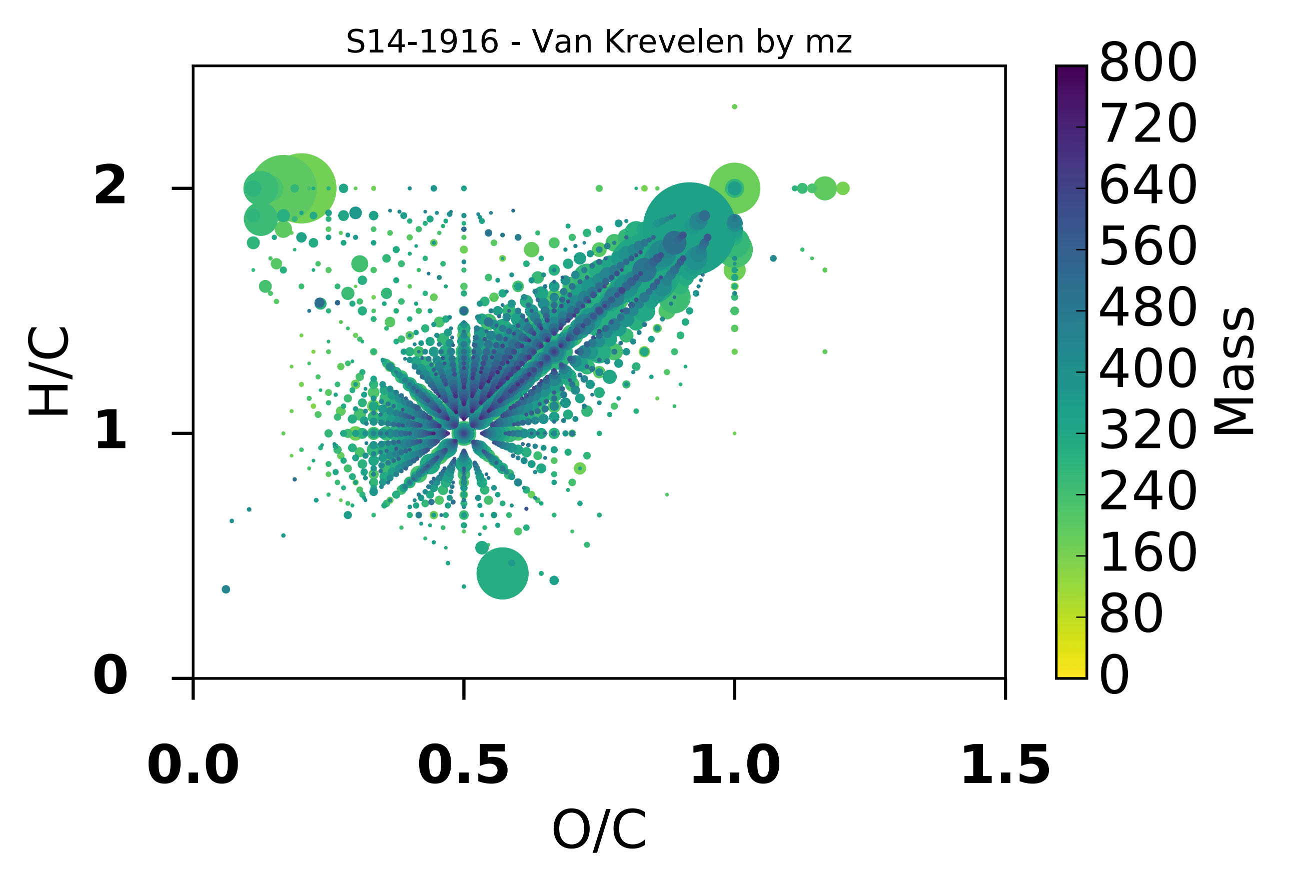

S14-1916 - Van Krevelen by mz
H/C
0
1
2
0.0
0.5
1.0
1.5
O/C
Mass
0
80
160
240
320
400
480
560
640
720
800

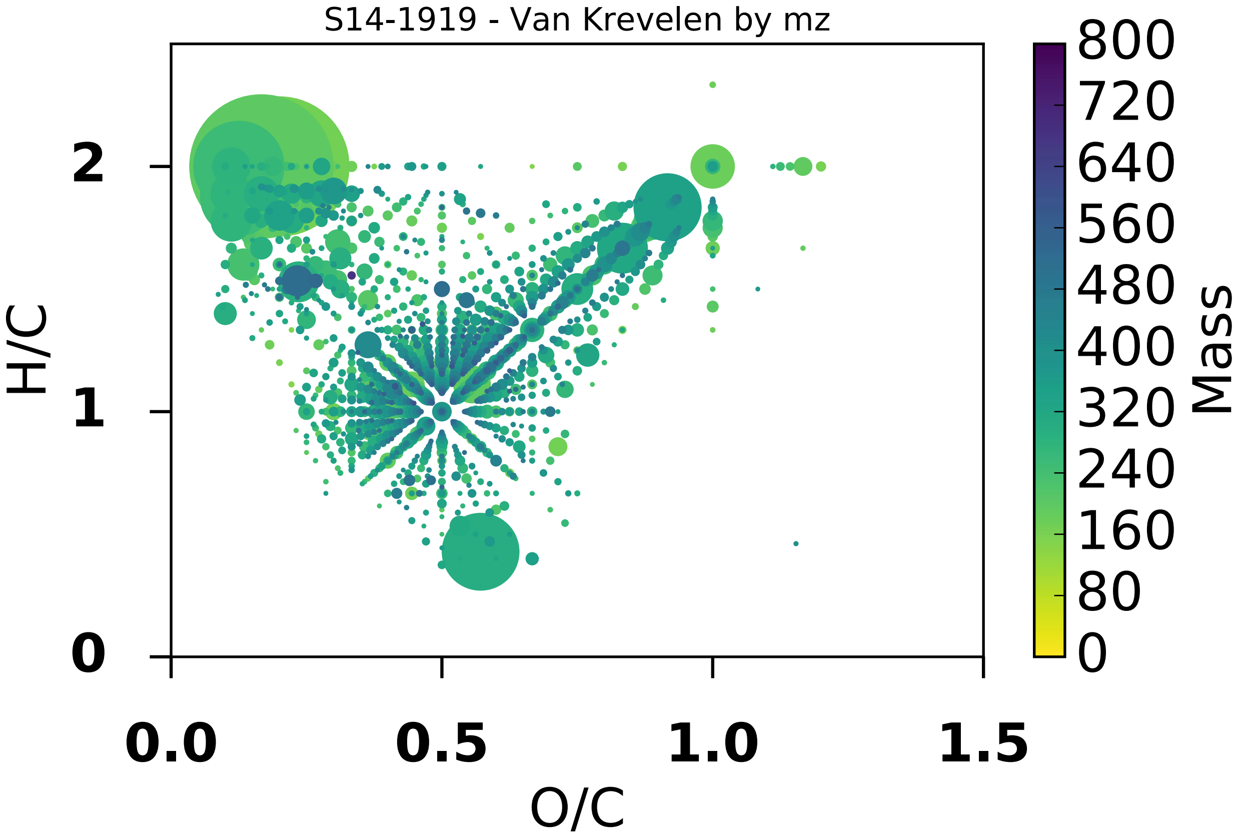
S14-1919 - Van Krevelen by mz
H/C
O/C
0
1
2
0.0
0.5
1.0
1.5
Mass
800
720
640
560
480
400
320
240
160
80
0

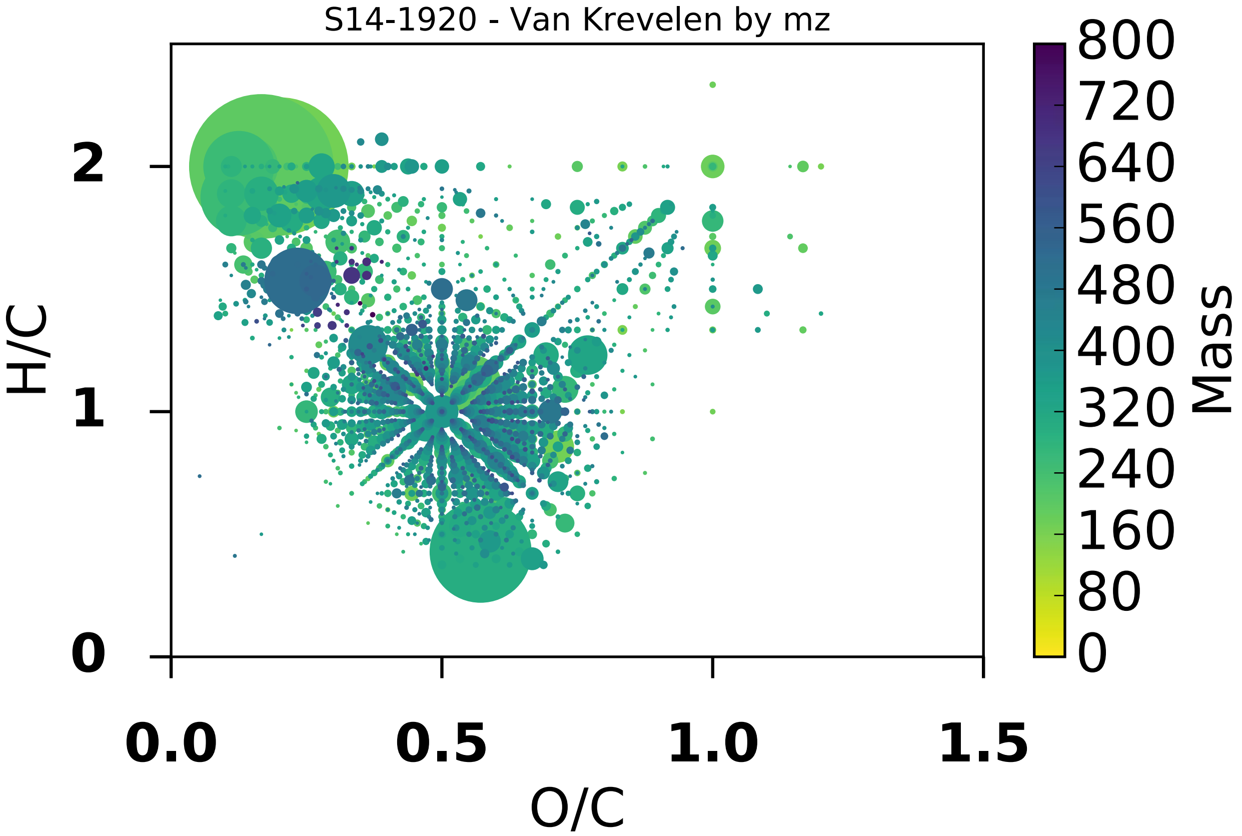
S14-1920 - Van Krevelen by mz
H/C
2
1
0
0.0
0.5
1.0
1.5
O/C
800
720
640
560
480
400
320
240
160
80
0
Mass

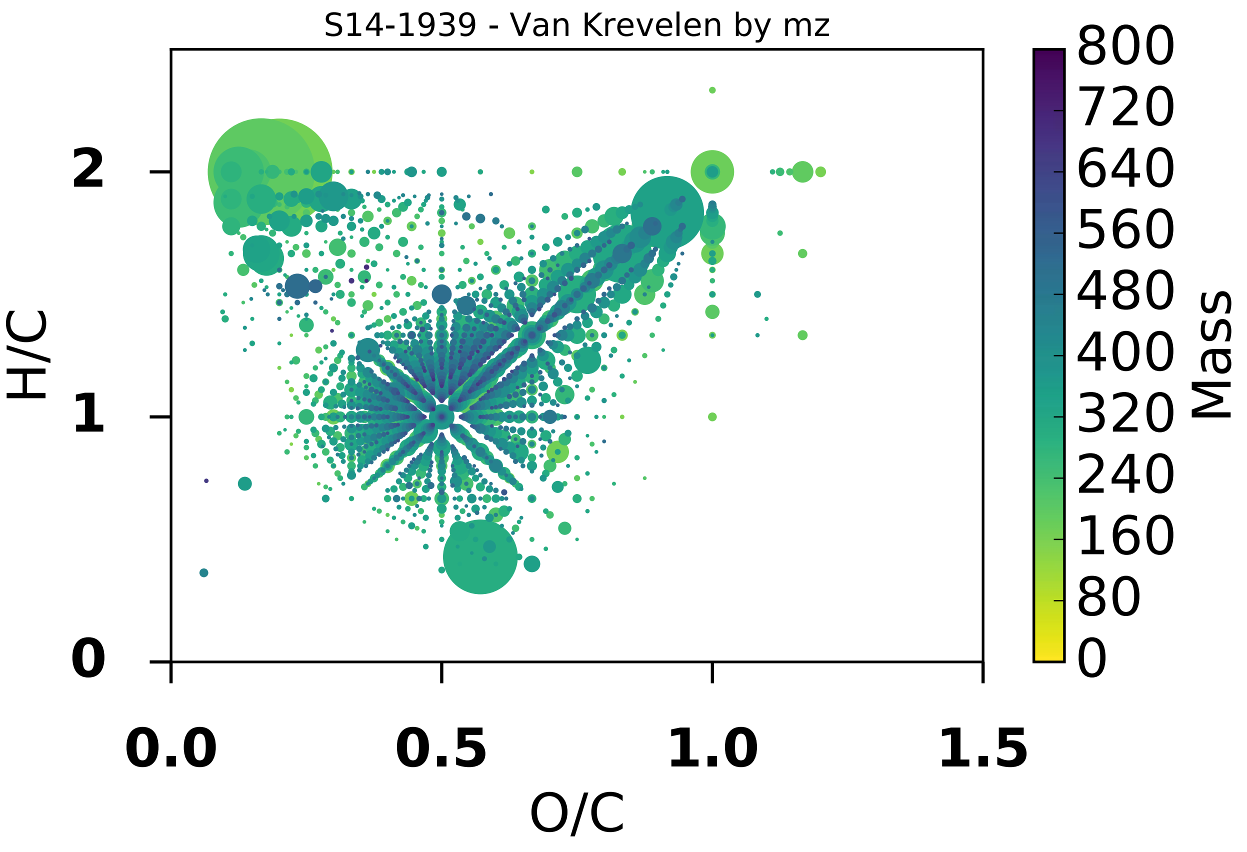

S14-1939 - Van Krevelen by mz
H/C
O/C
0
1
2
0.0
0.5
1.0
1.5
Mass
800
720
640
560
480
400
320
240
160
80
0

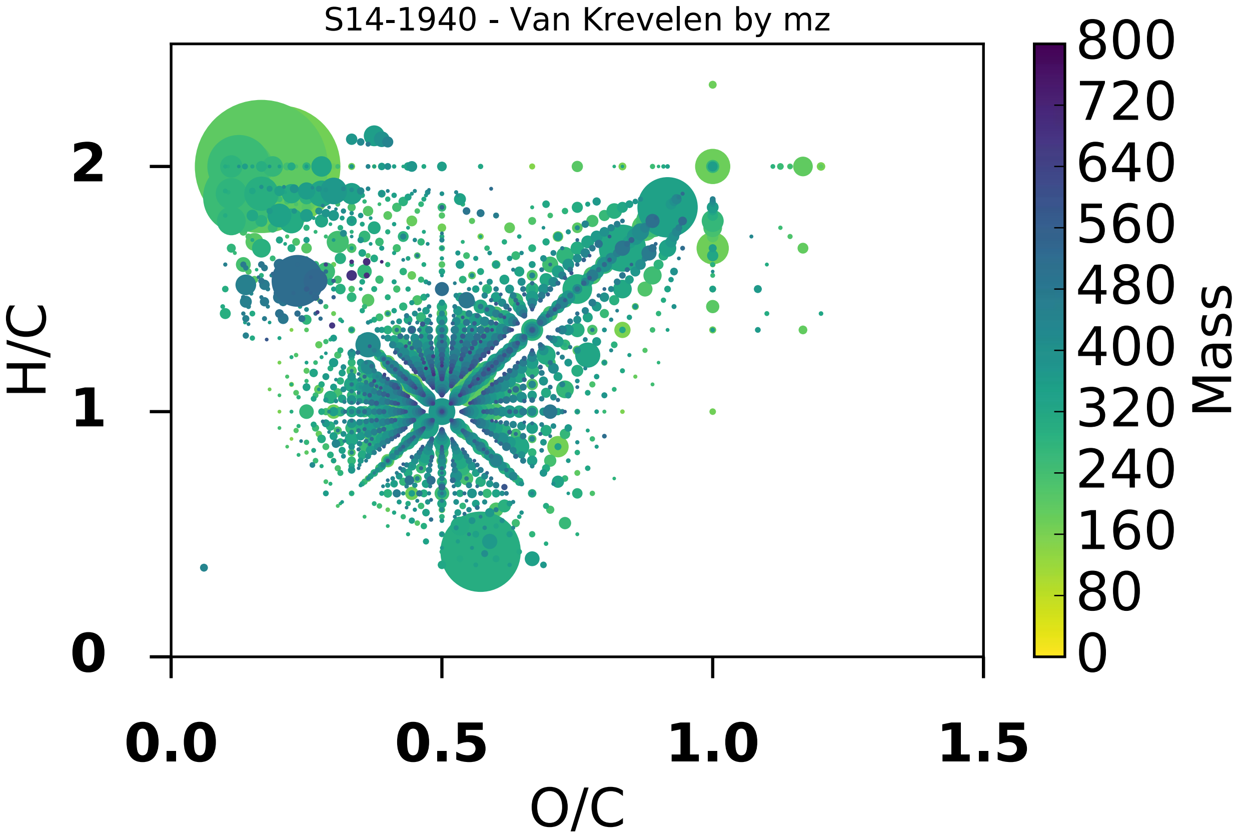
S14-1940 - Van Krevelen by mz
H/C
O/C
0
1
2
0.0
0.5
1.0
1.5
Mass
800
720
640
560
480
400
320
240
160
80
0

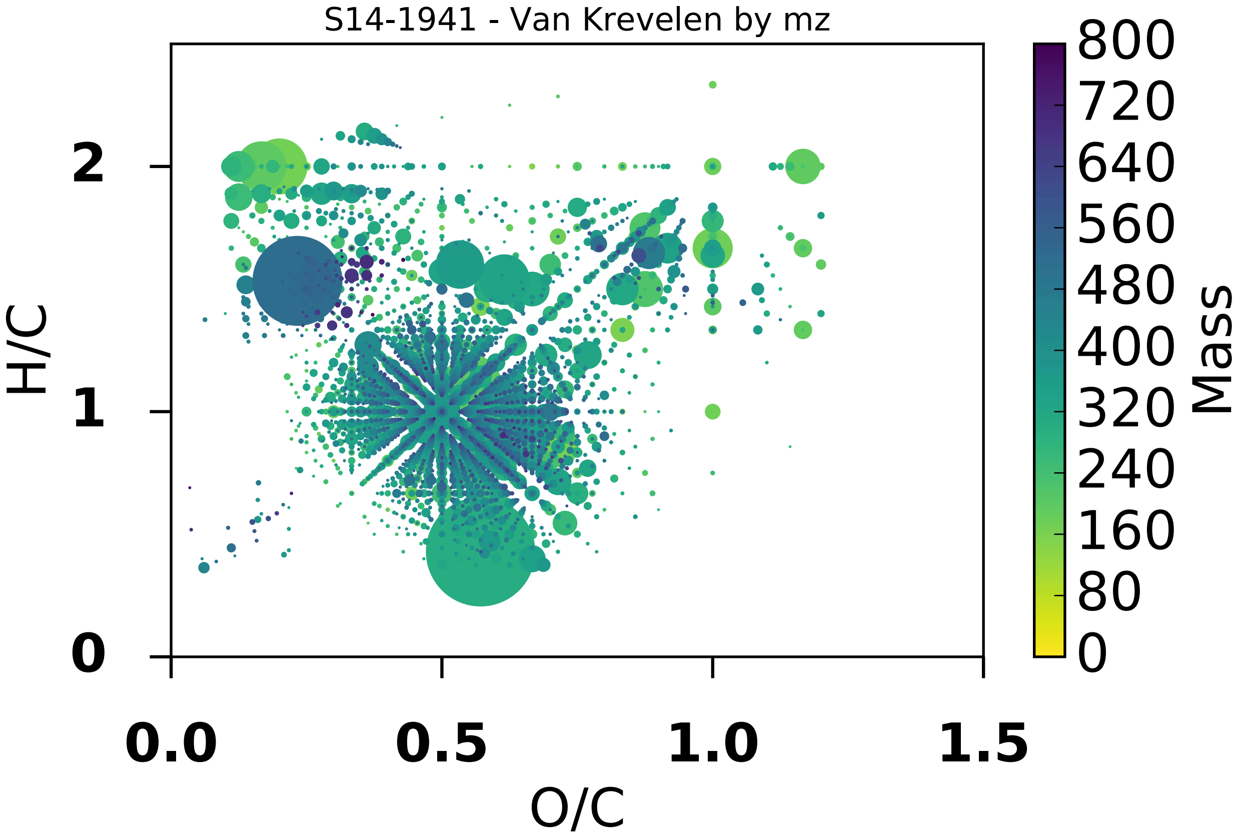

S14-1941 - Van Krevelen by mz
H/C
2
1
0
0.0
0.5
1.0
1.5
O/C
800
720
640
560
480
400
320
240
160
80
0
Mass

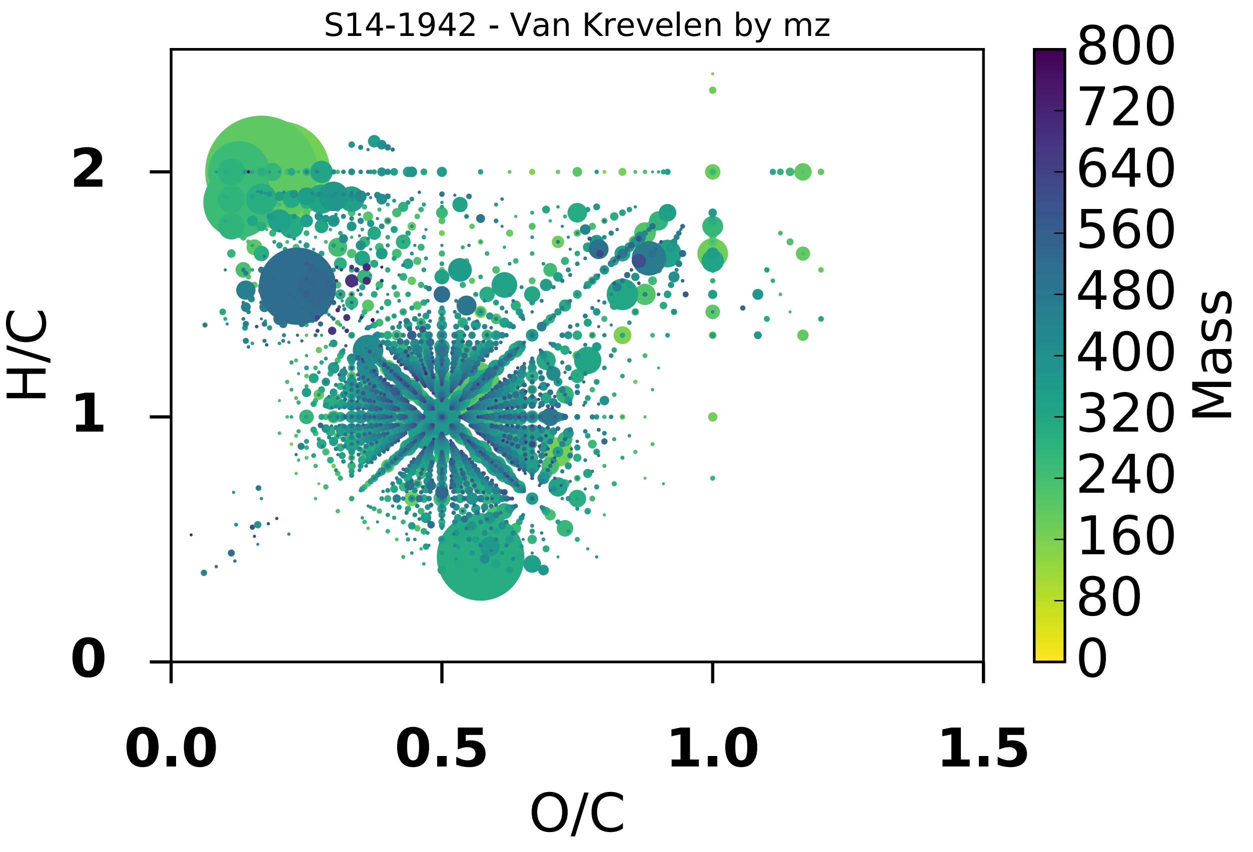

S14-1942 - Van Krevelen by mz
H/C
O/C
0
1
2
0.0
0.5
1.0
1.5
Mass
0
80
160
240
320
400
480
560
640
720
800

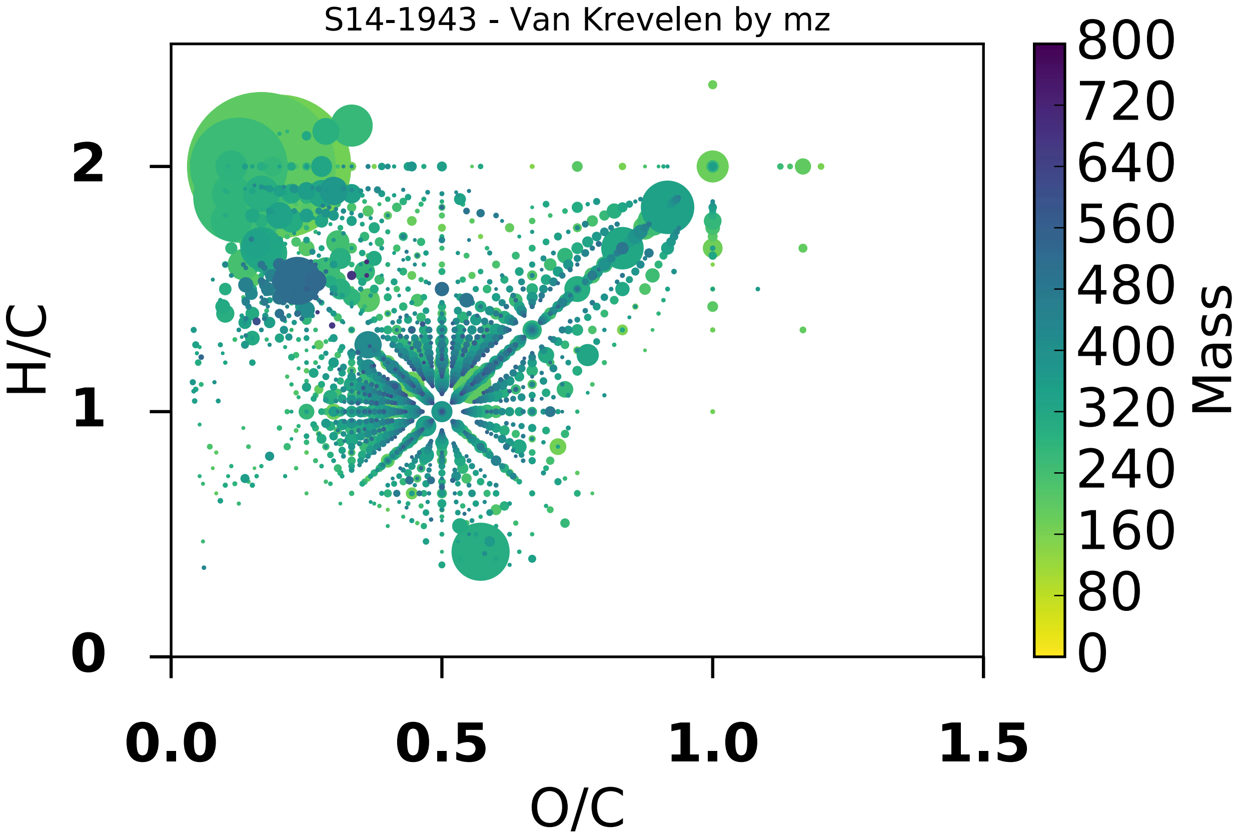
S14-1943 - Van Krevelen by mz
H/C
O/C
0
1
2
0.0
0.5
1.0
1.5
Mass
800
720
640
560
480
400
320
240
160
80
0

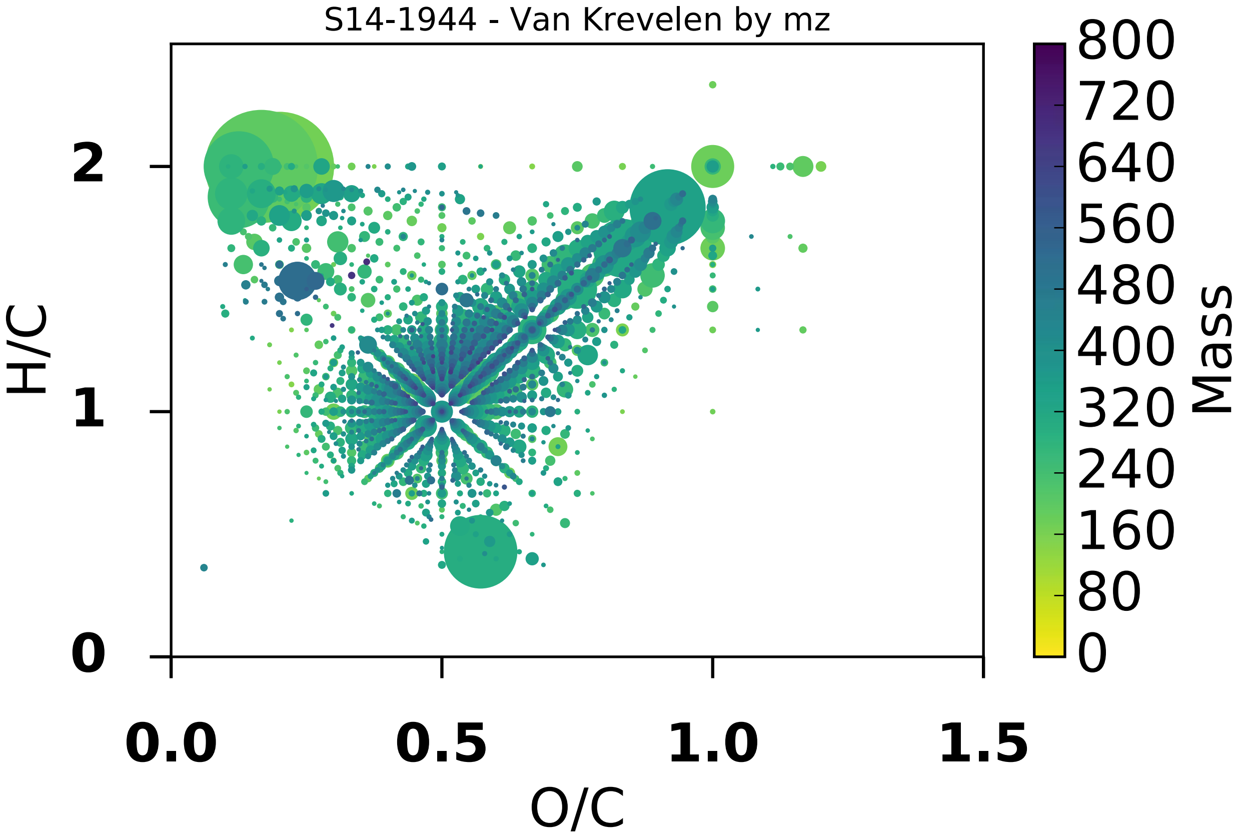

S14-1944 - Van Krevelen by mz
H/C
O/C
0
1
2
0.0
0.5
1.0
1.5
Mass
800
720
640
560
480
400
320
240
160
80
0

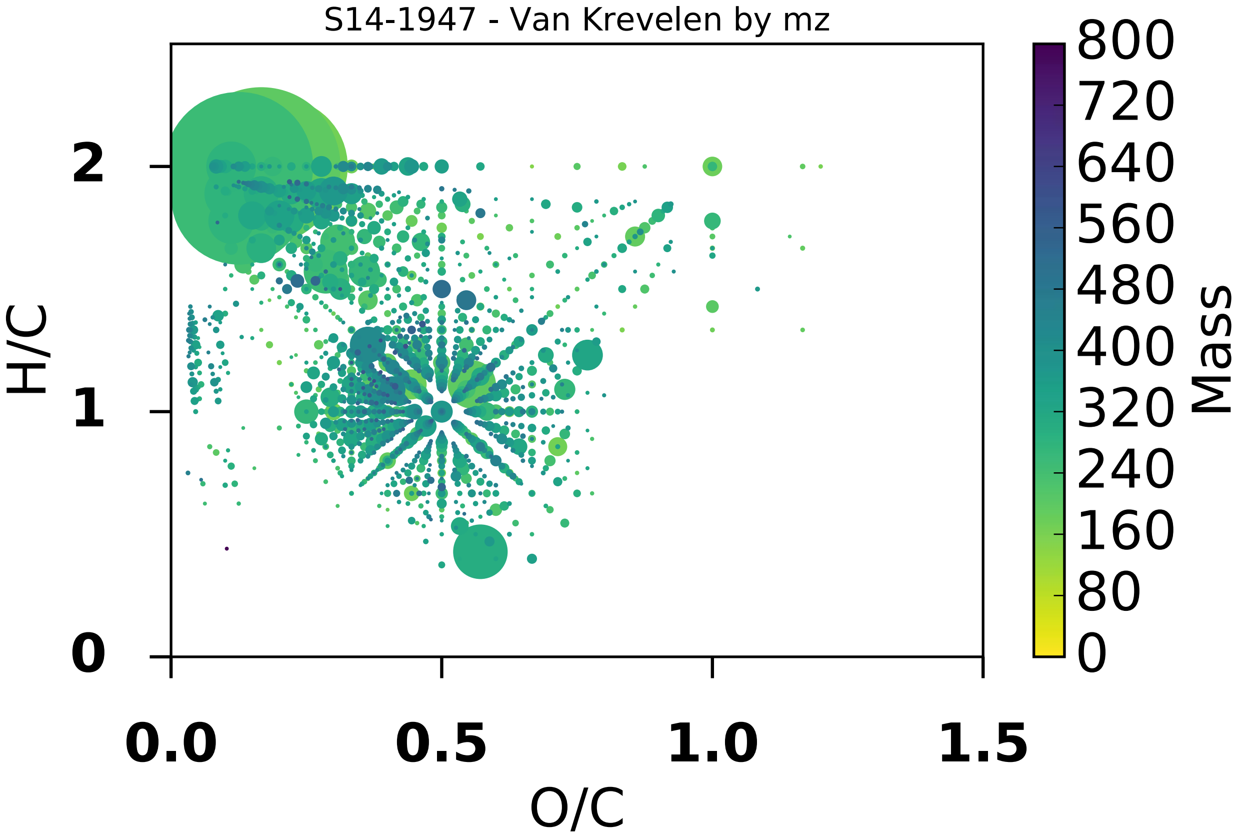

S14-1947 - Van Krevelen by mz
H/C
O/C
0
1
2
0.0
0.5
1.0
1.5
Mass
800
720
640
560
480
400
320
240
160
80
0

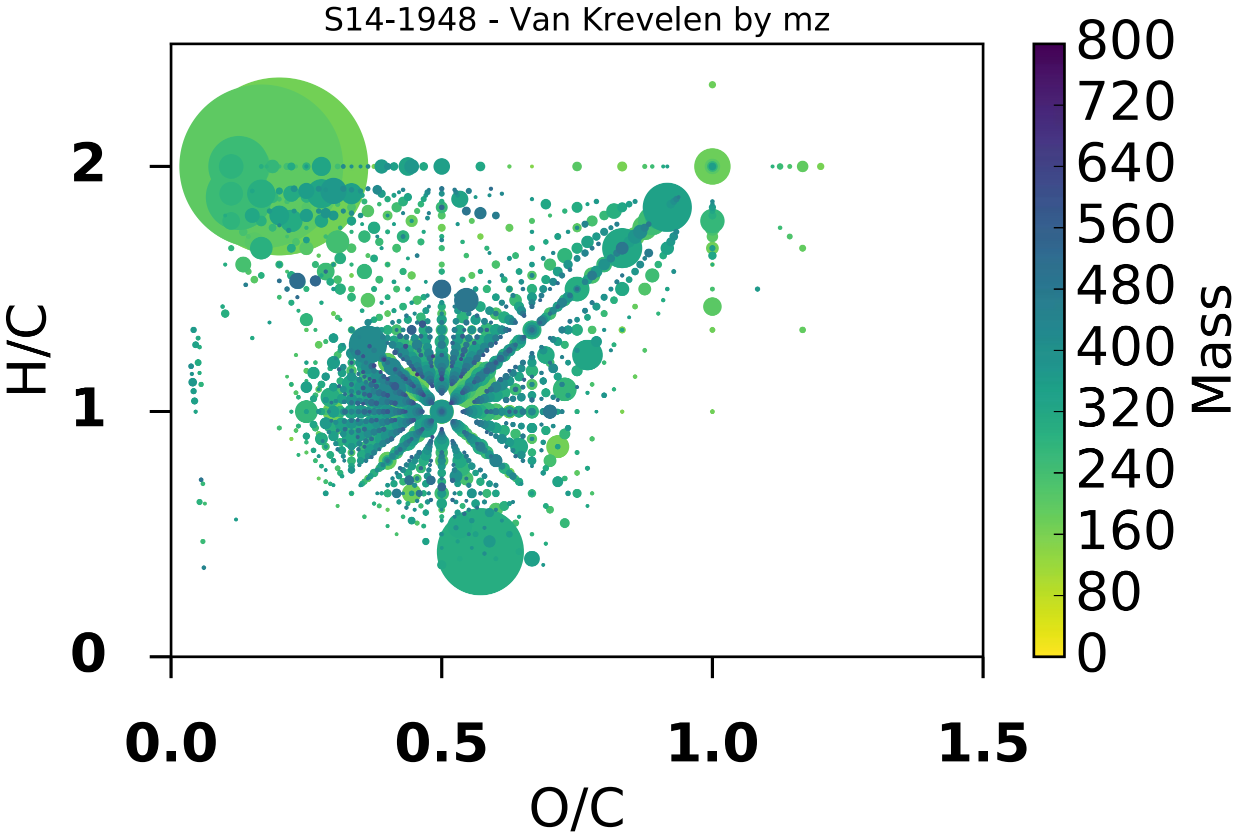

S14-1948 - Van Krevelen by mz
H/C
O/C
0
1
2
0.0
0.5
1.0
1.5
Mass
0
80
160
240
320
400
480
560
640
720
800

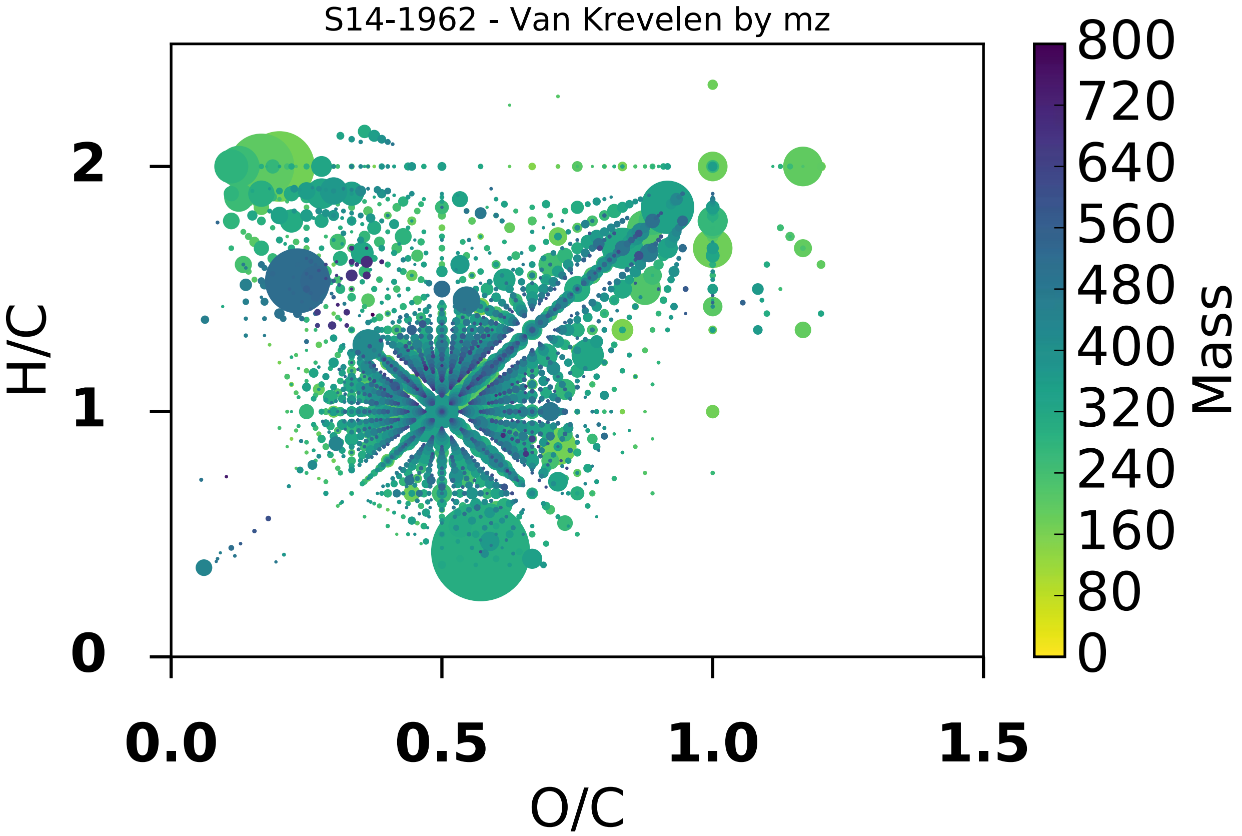

S14-1962 - Van Krevelen by mz
H/C
O/C
Mass
0
1
2
0.0
0.5
1.0
1.5
800
720
640
560
480
400
320
240
160
80
0

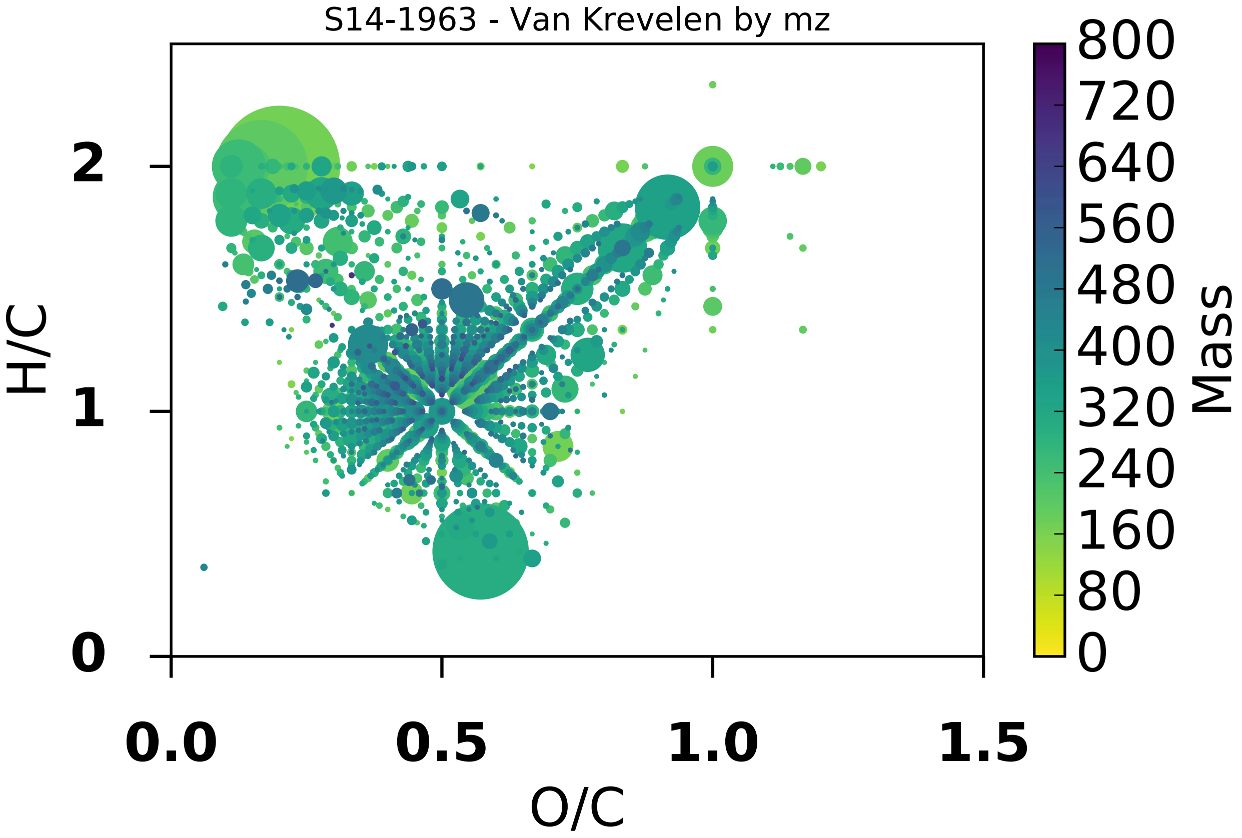
S14-1963 - Van Krevelen by mz
H/C
2
1
0
0.0
0.5
1.0
1.5
O/C
800
720
640
560
480
400
320
240
160
80
0
Mass

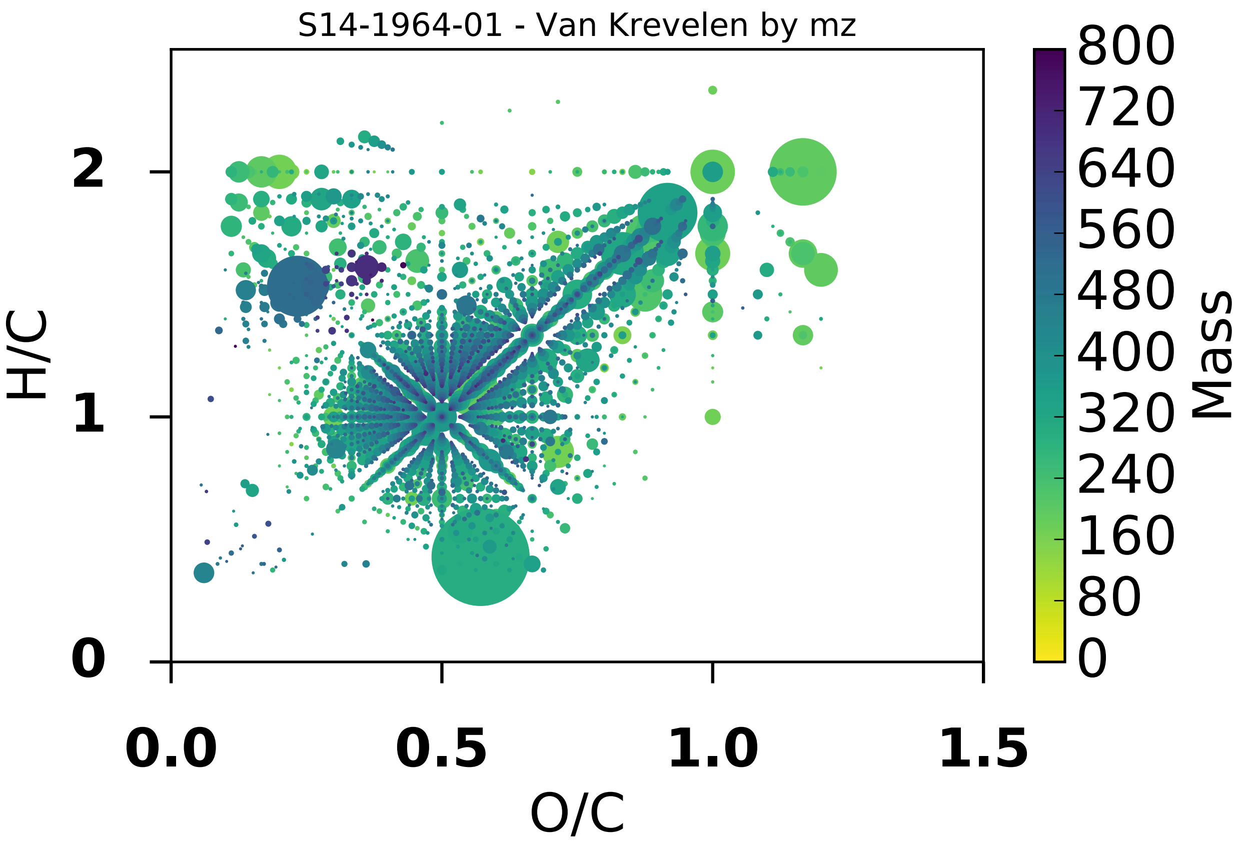

S14-1964-01 - Van Krevelen by mz
H/C
2
1
0
0.0
0.5
1.0
1.5
O/C
Mass
800
720
640
560
480
400
320
240
160
80
0

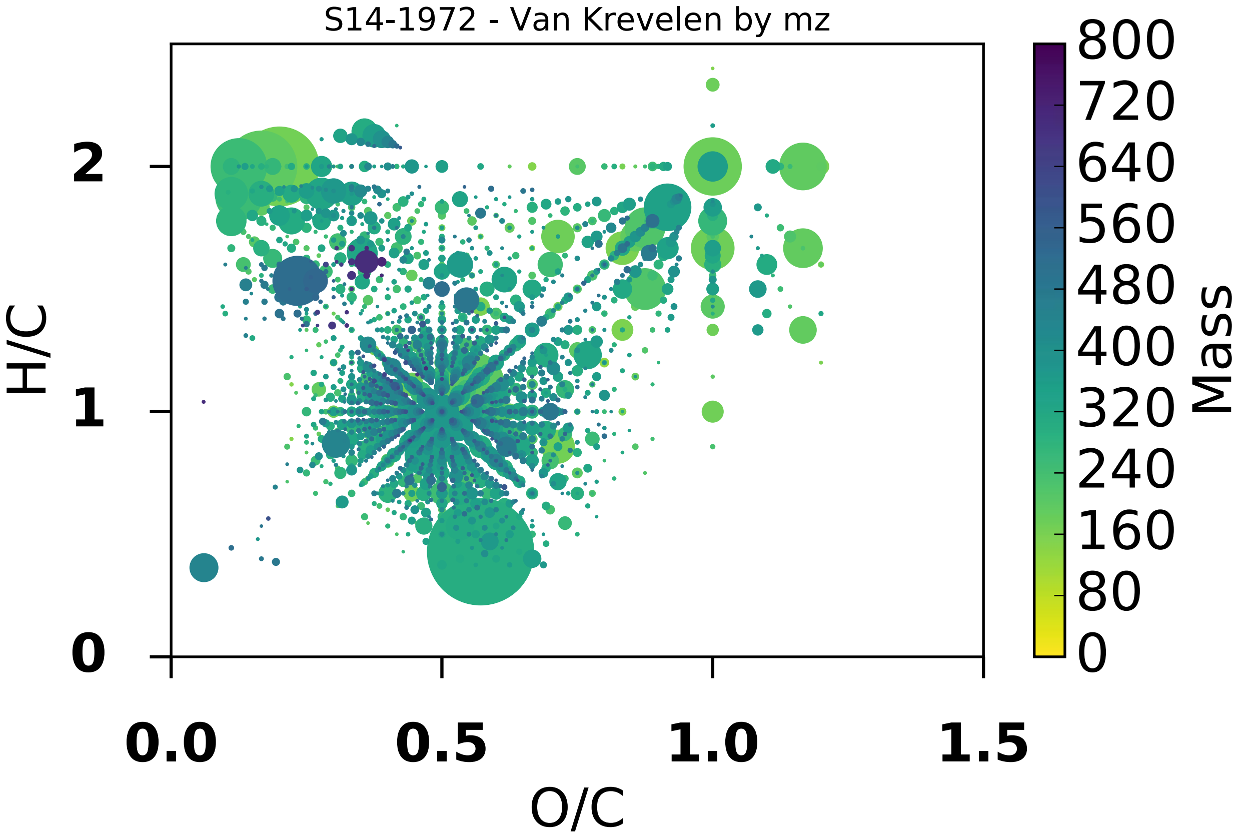

S14-1972 - Van Krevelen by mz
H/C
O/C
0
1
2
0.0
0.5
1.0
1.5
Mass
0
80
160
240
320
400
480
560
640
720
800

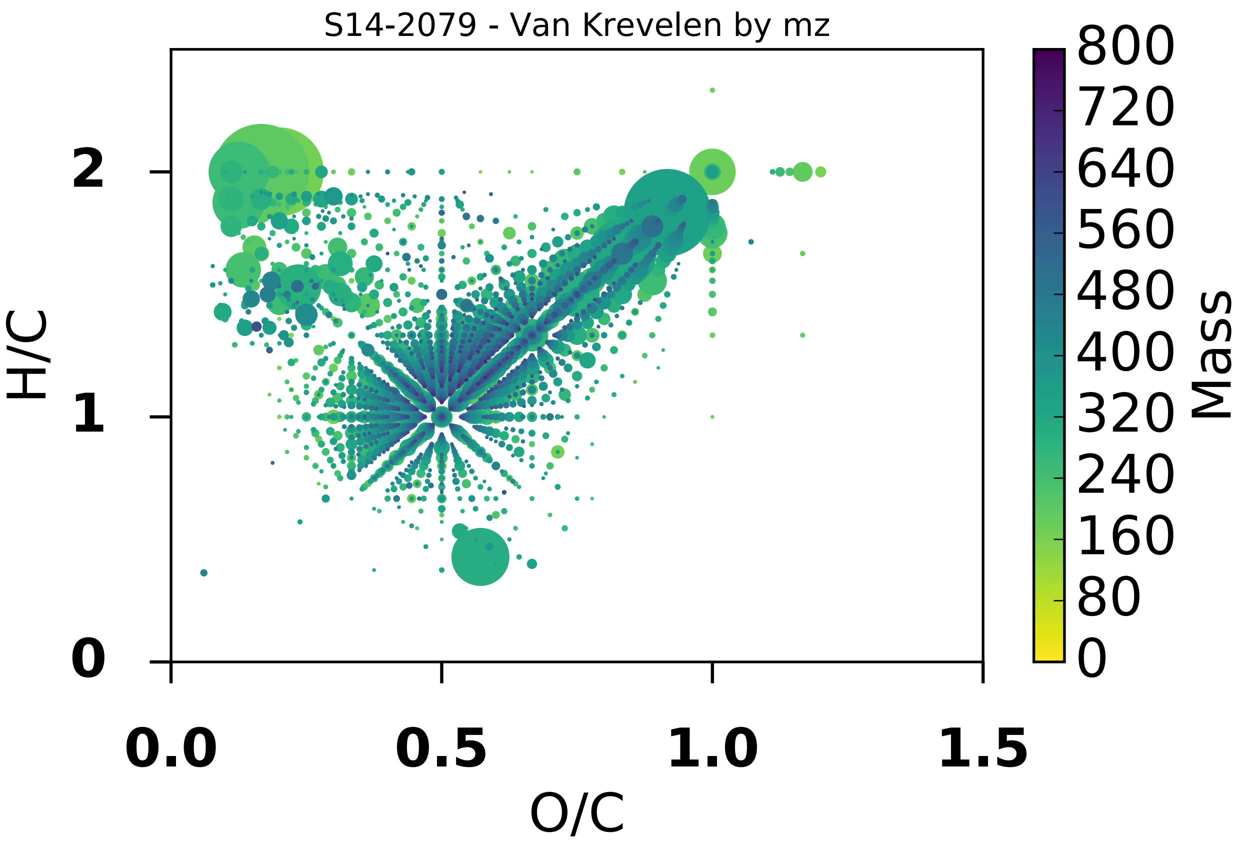
S14-2079 - Van Krevelen by mz
H/C
2
1
0
0.0
0.5
1.0
1.5
O/C
Mass
800
720
640
560
480
400
320
240
160
80
0

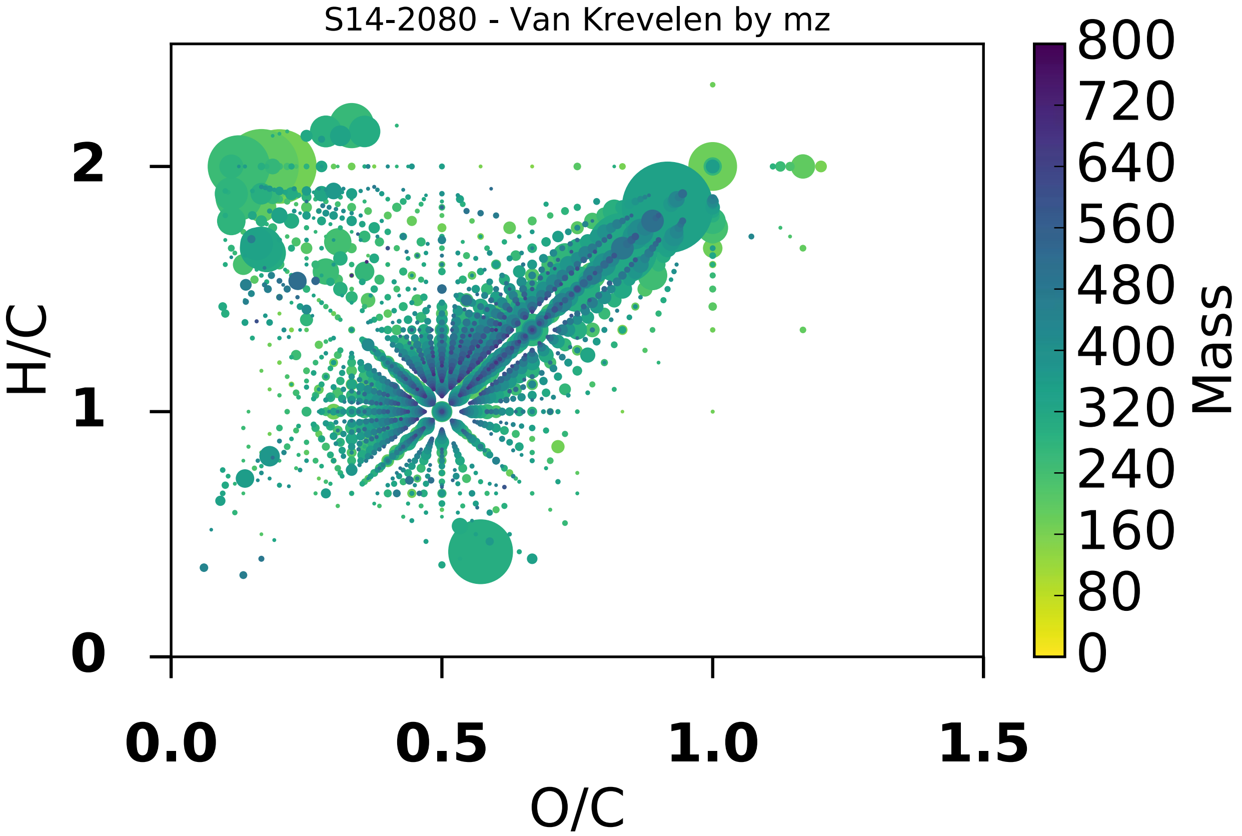

S14-2080 - Van Krevelen by mz
H/C
O/C
0
1
2
0.0
0.5
1.0
1.5
Mass
800
720
640
560
480
400
320
240
160
80
0

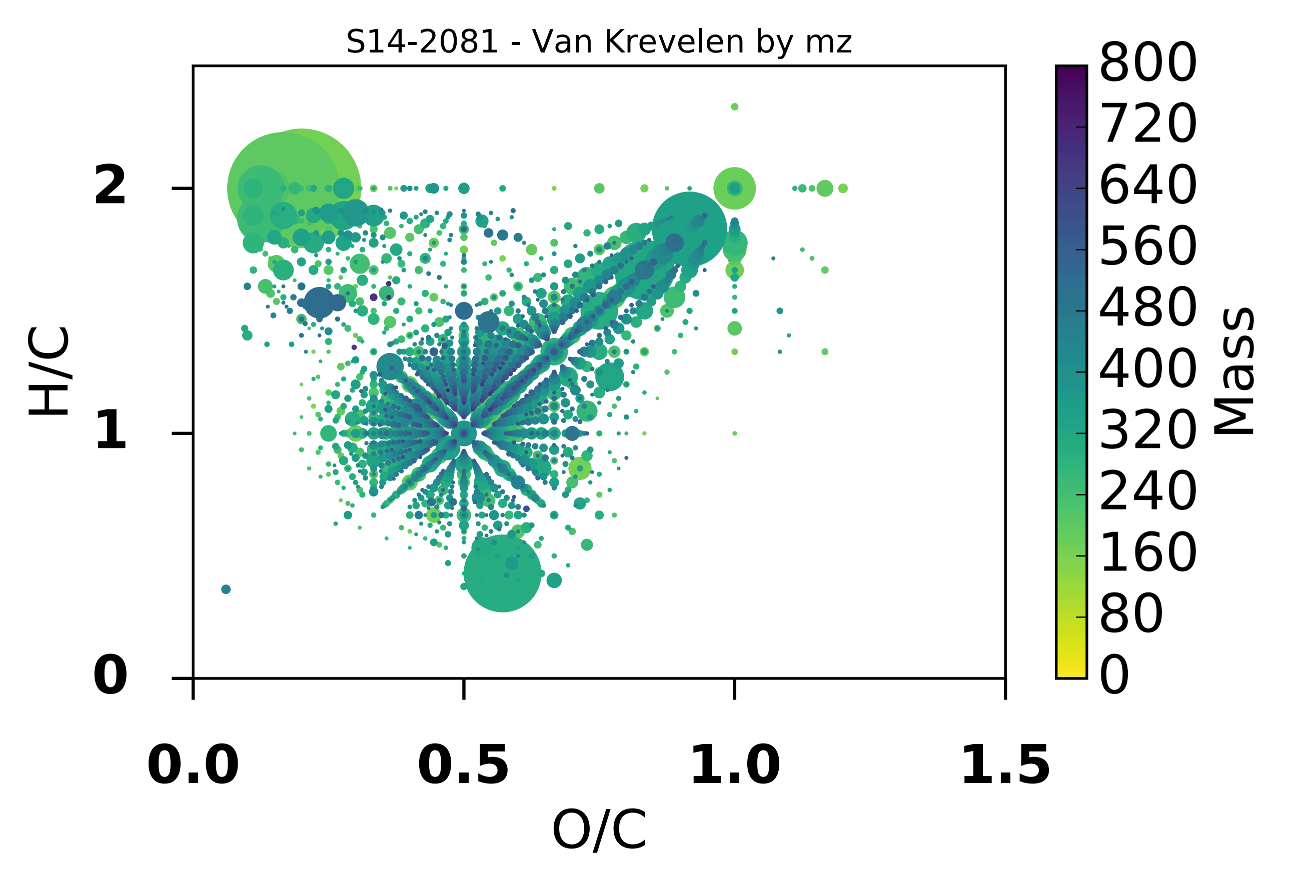

S14-2081 - Van Krevelen by mz
H/C
O/C
0
1
2
0.0
0.5
1.0
1.5
Mass
800
720
640
560
480
400
320
240
160
80
0

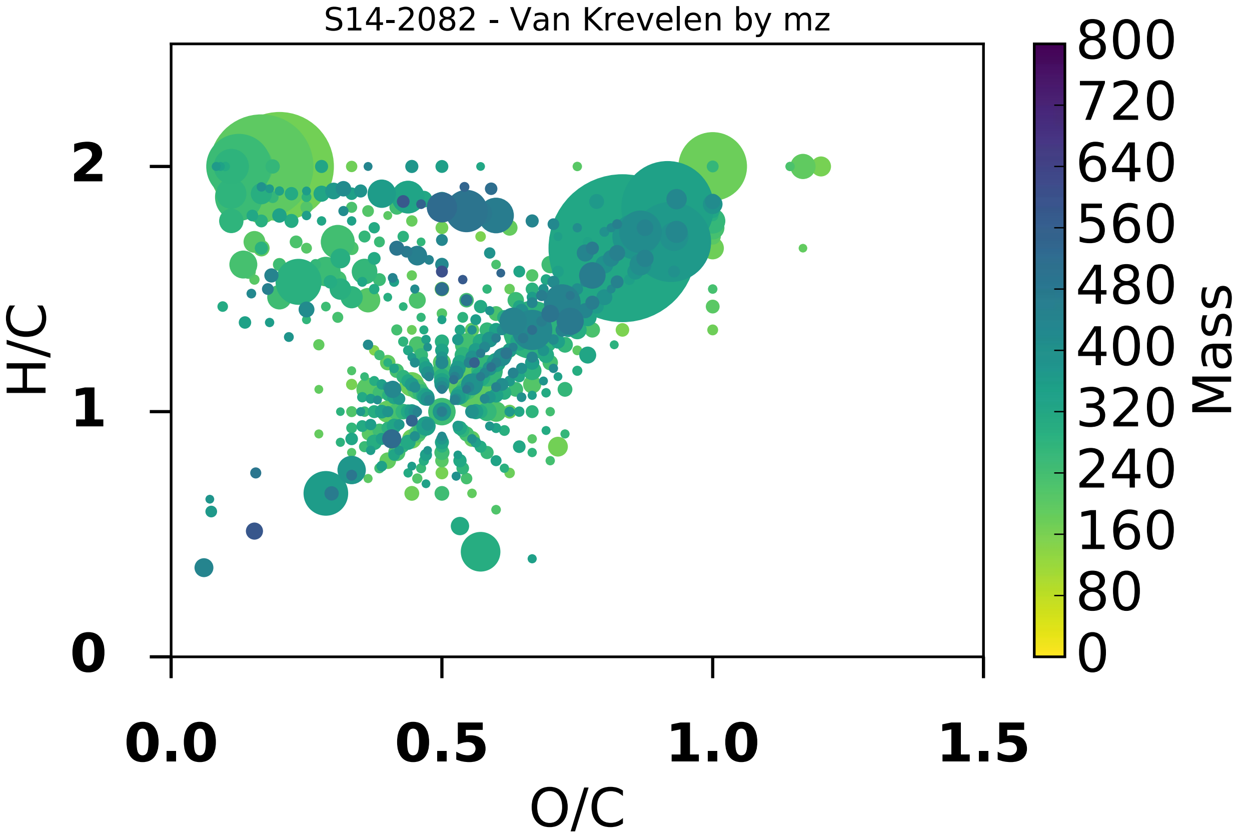

S14-2082 - Van Krevelen by mz
H/C
0
1
2
0.0
0.5
1.0
1.5
O/C
Mass
0
80
160
240
320
400
480
560
640
720
800

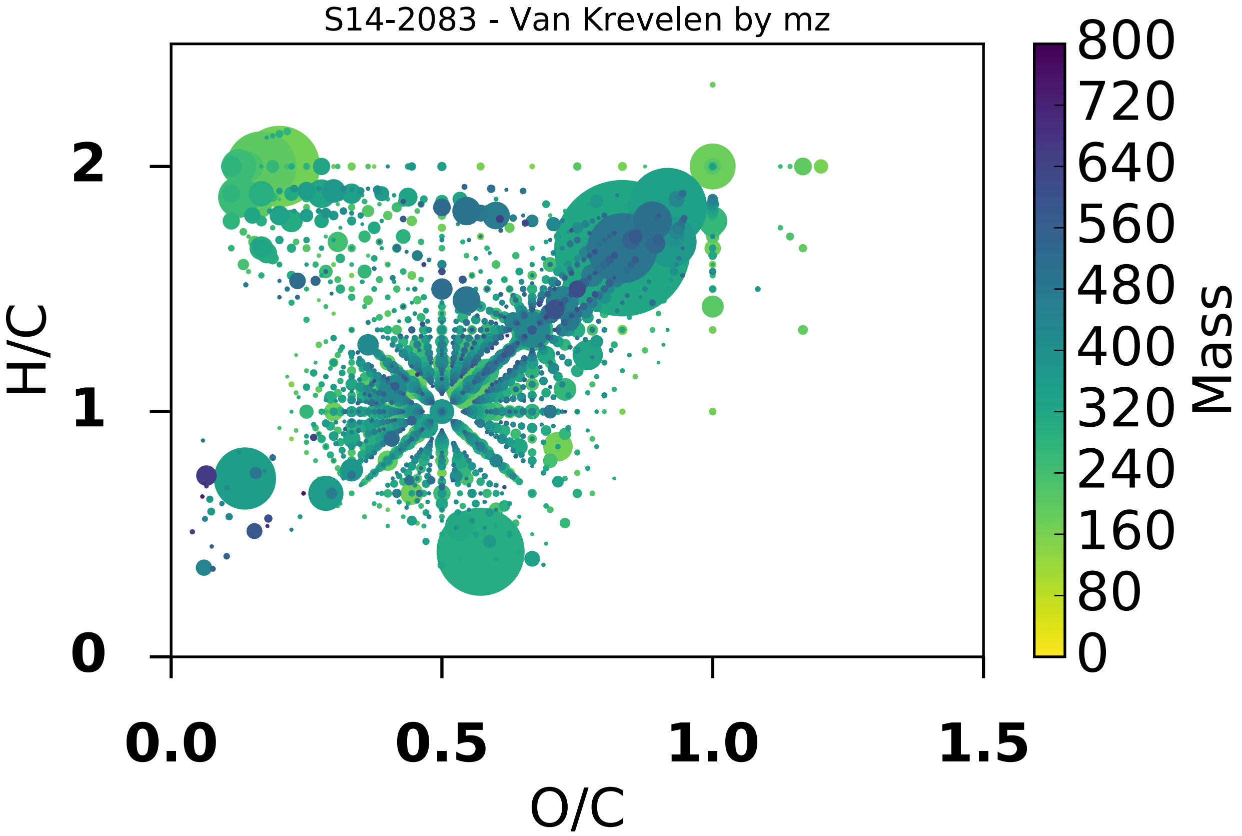

S14-2083 - Van Krevelen by mz
H/C
O/C
0
1
2
0.0
0.5
1.0
1.5
Mass
0
80
160
240
320
400
480
560
640
720
800

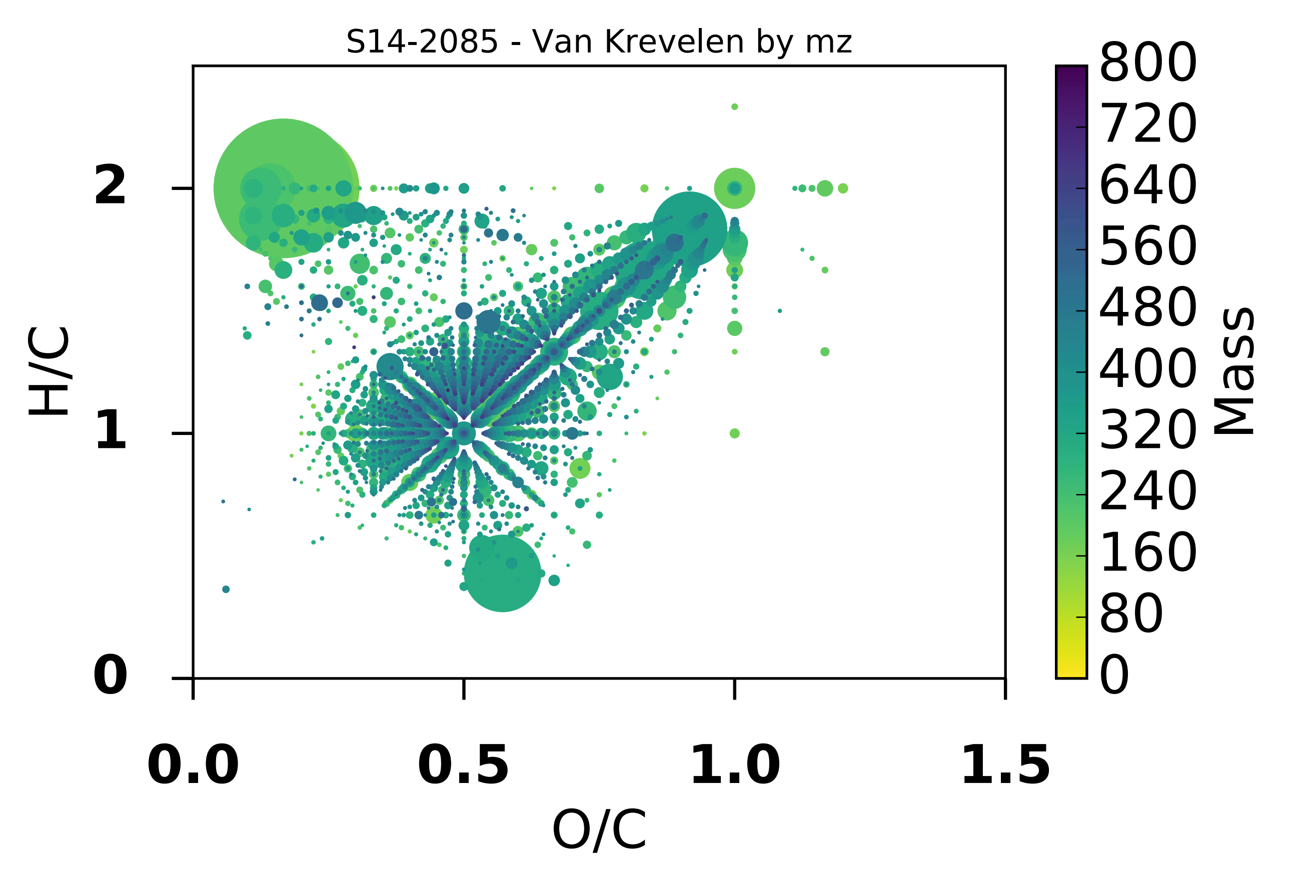

S14-2085 - Van Krevelen by mz
H/C
2
1
0
0.0
0.5
1.0
1.5
O/C
800
720
640
560
480
400
320
240
160
80
0
Mass

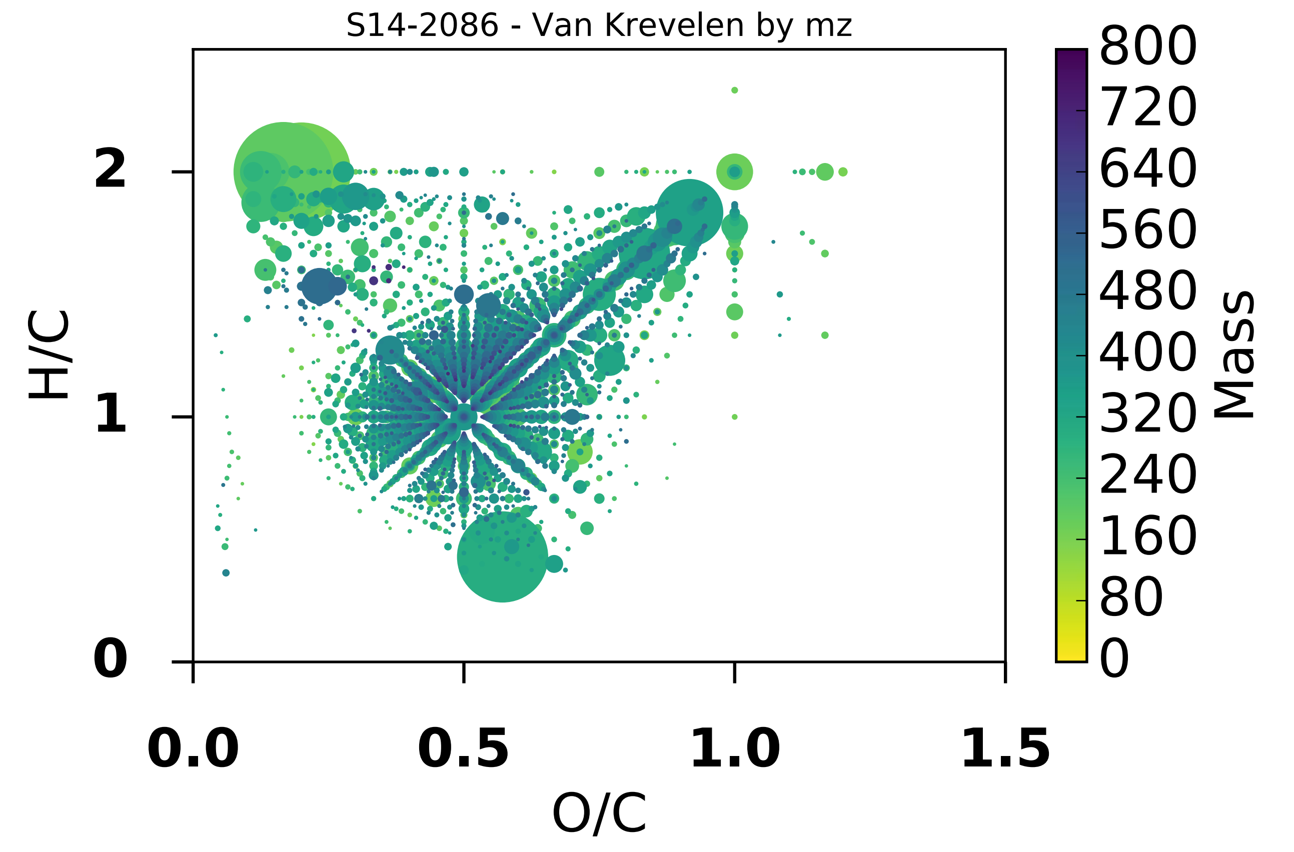
S14-2086 - Van Krevelen by mz
H/C
O/C
2
1
0
0.0
0.5
1.0
1.5
800
720
640
560
480
400
320
240
160
80
0
Mass

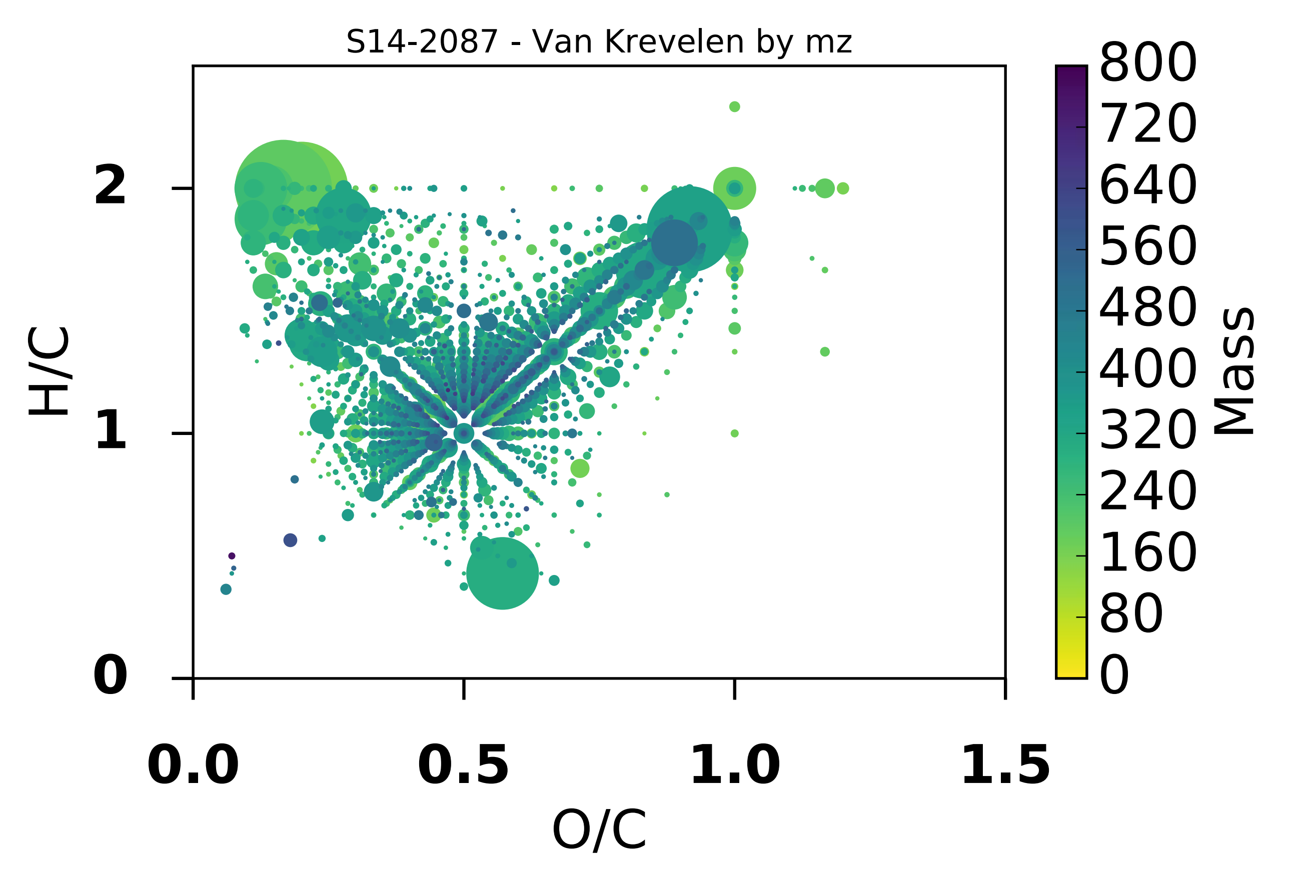
S14-2087 - Van Krevelen by mz
H/C
O/C
0
1
2
0.0
0.5
1.0
1.5
Mass
800
720
640
560
480
400
320
240
160
80
0

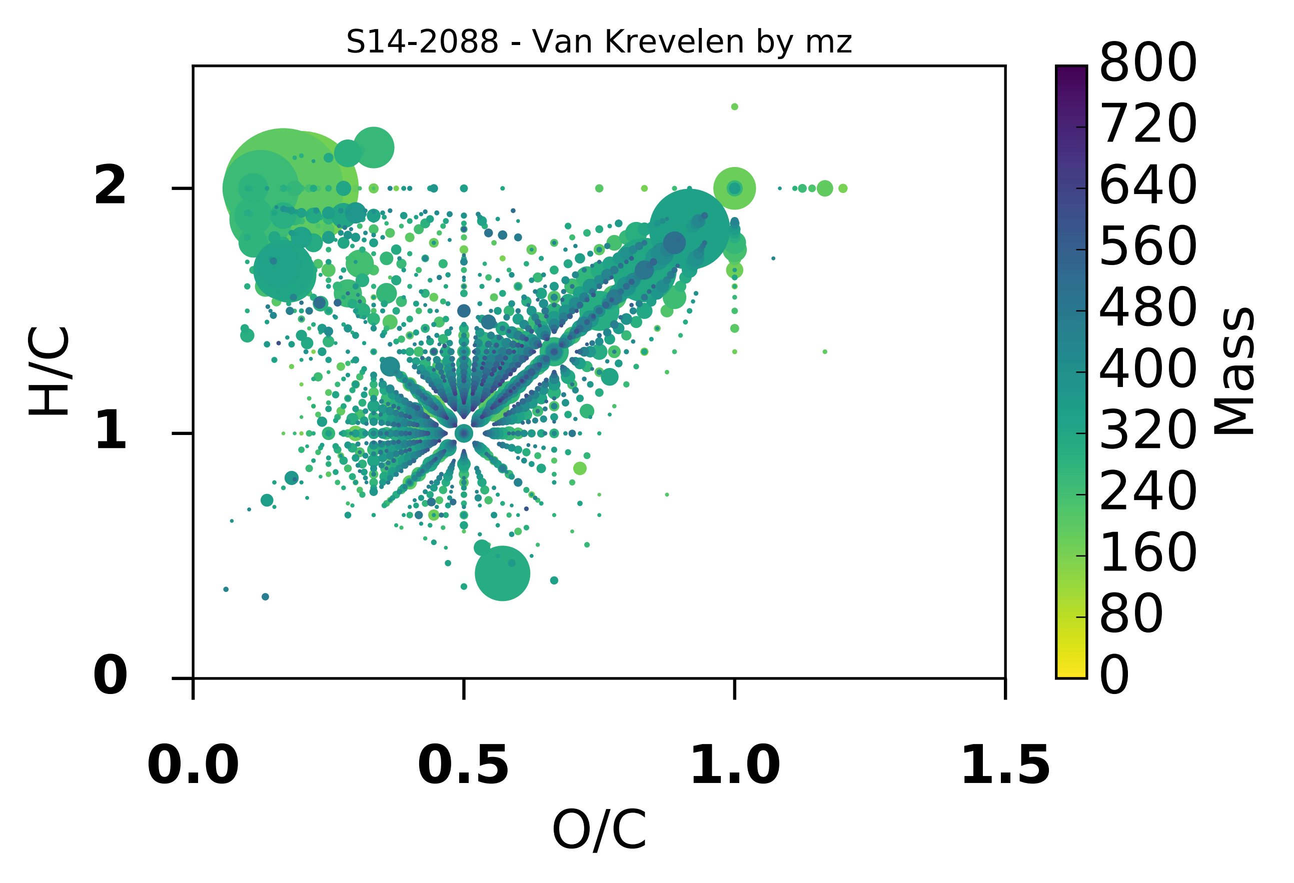

S14-2088 - Van Krevelen by mz
H/C
O/C
0
1
2
0.0
0.5
1.0
1.5
Mass
0
80
160
240
320
400
480
560
640
720
800

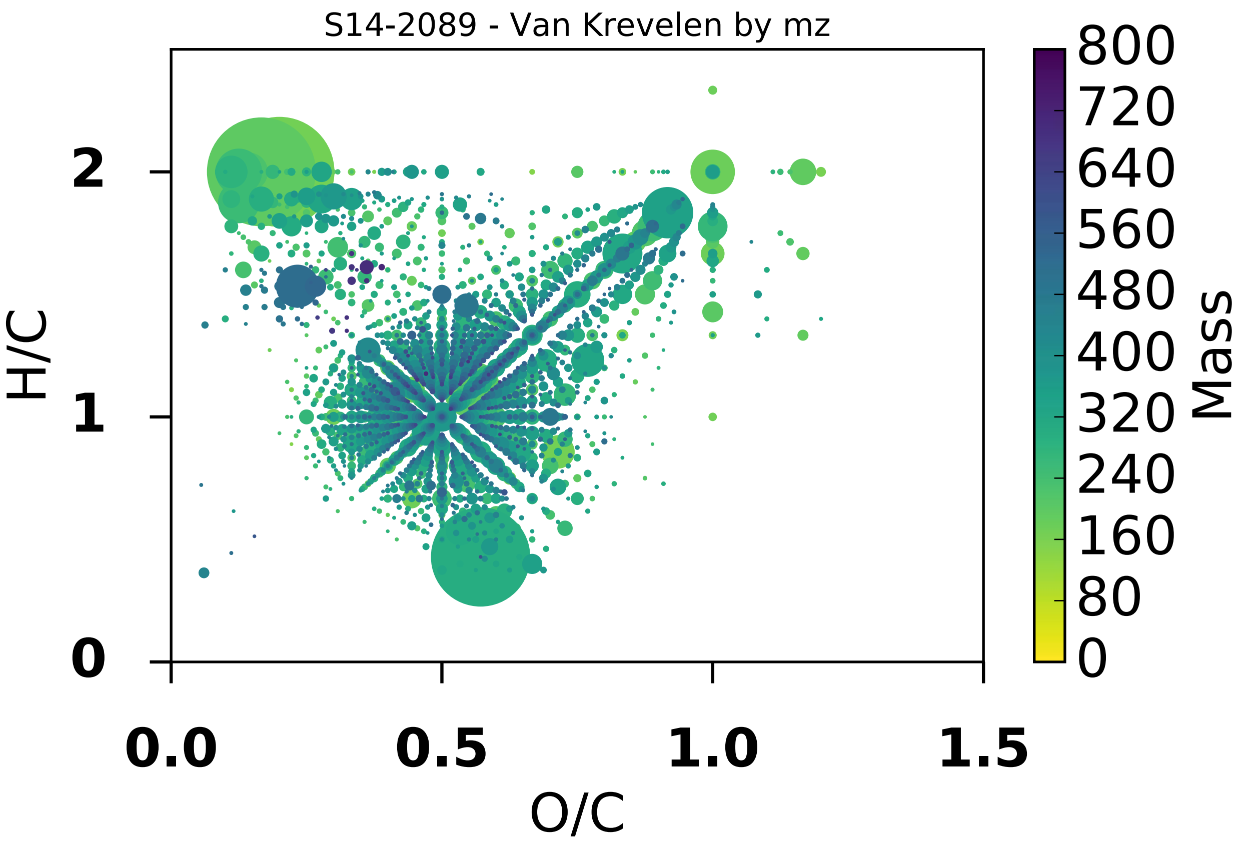

S14-2089 - Van Krevelen by mz
H/C
O/C
Mass
0
1
2
0.0
0.5
1.0
1.5
800
720
640
560
480
400
320
240
160
80
0

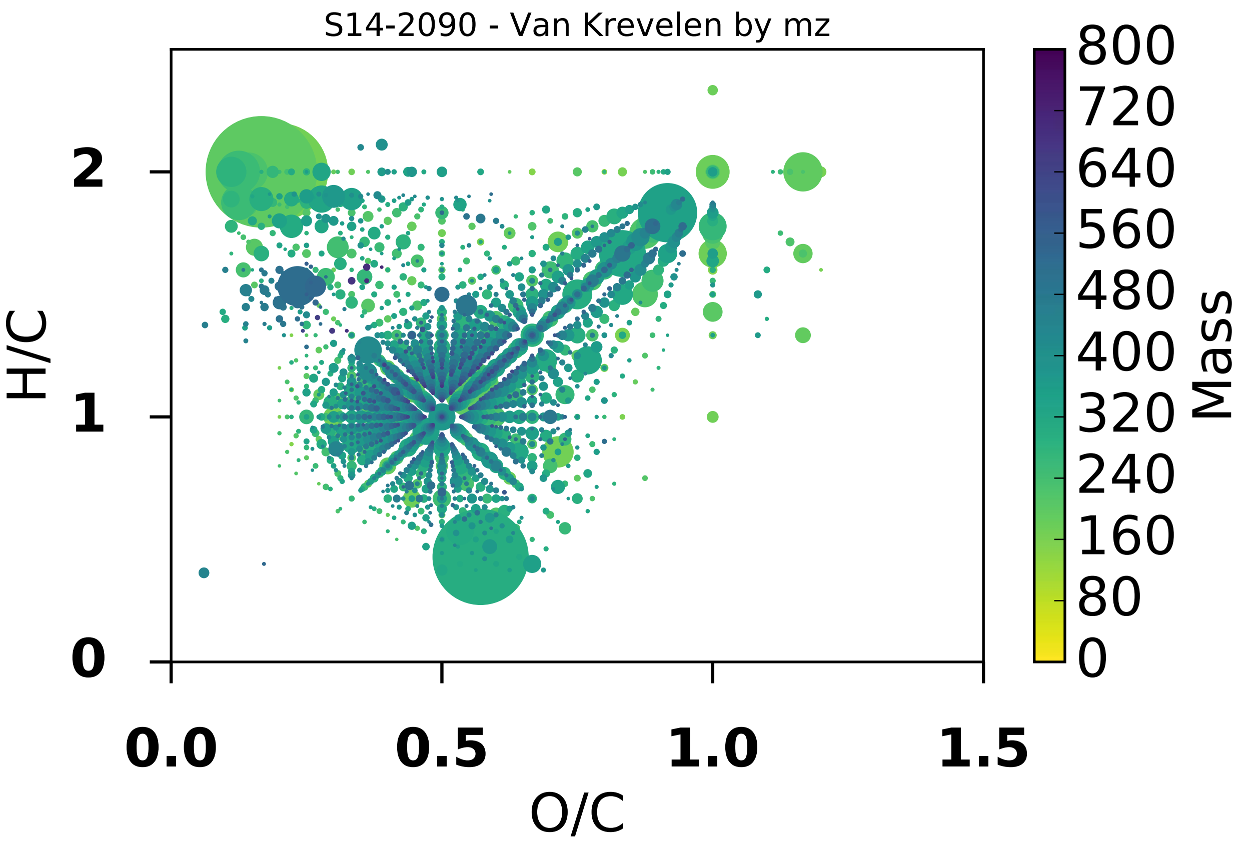

S14-2090 - Van Krevelen by mz
H/C
O/C
0
1
2
0.0
0.5
1.0
1.5
Mass
0
80
160
240
320
400
480
560
640
720
800

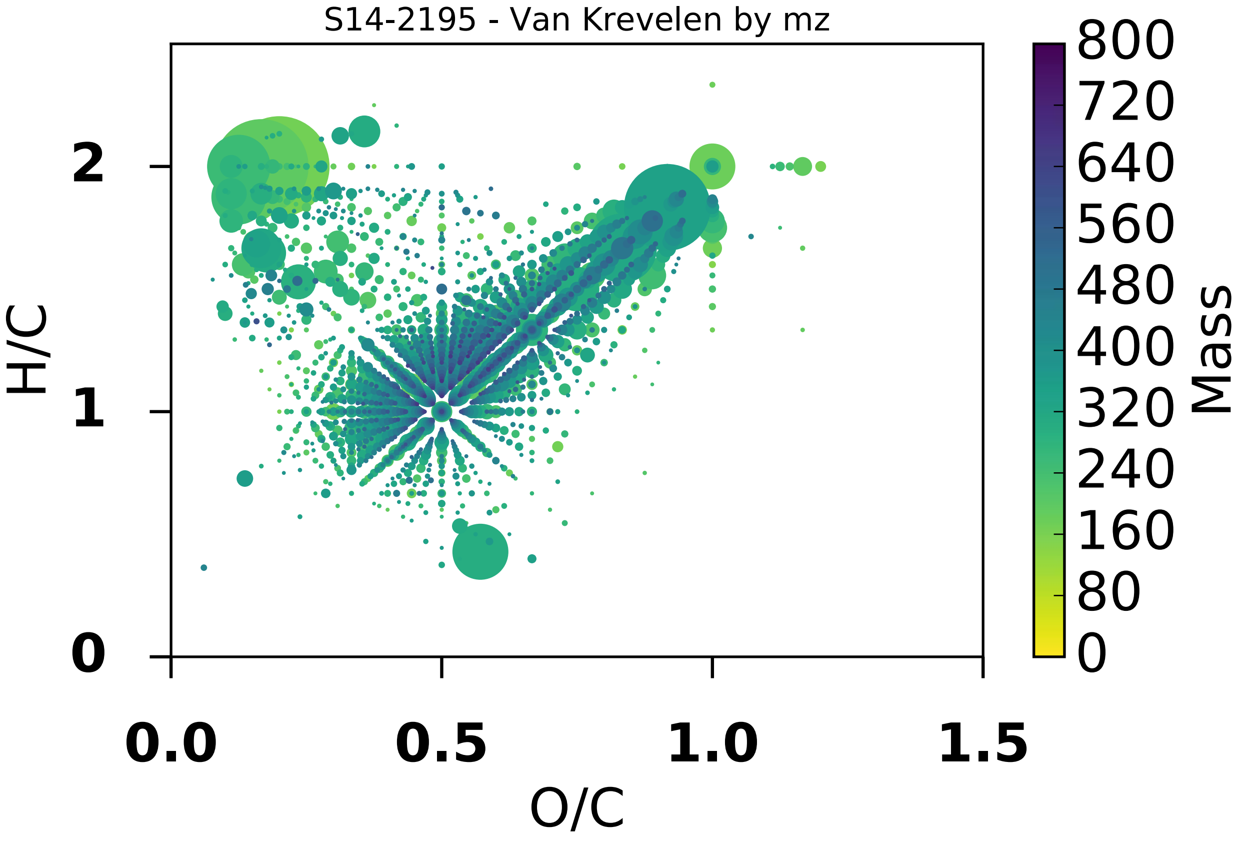

S14-2195 - Van Krevelen by mz
H/C
O/C
0.0
0.5
1.0
1.5
0
1
2
Mass
800
720
640
560
480
400
320
240
160
80
0

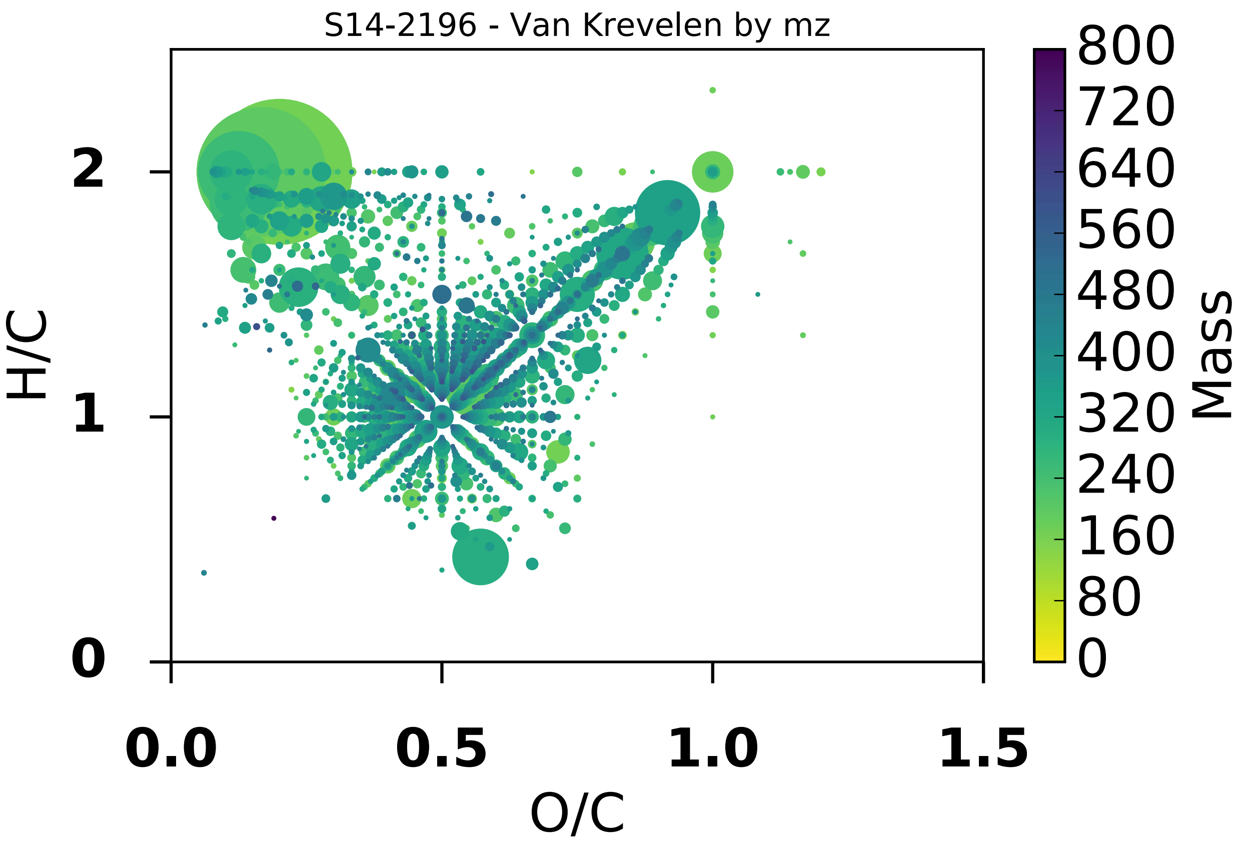

S14-2196 - Van Krevelen by mz
H/C
0
1
2
O/C
0.0
0.5
1.0
1.5
Mass
0
80
160
240
320
400
480
560
640
720
800

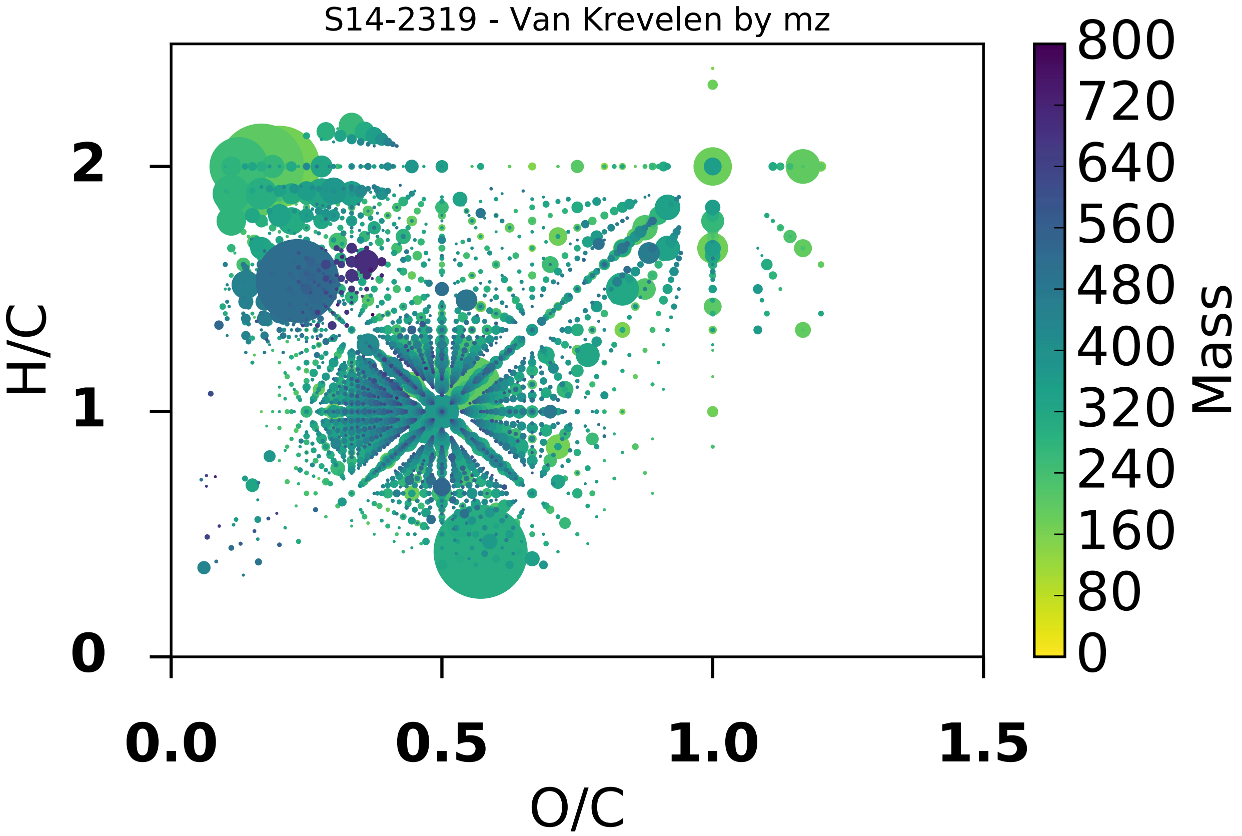
S14-2319 - Van Krevelen by mz
H/C
O/C
0
1
2
0.0
0.5
1.0
1.5
Mass
800
720
640
560
480
400
320
240
160
80
0

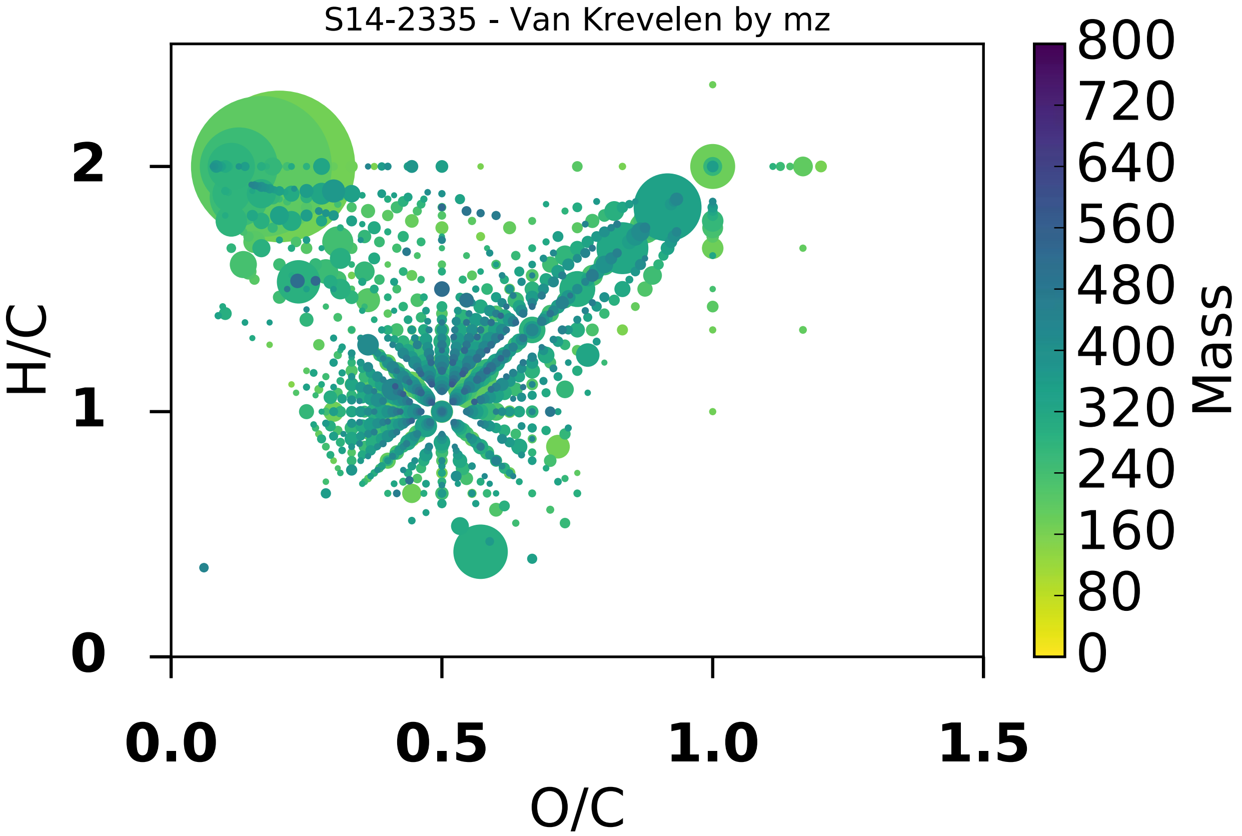

S14-2335 - Van Krevelen by mz
H/C
O/C
Mass
0
1
2
0.0
0.5
1.0
1.5
800
720
640
560
480
400
320
240
160
80
0

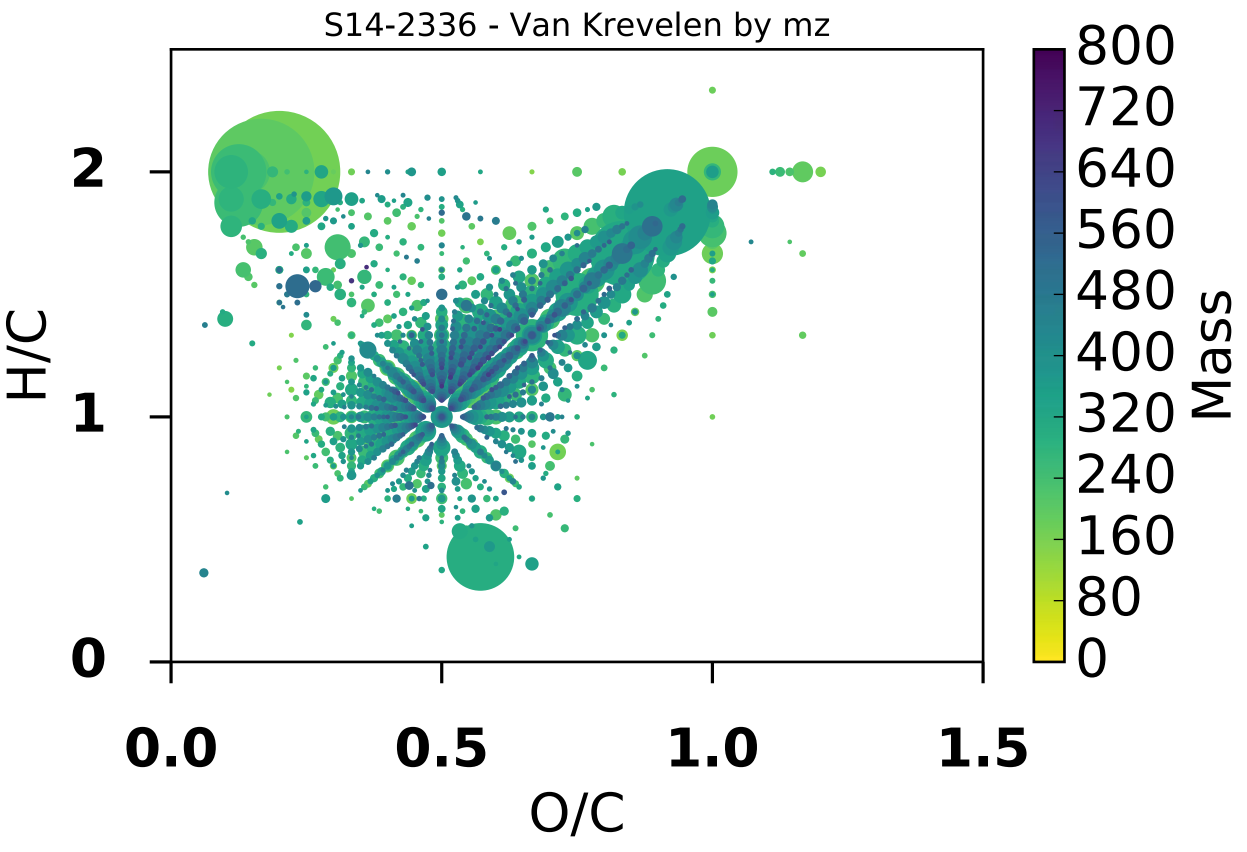
S14-2336 - Van Krevelen by mz
H/C
O/C
0
1
2
0.0
0.5
1.0
1.5
Mass
800
720
640
560
480
400
320
240
160
80
0

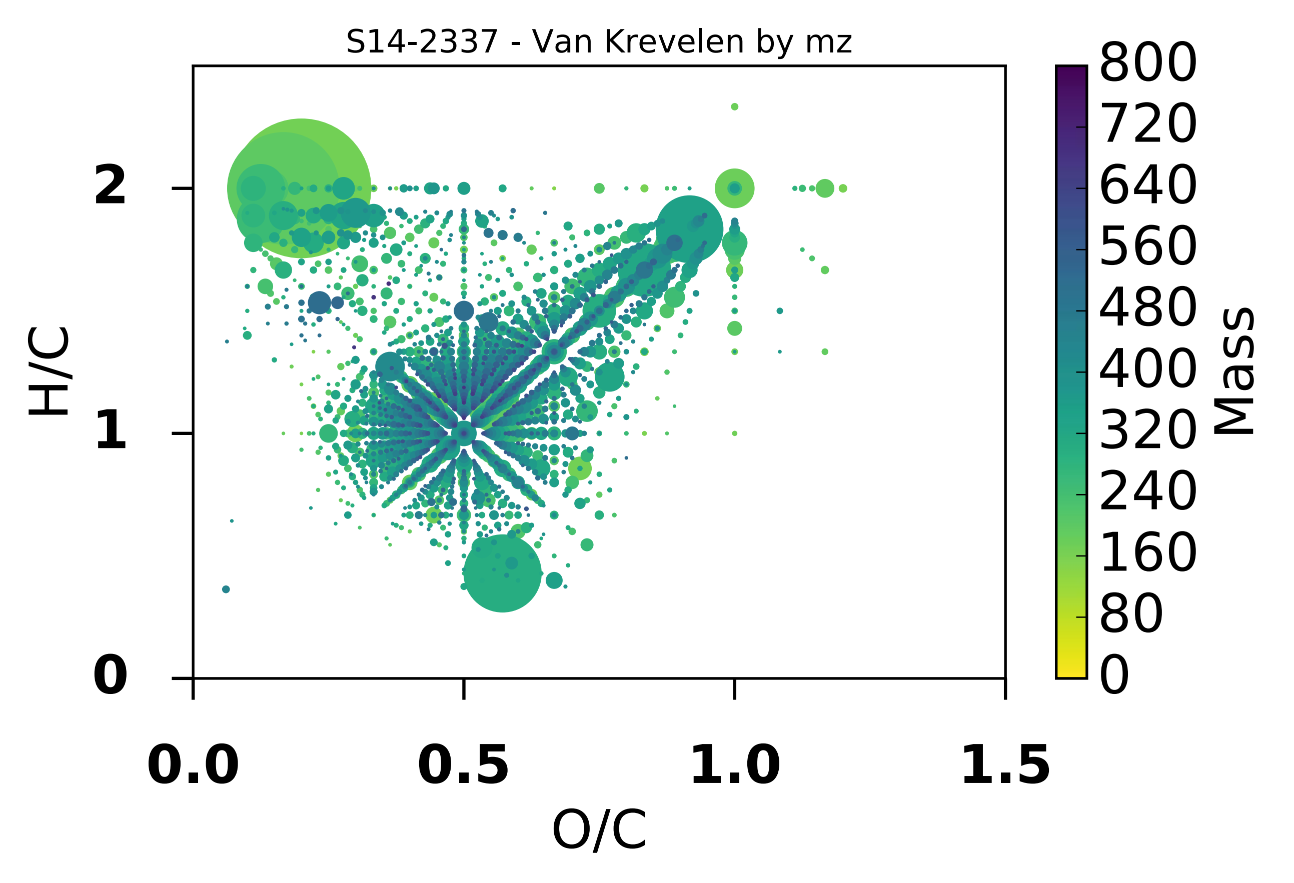

S14-2337 - Van Krevelen by mz
H/C
2
1
0
0.0
0.5
1.0
1.5
O/C
800
720
640
560
480
400
320
240
160
80
0
Mass

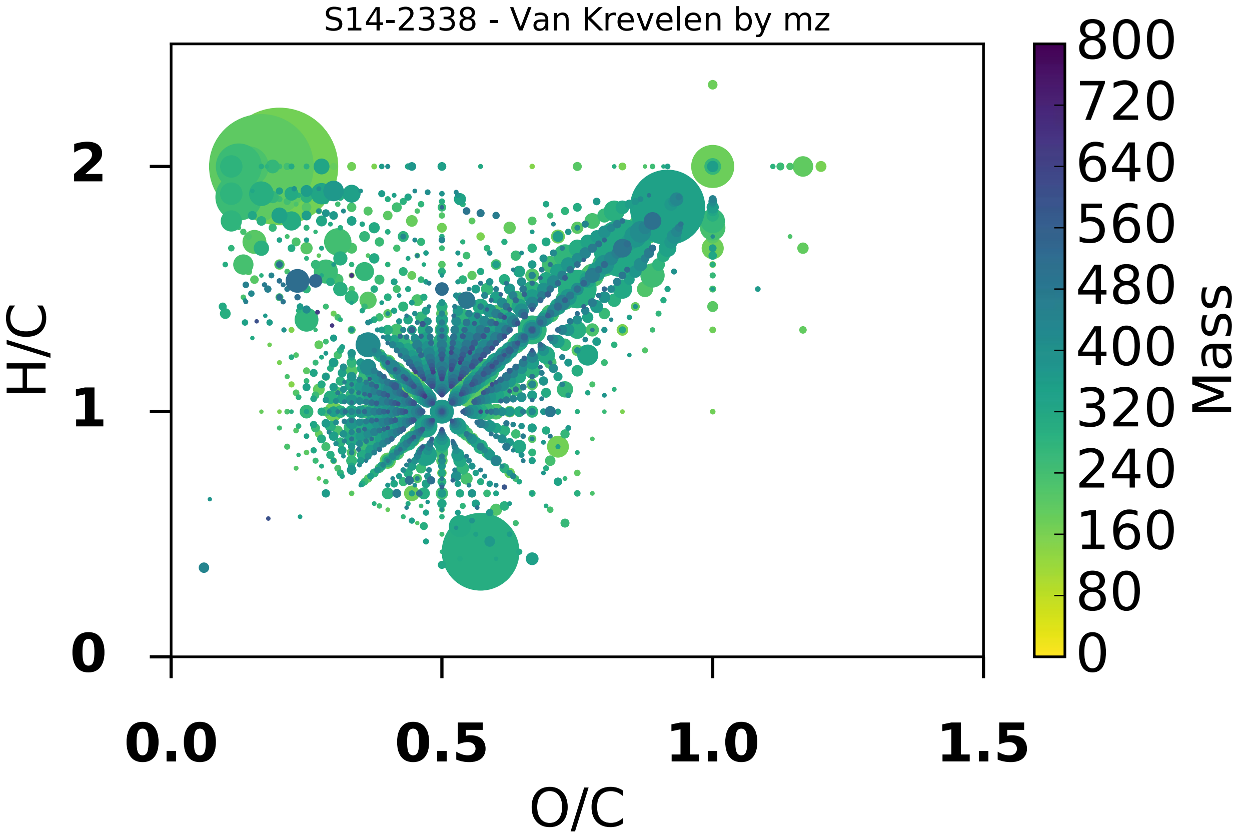

S14-2338 - Van Krevelen by mz
H/C
O/C
0.0
0.5
1.0
1.5
0
1
2
Mass
800
720
640
560
480
400
320
240
160
80
0

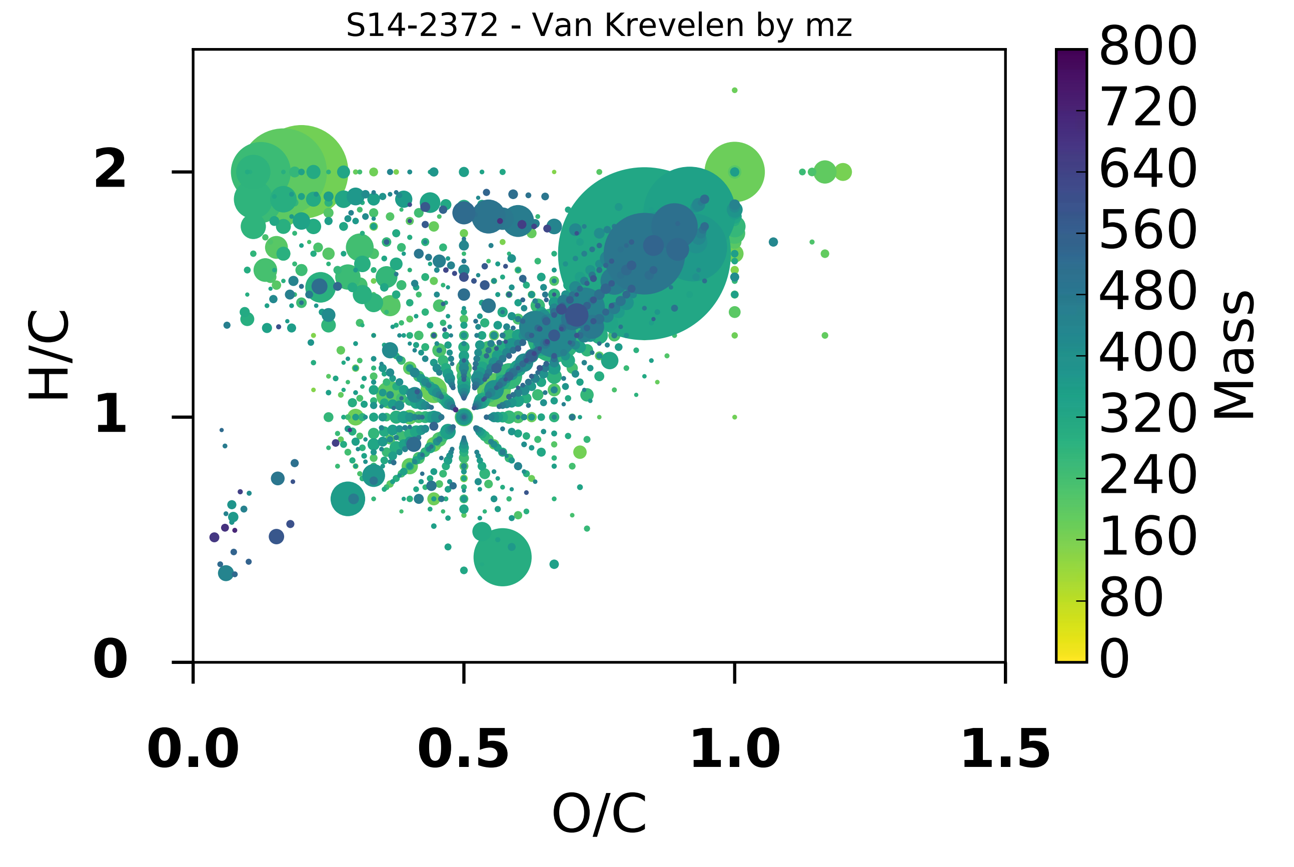

S14-2372 - Van Krevelen by mz
H/C
O/C
0
1
2
0.0
0.5
1.0
1.5
Mass
0
80
160
240
320
400
480
560
640
720
800

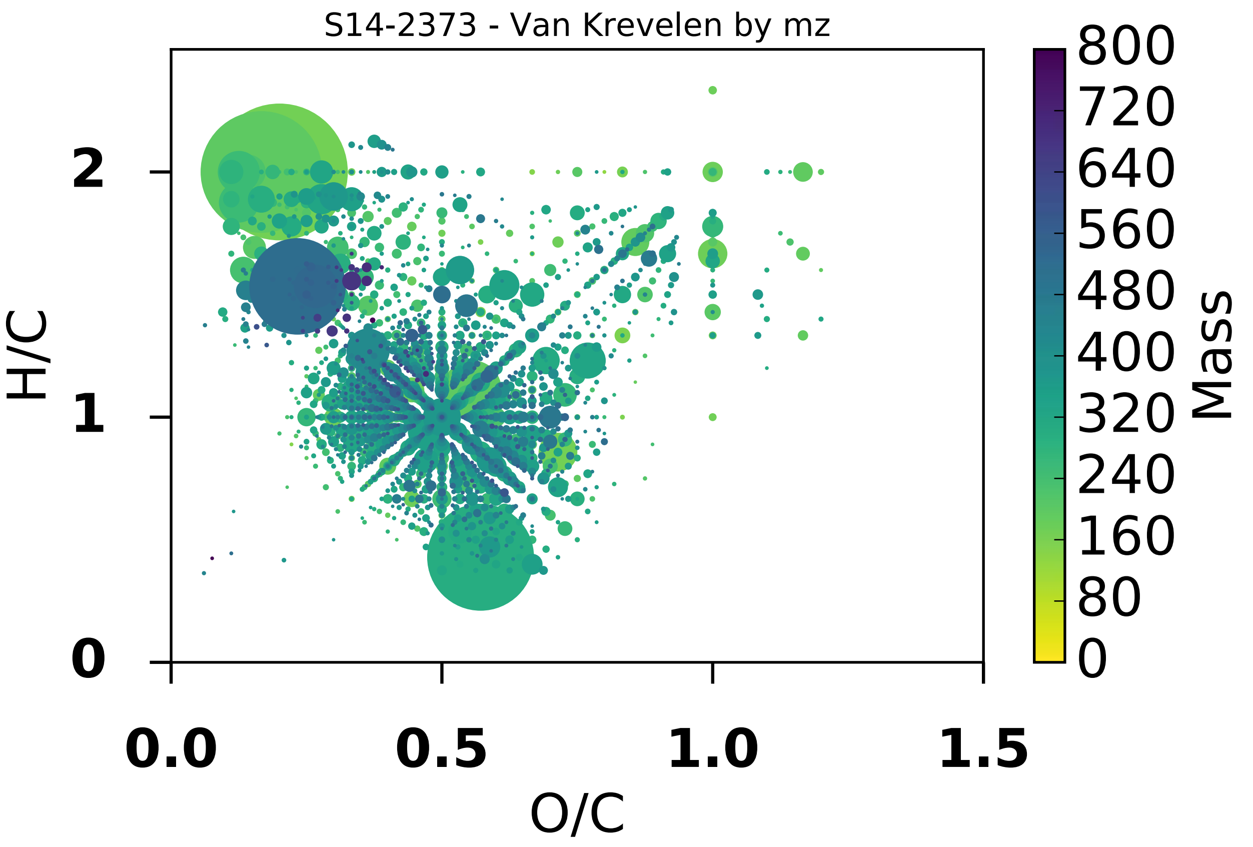
S14-2373 - Van Krevelen by mz
H/C
2
1
0
0.0
0.5
1.0
1.5
O/C
800
720
640
560
480
400
320
240
160
80
0
Mass

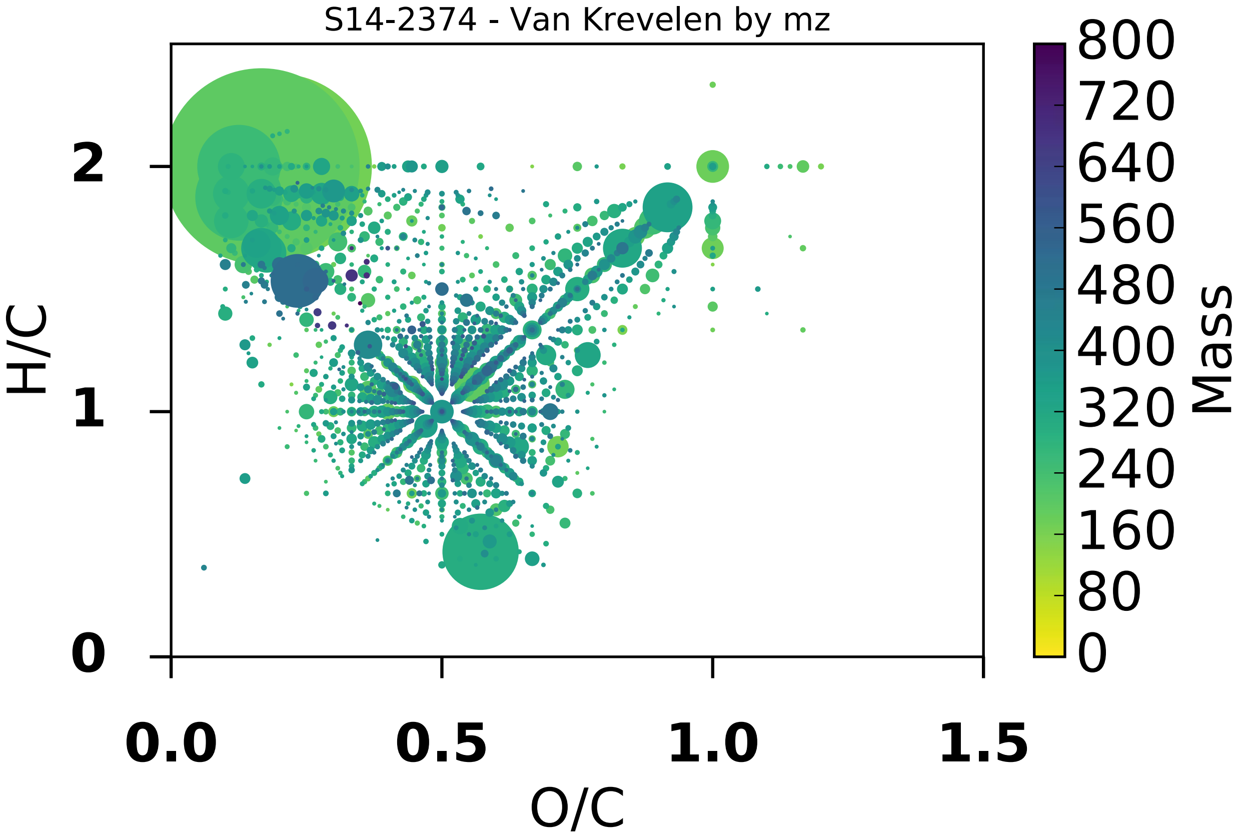
S14-2374 - Van Krevelen by mz
H/C
O/C
0
1
2
0.0
0.5
1.0
1.5
Mass
800
720
640
560
480
400
320
240
160
80
0

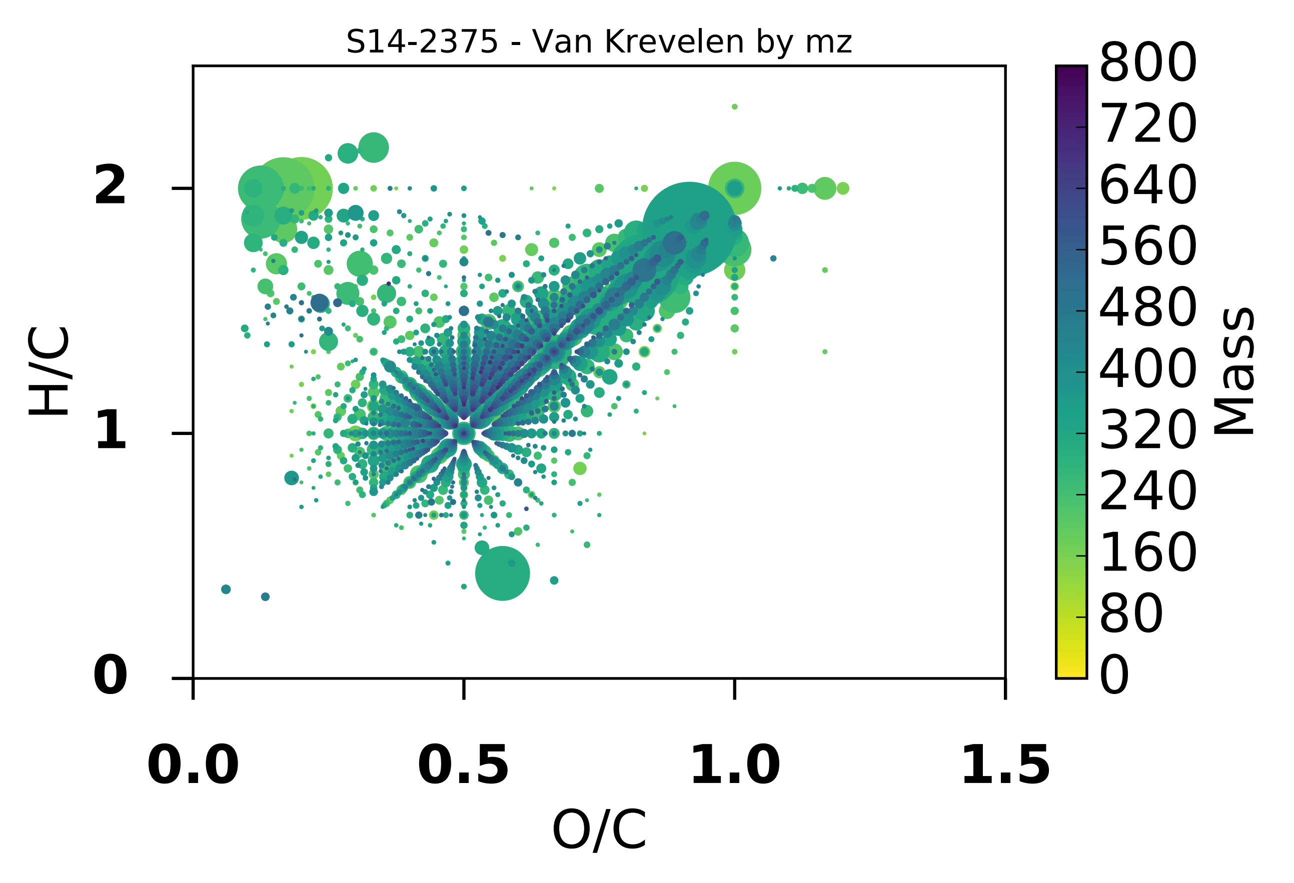

S14-2375 - Van Krevelen by mz
H/C
O/C
0
1
2
0.0
0.5
1.0
1.5
Mass
0
80
160
240
320
400
480
560
640
720
800

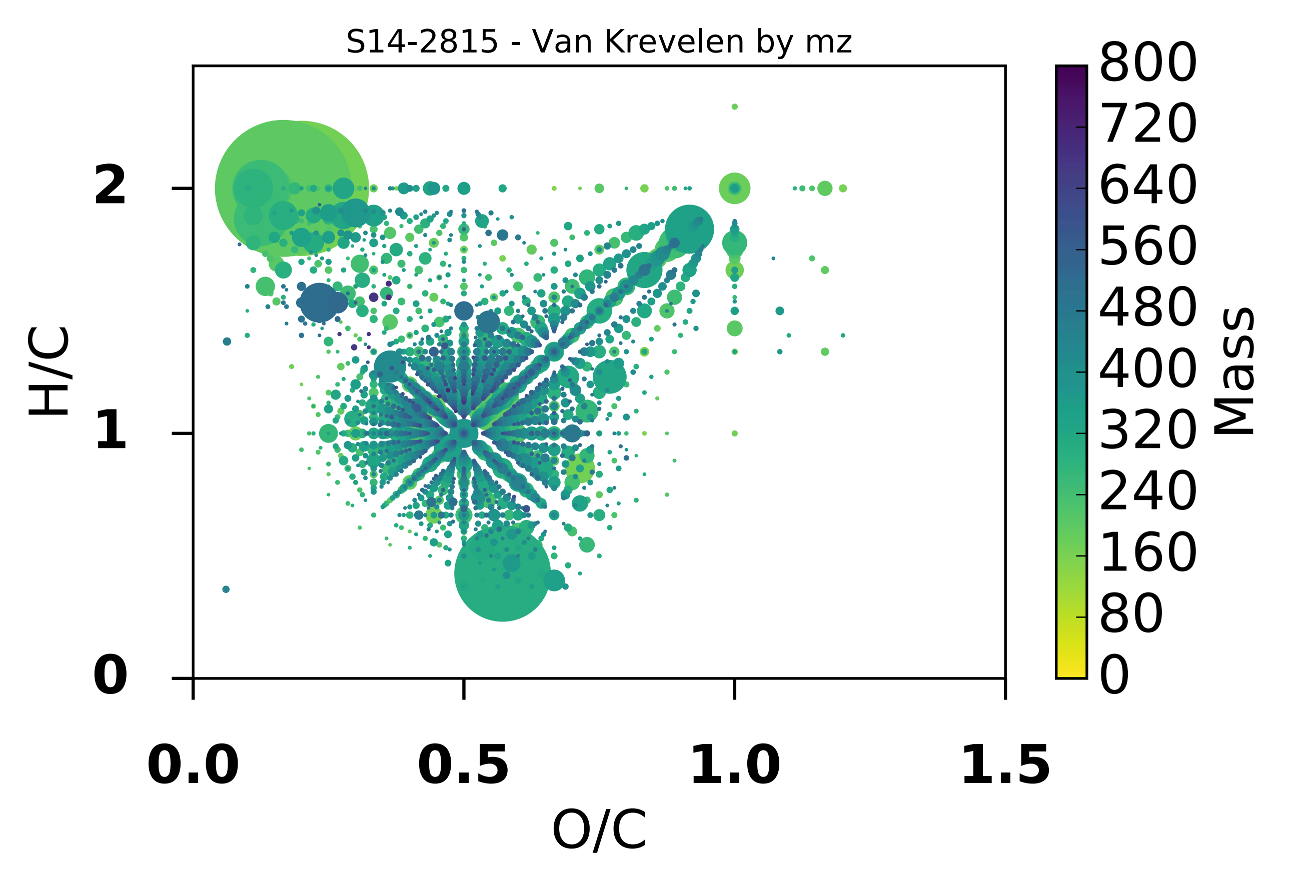
S14-2815 - Van Krevelen by mz
H/C
O/C
0.0
0.5
1.0
1.5
0
1
2
Mass
800
720
640
560
480
400
320
240
160
80
0

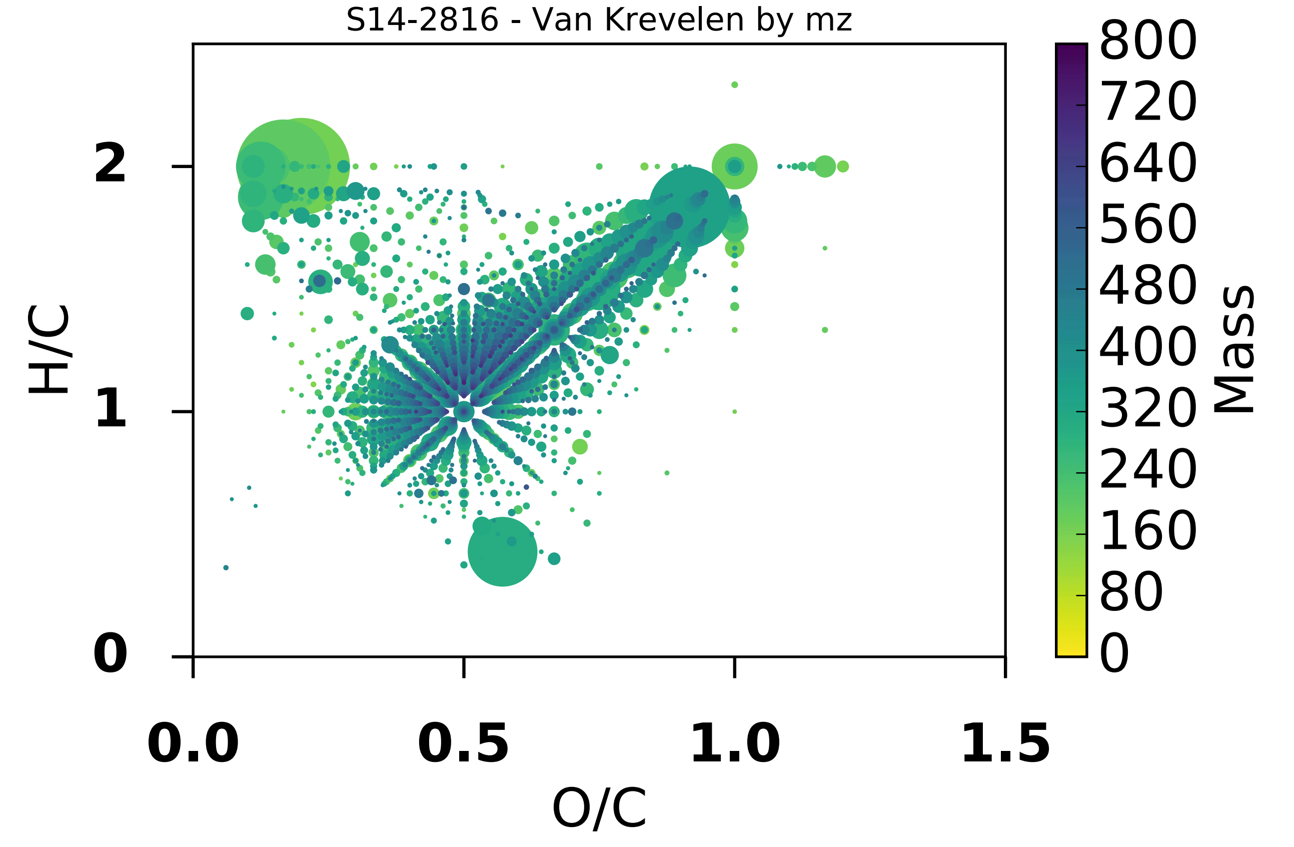

S14-2816 - Van Krevelen by mz
H/C
O/C
0
1
2
0.0
0.5
1.0
1.5
Mass
0
80
160
240
320
400
480
560
640
720
800

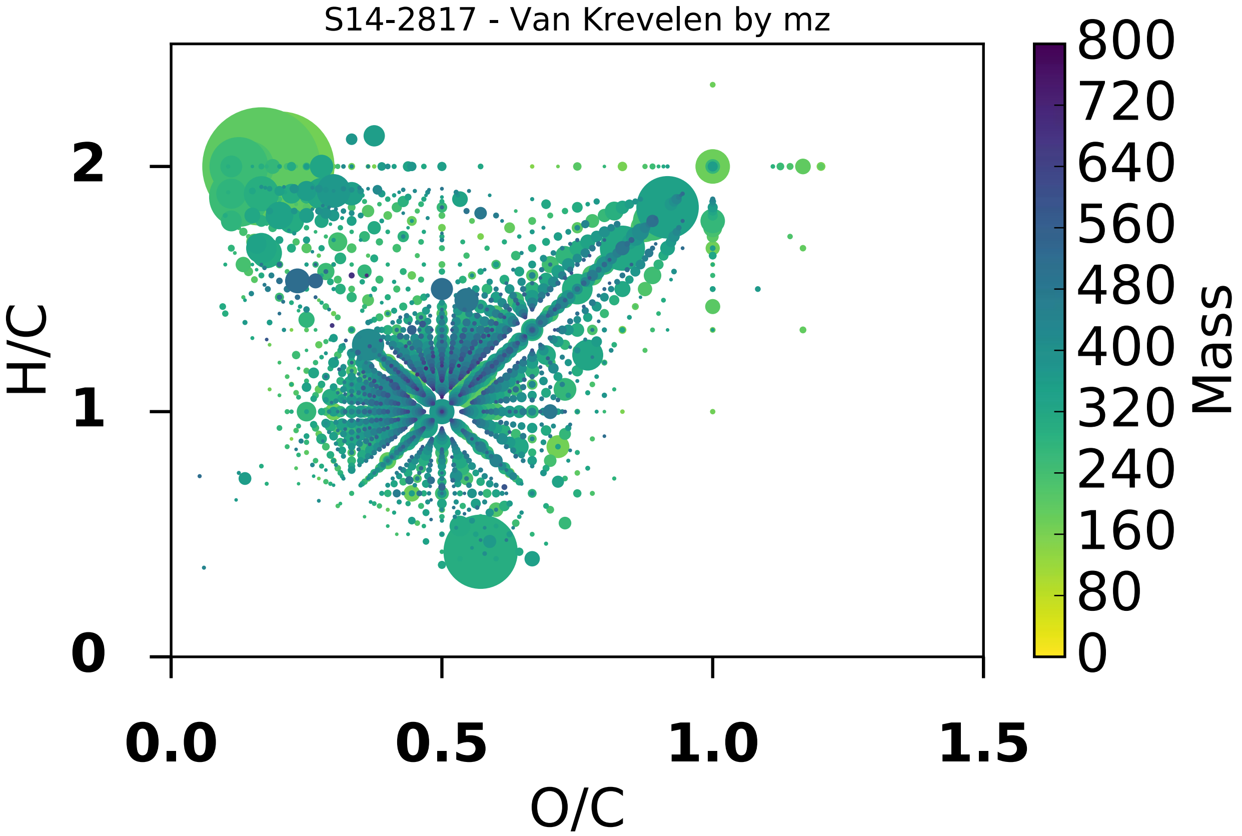
S14-2817 - Van Krevelen by mz
H/C
O/C
0
1
2
0.0
0.5
1.0
1.5
Mass
800
720
640
560
480
400
320
240
160
80
0

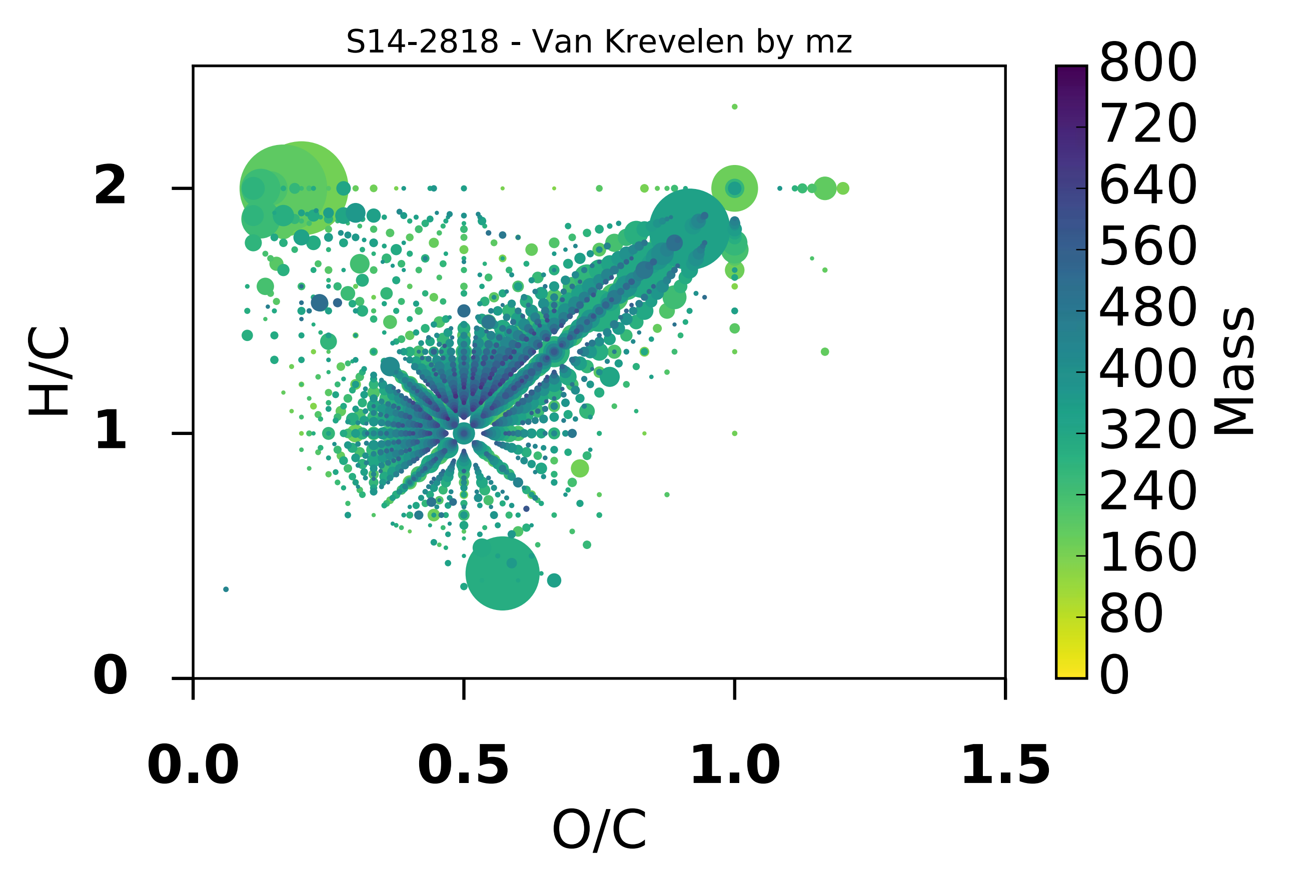

S14-2818 - Van Krevelen by mz
H/C
O/C
Mass
0
1
2
0.0
0.5
1.0
1.5
800
720
640
560
480
400
320
240
160
80
0

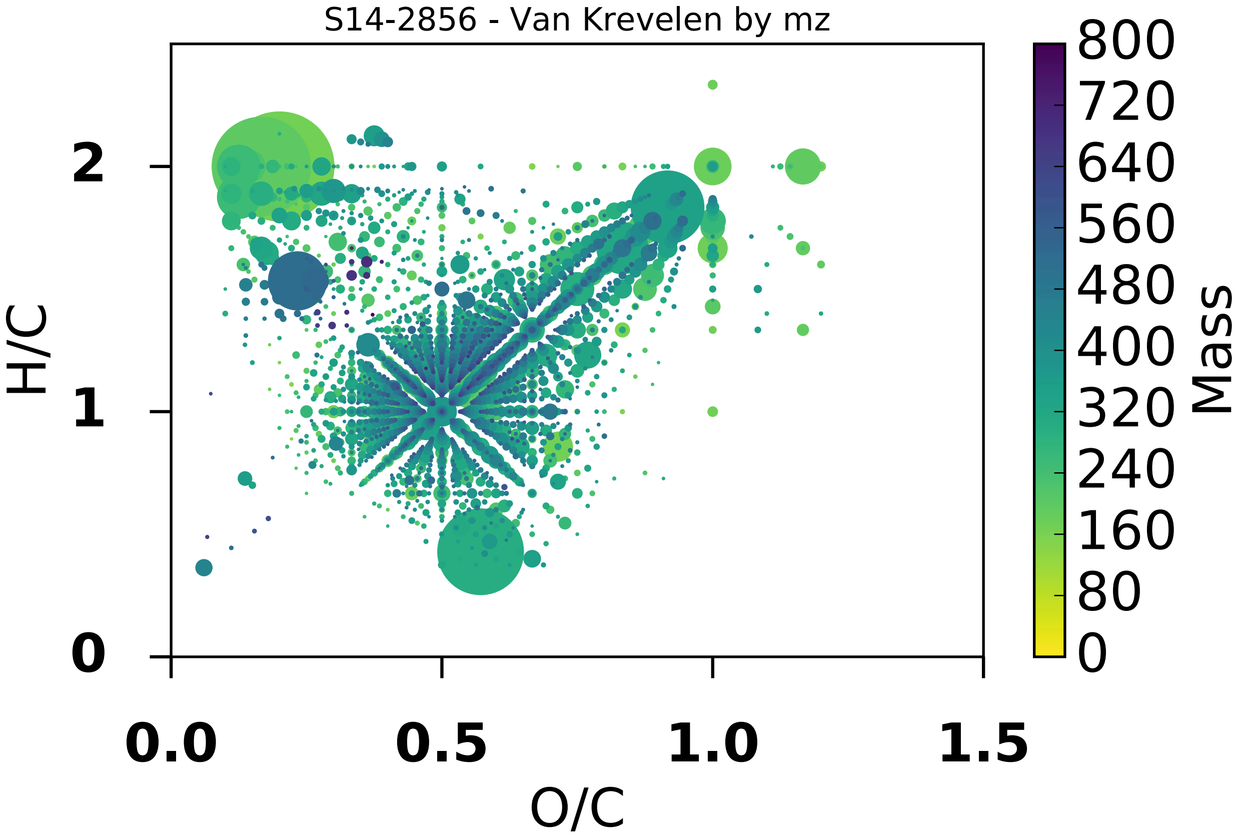

S14-2856 - Van Krevelen by mz
H/C
O/C
0
1
2
0.0
0.5
1.0
1.5
Mass
800
720
640
560
480
400
320
240
160
80
0

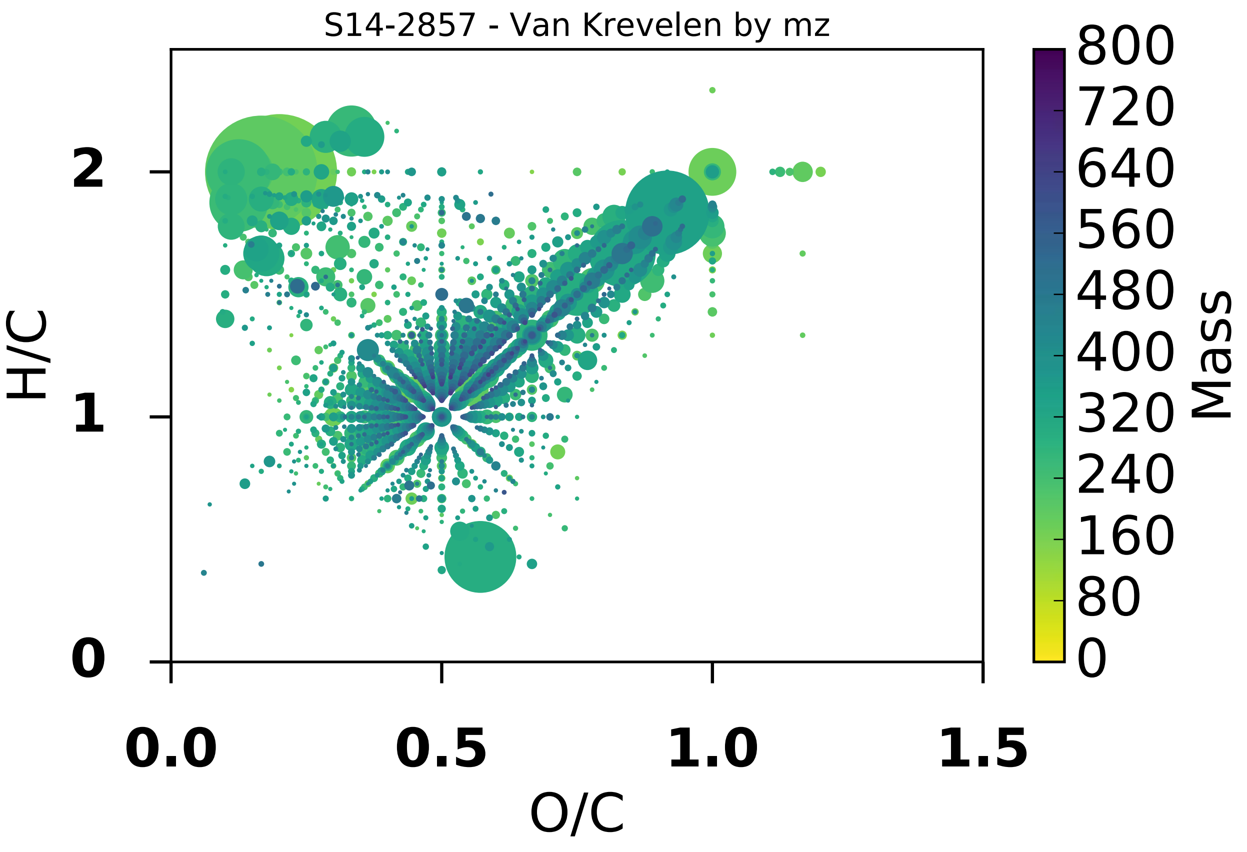

S14-2857 - Van Krevelen by mz
H/C
2
1
0
0.0
0.5
1.0
1.5
O/C
800
720
640
560
480
400
320
240
160
80
0
Mass

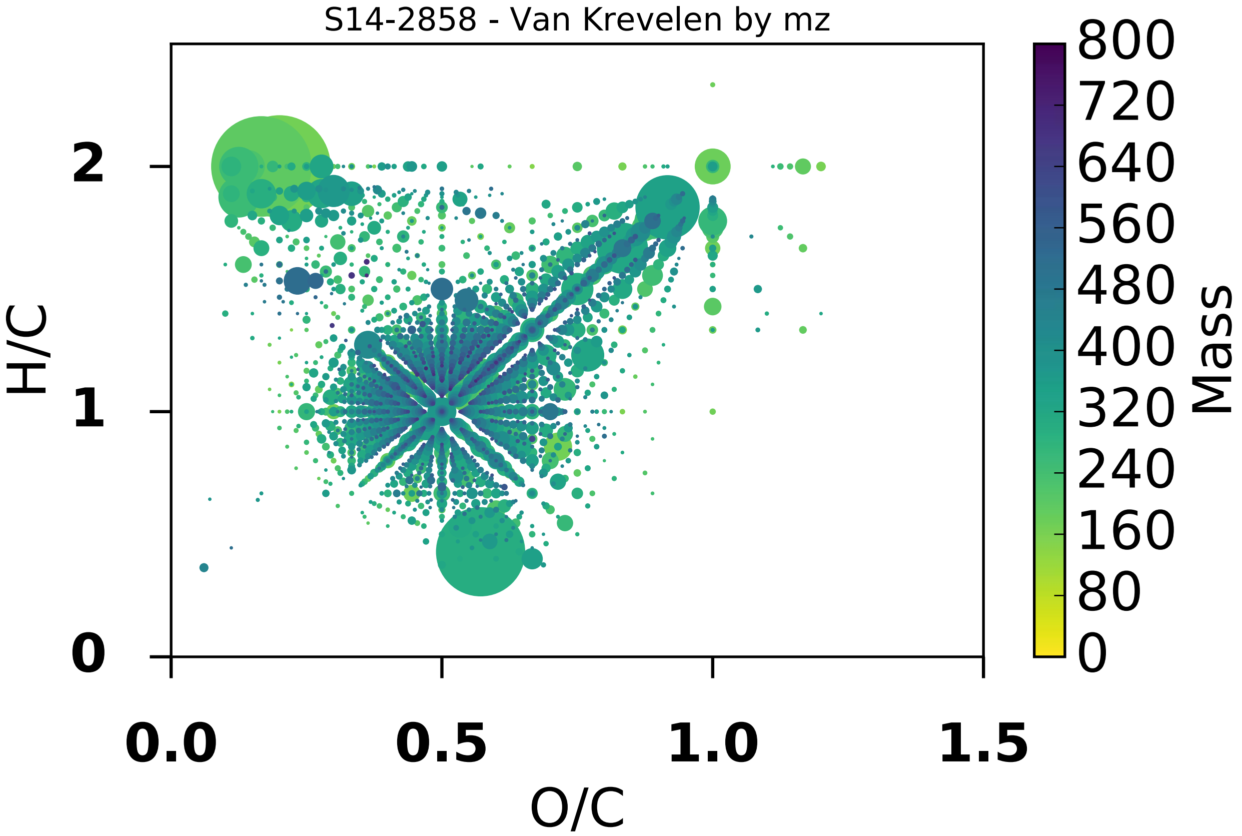

S14-2858 - Van Krevelen by mz
H/C
0
1
2
O/C
0.0
0.5
1.0
1.5
Mass
0
80
160
240
320
400
480
560
640
720
800
