# Supplementary material for: Chemical Diversity and Complexity of Scotch Whisky as Revealed by High-Resolution Mass Spectrometry
Source: J Am Soc Mass Spectrom. 2016 Oct 17;28(1):200–13. doi: 10.1007/s13361-016-1513-y (PMC5174148; doi:10.1007/s13361-016-1513-y)
Supplement: Supplementary file 3 — (PDF 13686 kb) [file 13361_2016_1513_MOESM3_ESM.pdf]

Chemical Diversity and Complexity of Scotch Whisky as Revealed by High-Resolution Mass Spectrometry

W. Kew, I. Goodall, D. Clarke and D. Uhrín

Journal of the American Society for Mass Spectrometry

Corresponding Authors:

- Dr David Clarke, EaStCHEM, School of Chemistry, Joseph Black Building, University of Edinburgh, Edinburgh, UK, EH9 3FJ, Tel: +44(0)131 650 4808, email: david.clarke@ed.ac.uk
- Dr Dušan Uhrín, EaStCHEM, School of Chemistry, Joseph Black Building, University of Edinburgh, Edinburgh, UK, EH9 3FJ, Tel: +44(0)131 650 4742, email: dusan.uhrin@ed.ac.uk

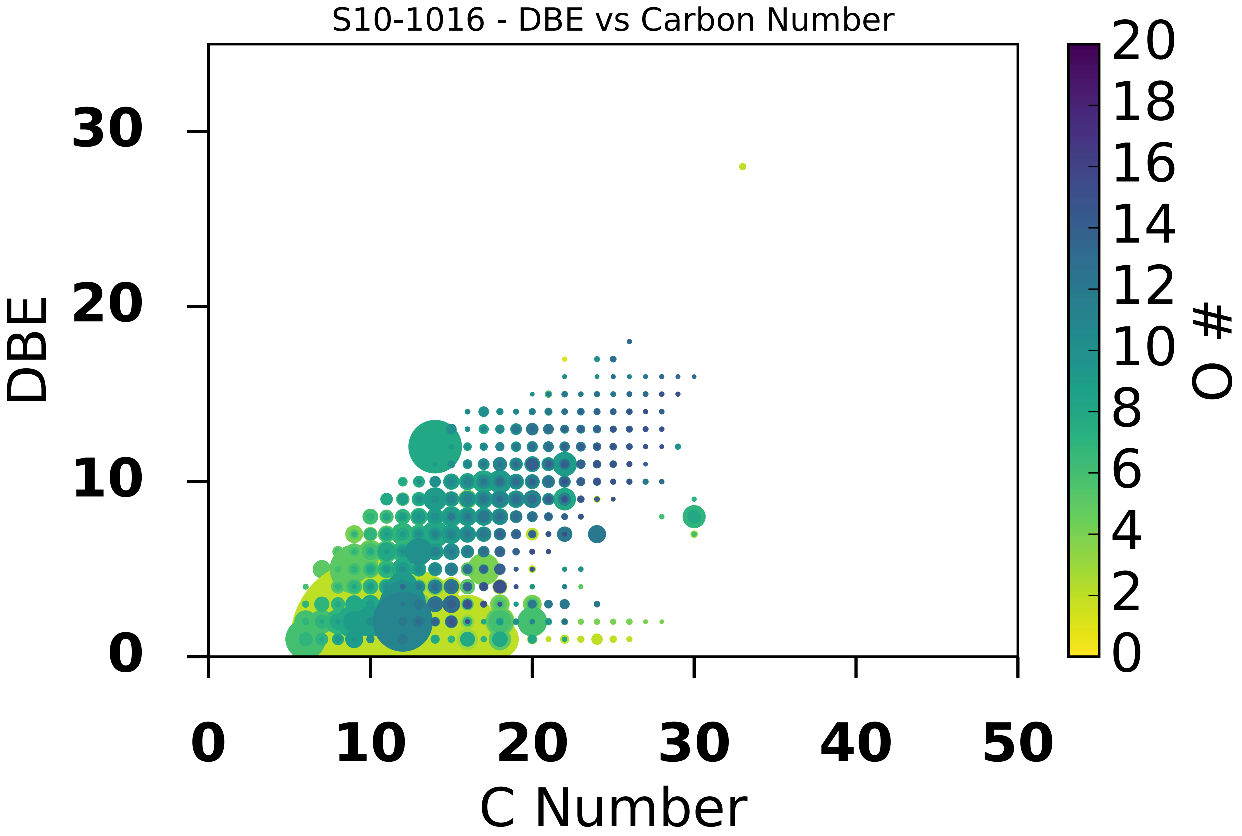

S10-1016 - DBE vs Carbon Number
DBE
30
20
10
0
0
10
20
30
40
50
C Number
20
18
16
14
12
10
8
6
4
2
0
O #

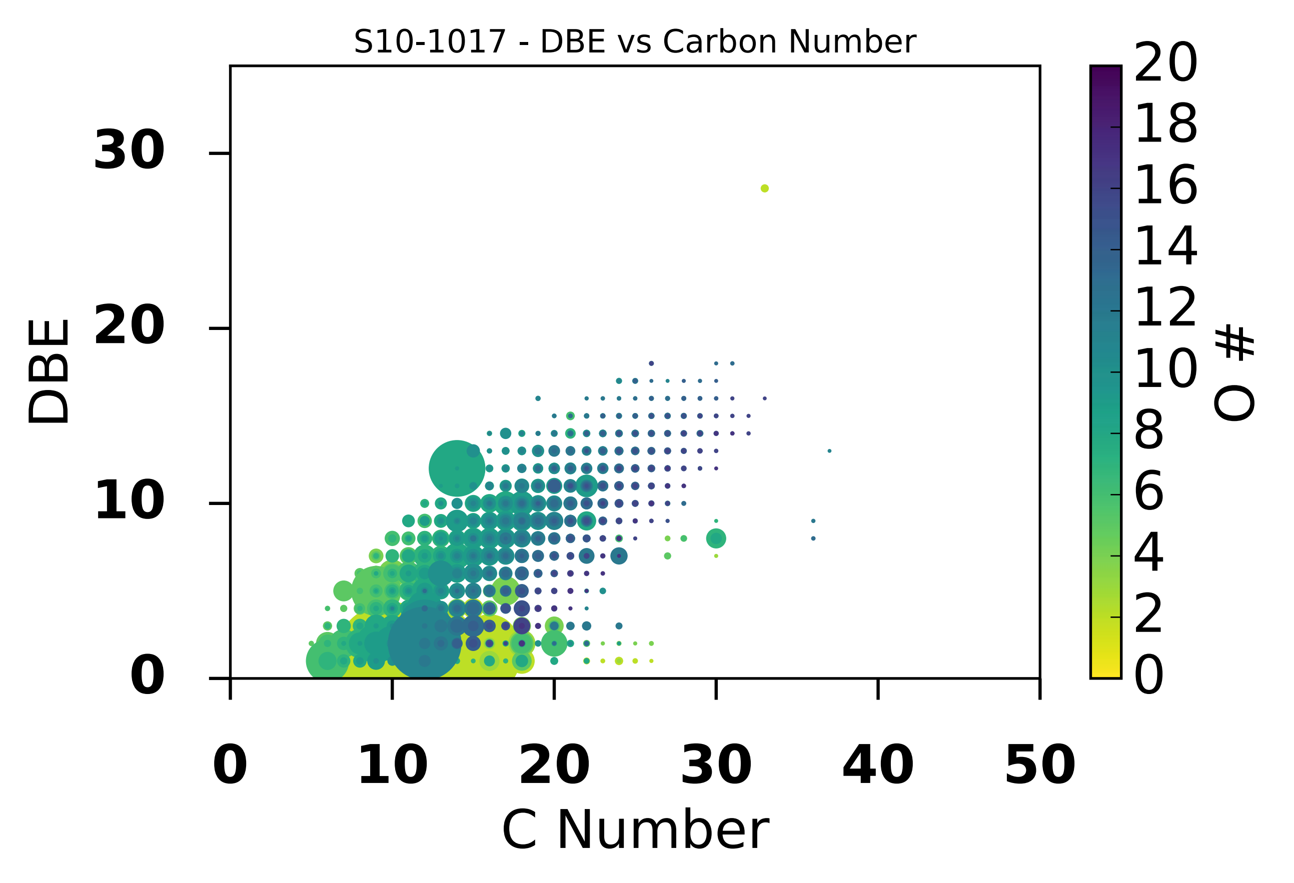

S10-1017 - DBE vs Carbon Number
DBE
30
20
10
0
0
10
20
30
40
50
C Number
O #
20
18
16
14
12
10
8
6
4
2
0

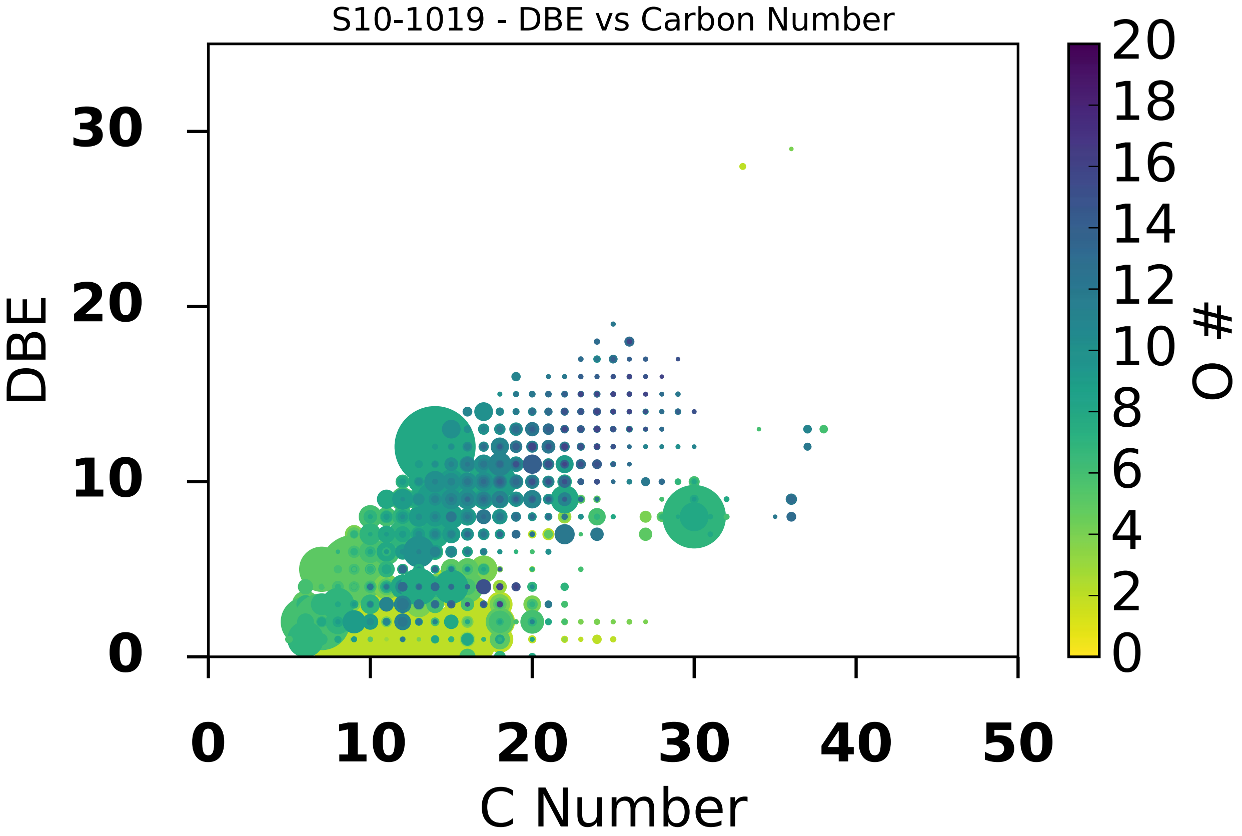
S10-1019 - DBE vs Carbon Number
DBE
30
20
10
0
0
10
20
30
40
50
C Number
O #
20
18
16
14
12
10
8
6
4
2
0

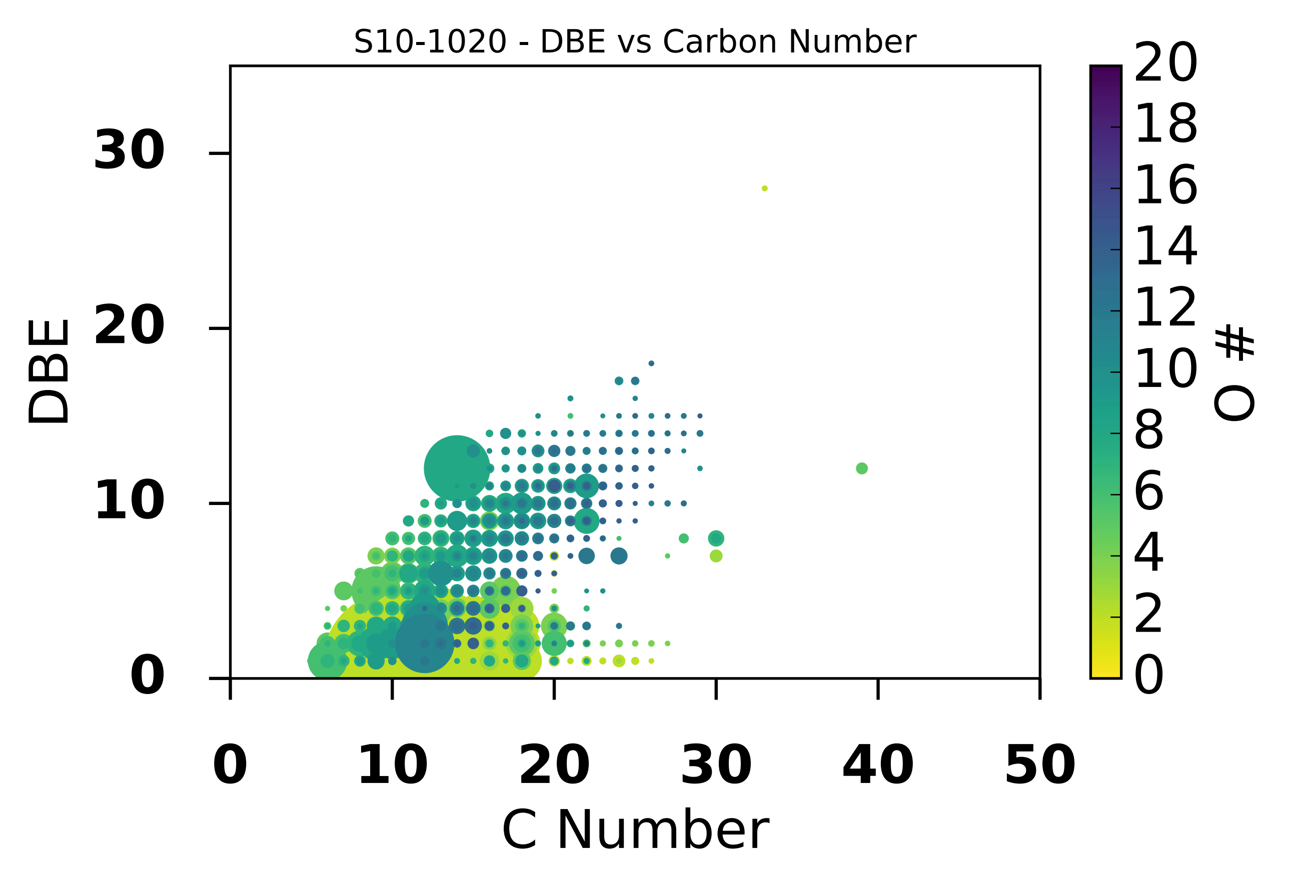
S10-1020 - DBE vs Carbon Number
DBE
30
20
10
0
0
10
20
30
40
50
C Number
20
18
16
14
12
10
8
6
4
2
0
O #

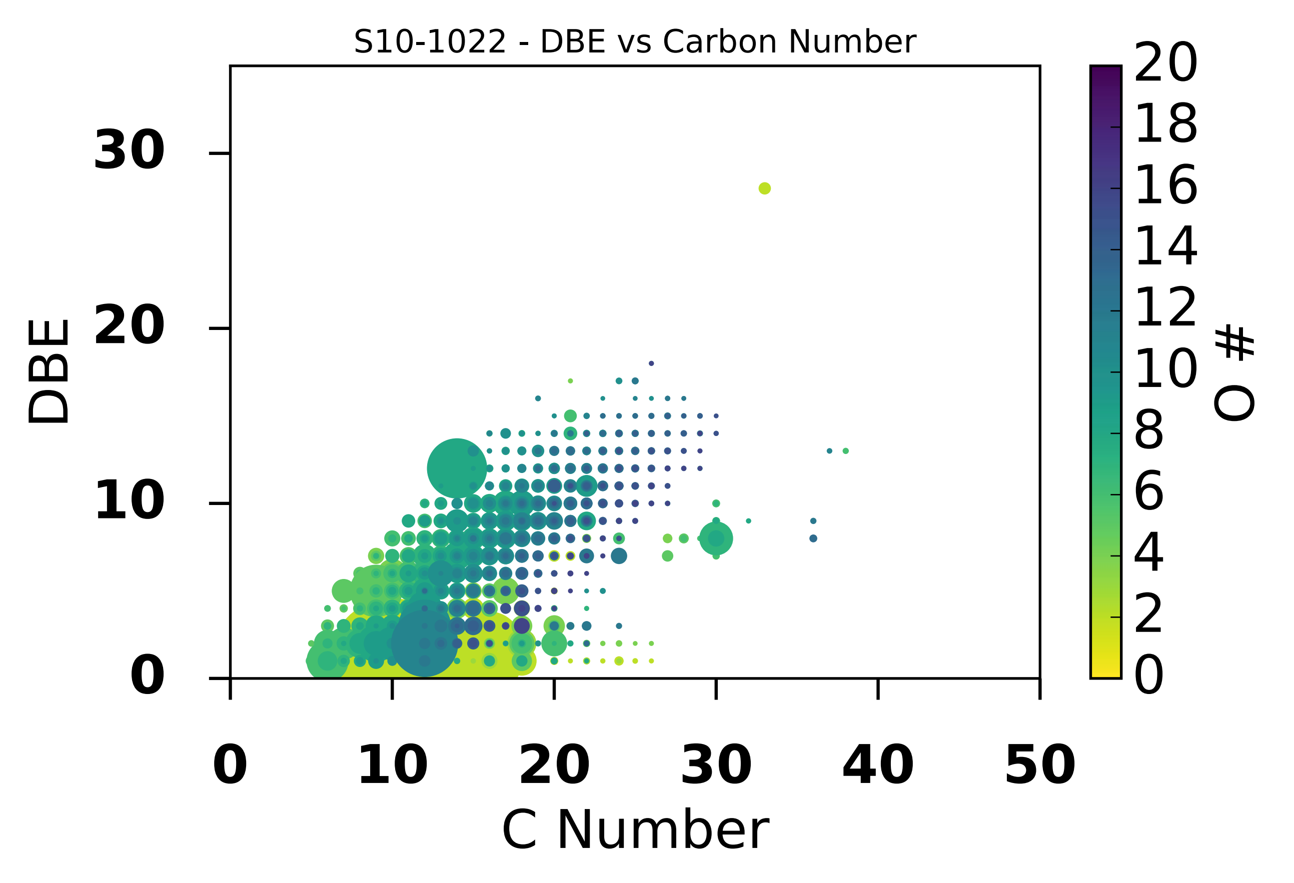
S10-1022 - DBE vs Carbon Number
DBE
0
10
20
30
C Number
0
10
20
30
40
50
O #
0
2
4
6
8
10
12
14
16
18
20

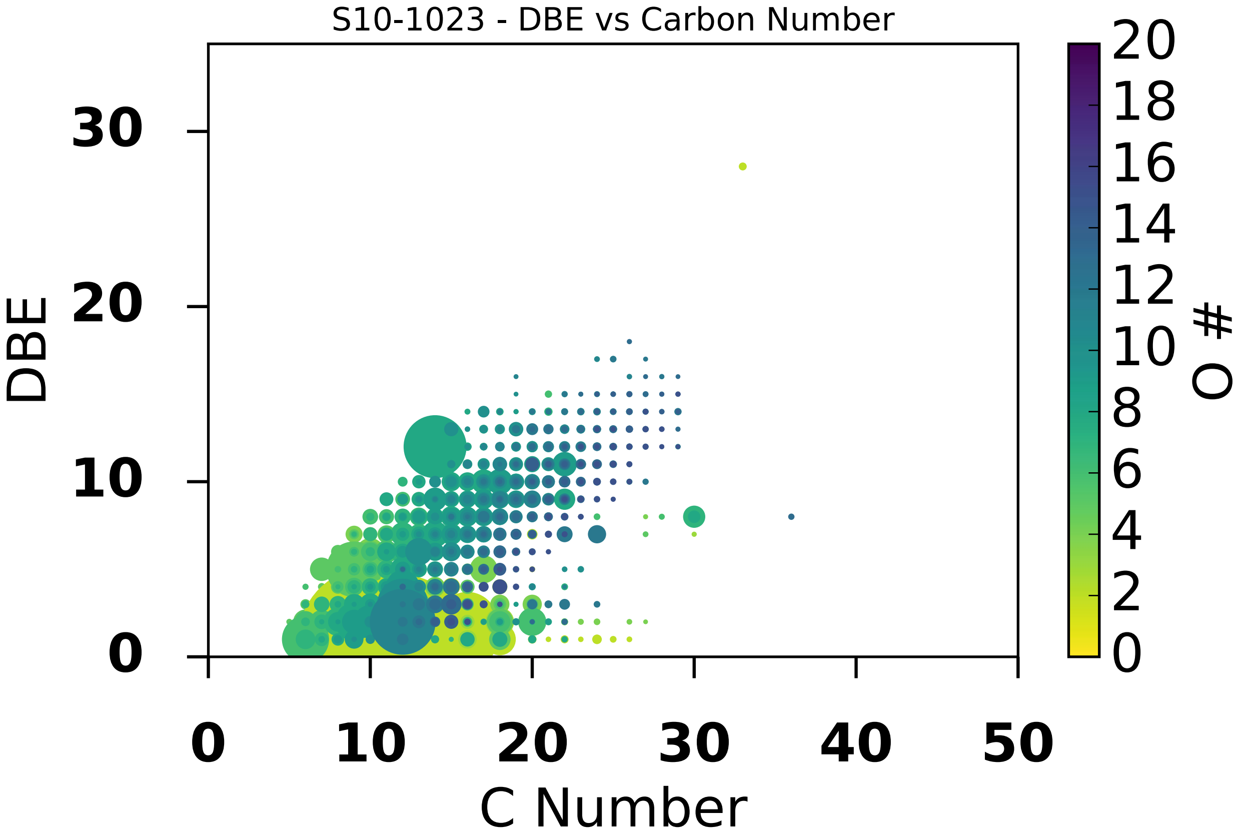

S10-1023 - DBE vs Carbon Number
DBE
30
20
10
0
0
10
20
30
40
50
C Number
20
18
16
14
12
10
8
6
4
2
0
O #

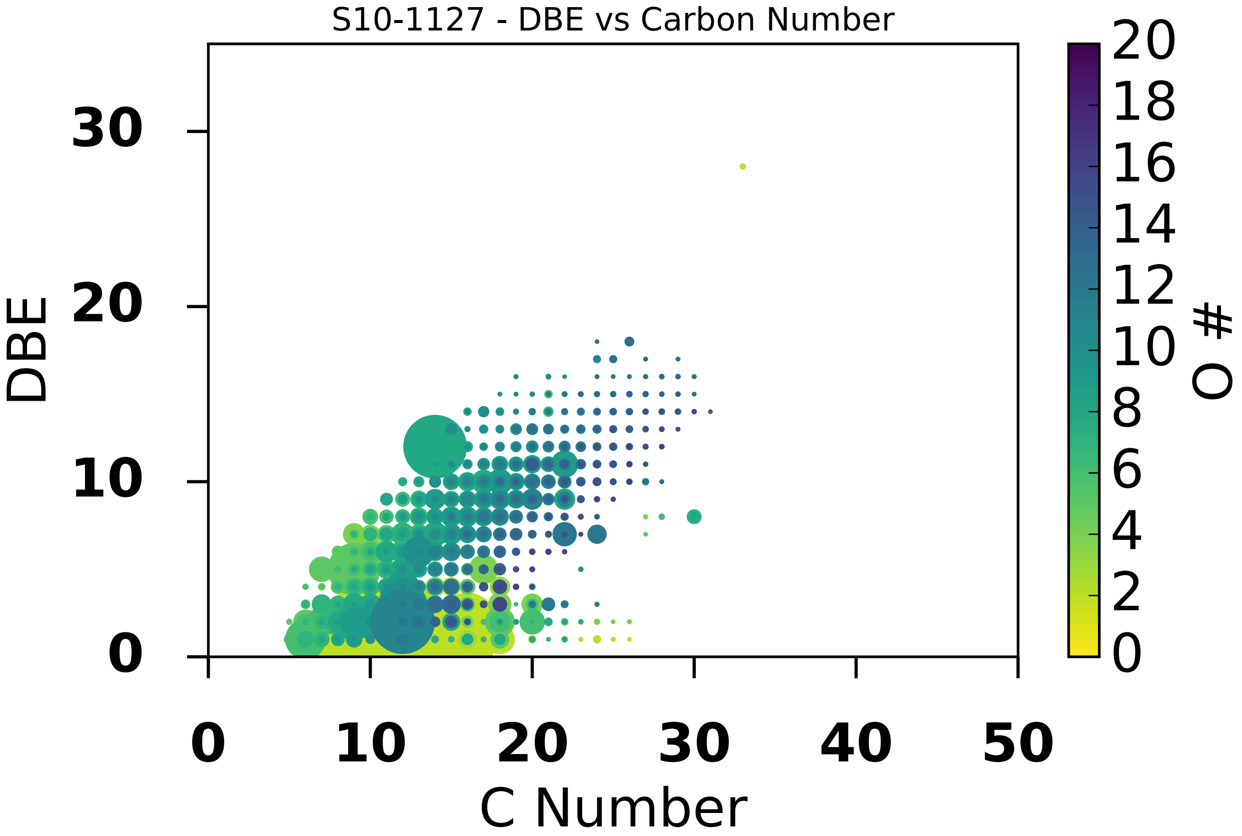
S10-1127 - DBE vs Carbon Number
DBE
30
20
10
0
0
10
20
30
40
50
C Number
20
18
16
14
12
10
8
6
4
2
0
O #

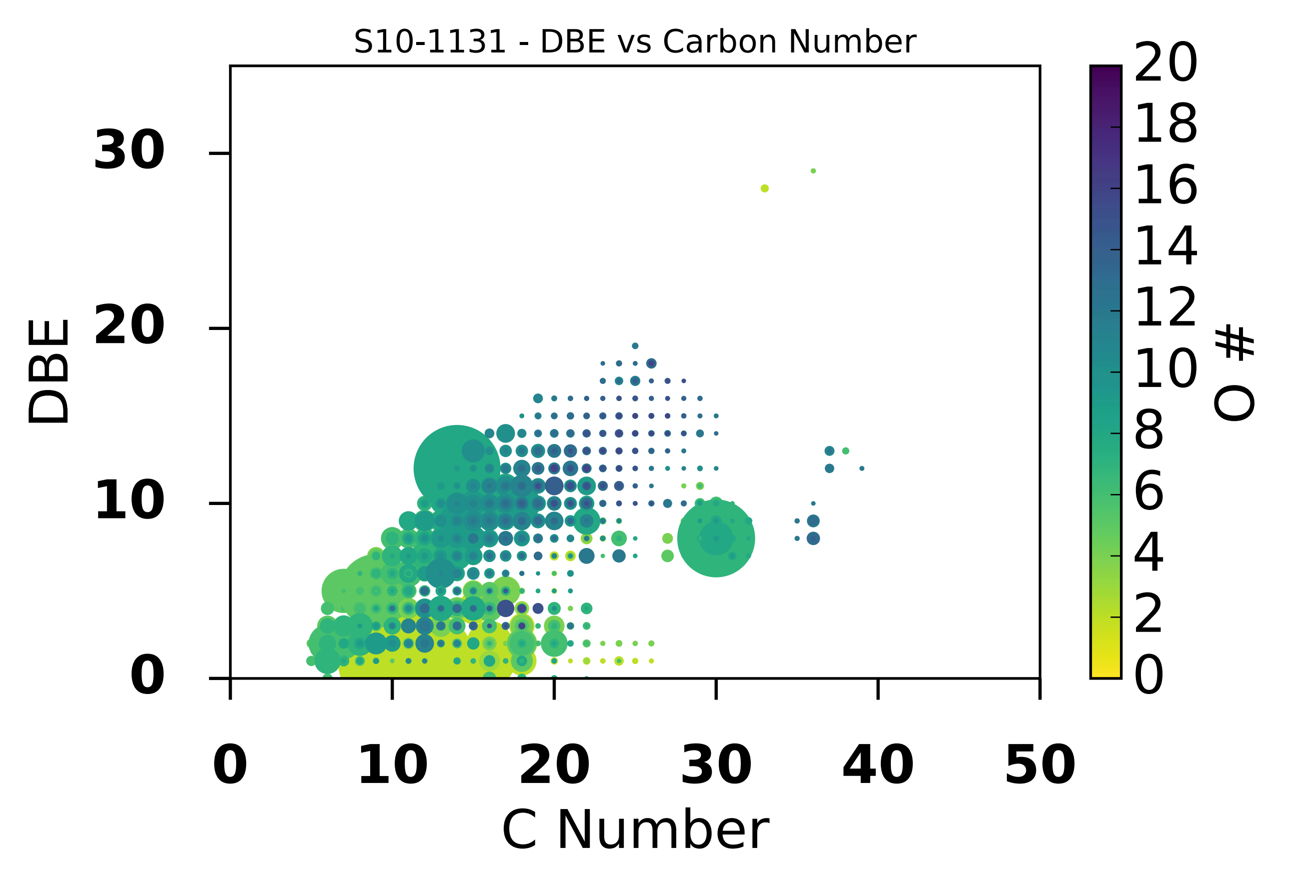
S10-1131 - DBE vs Carbon Number
DBE
C Number
O #
0
10
20
30
40
50
0
2
4
6
8
10
12
14
16
18
20

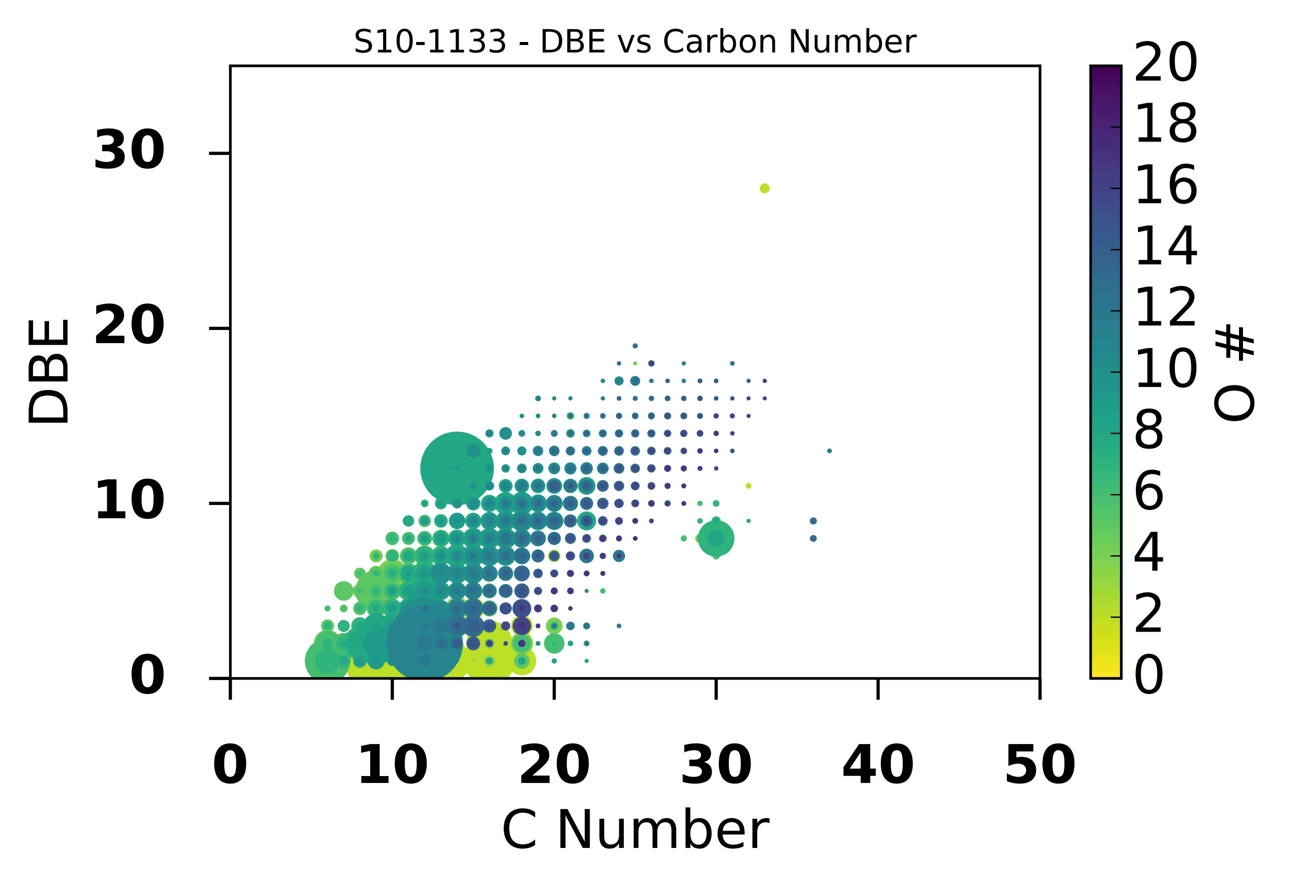

S10-1133 - DBE vs Carbon Number
DBE
30
20
10
0
0
10
20
30
40
50
C Number
O #
20
18
16
14
12
10
8
6
4
2
0

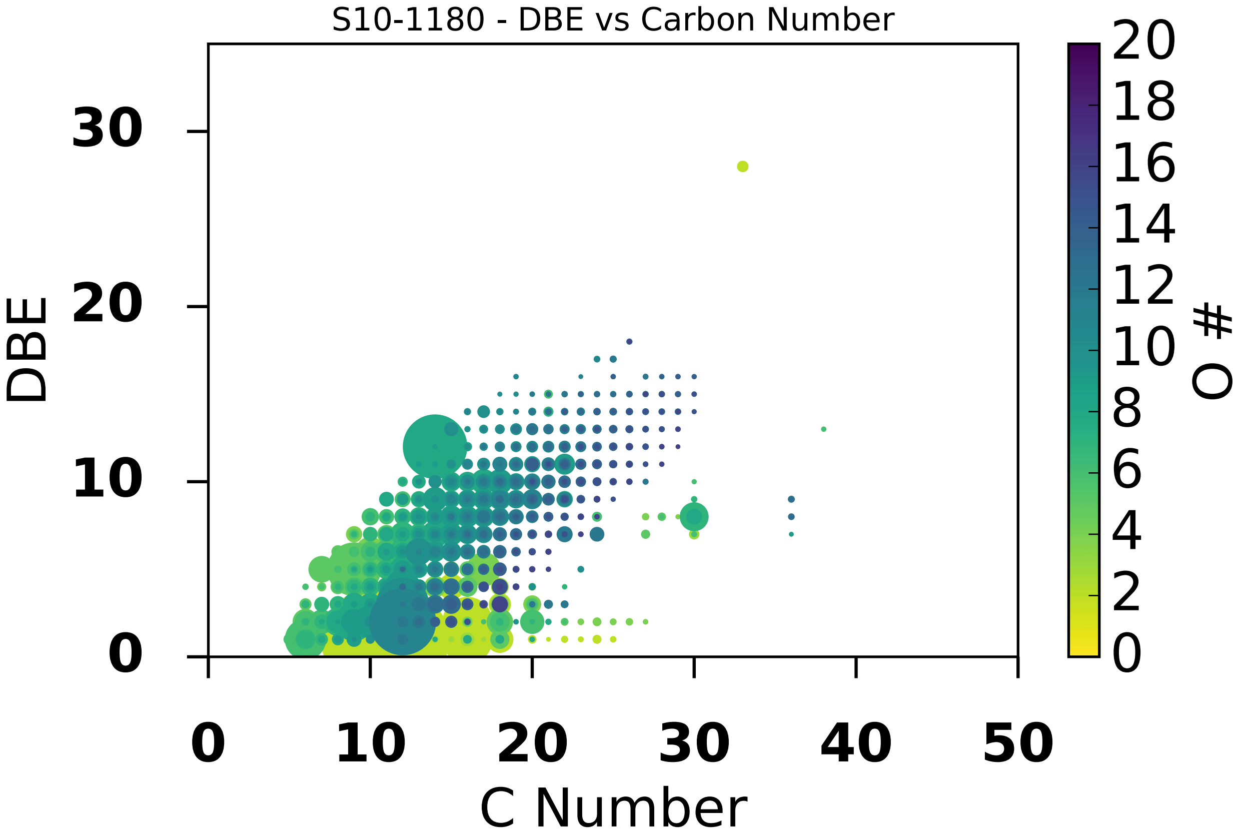

S10-1180 - DBE vs Carbon Number
DBE
0
10
20
30
C Number
0
10
20
30
40
50
O #
0
2
4
6
8
10
12
14
16
18
20

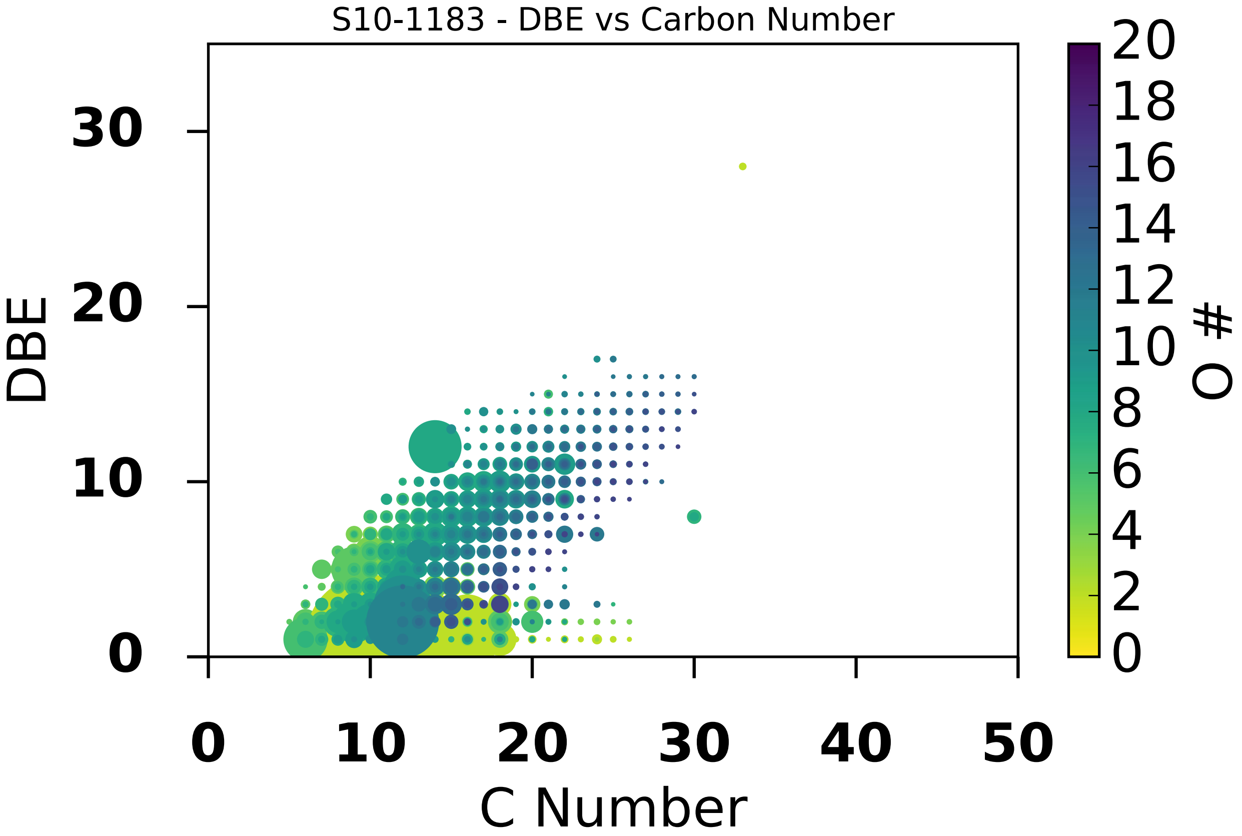
S10-1183 - DBE vs Carbon Number
DBE
0
10
20
30
C Number
0
10
20
30
40
50
O #
0
2
4
6
8
10
12
14
16
18
20

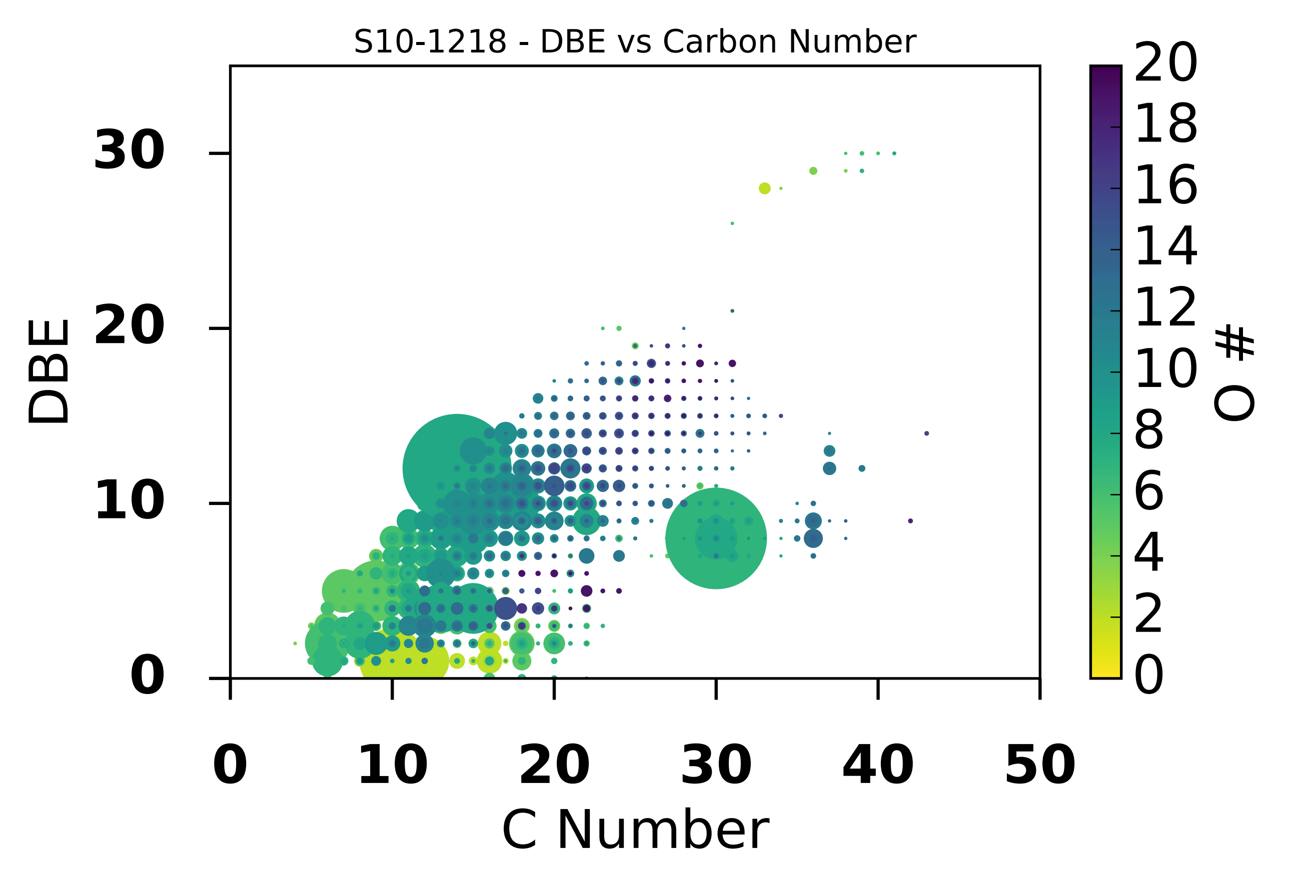
S10-1218 - DBE vs Carbon Number
DBE
0
10
20
30
C Number
0
10
20
30
40
50
O #
0
2
4
6
8
10
12
14
16
18
20

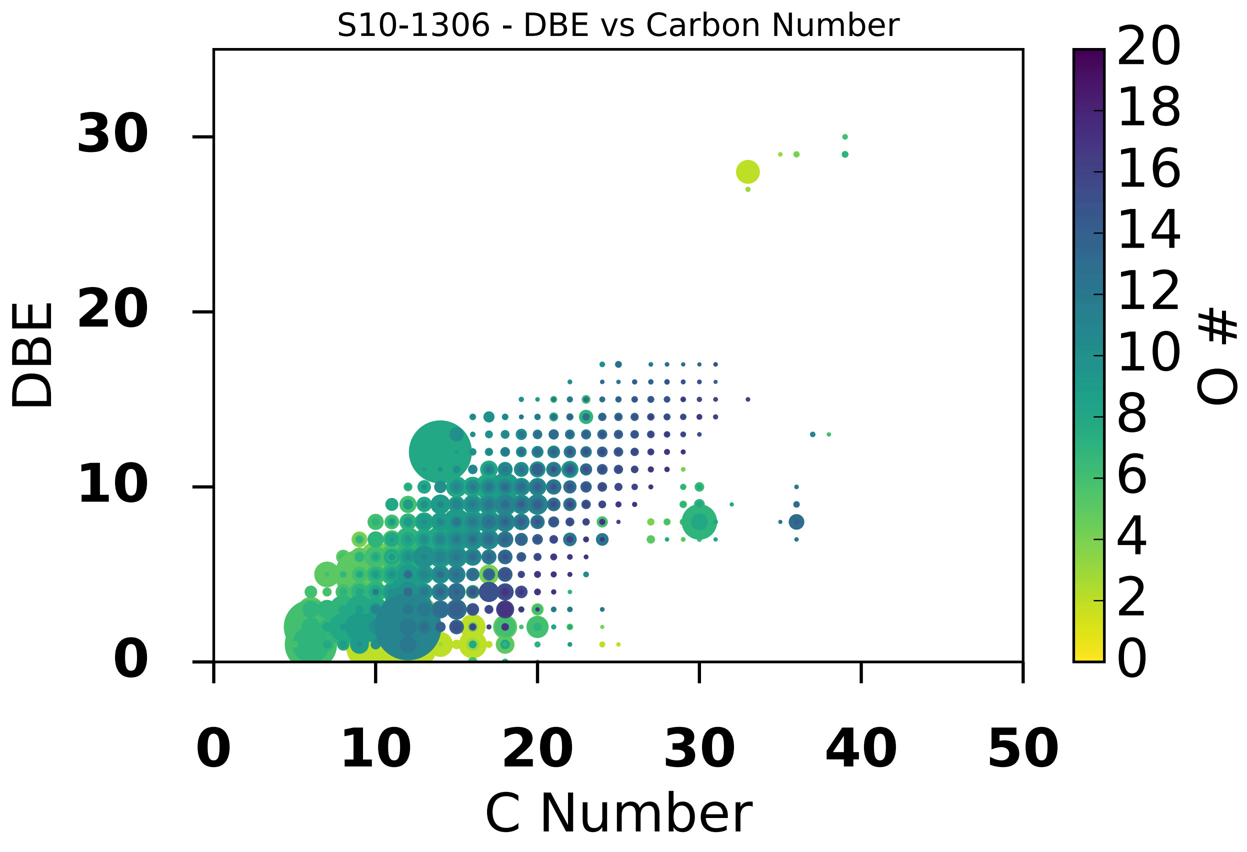

S10-1306 - DBE vs Carbon Number
DBE
30
20
10
0
0
10
20
30
40
50
C Number
20
18
16
14
12
10
8
6
4
2
0
O #

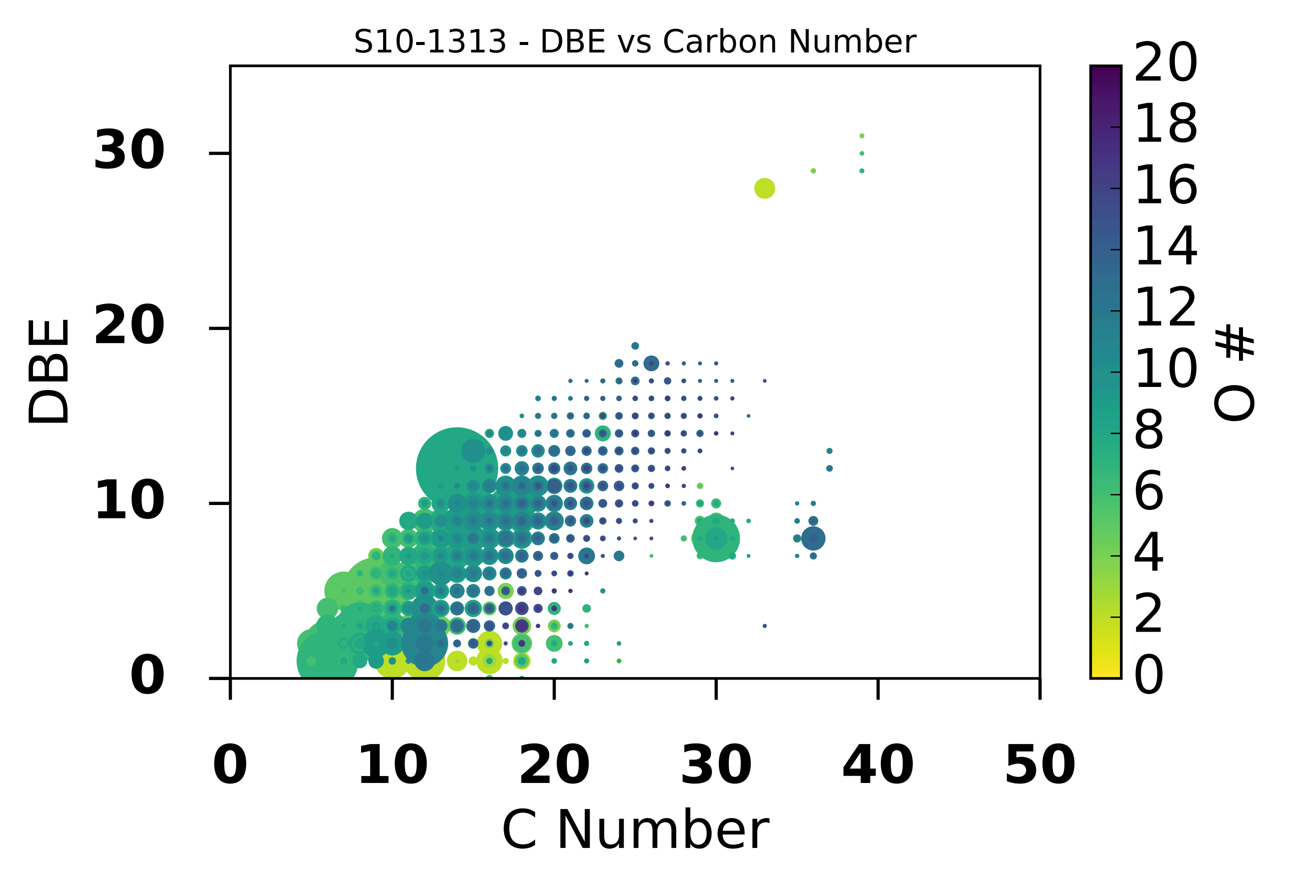

S10-1313 - DBE vs Carbon Number
DBE
C Number
O #
0
10
20
30
40
50
0
2
4
6
8
10
12
14
16
18
20

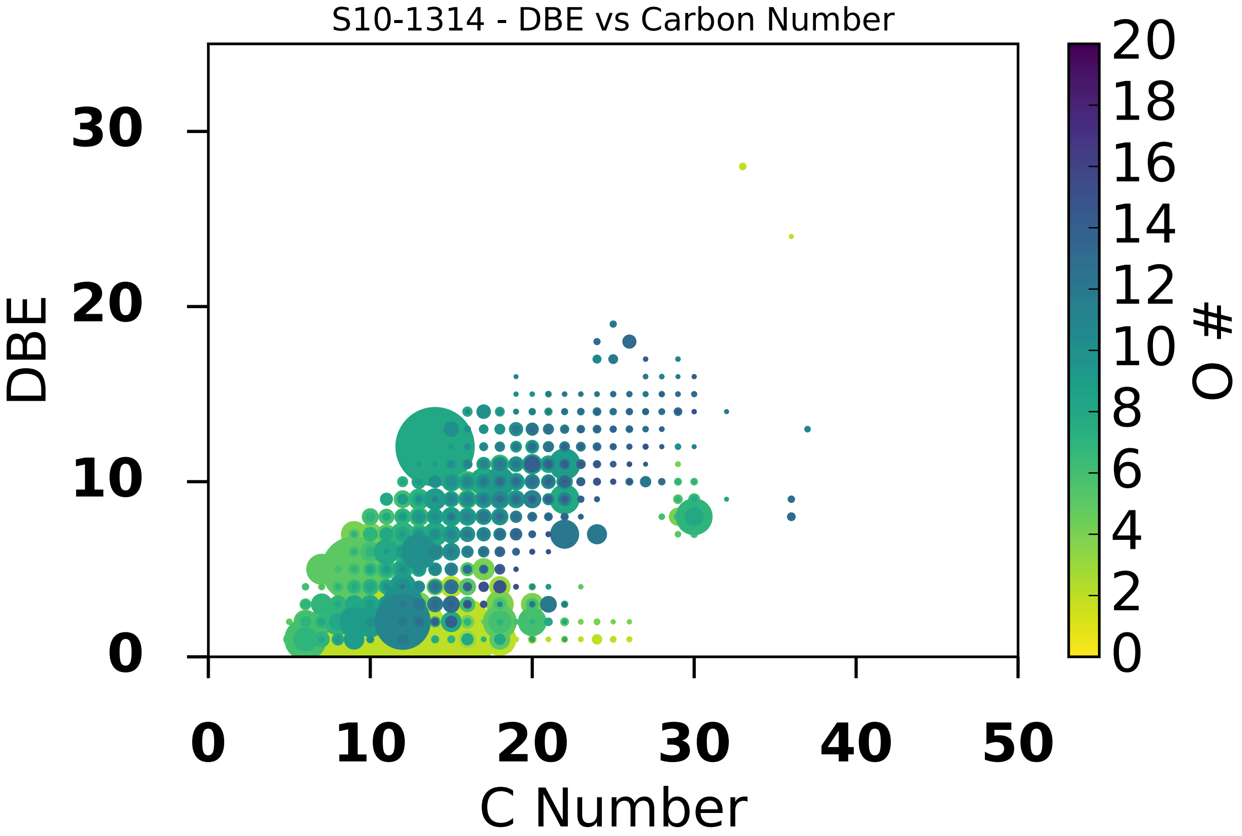
S10-1314 - DBE vs Carbon Number
DBE
30
20
10
0
0
10
20
30
40
50
C Number
O #
20
18
16
14
12
10
8
6
4
2
0

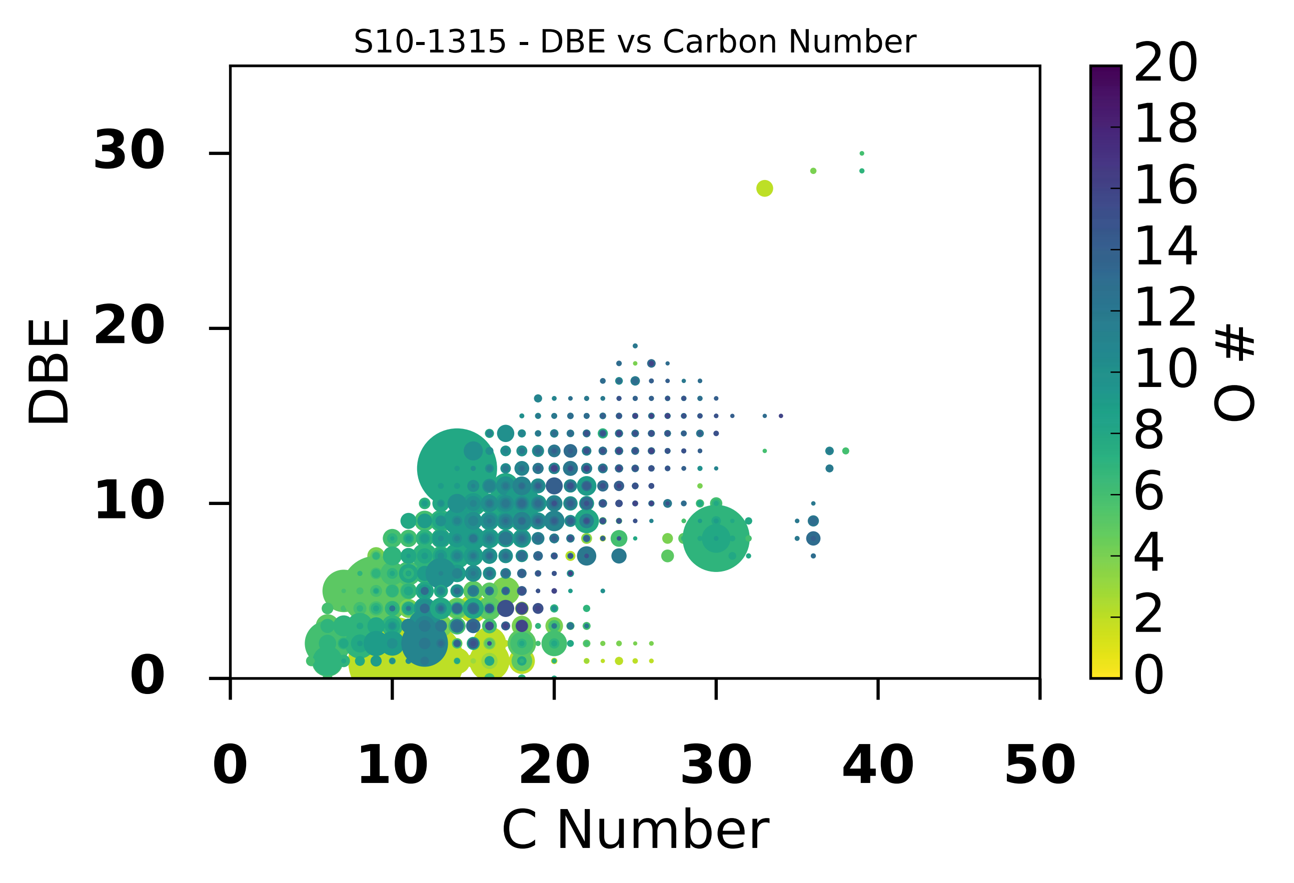
S10-1315 - DBE vs Carbon Number
DBE
30
20
10
0
0
10
20
30
40
50
C Number
O #
20
18
16
14
12
10
8
6
4
2
0

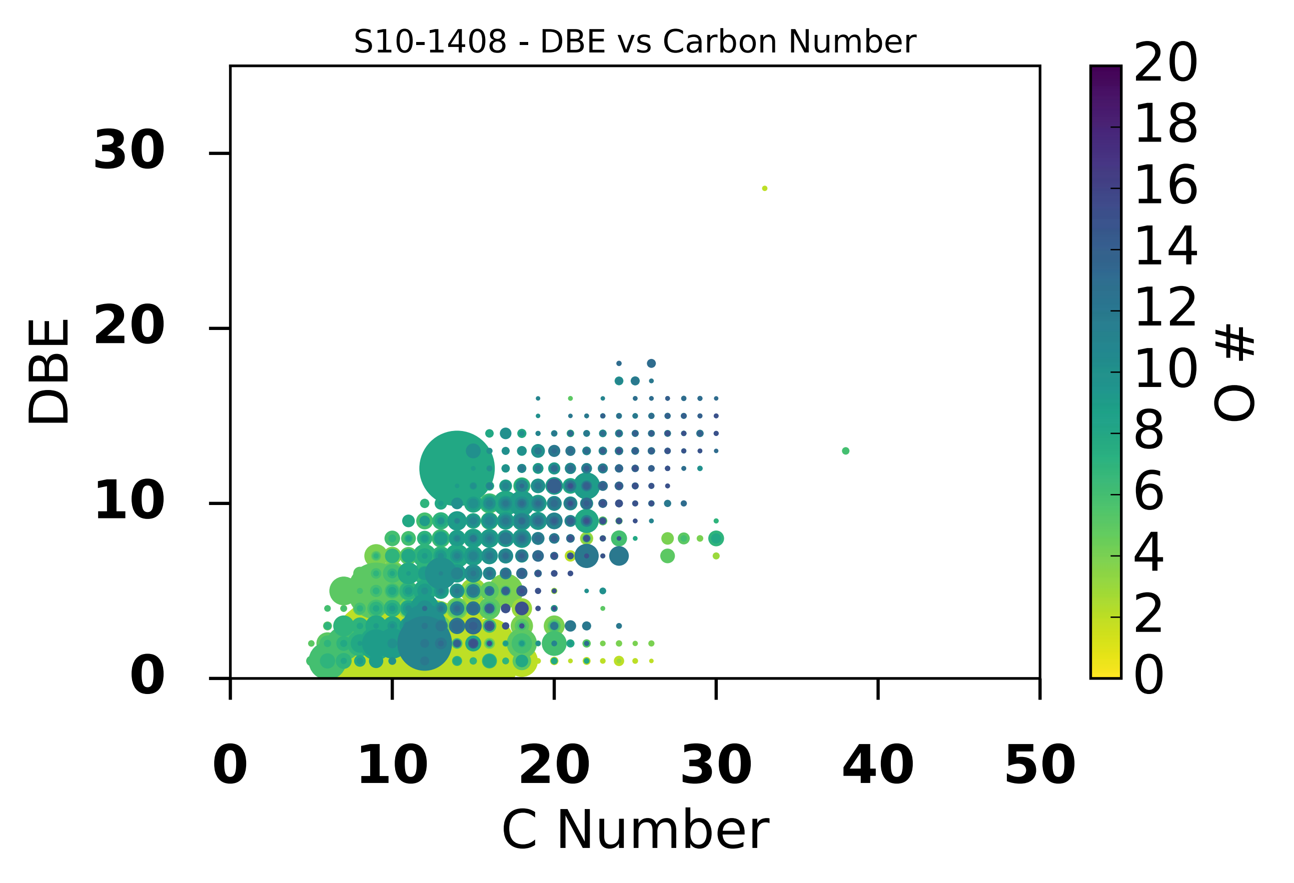

S10-1408 - DBE vs Carbon Number
DBE
C Number
O #
0
10
20
30
40
50
0
2
4
6
8
10
12
14
16
18
20

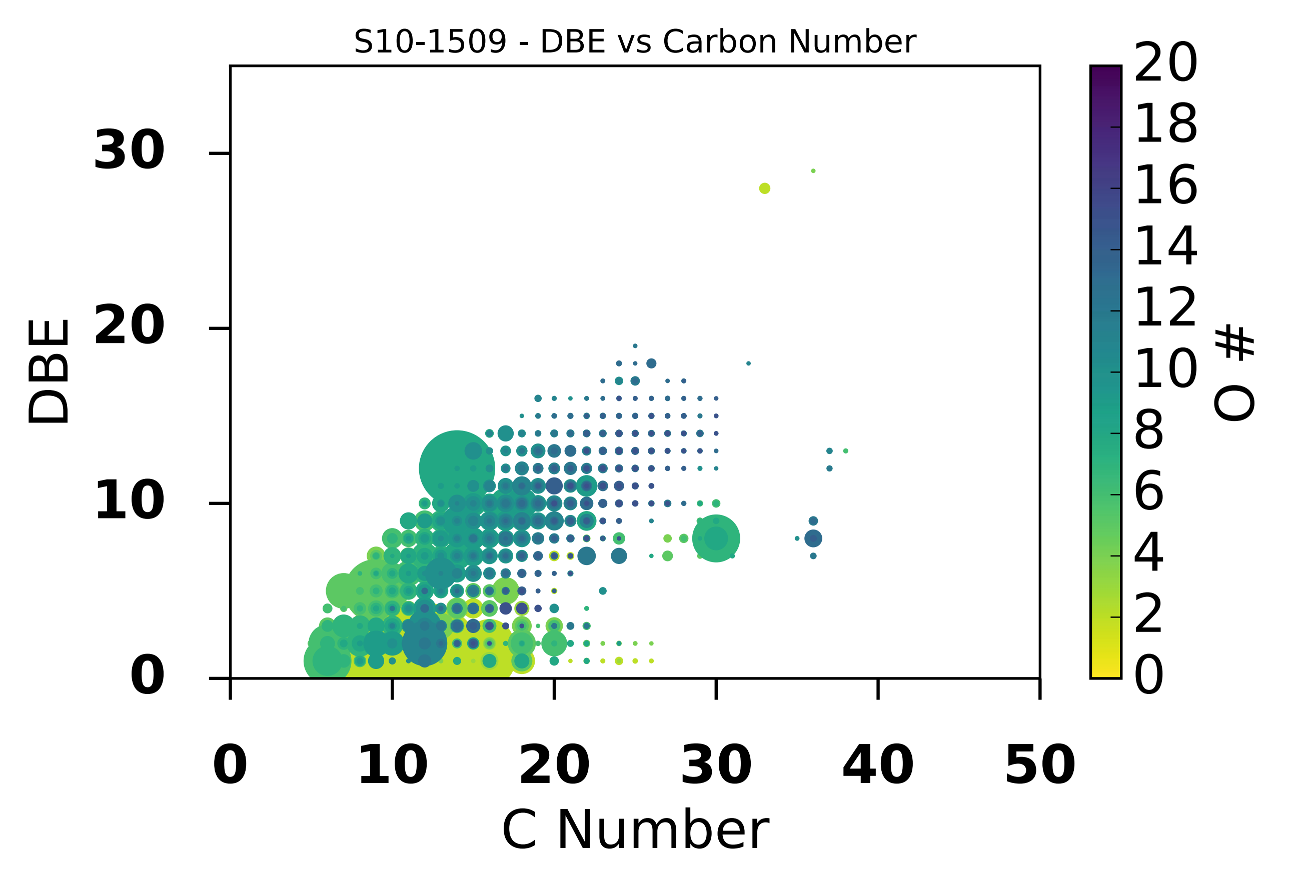
S10-1509 - DBE vs Carbon Number
DBE
C Number
O #
0
10
20
30
0
10
20
30
40
50
0
2
4
6
8
10
12
14
16
18
20

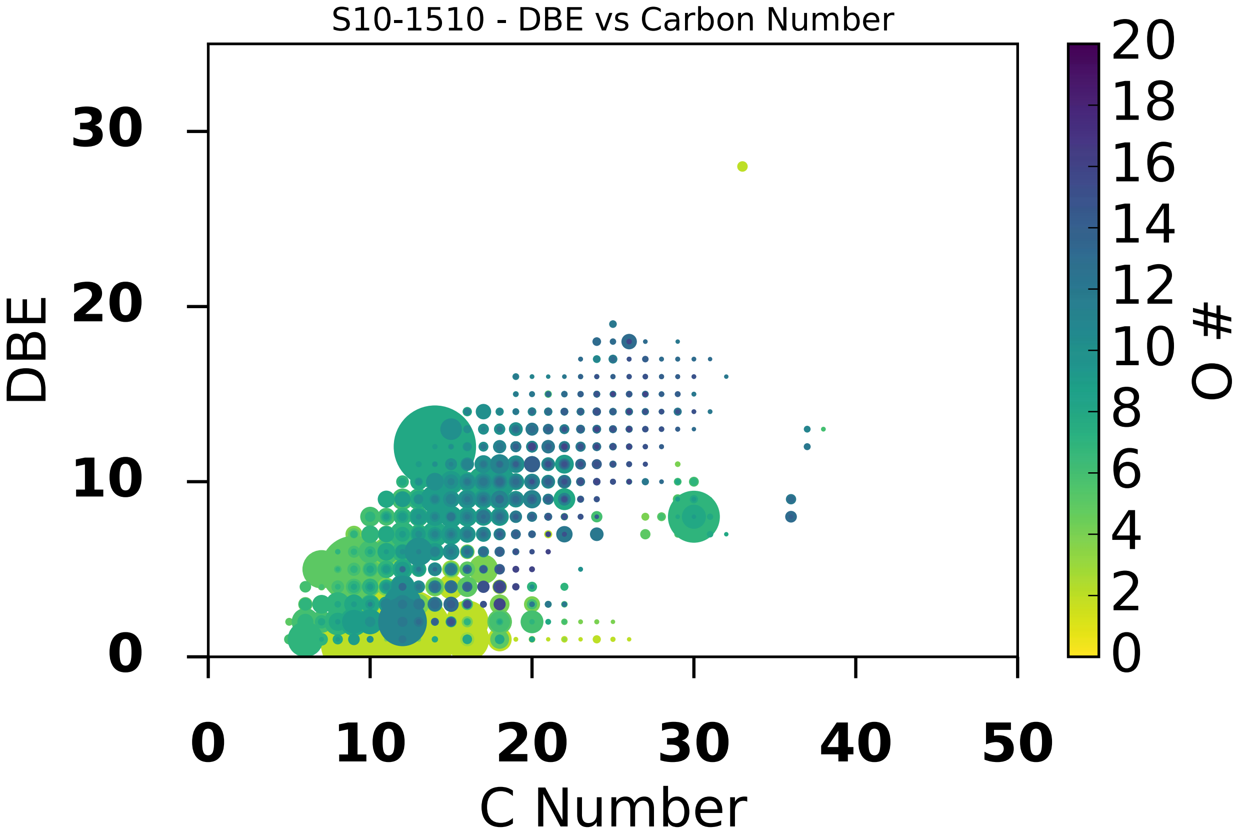
S10-1510 - DBE vs Carbon Number
DBE
30
20
10
0
0
10
20
30
40
50
C Number
O #
20
18
16
14
12
10
8
6
4
2
0

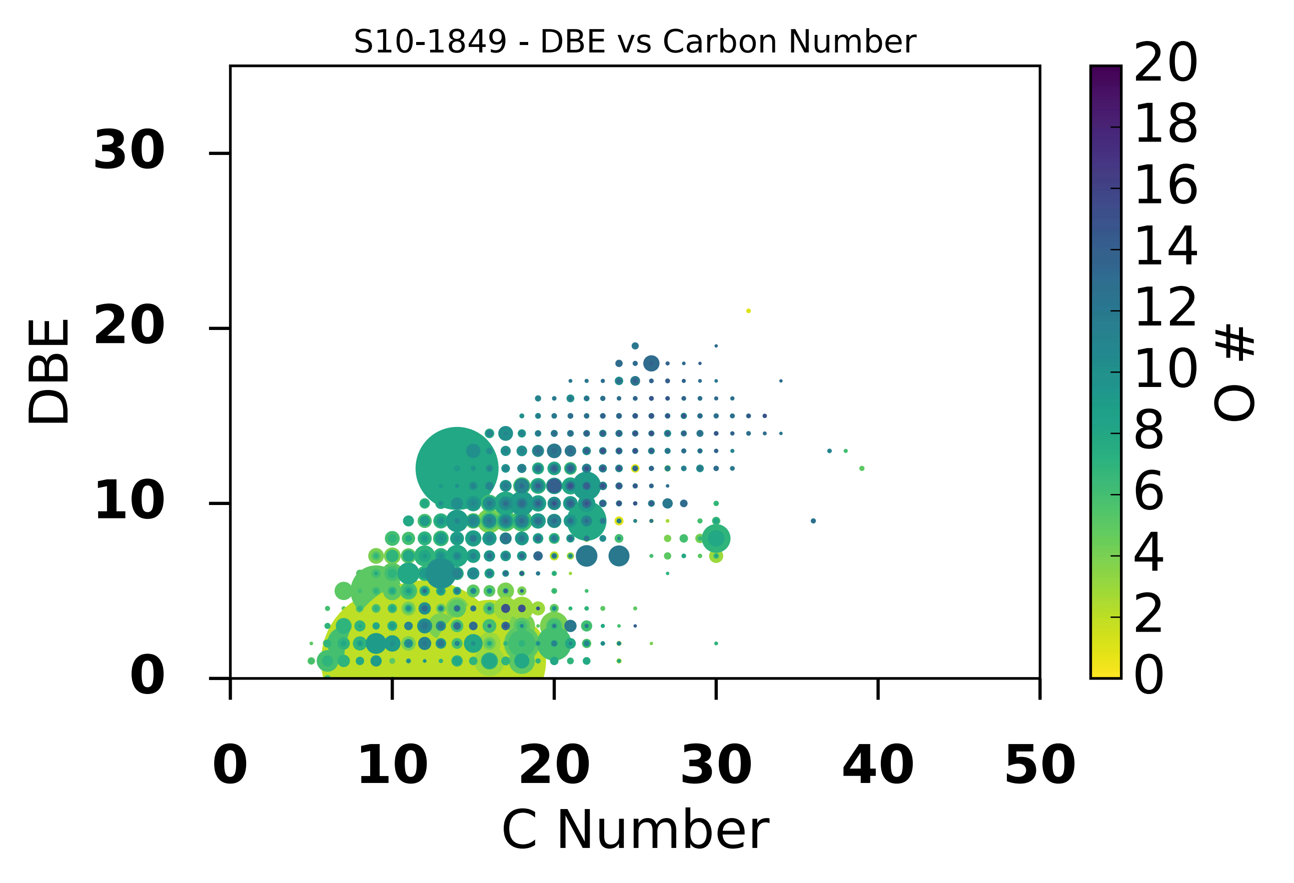
S10-1849 - DBE vs Carbon Number
DBE
30
20
10
0
0
10
20
30
40
50
C Number
O #
20
18
16
14
12
10
8
6
4
2
0

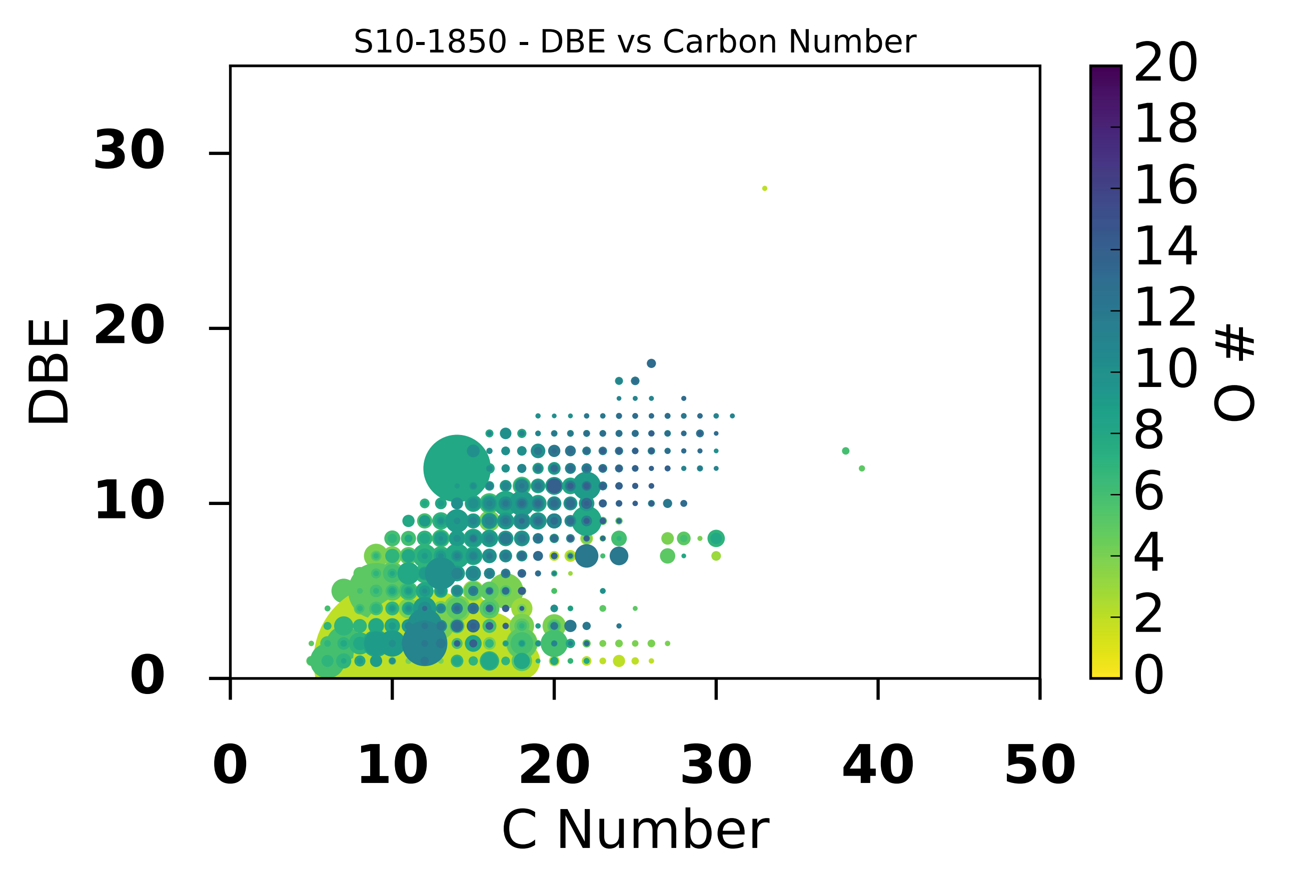
S10-1850 - DBE vs Carbon Number
DBE
C Number
O #
0
10
20
30
40
50
0
2
4
6
8
10
12
14
16
18
20

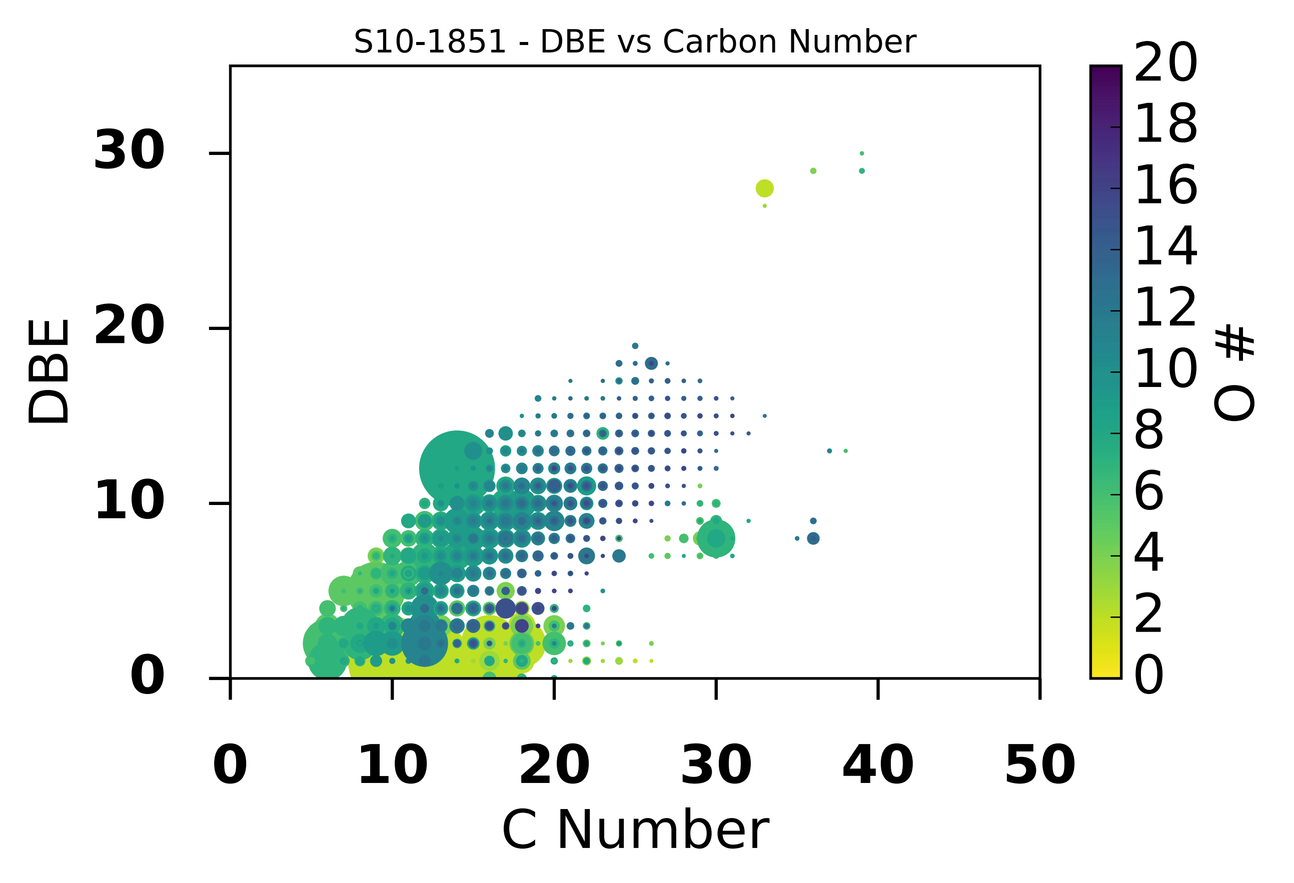
S10-1851 - DBE vs Carbon Number
DBE
C Number
O #
0
10
20
30
40
50
0
10
20
30
0
2
4
6
8
10
12
14
16
18
20

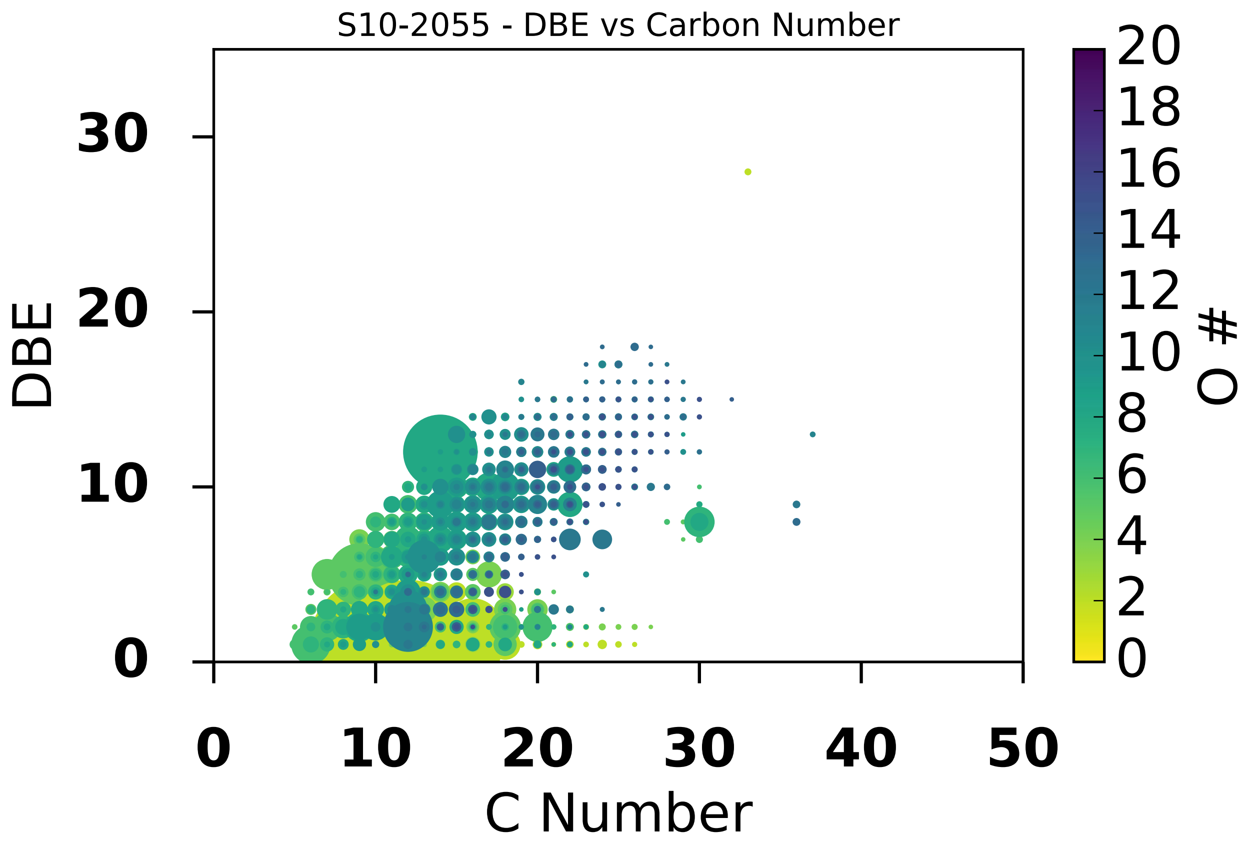
S10-2055 - DBE vs Carbon Number
DBE
0
10
20
30
C Number
0
10
20
30
40
50
O #
0
2
4
6
8
10
12
14
16
18
20

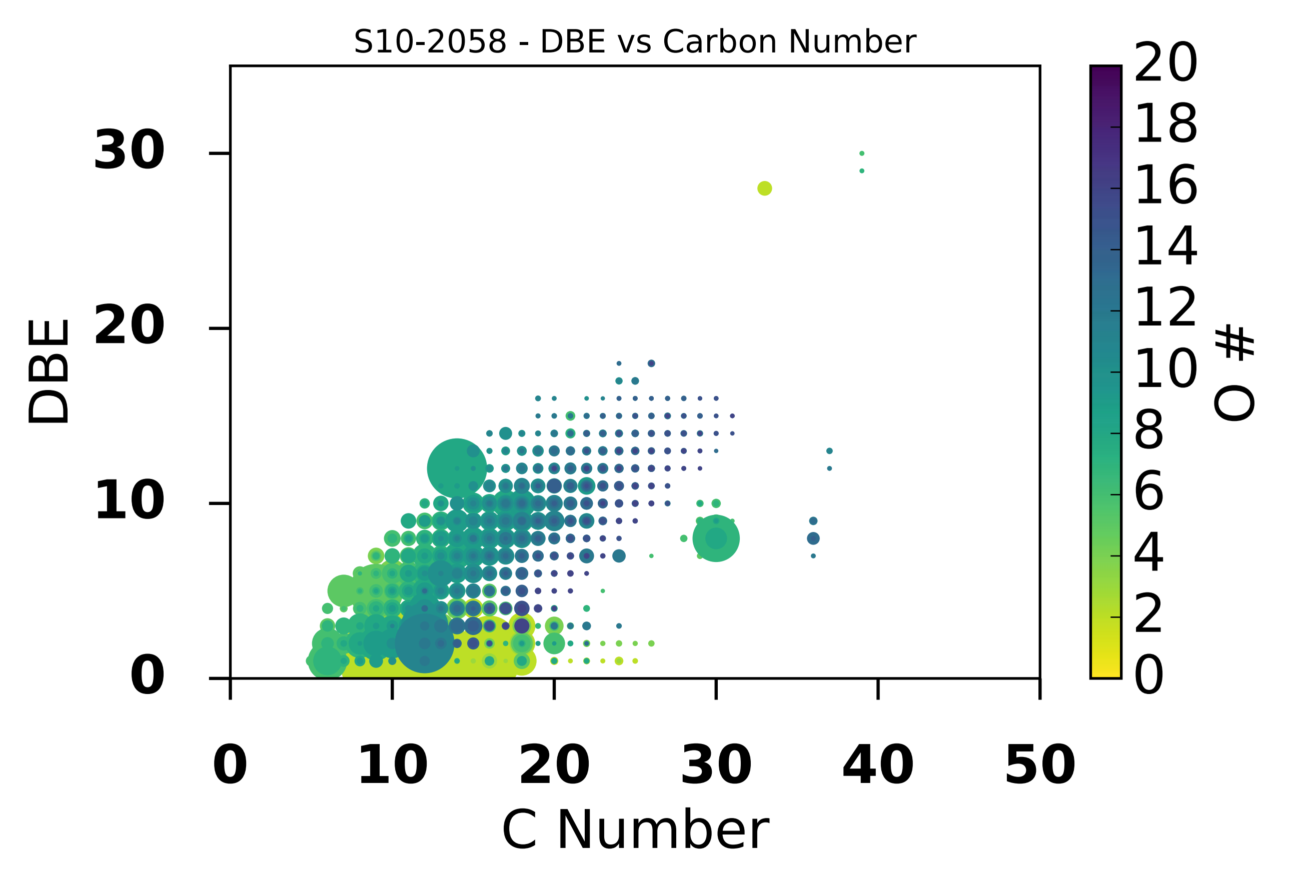
S10-2058 - DBE vs Carbon Number
DBE
0
10
20
30
C Number
0
10
20
30
40
50
O #
0
2
4
6
8
10
12
14
16
18
20

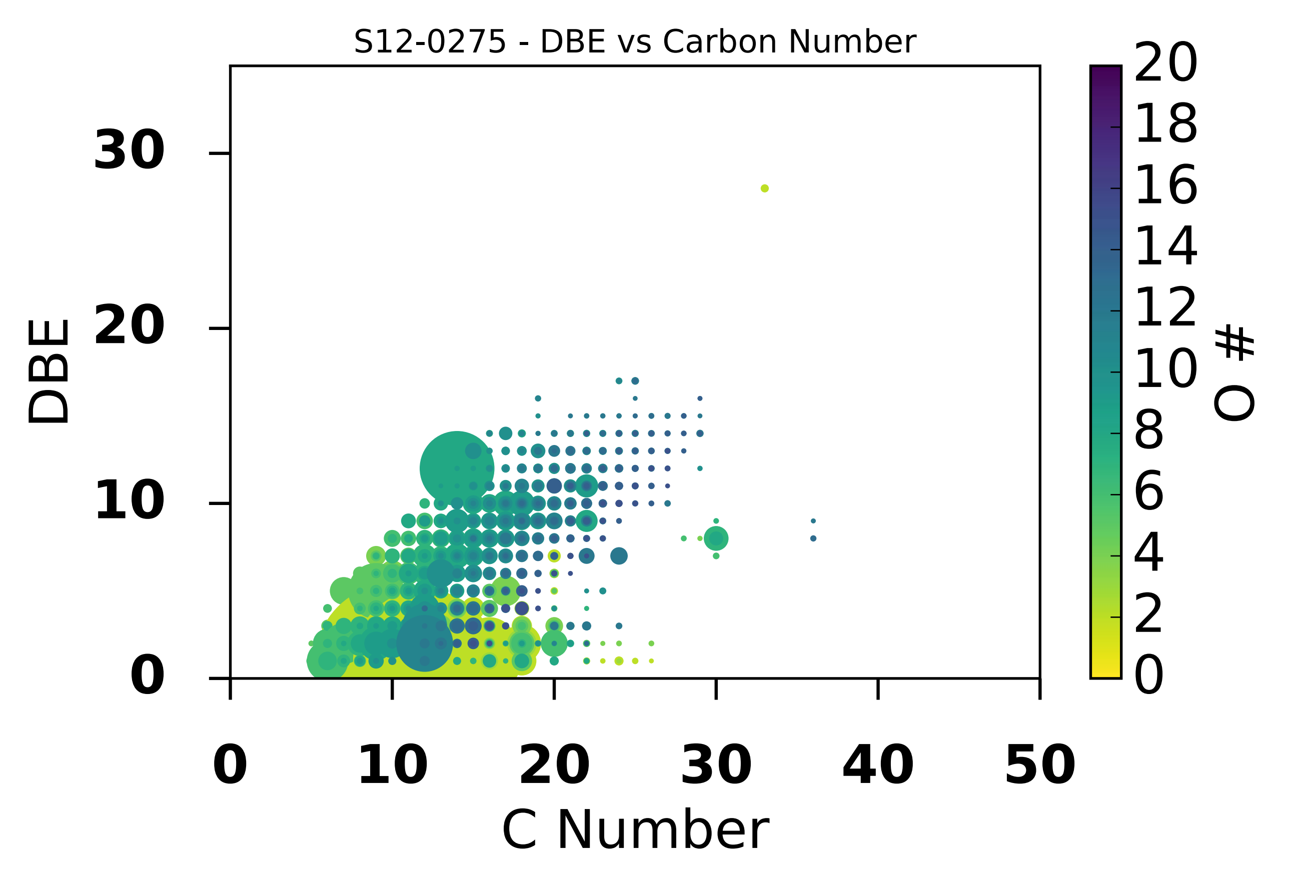
S12-0275 - DBE vs Carbon Number
DBE
30
20
10
0
0
10
20
30
40
50
C Number
O #
20
18
16
14
12
10
8
6
4
2
0

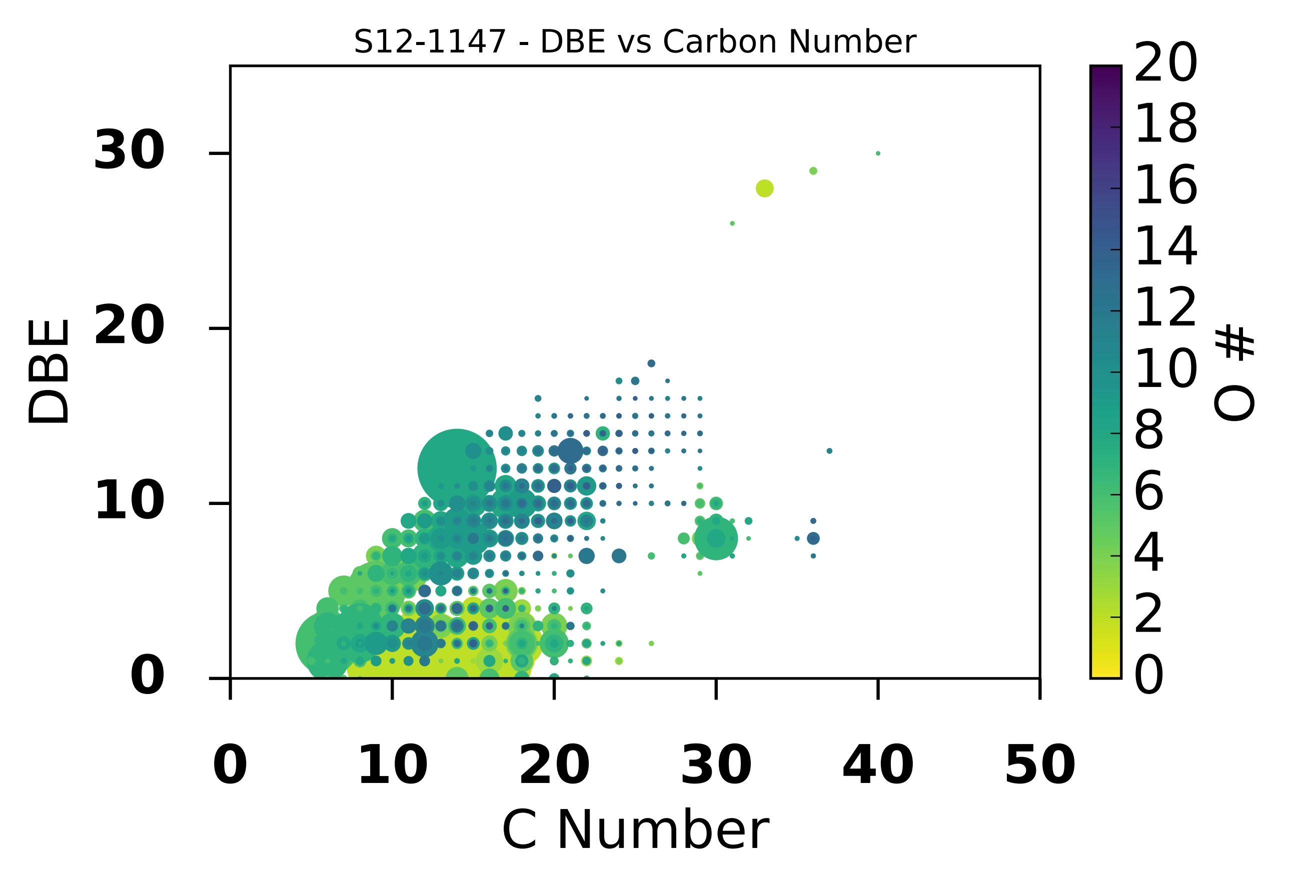

S12-1147 - DBE vs Carbon Number
DBE
30
20
10
0
0
10
20
30
40
50
C Number
O #
20
18
16
14
12
10
8
6
4
2
0

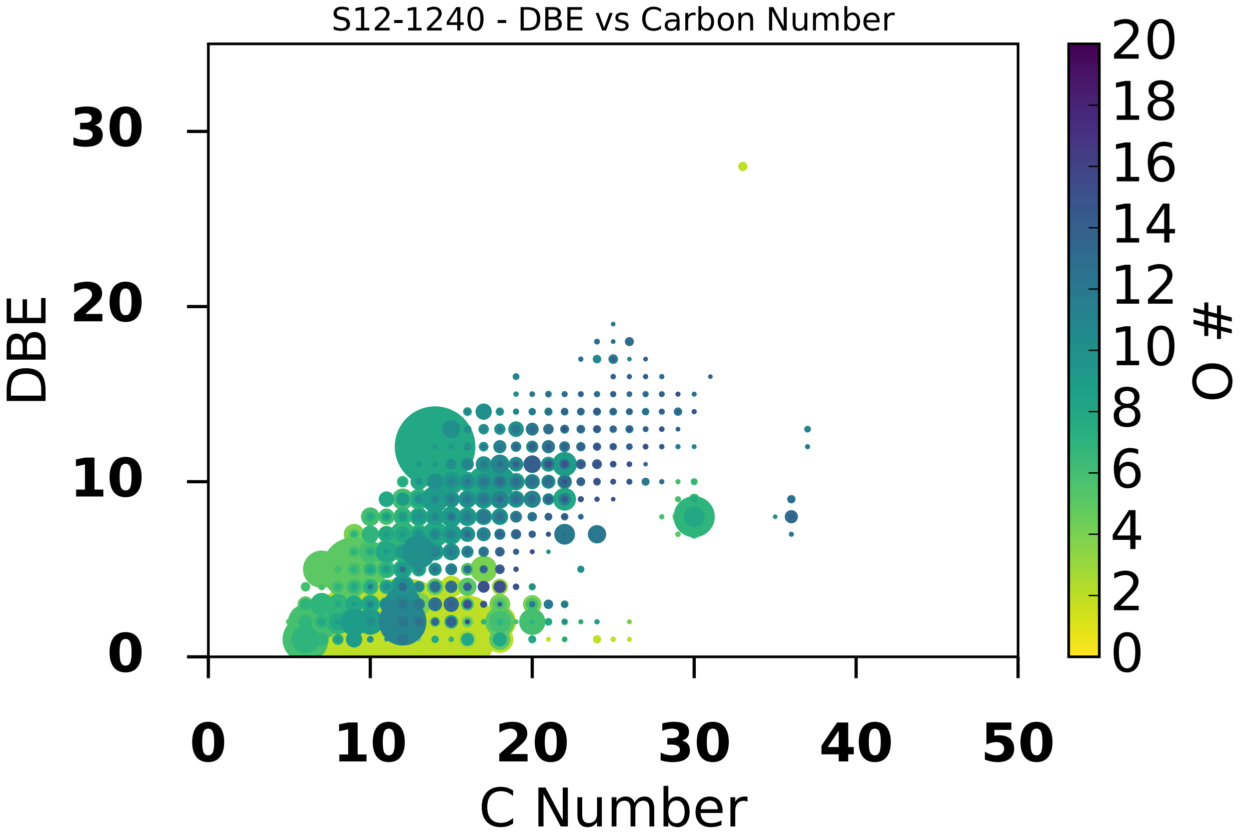

S12-1240 - DBE vs Carbon Number
DBE
C Number
O #
0
10
20
30
40
50
2
4
6
8
12
14
16
18

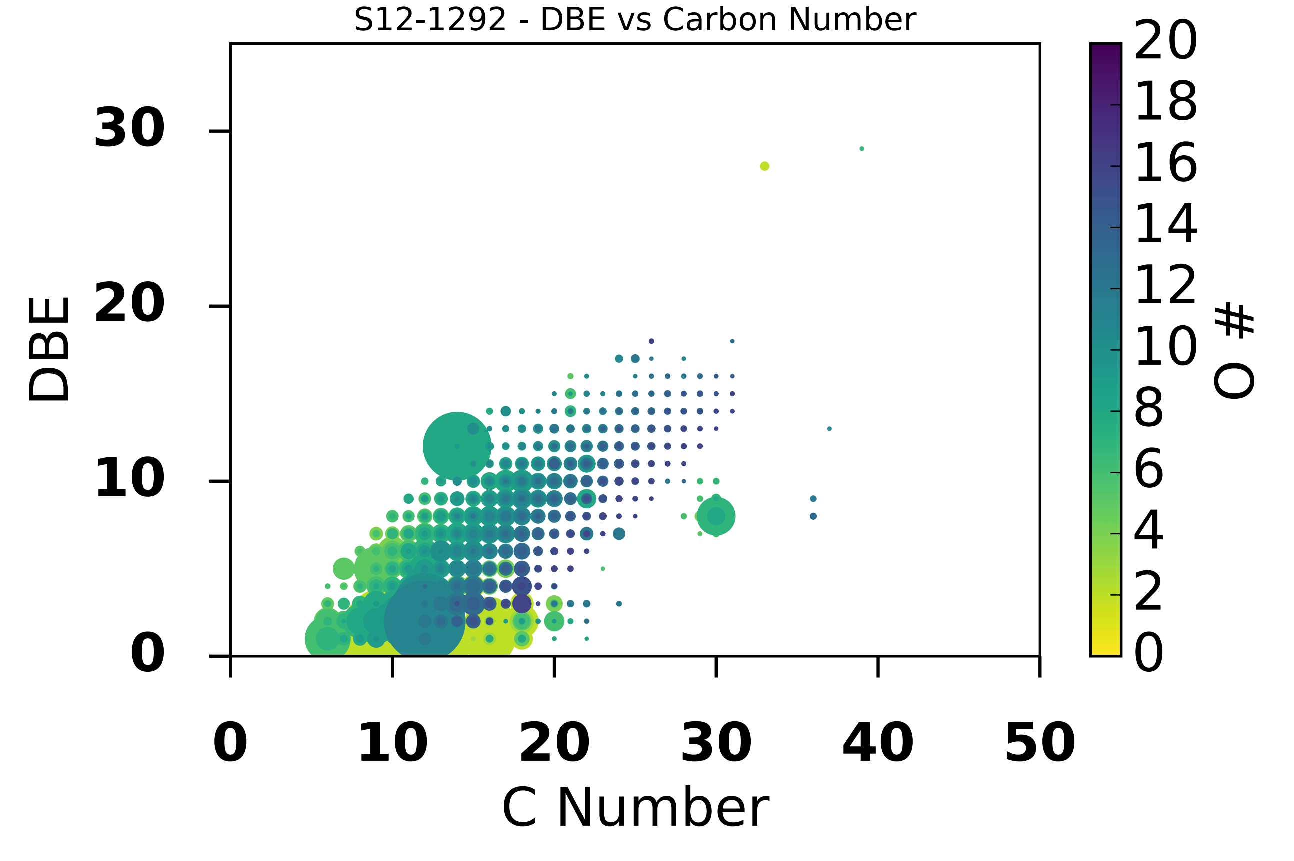

S12-1292 - DBE vs Carbon Number
DBE
30
20
10
0
0
10
20
30
40
50
C Number
O #
20
18
16
14
12
10
8
6
4
2
0

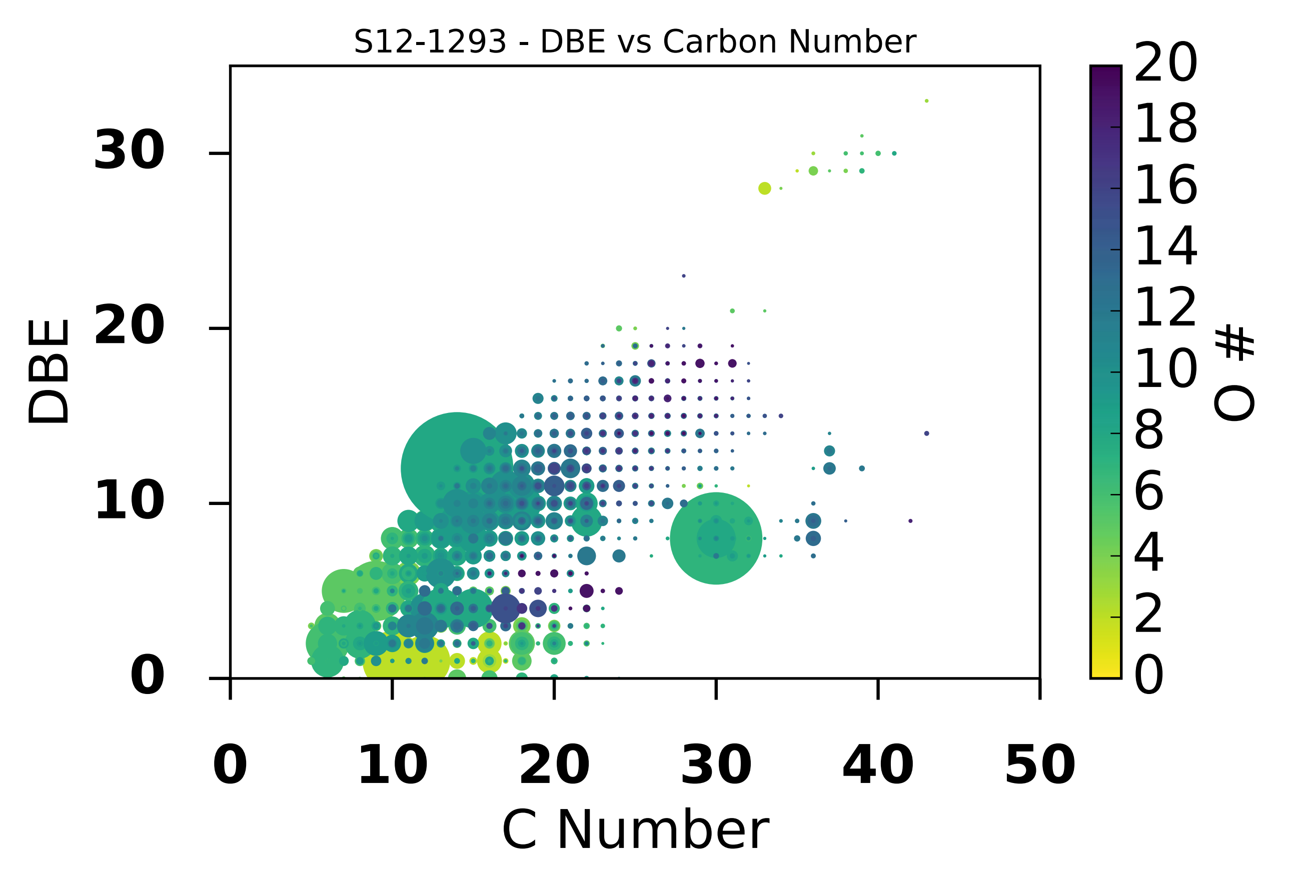
S12-1293 - DBE vs Carbon Number
DBE
0
10
20
30
C Number
0
10
20
30
40
50
O #
0
2
4
6
8
10
12
14
16
18
20

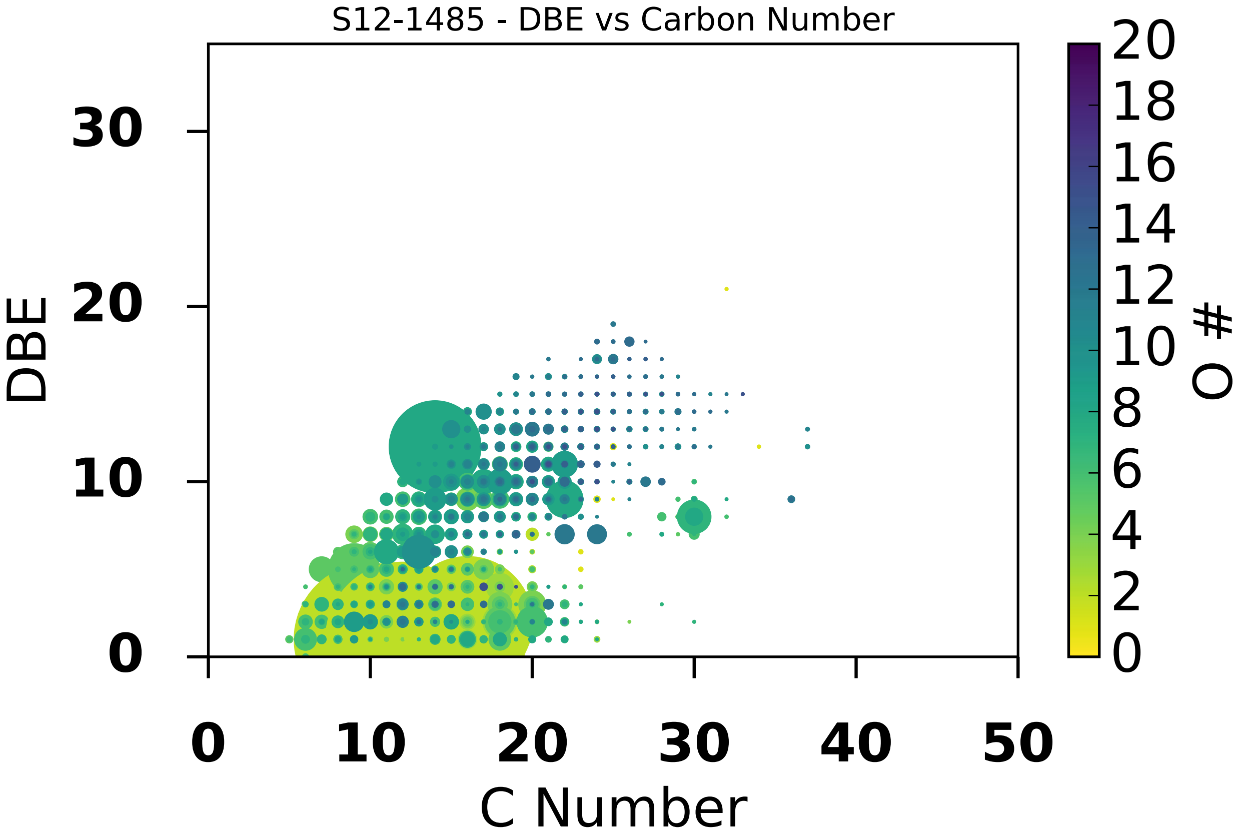
S12-1485 - DBE vs Carbon Number
DBE
0
10
20
30
C Number
0
10
20
30
40
50
O #
0
2
4
6
8
10
12
14
16
18
20

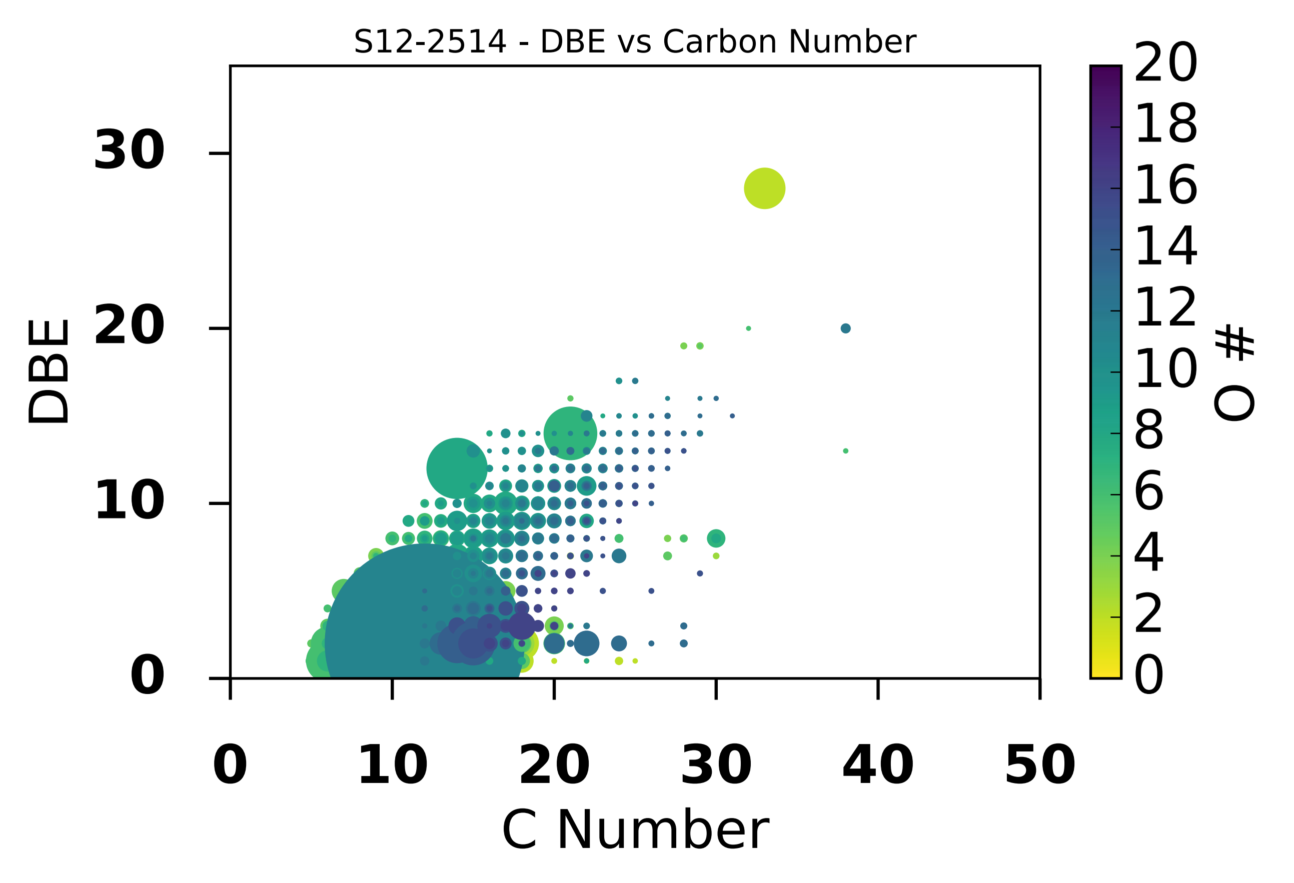
S12-2514 - DBE vs Carbon Number
DBE
30
20
10
0
0
10
20
30
40
50
C Number
O #
20
18
16
14
12
10
8
6
4
2
0

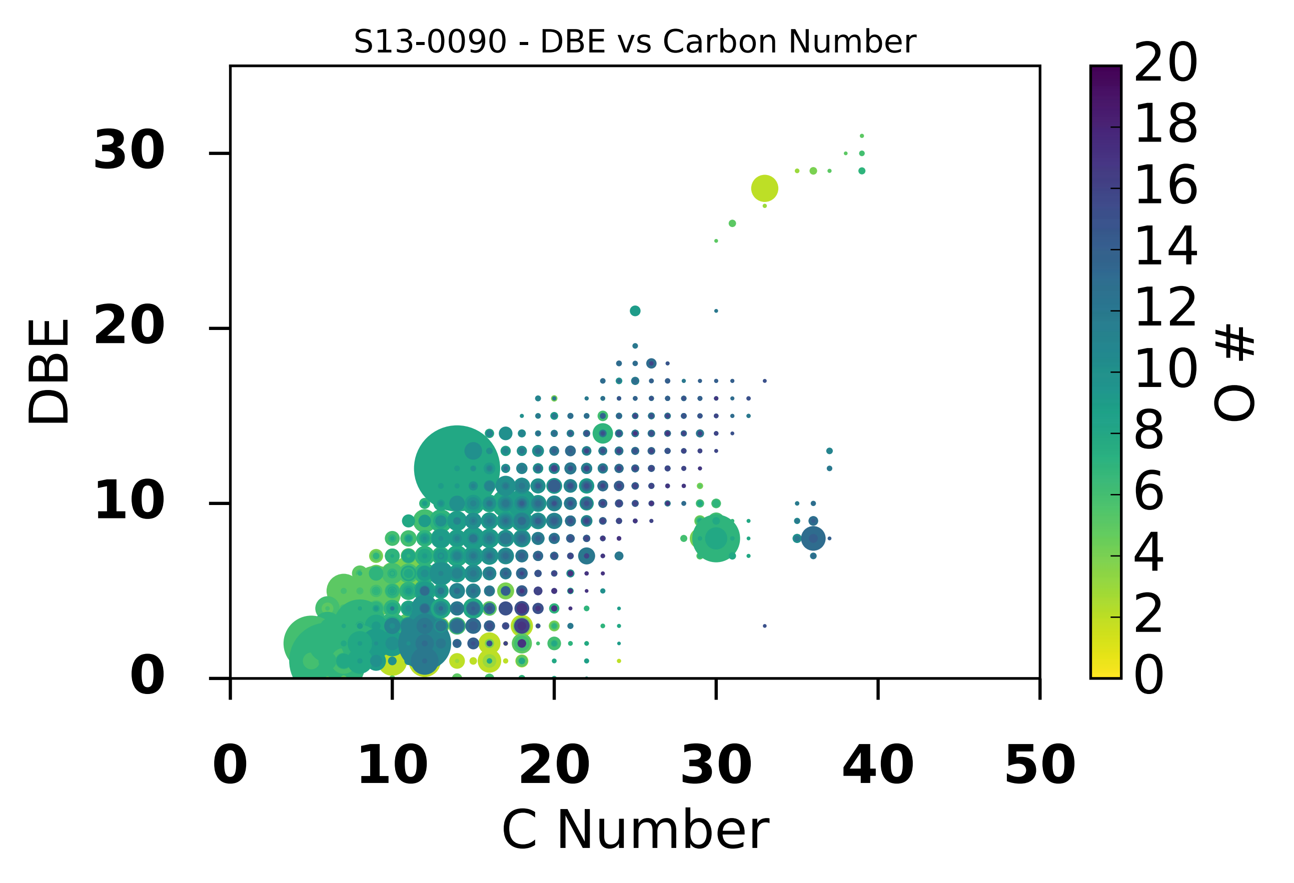

S13-0090 - DBE vs Carbon Number
DBE
30
20
10
0
0
10
20
30
40
50
C Number
20
18
16
14
12
10
8
6
4
2
0
O #

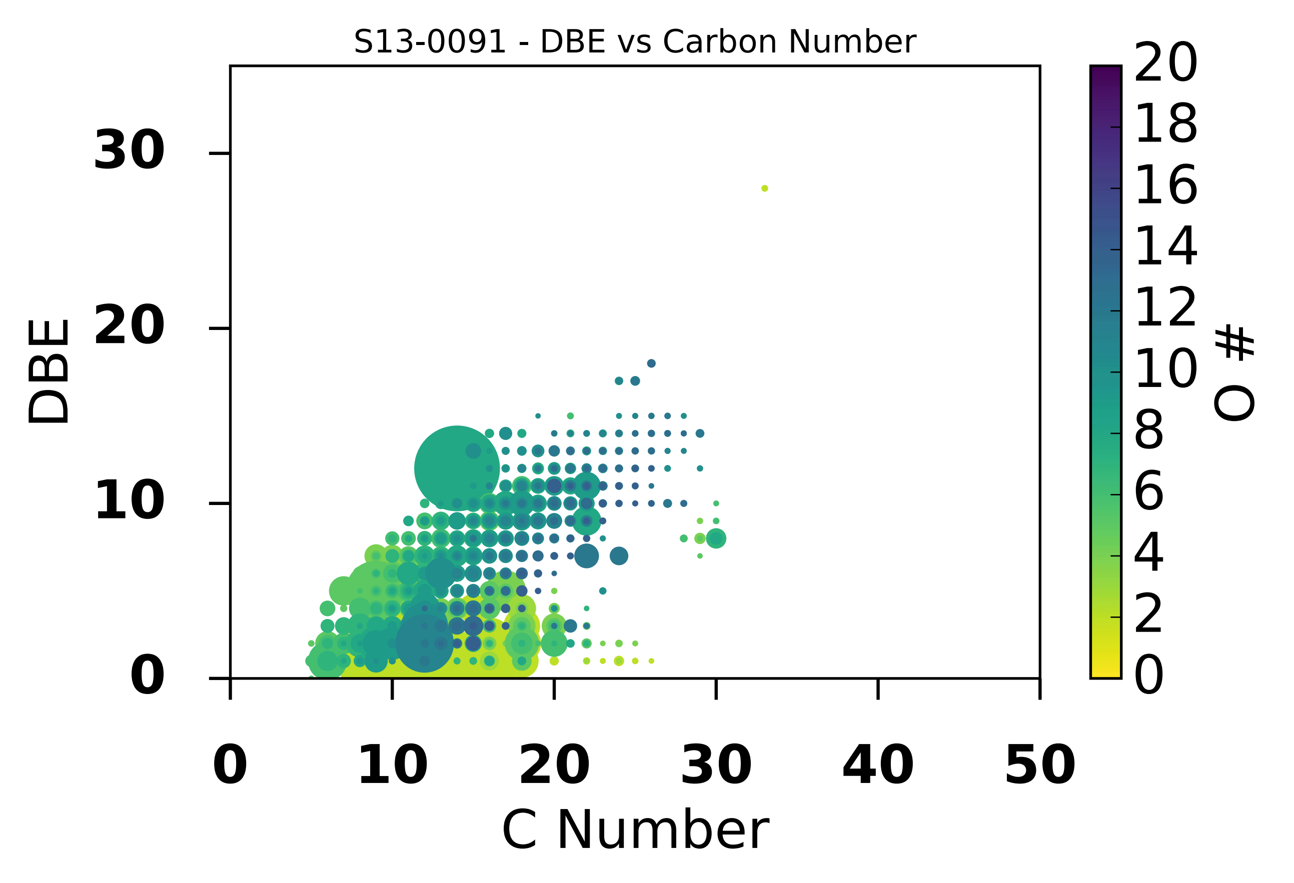
S13-0091 - DBE vs Carbon Number
DBE
30
20
10
0
0
10
20
30
40
50
C Number
O #
20
18
16
14
12
10
8
6
4
2
0

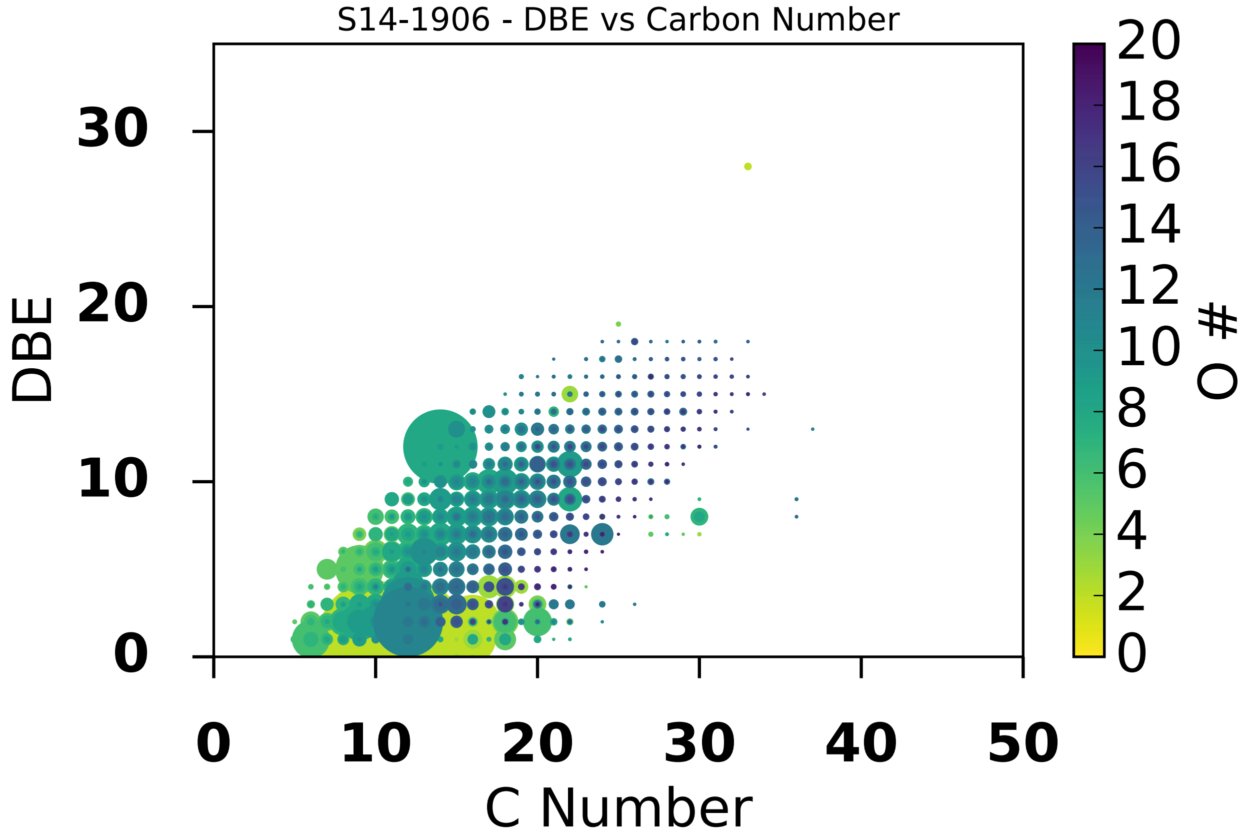

S14-1906 - DBE vs Carbon Number
DBE
0
10
20
30
C Number
0
10
20
30
40
50
O #
0
2
4
6
8
10
12
14
16
18
20

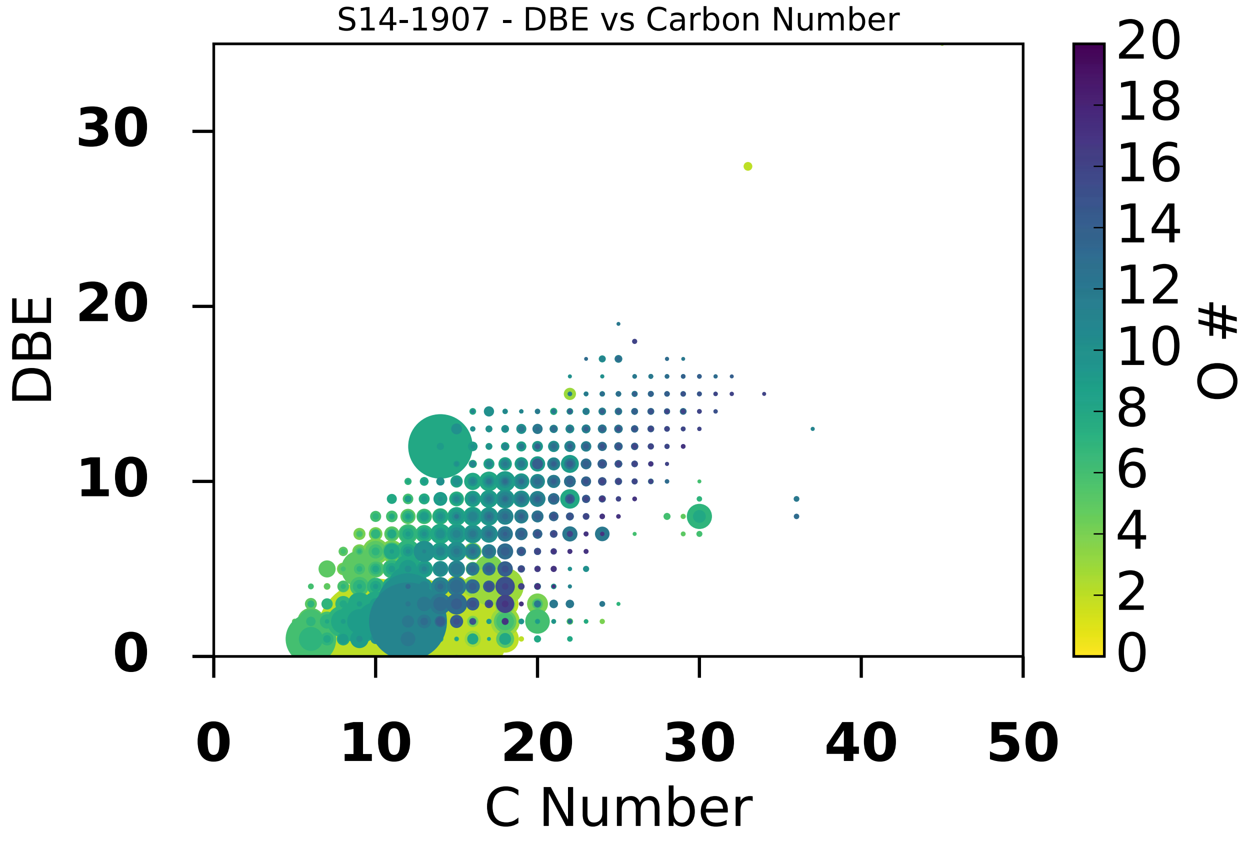
S14-1907 - DBE vs Carbon Number
DBE
30
20
10
0
0
10
20
30
40
50
C Number
20
18
16
14
12
10
8
6
4
2
0
O #

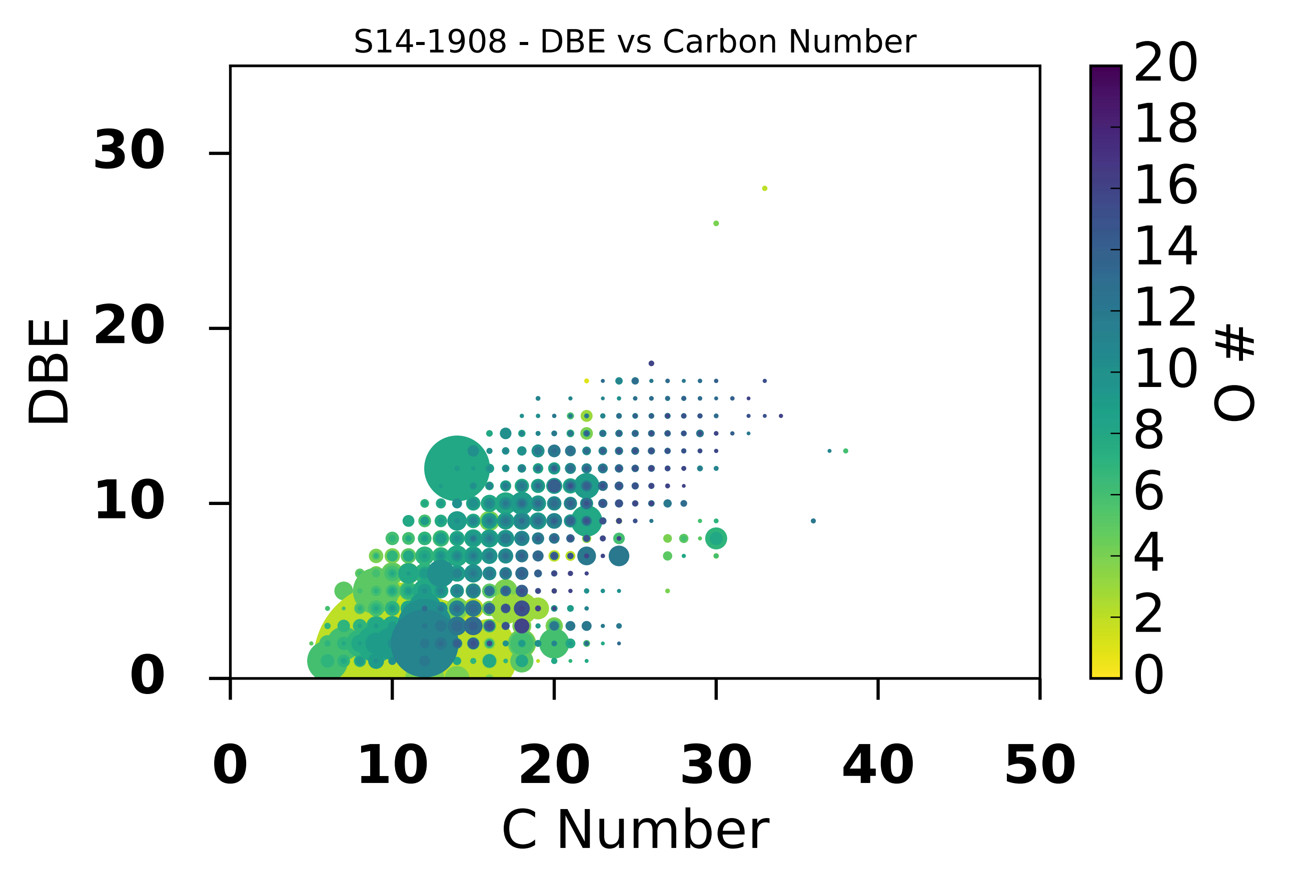

S14-1908 - DBE vs Carbon Number
DBE
0
10
20
30
C Number
0
10
20
30
40
50
O #
0
2
4
6
8
10
12
14
16
18
20

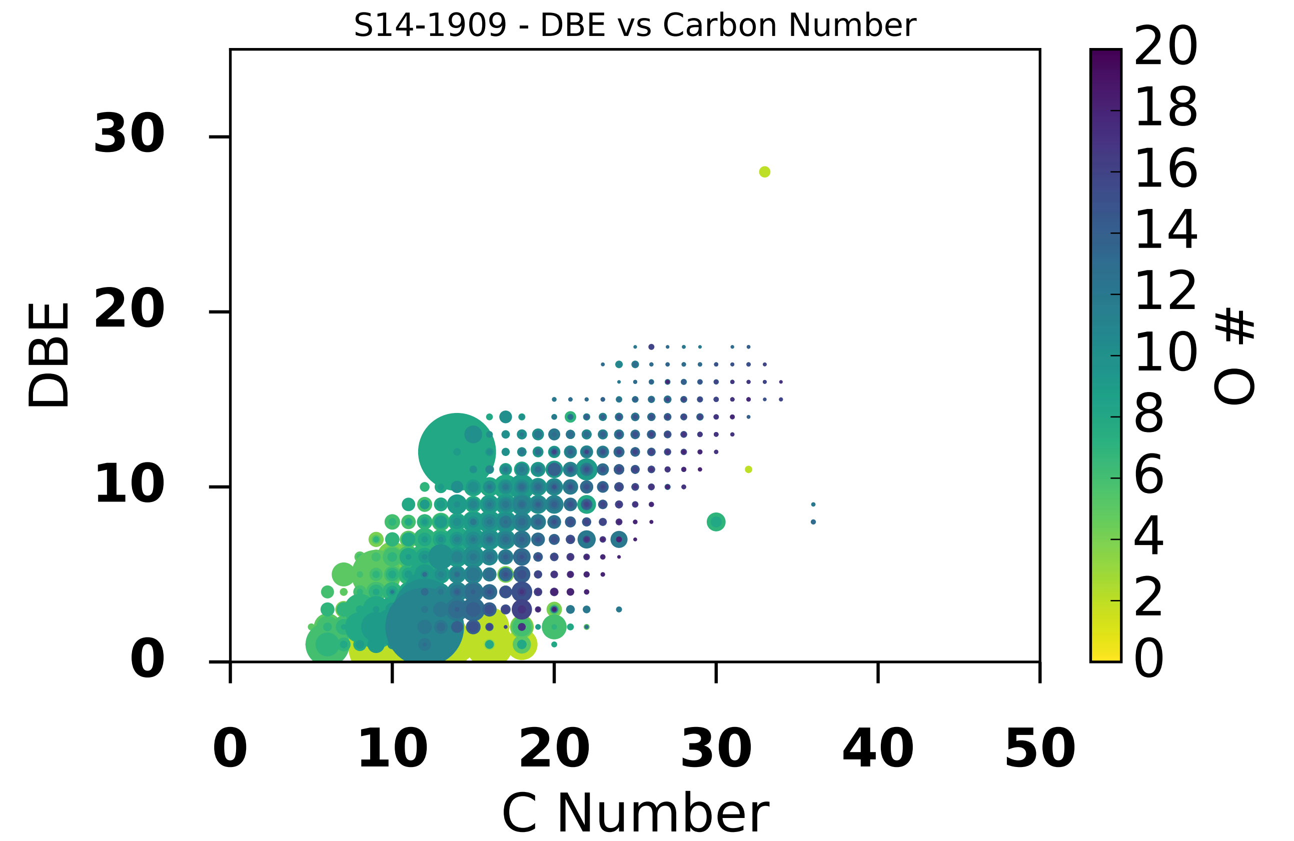

S14-1909 - DBE vs Carbon Number
DBE
30
20
10
0
0
10
20
30
40
50
C Number
O #
20
18
16
14
12
10
8
6
4
2
0

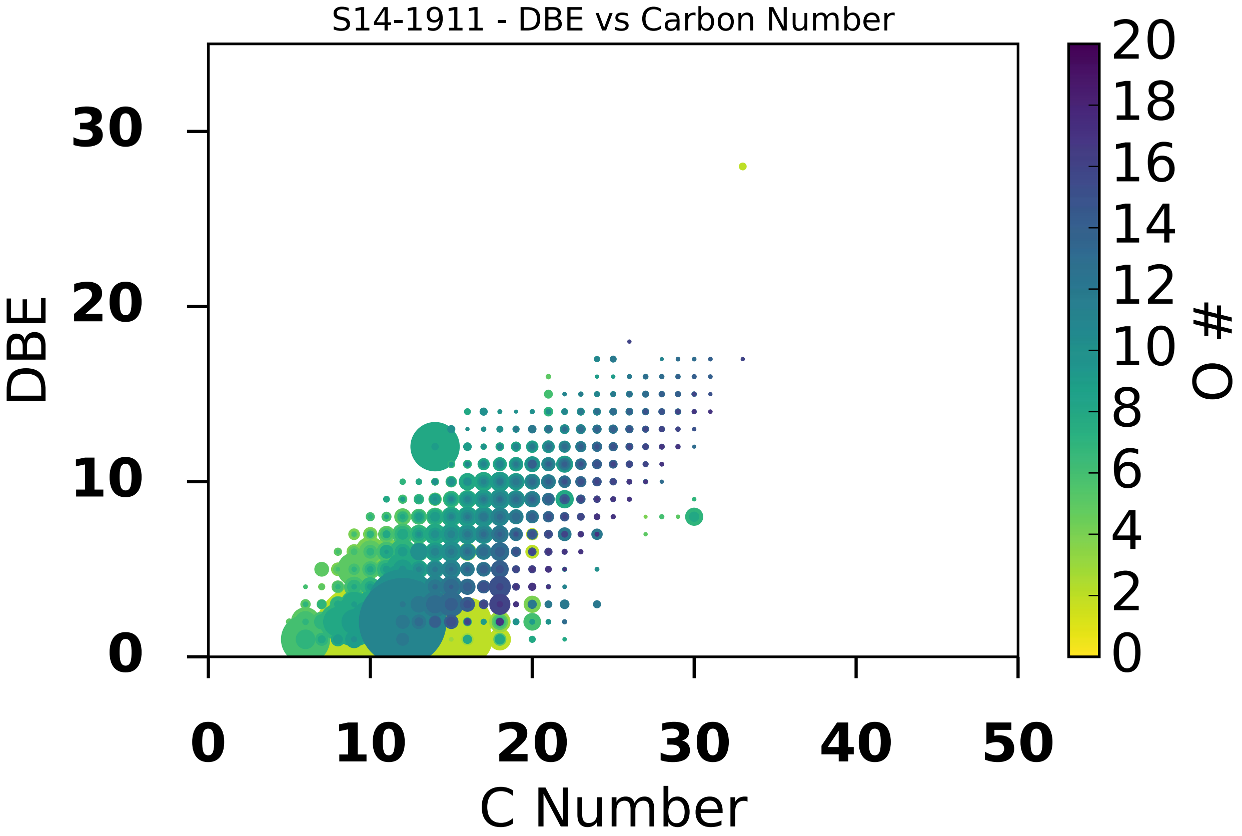

S14-1911 - DBE vs Carbon Number
DBE
C Number
O #
0
10
20
30
40
50
0
2
4
6
8
10
12
14
16
18
20

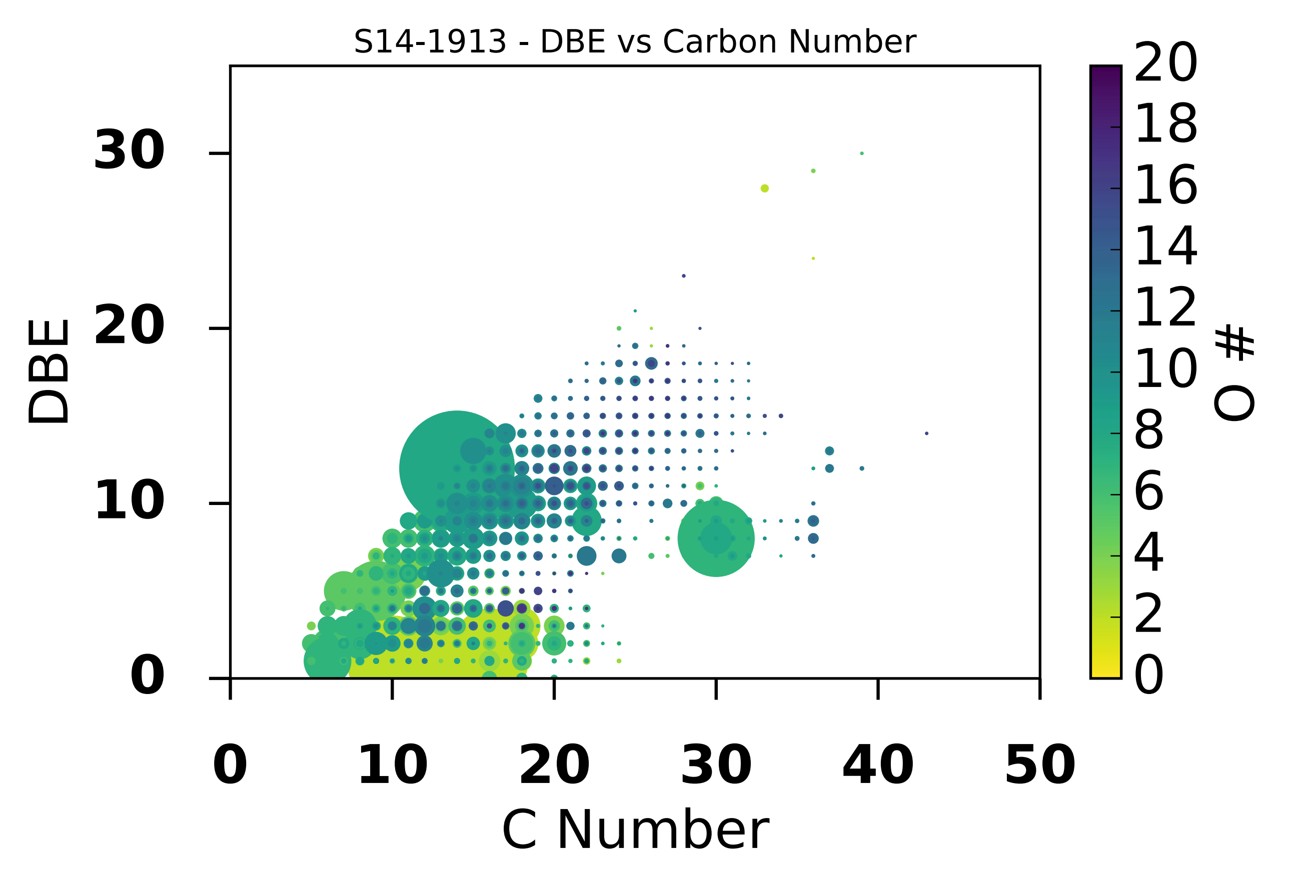

S14-1913 - DBE vs Carbon Number
DBE
0
10
20
30
C Number
0
10
20
30
40
50
O #
0
2
4
6
8
10
12
14
16
18
20

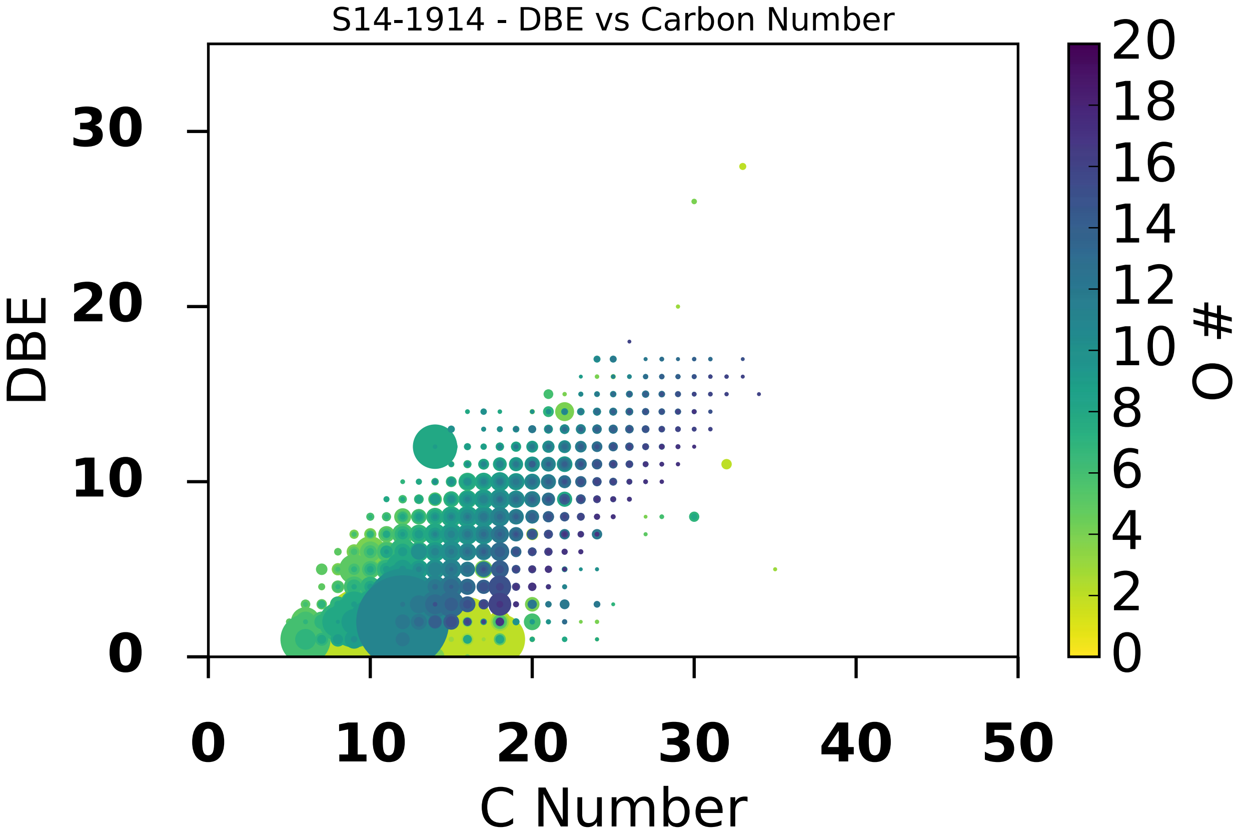

S14-1914 - DBE vs Carbon Number
DBE
0
10
20
30
C Number
0
10
20
30
40
50
O #
0
2
4
6
8
10
12
14
16
18
20

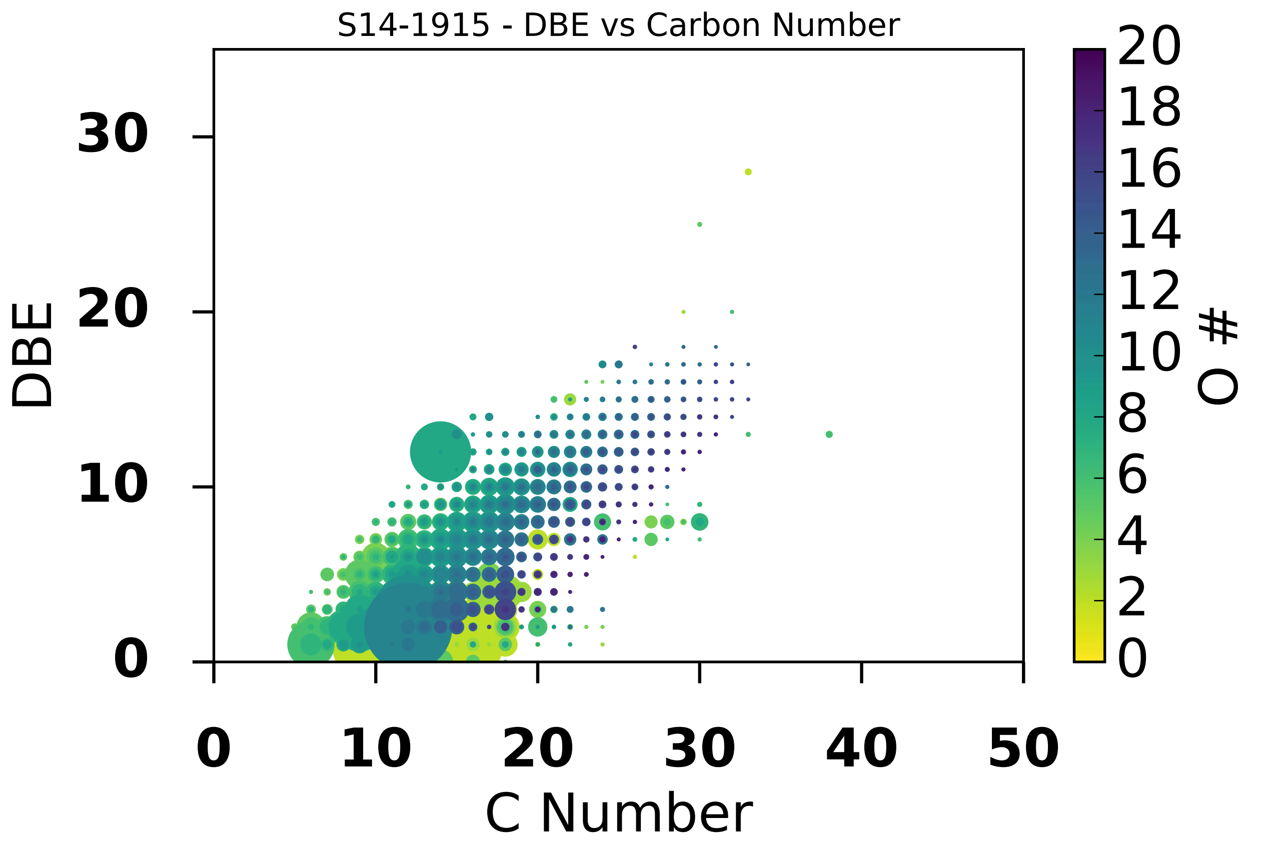

S14-1915 - DBE vs Carbon Number
DBE
0
10
20
30
C Number
0
10
20
30
40
50
O #
0
2
4
6
8
10
12
14
16
18
20

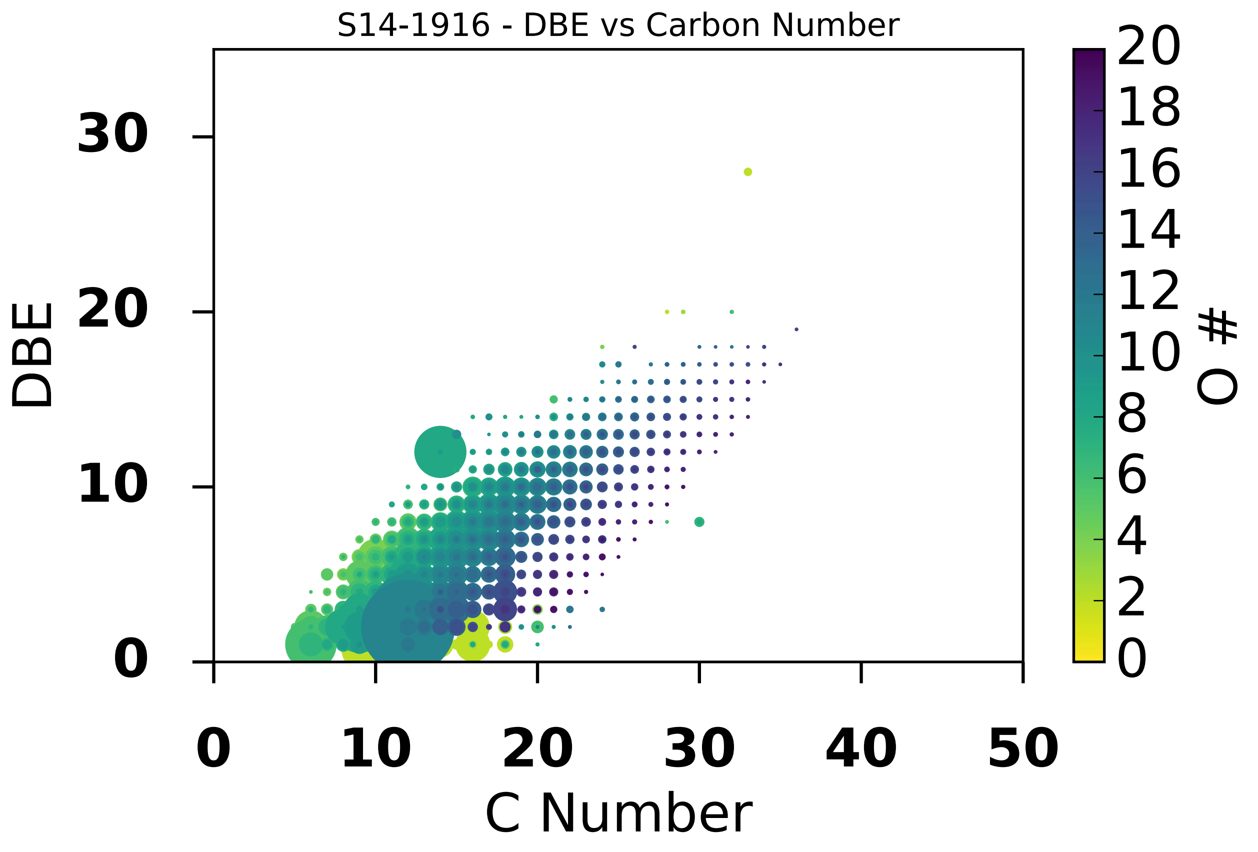
S14-1916 - DBE vs Carbon Number
DBE
0
10
20
30
C Number
0
10
20
30
40
50
O #
0
2
4
6
8
10
12
14
16
18
20

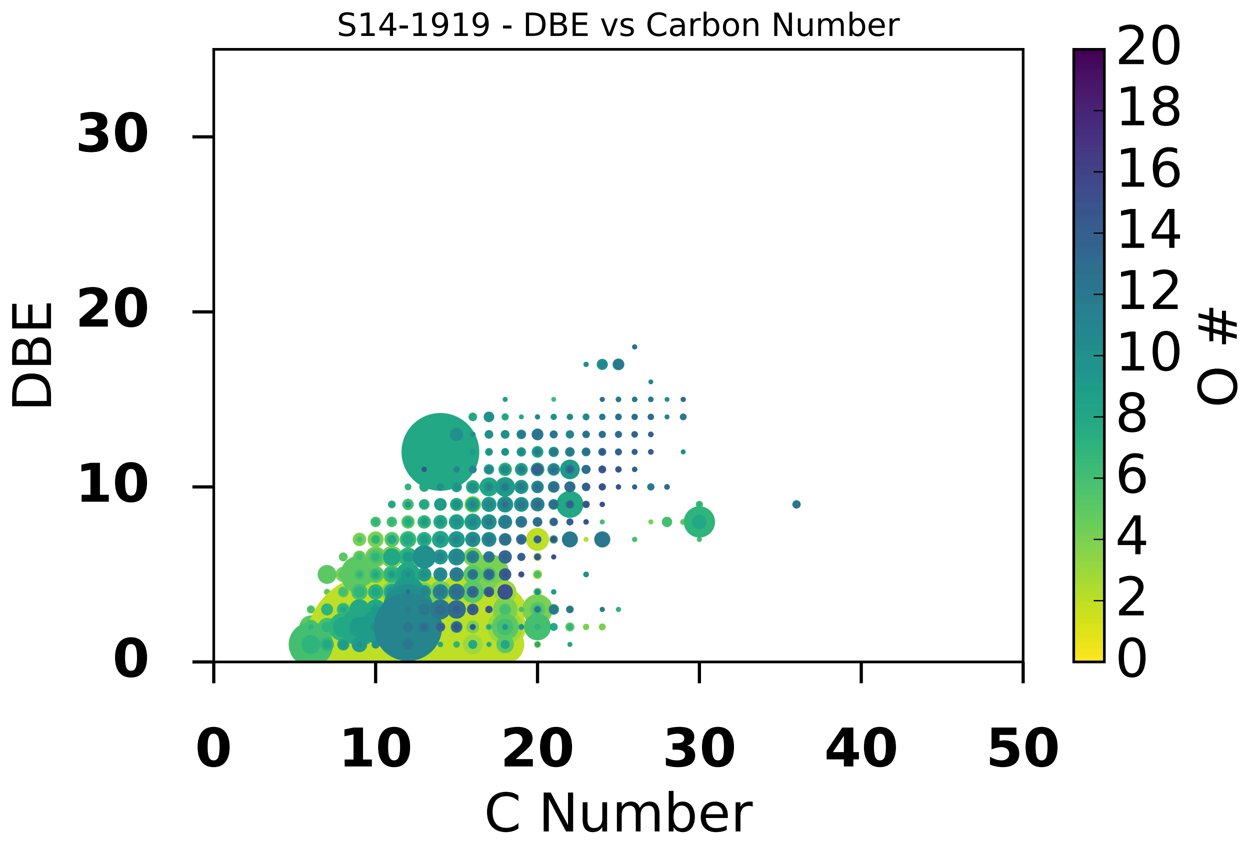

S14-1919 - DBE vs Carbon Number
DBE
0
10
20
30
C Number
0
10
20
30
40
50
O #
0
2
4
6
8
10
12
14
16
18
20

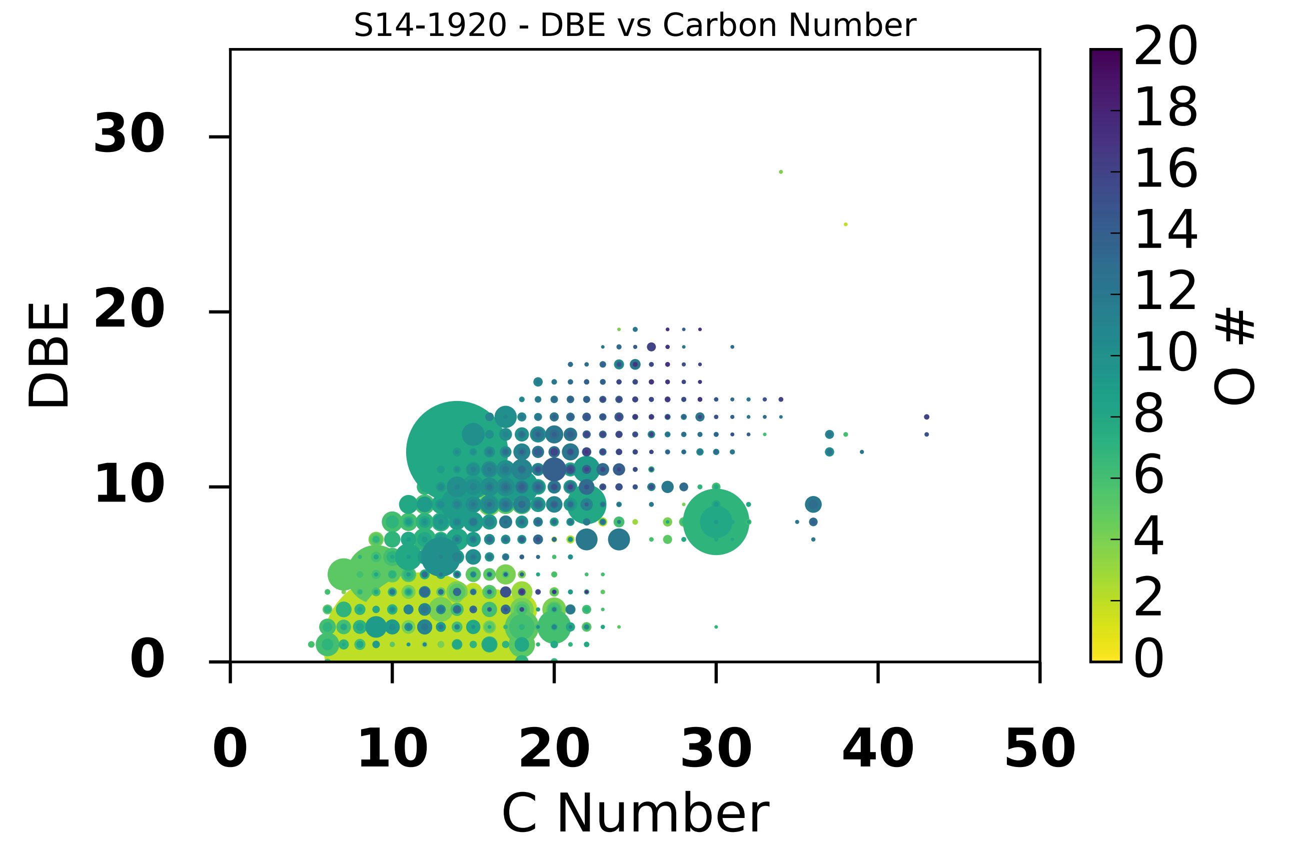
S14-1920 - DBE vs Carbon Number
DBE
0
10
20
30
C Number
0
10
20
30
40
50
O #
0
2
4
6
8
10
12
14
16
18
20

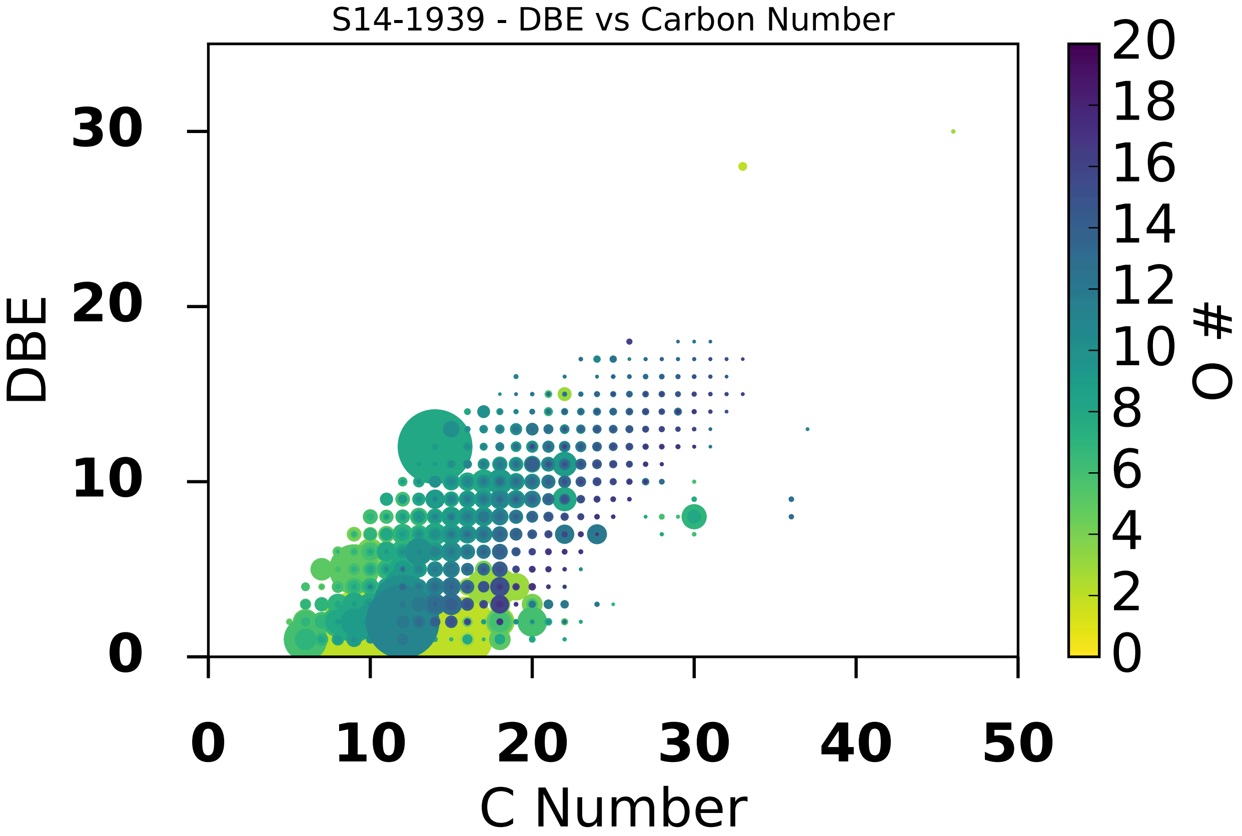
S14-1939 - DBE vs Carbon Number
DBE
0
10
20
30
C Number
0
10
20
30
40
50
O #
0
2
4
6
8
10
12
14
16
18
20

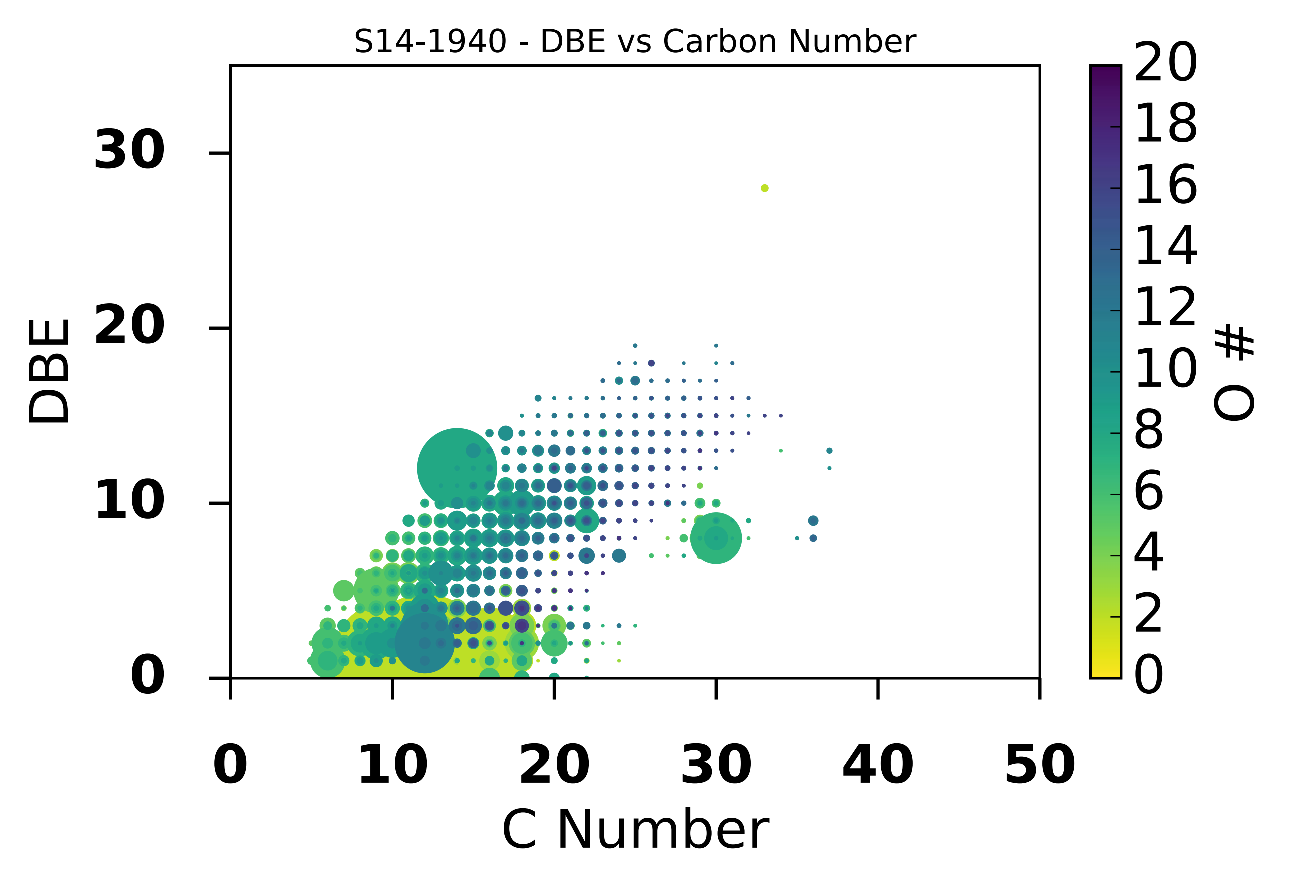
S14-1940 - DBE vs Carbon Number
DBE
30
20
10
0
0
10
20
30
40
50
C Number
O #
20
18
16
14
12
10
8
6
4
2
0

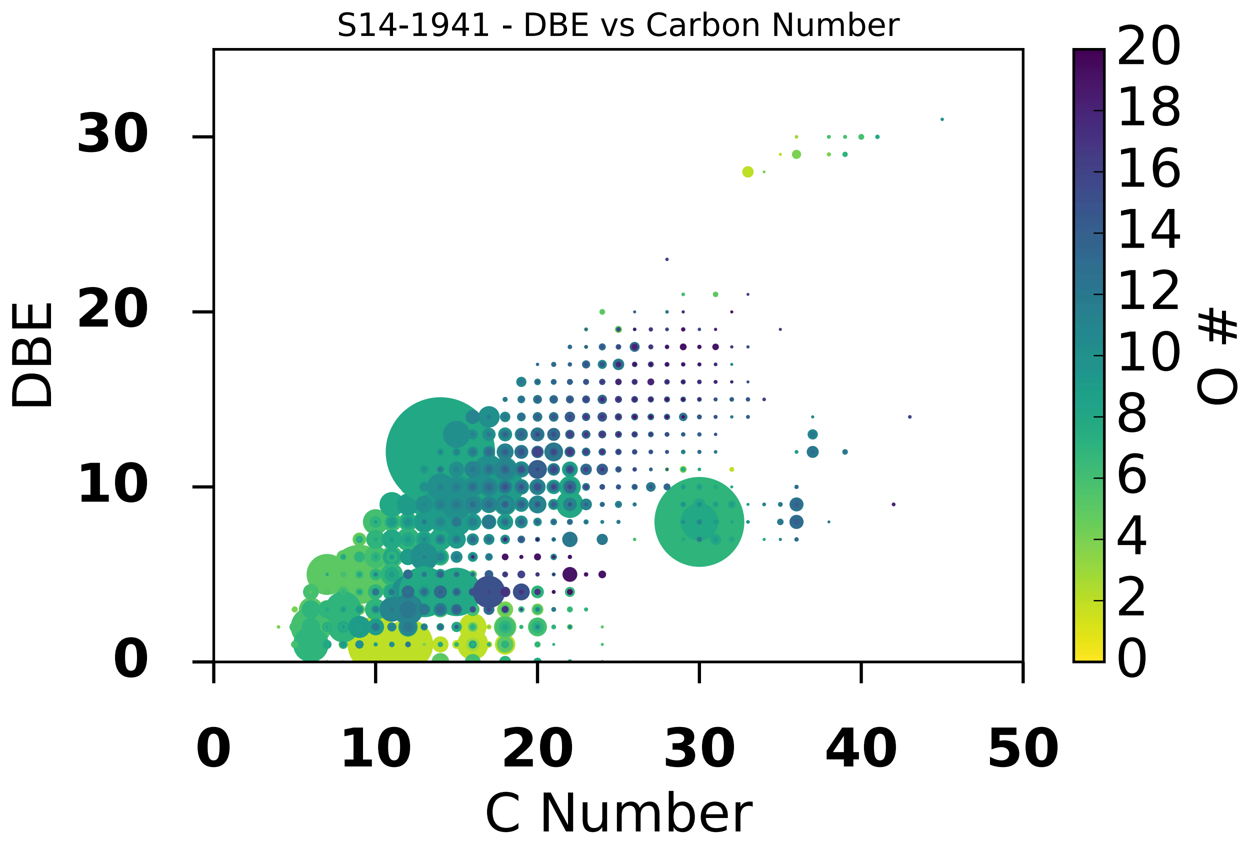
S14-1941 - DBE vs Carbon Number
DBE
0
10
20
30
C Number
0
10
20
30
40
50
O #
0
2
4
6
8
10
12
14
16
18
20

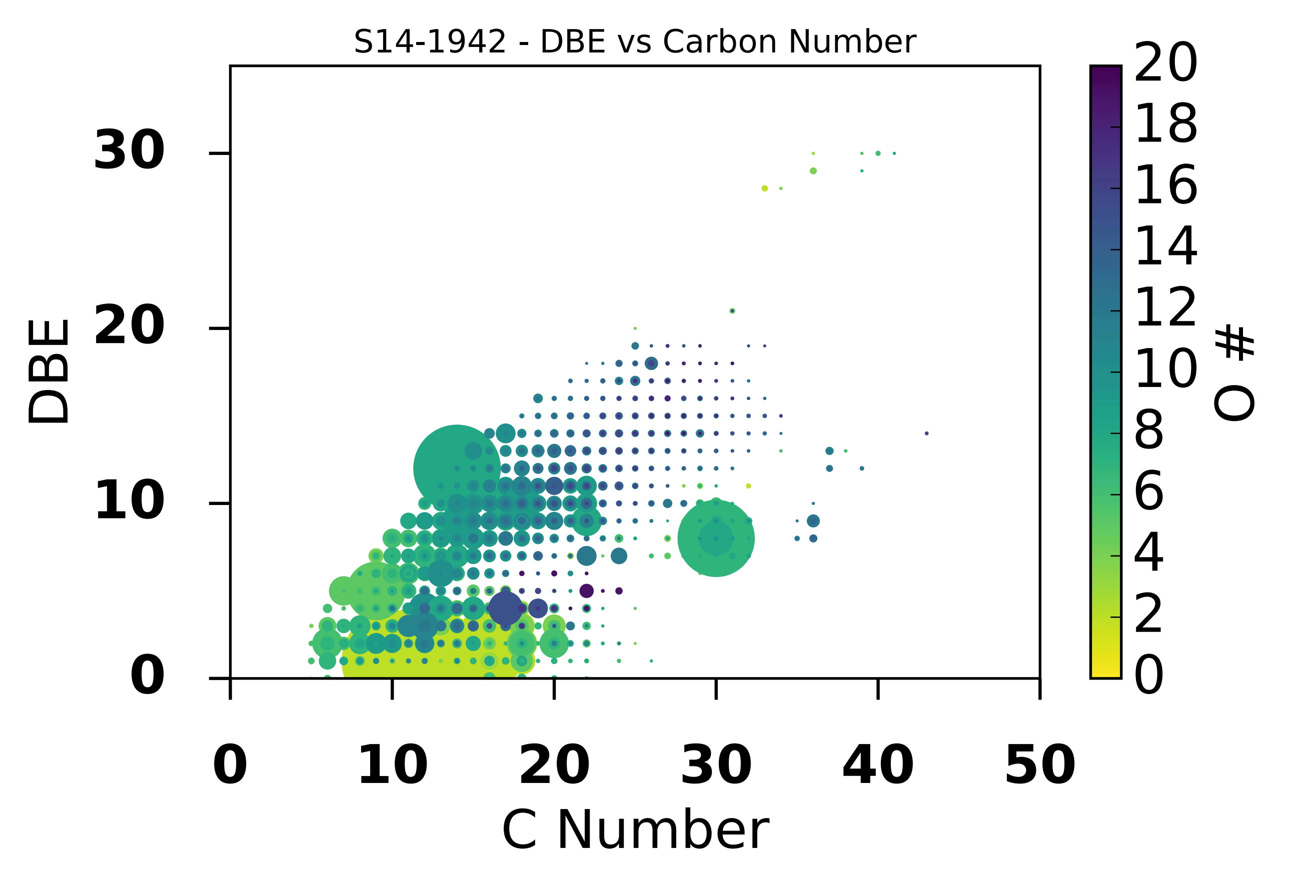

S14-1942 - DBE vs Carbon Number
DBE
0
10
20
30
C Number
0
10
20
30
40
50
O #
0
2
4
6
8
10
12
14
16
18
20

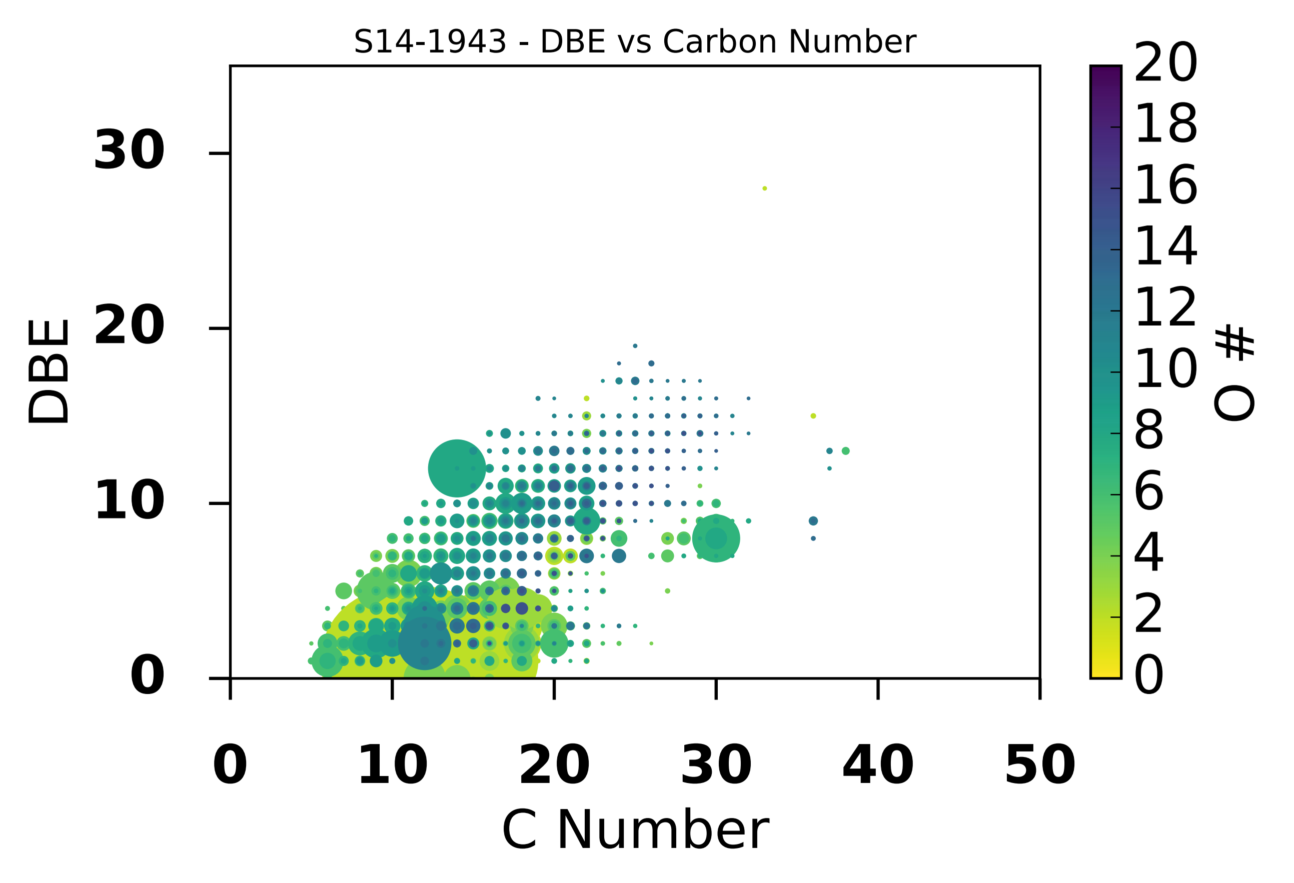

S14-1943 - DBE vs Carbon Number
DBE
30
20
10
0
0
10
20
30
40
50
C Number
20
18
16
14
12
10
8
6
4
2
0
O #

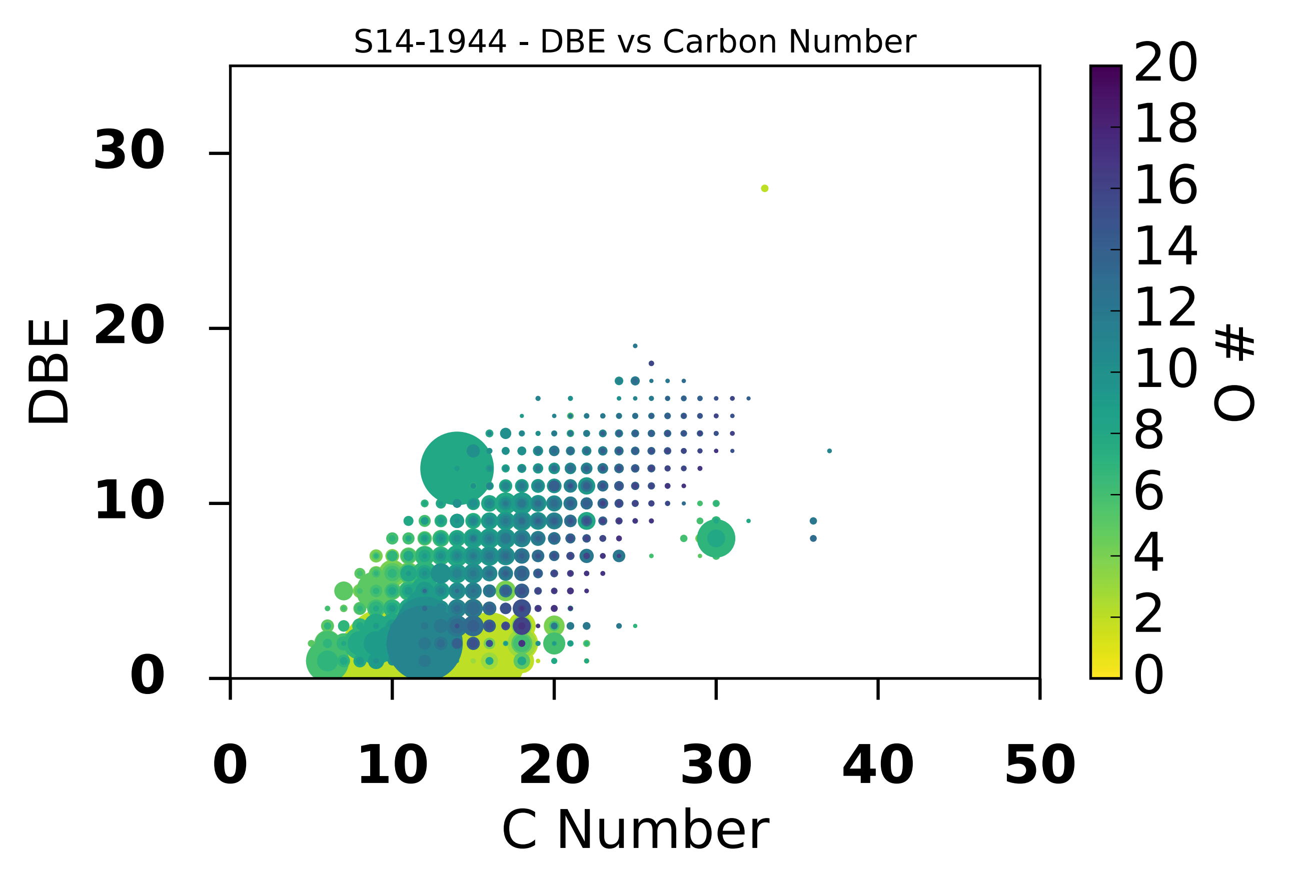

S14-1944 - DBE vs Carbon Number
DBE
C Number
O #
0
10
20
30
40
50

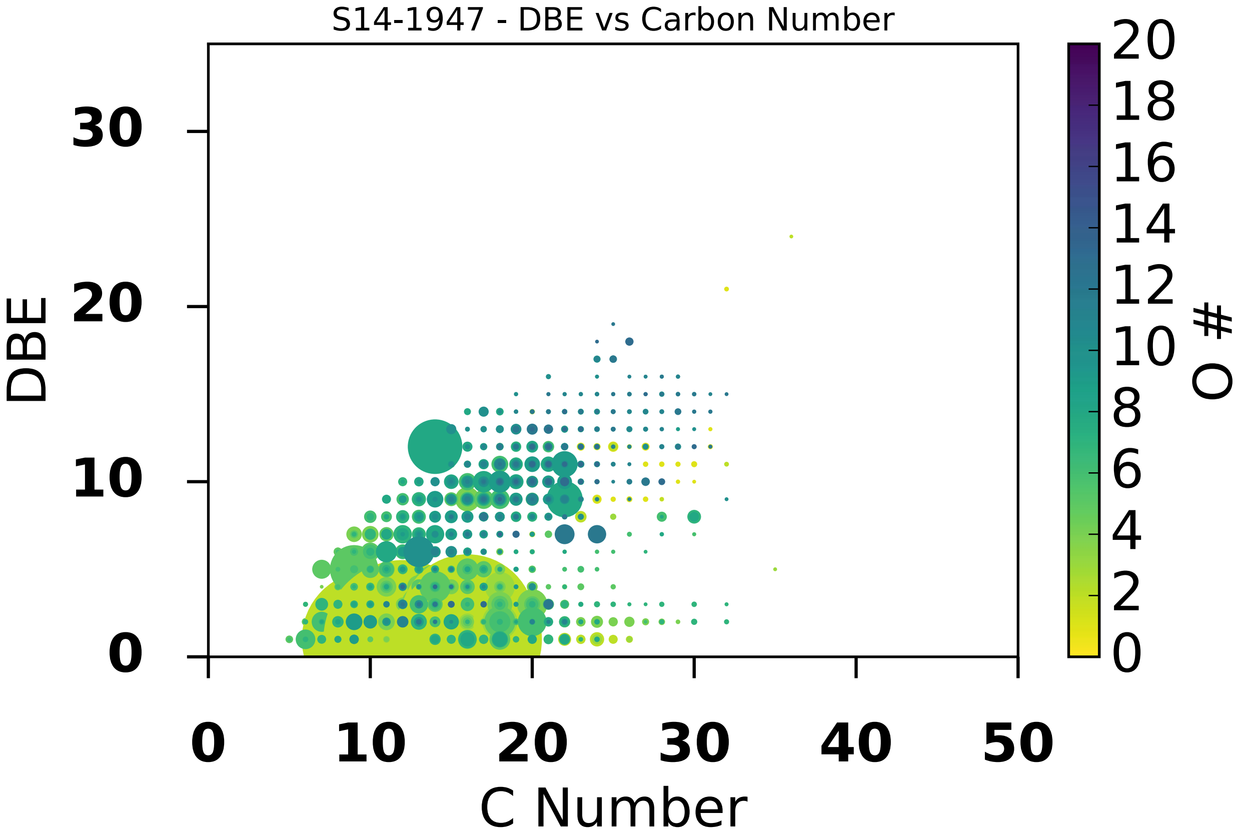

S14-1947 - DBE vs Carbon Number
DBE
30
20
10
0
0
10
20
30
40
50
C Number
O #
20
18
16
14
12
10
8
6
4
2
0

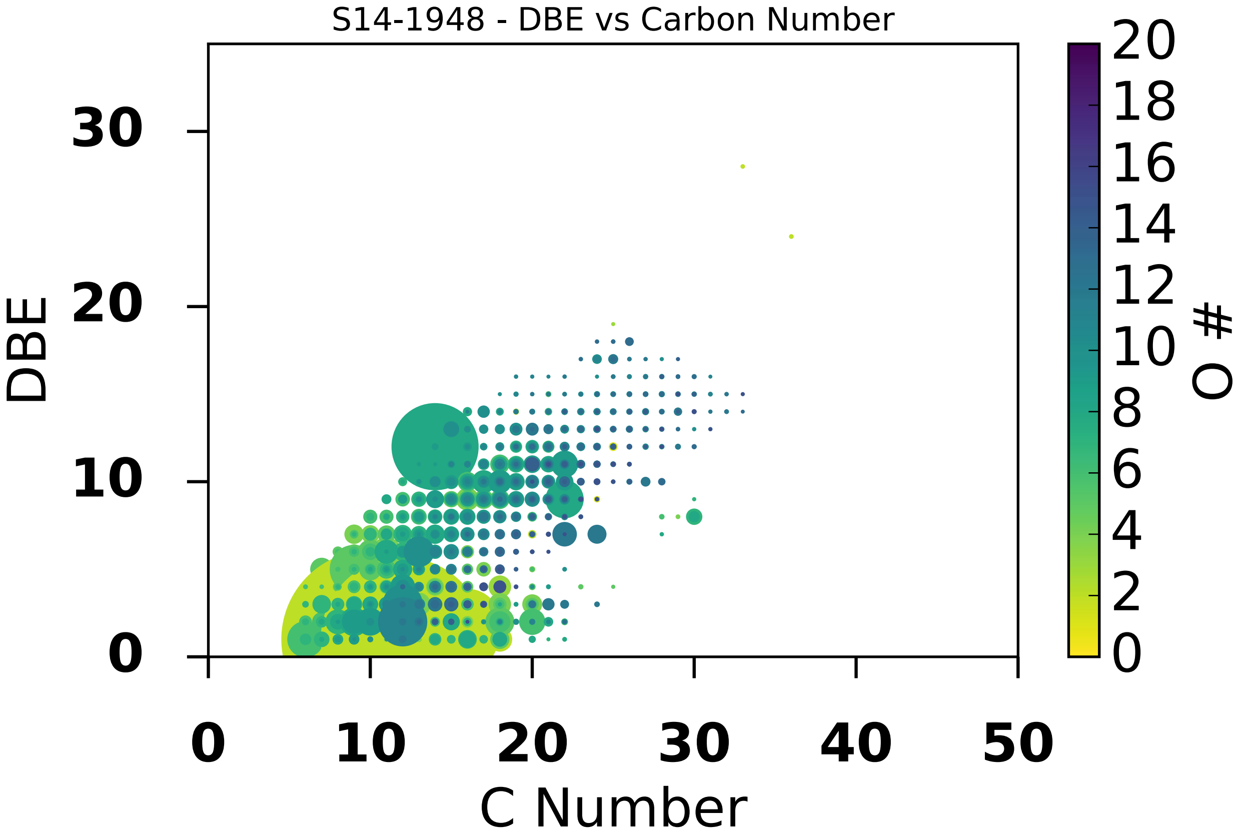

S14-1948 - DBE vs Carbon Number
DBE
C Number
O #
0
10
20
30
0
10
20
30
40
50
0
2
4
6
8
10
12
14
16
18
20

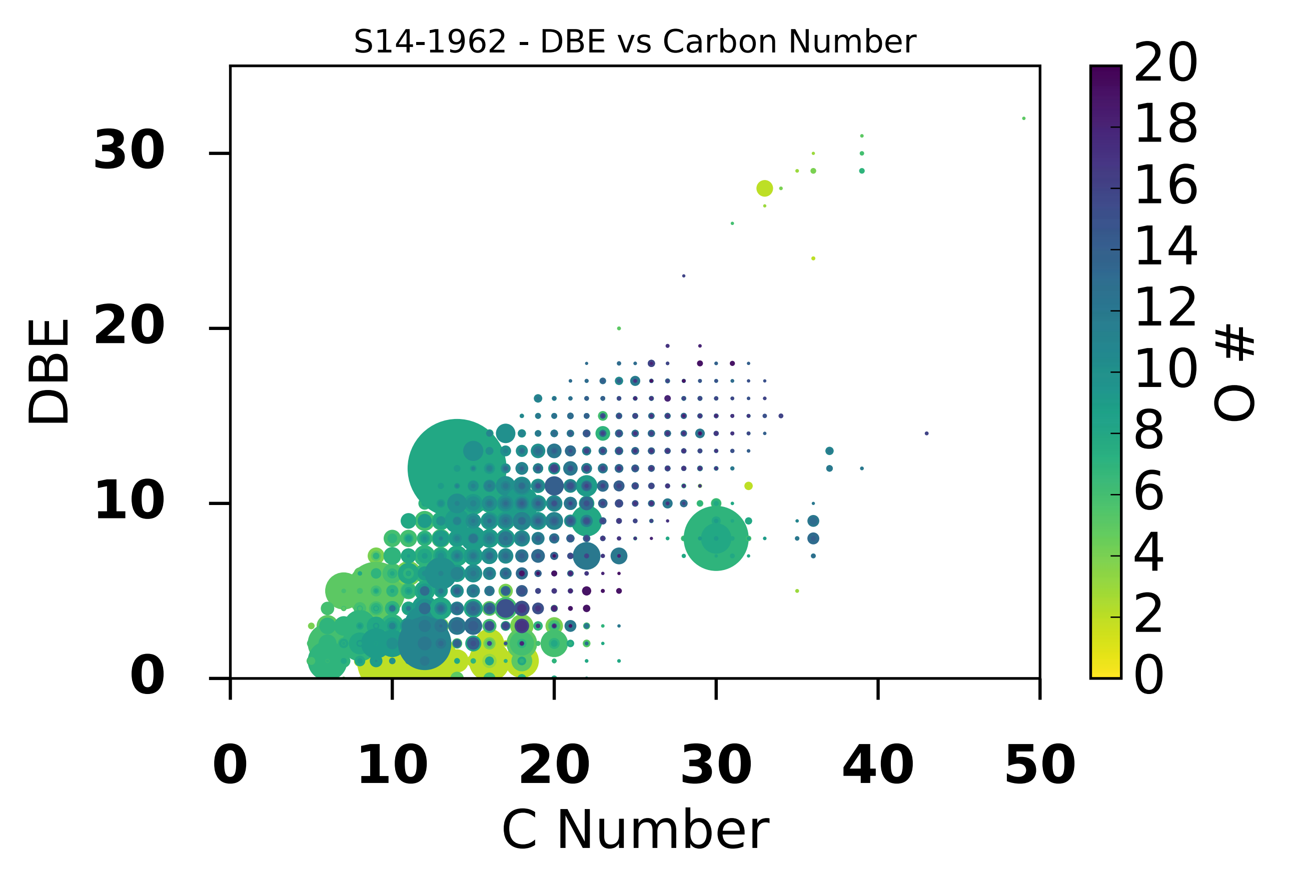
S14-1962 - DBE vs Carbon Number
DBE
0
10
20
30
C Number
0
10
20
30
40
50
O #
0
2
4
6
8
10
12
14
16
18
20

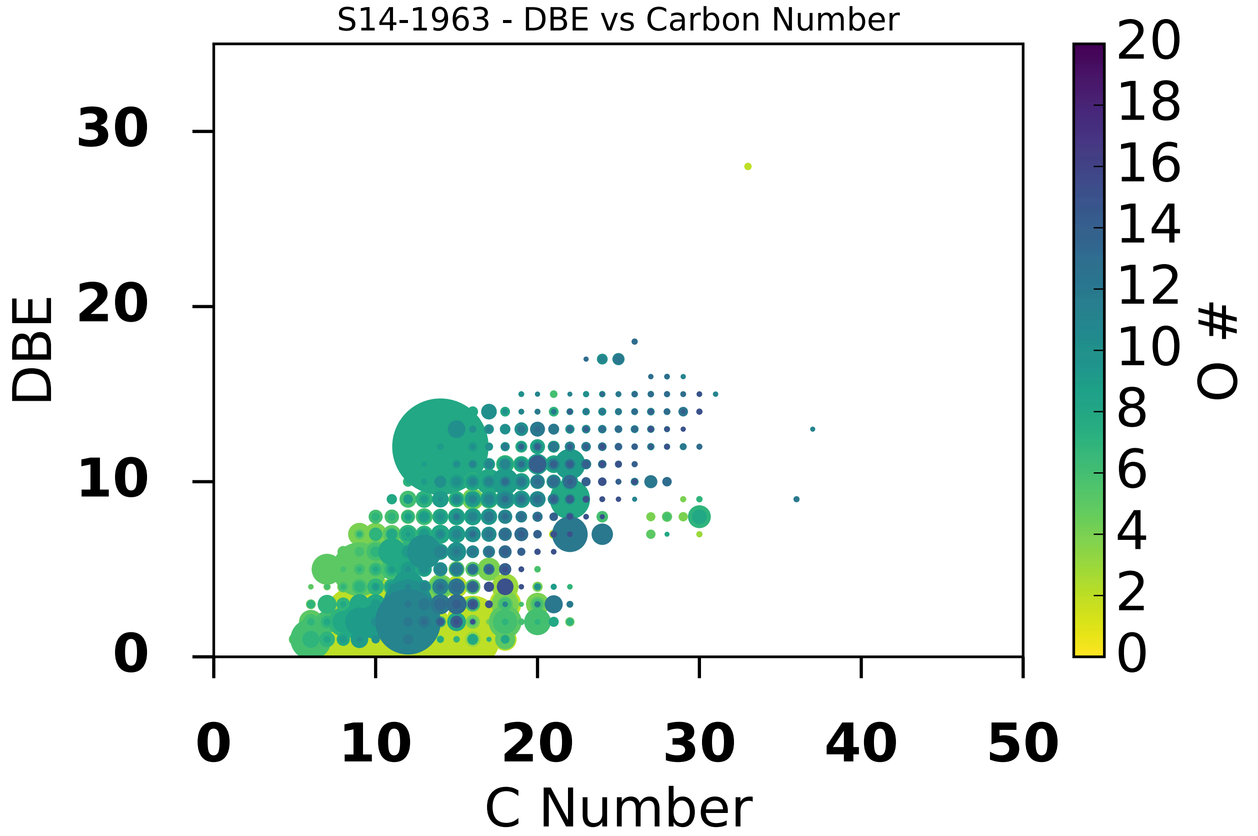
S14-1963 - DBE vs Carbon Number
DBE
30
20
10
0
0
10
20
30
40
50
C Number
O #
20
18
16
14
12
10
8
6
4
2
0

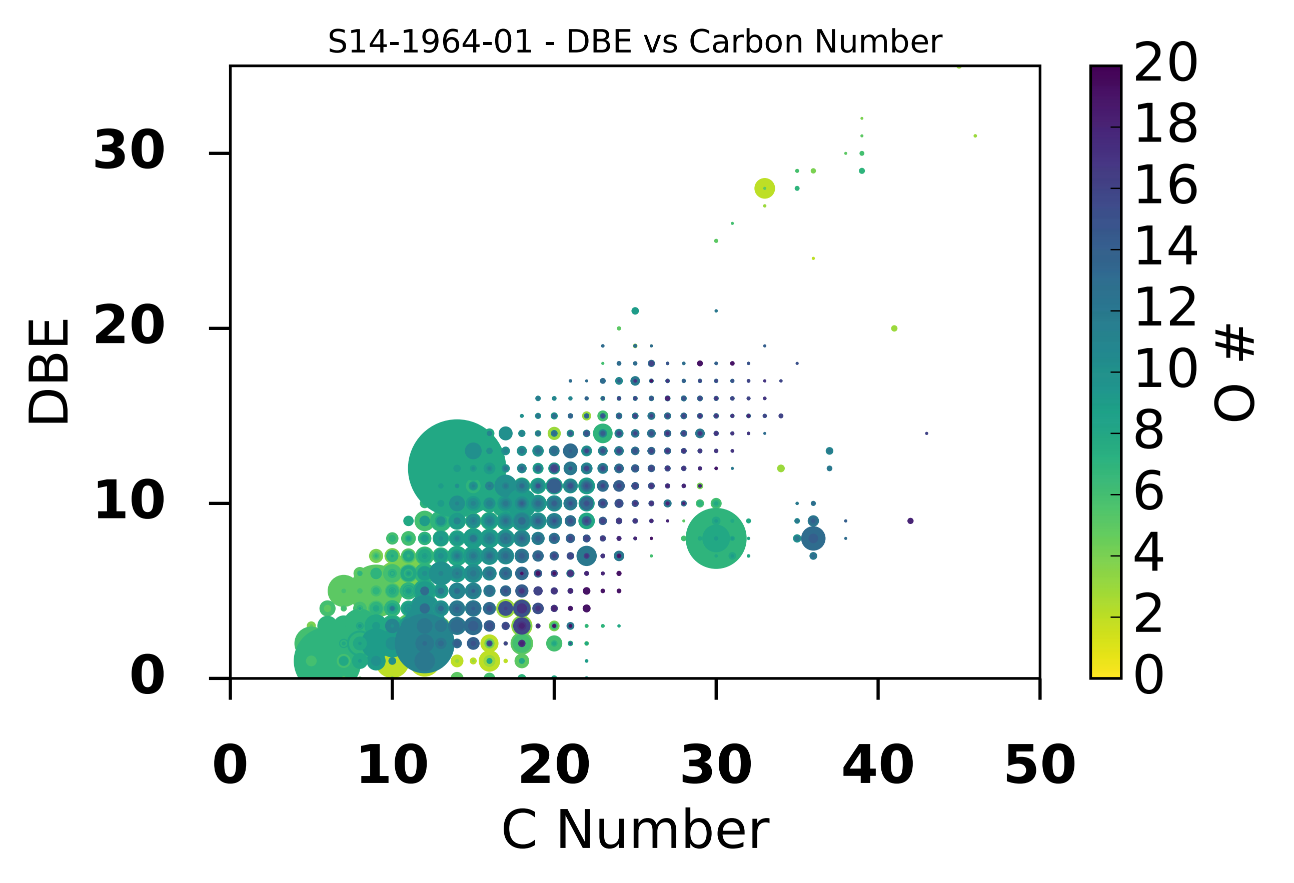
S14-1964-01 - DBE vs Carbon Number
DBE
0
10
20
30
C Number
0
10
20
30
40
50
O #
0
2
4
6
8
10
12
14
16
18
20

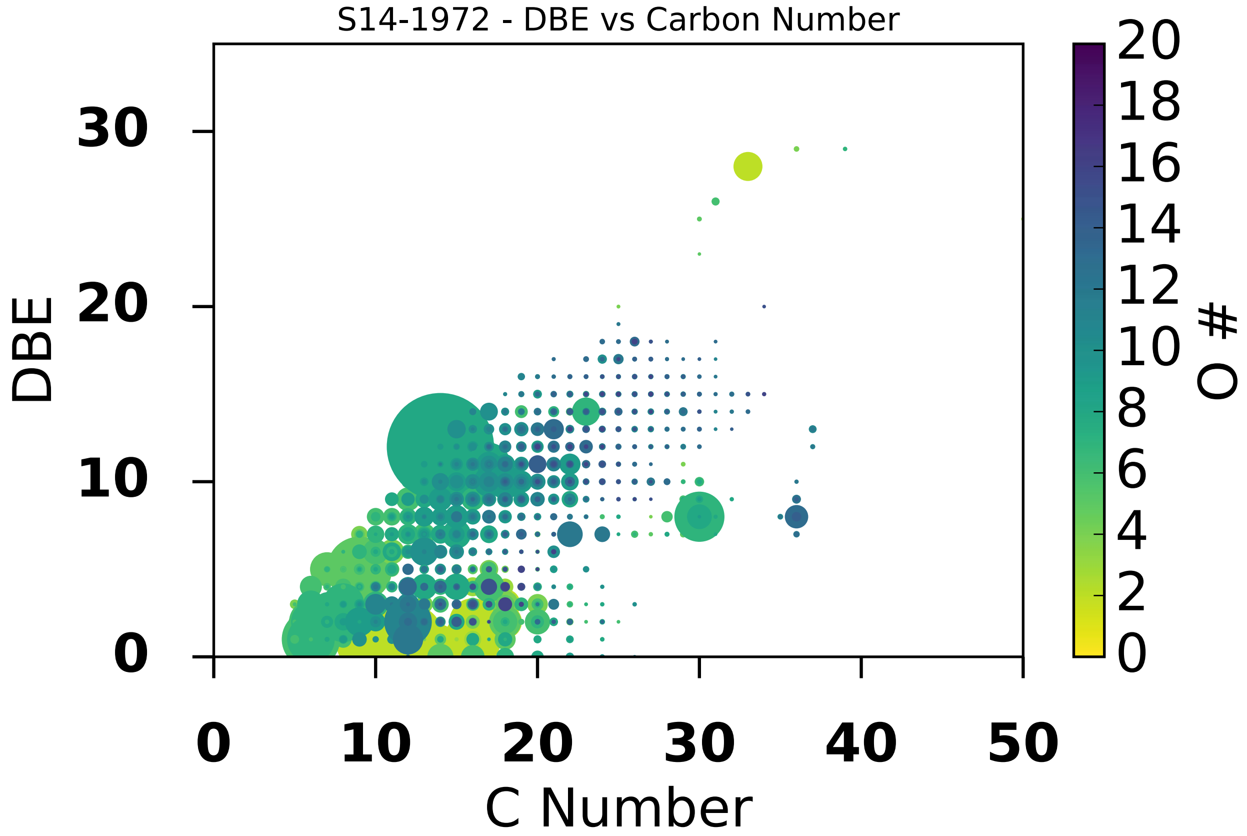
S14-1972 - DBE vs Carbon Number
DBE
0
10
20
30
C Number
0
10
20
30
40
50
O #
0
2
4
6
8
10
12
14
16
18
20

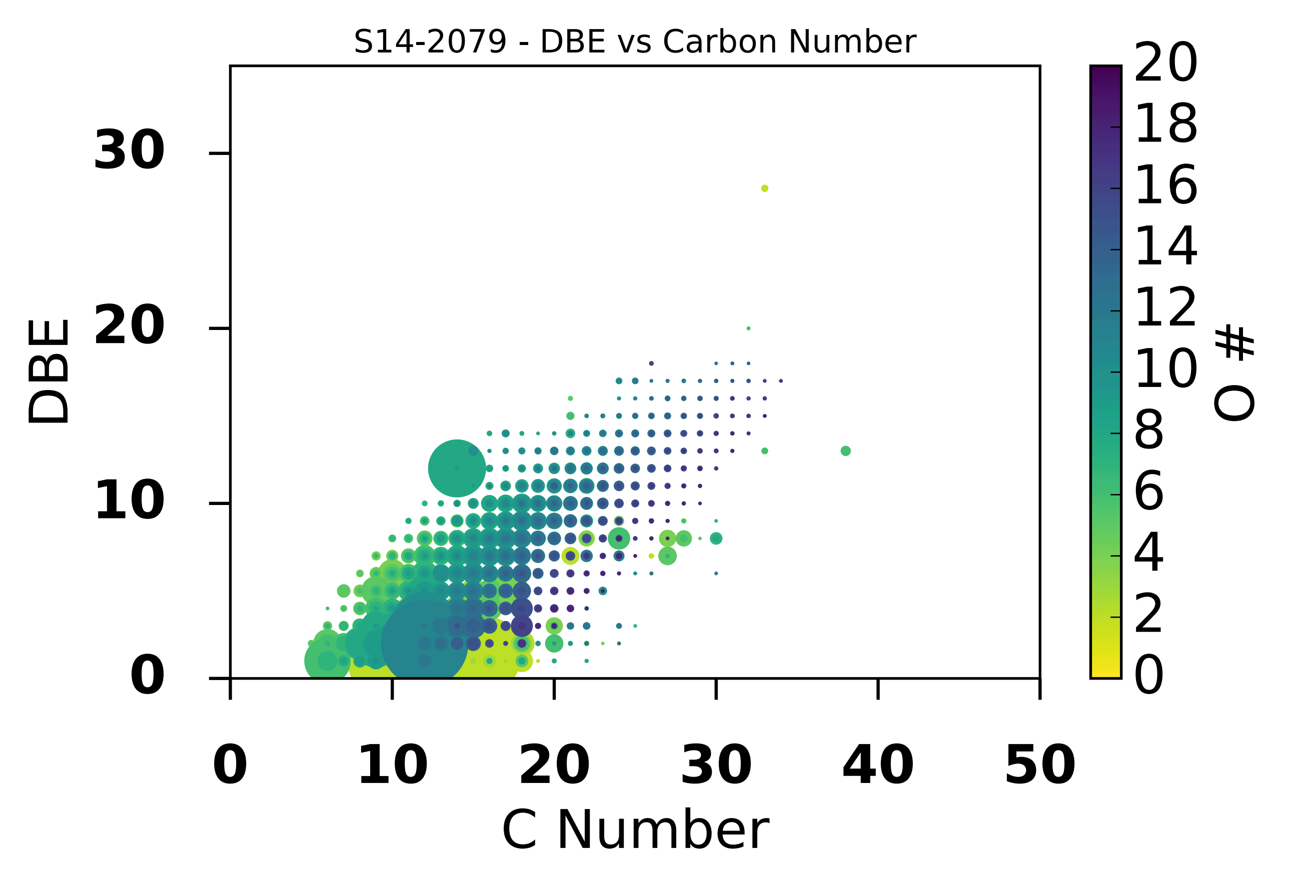

S14-2079 - DBE vs Carbon Number
DBE
0
10
20
30
C Number
0
10
20
30
40
50
O #
0
2
4
6
8
10
12
14
16
18
20

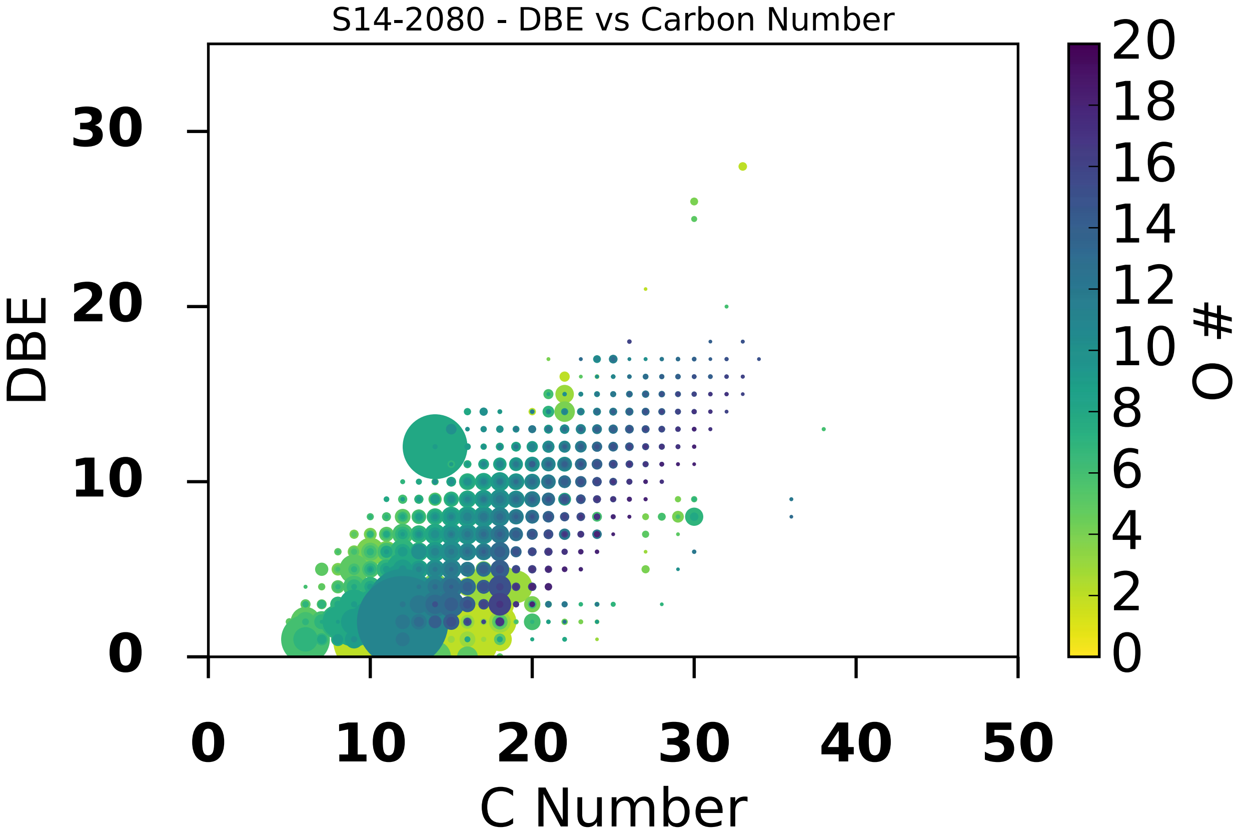

S14-2080 - DBE vs Carbon Number
DBE
30
20
10
0
0
10
20
30
40
50
C Number
O #
20
18
16
14
12
10
8
6
4
2
0

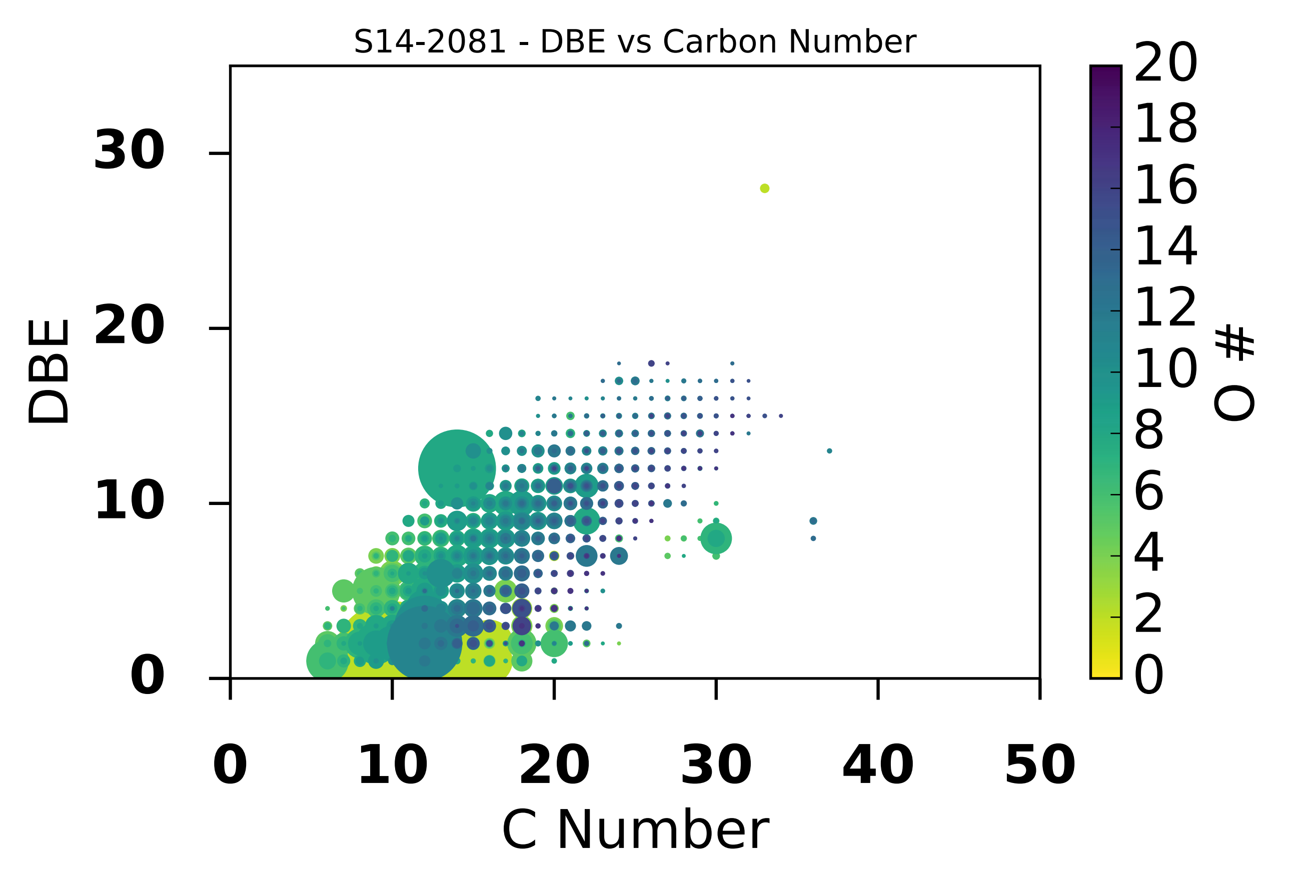

S14-2081 - DBE vs Carbon Number
DBE
C Number
O #
0
10
20
30
40
50
0
2
4
6
8
10
12
14
16
18
20

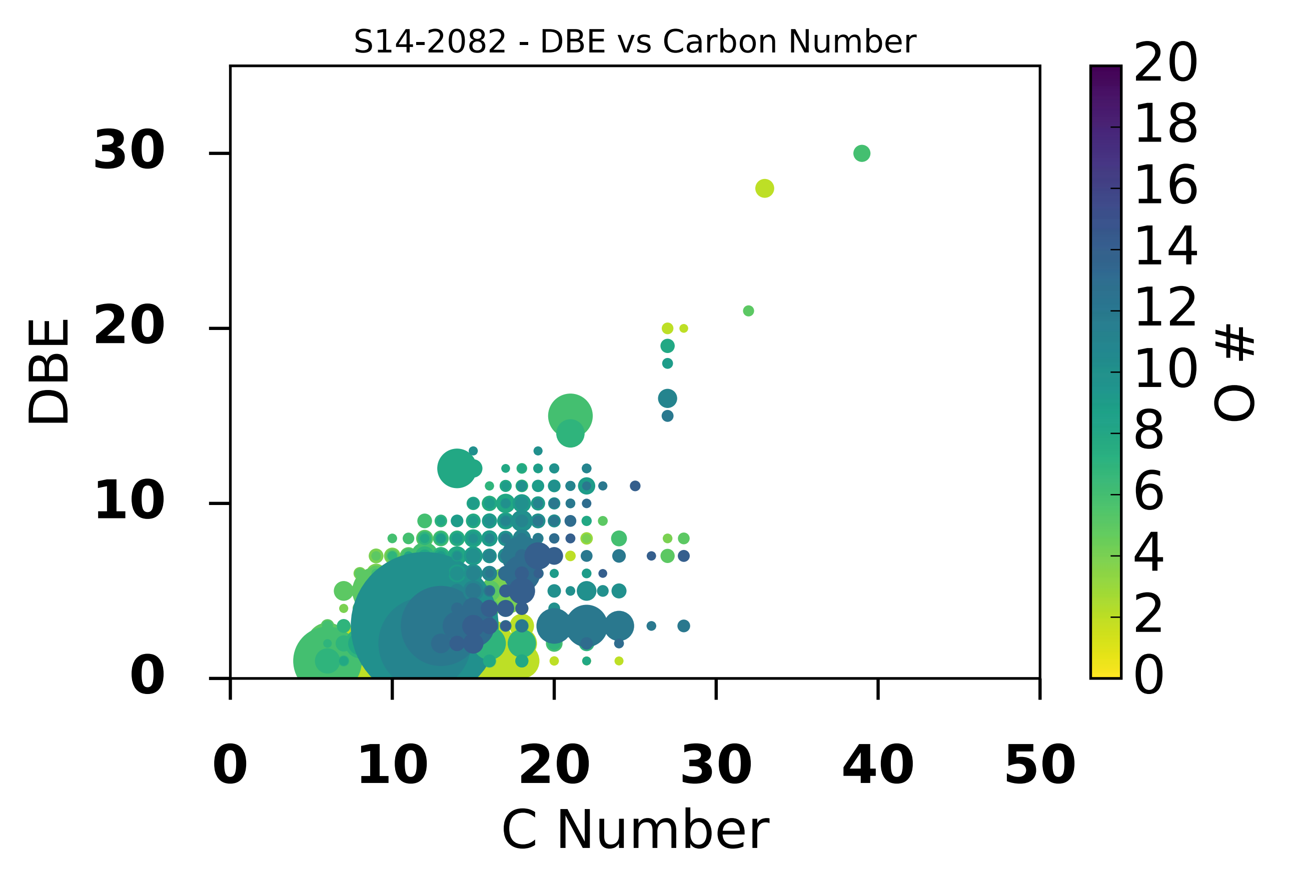
S14-2082 - DBE vs Carbon Number
DBE
C Number
O #
0
10
20
30
40
50
0
2
4
6
8
10
12
14
16
18
20

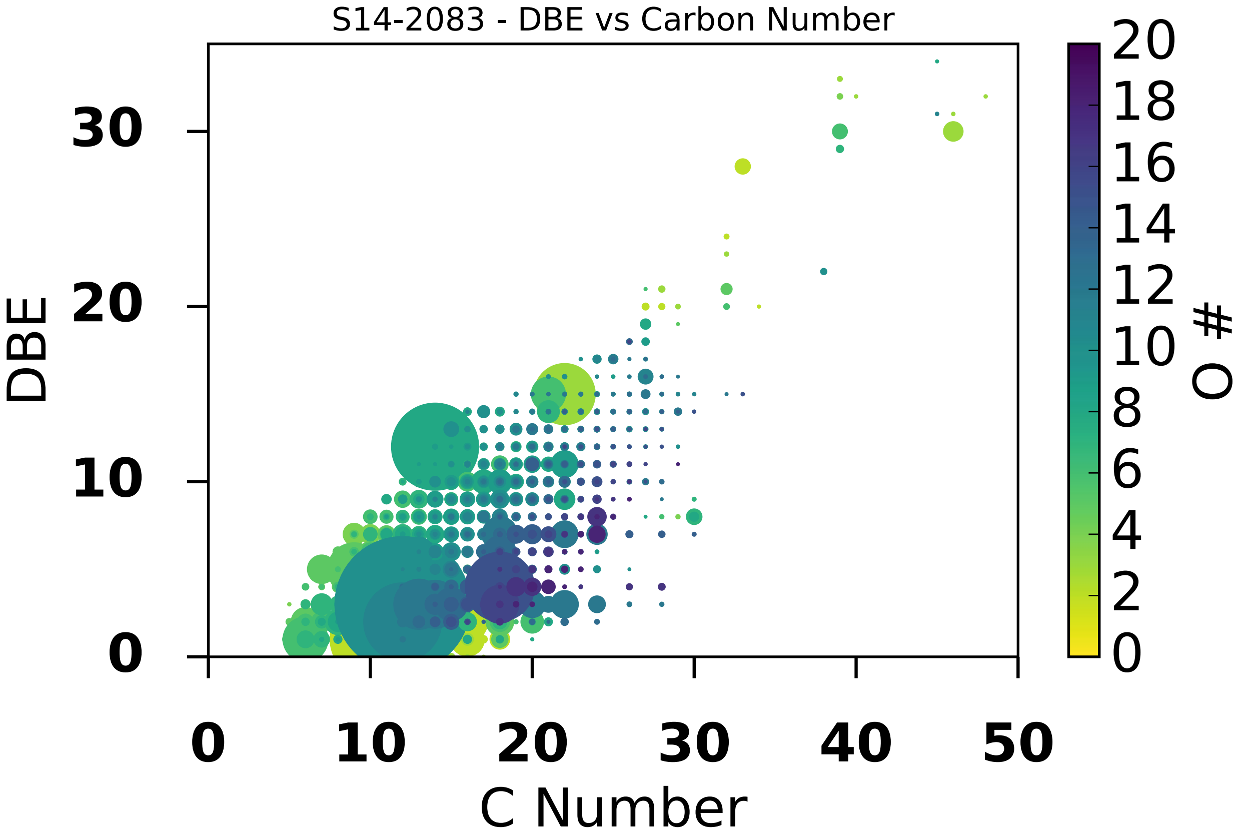
S14-2083 - DBE vs Carbon Number
DBE
0
10
20
30
C Number
0
10
20
30
40
50
O #
0
2
4
6
8
10
12
14
16
18
20

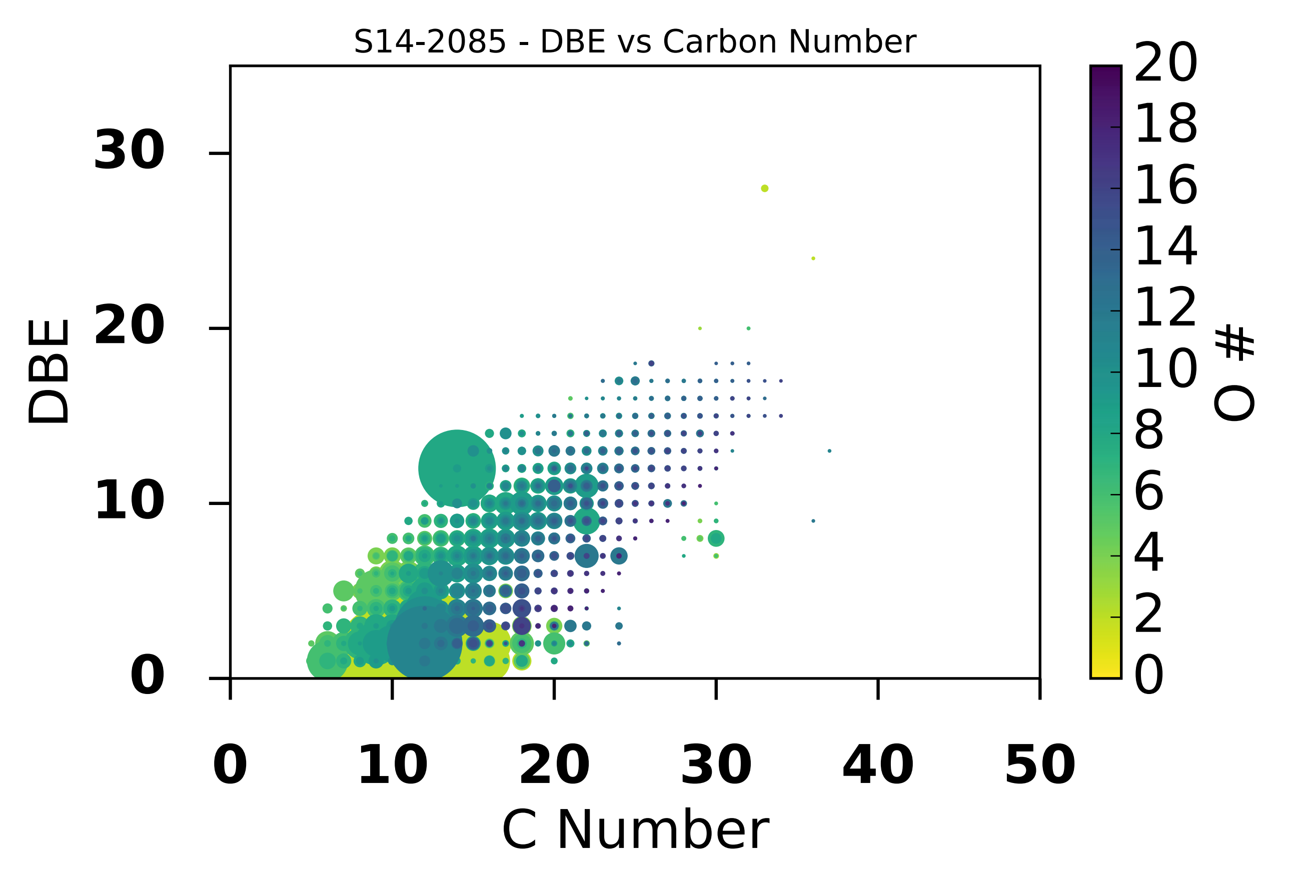

S14-2085 - DBE vs Carbon Number
DBE
30
20
10
0
0
10
20
30
40
50
C Number
O #
20
18
16
14
12
10
8
6
4
2
0

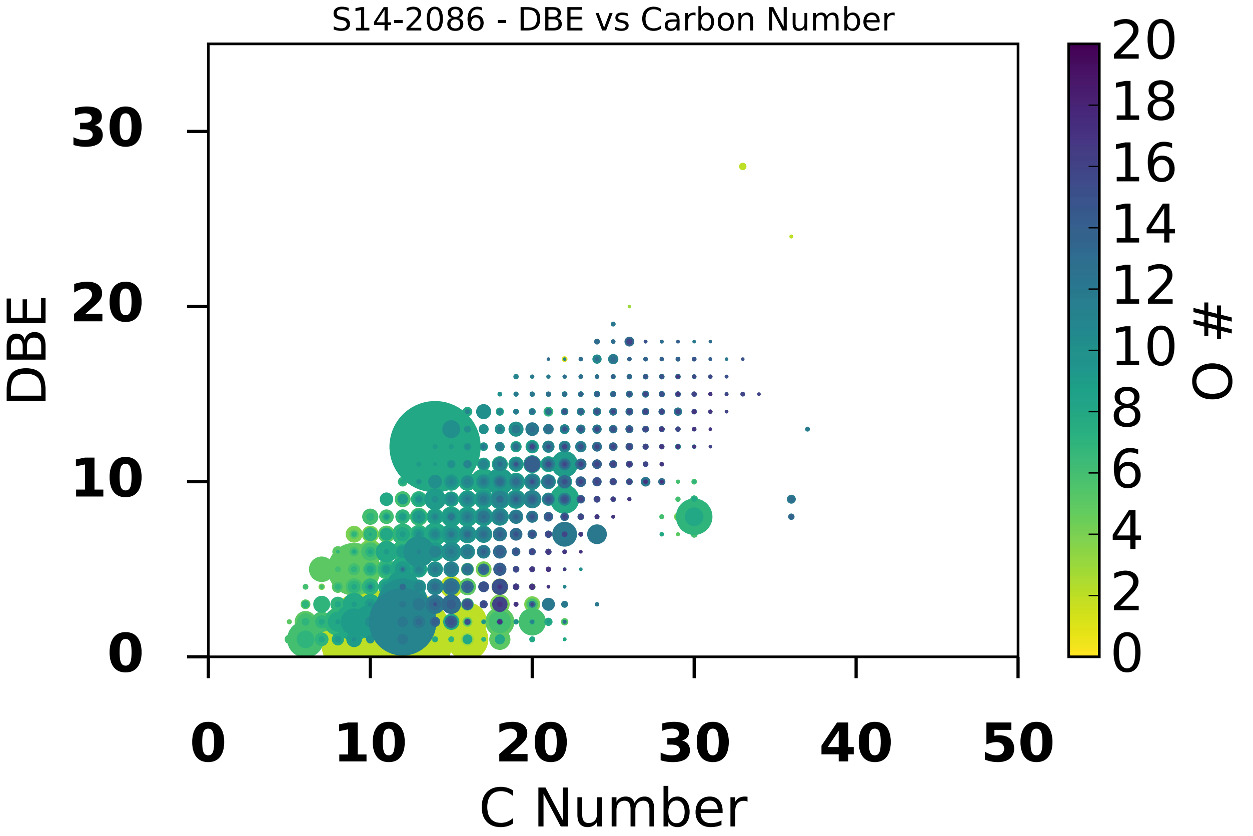
S14-2086 - DBE vs Carbon Number
DBE
0
10
20
30
0
10
20
30
40
50
C Number
O #
0
2
4
6
8
10
12
14
16
18
20

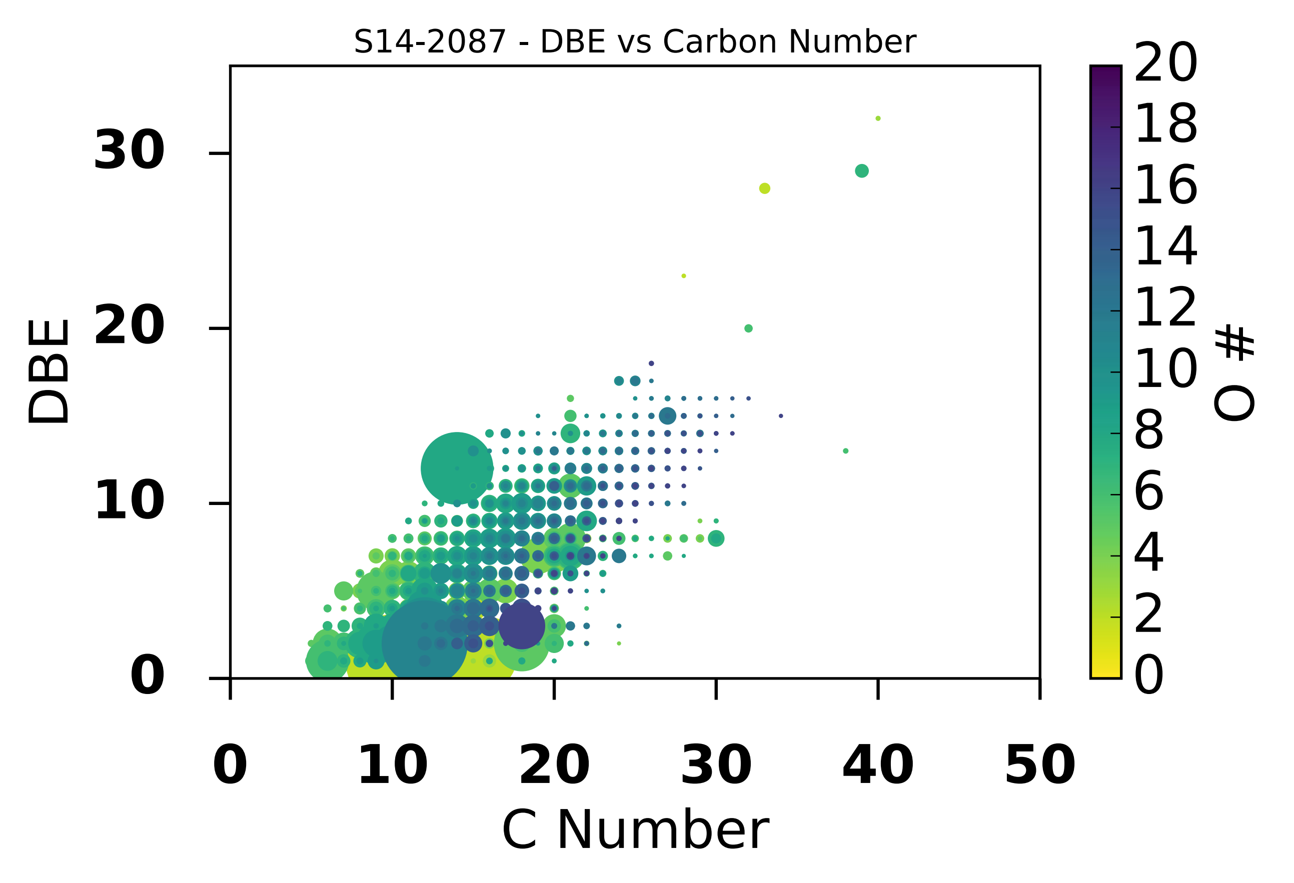
S14-2087 - DBE vs Carbon Number
DBE
30
20
10
0
0
10
20
30
40
50
C Number
O #
20
18
16
14
12
10
8
6
4
2
0

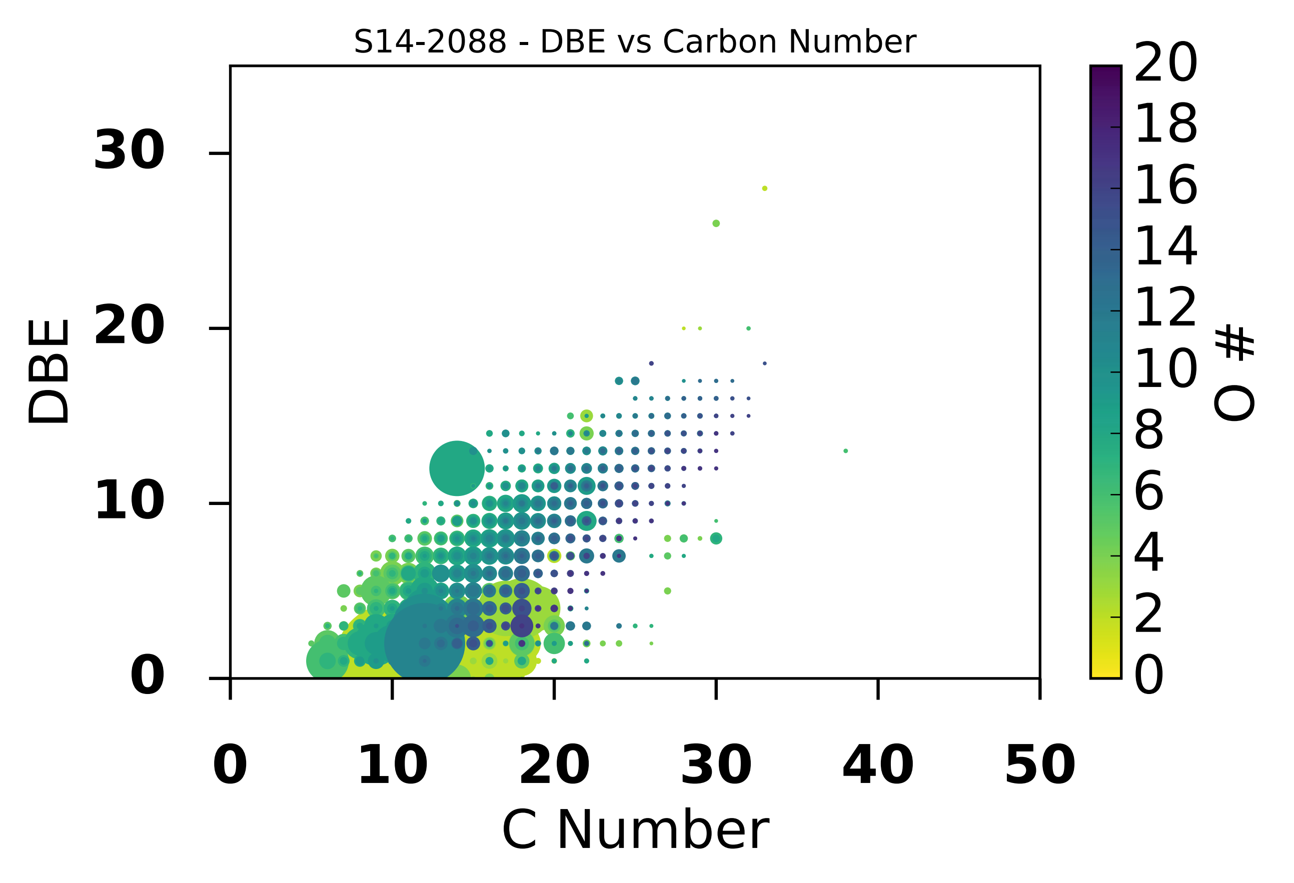
S14-2088 - DBE vs Carbon Number
DBE
30
20
10
0
0
10
20
30
40
50
C Number
20
18
16
14
12
10
8
6
4
2
0
O #

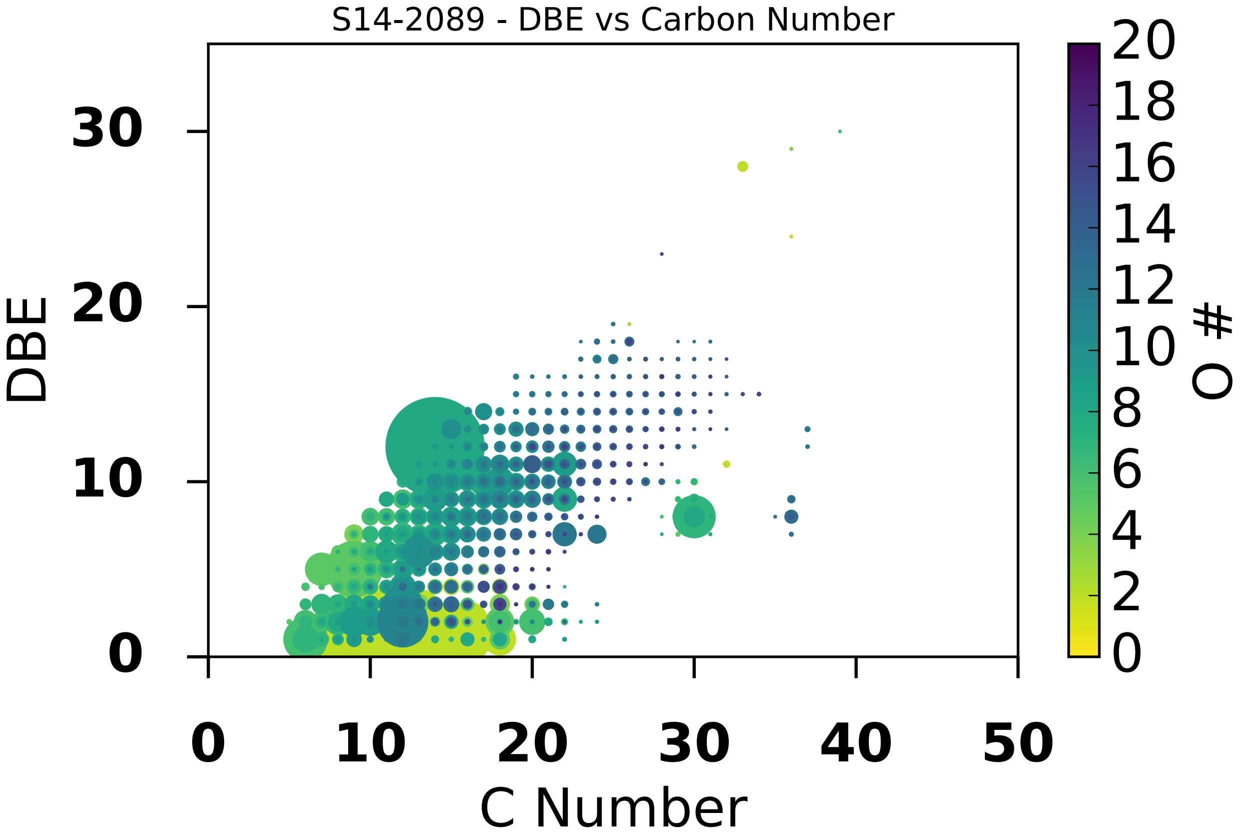
S14-2089 - DBE vs Carbon Number
DBE
30
20
10
0
0
10
20
30
40
50
C Number
O #
20
18
16
14
12
10
8
6
4
2
0

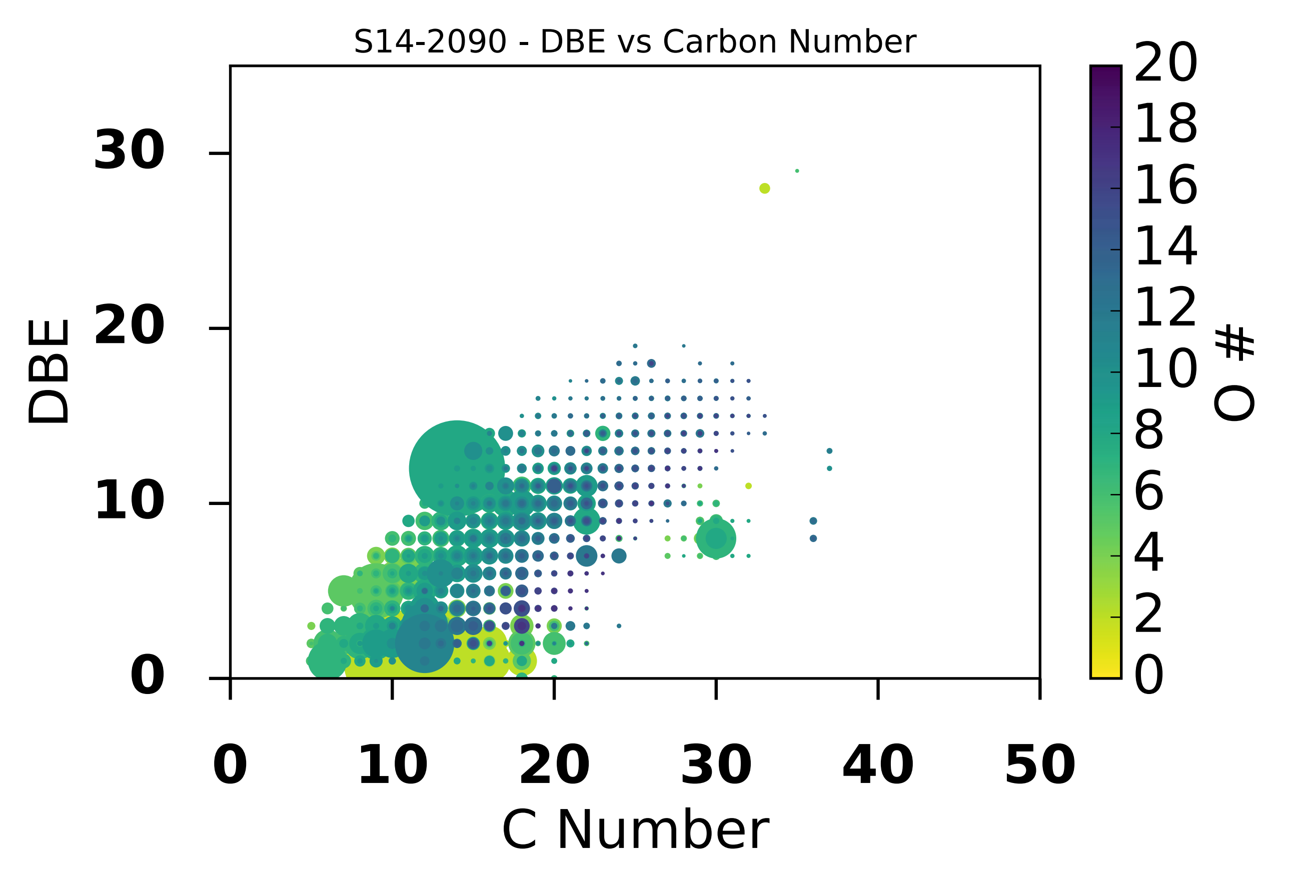
S14-2090 - DBE vs Carbon Number
DBE
C Number
O #
0
10
20
30
0
10
20
30
40
50
0
2
4
6
8
10
12
14
16
18
20

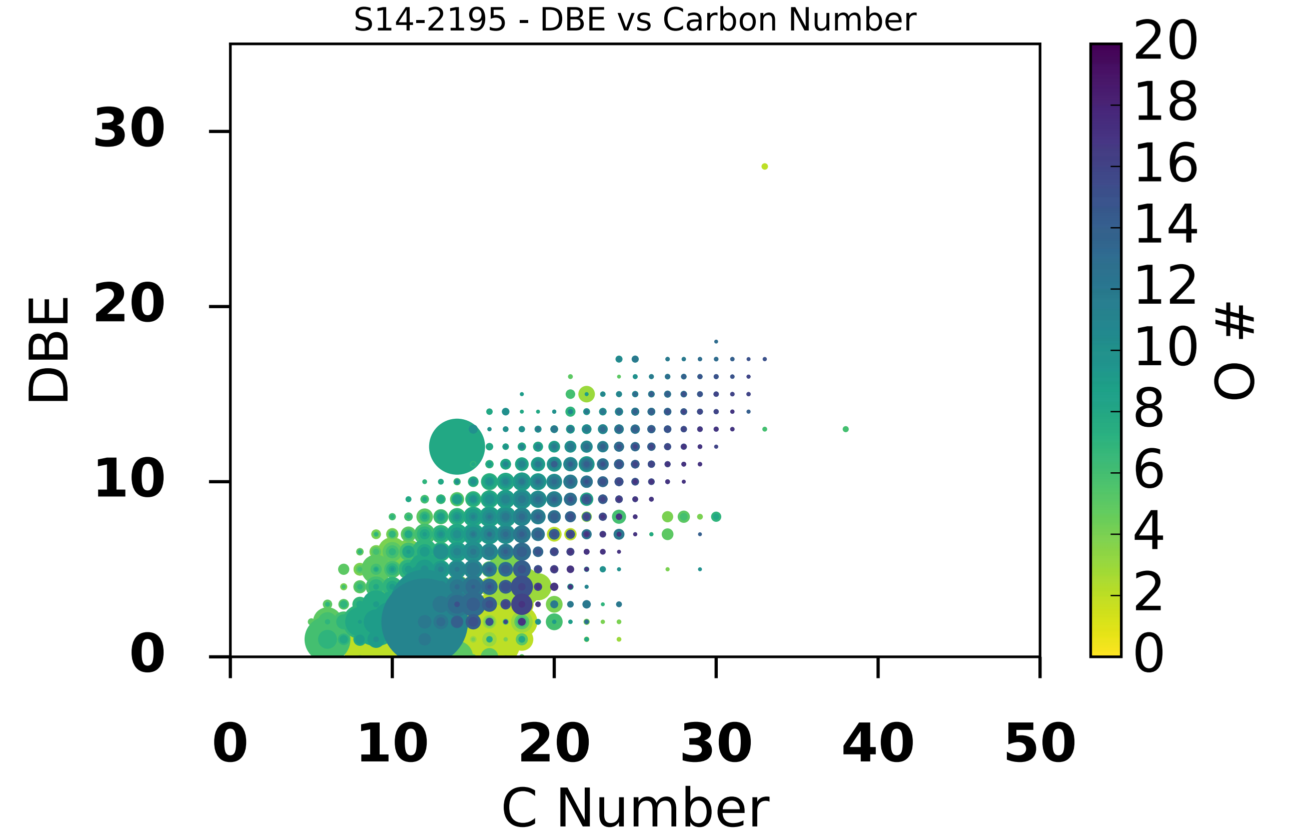
S14-2195 - DBE vs Carbon Number
DBE
30
20
10
0
0
10
20
30
40
50
C Number
20
18
16
14
12
10
8
6
4
2
0
O #

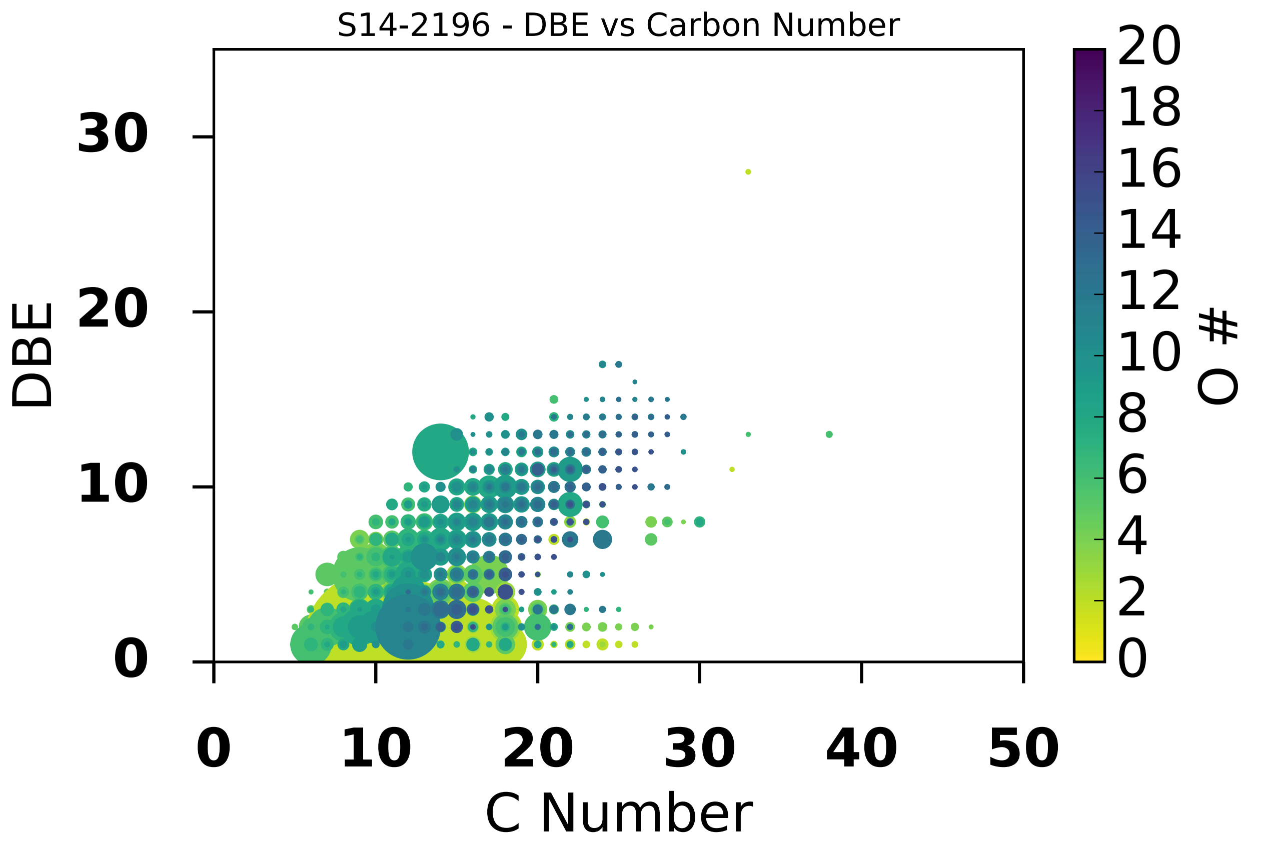
S14-2196 - DBE vs Carbon Number
DBE
0
10
20
30
C Number
0
10
20
30
40
50
O #
0
2
4
6
8
10
12
14
16
18
20

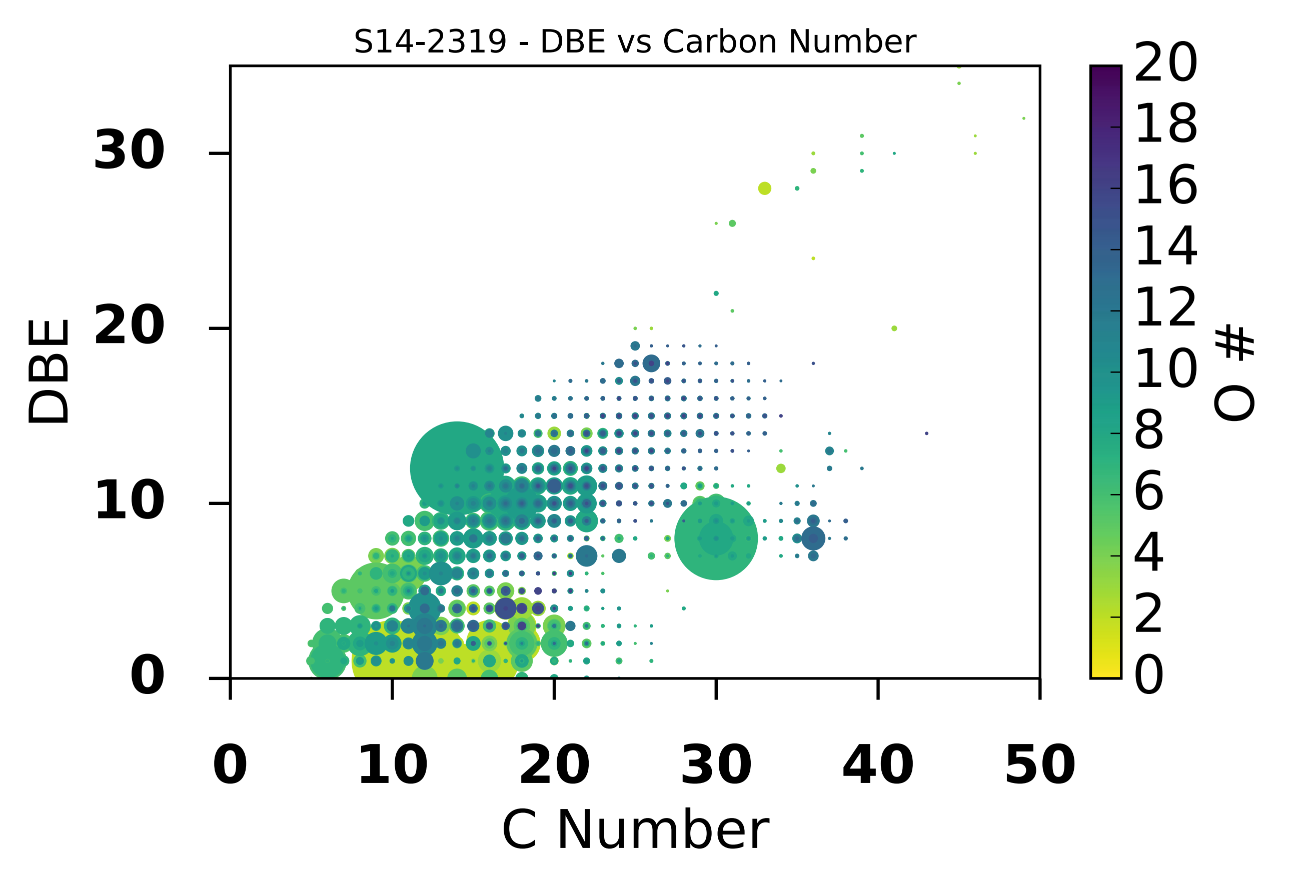
S14-2319 - DBE vs Carbon Number
DBE
30
20
10
0
0
10
20
30
40
50
C Number
O #
20
18
16
14
12
10
8
6
4
2
0

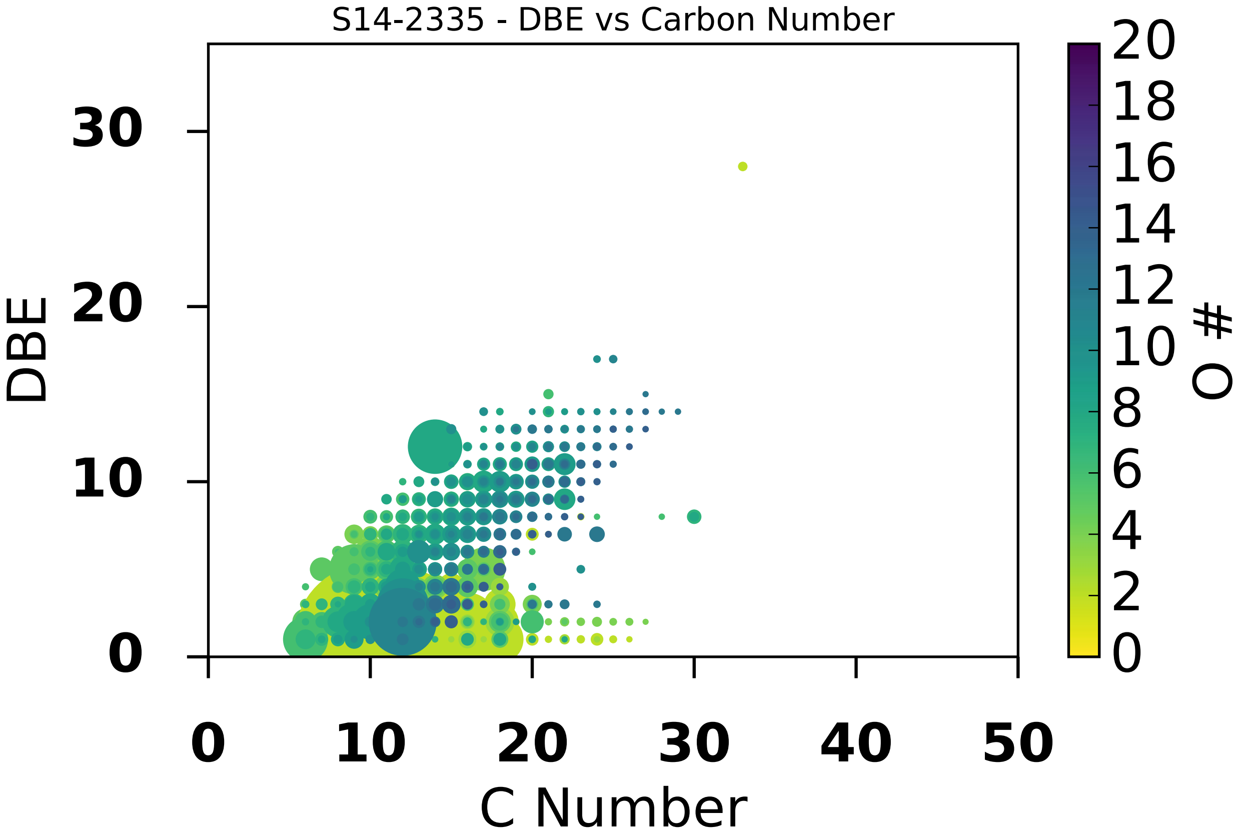
S14-2335 - DBE vs Carbon Number
DBE
30
20
10
0
0
10
20
30
40
50
C Number
O #
20
18
16
14
12
10
8
6
4
2
0

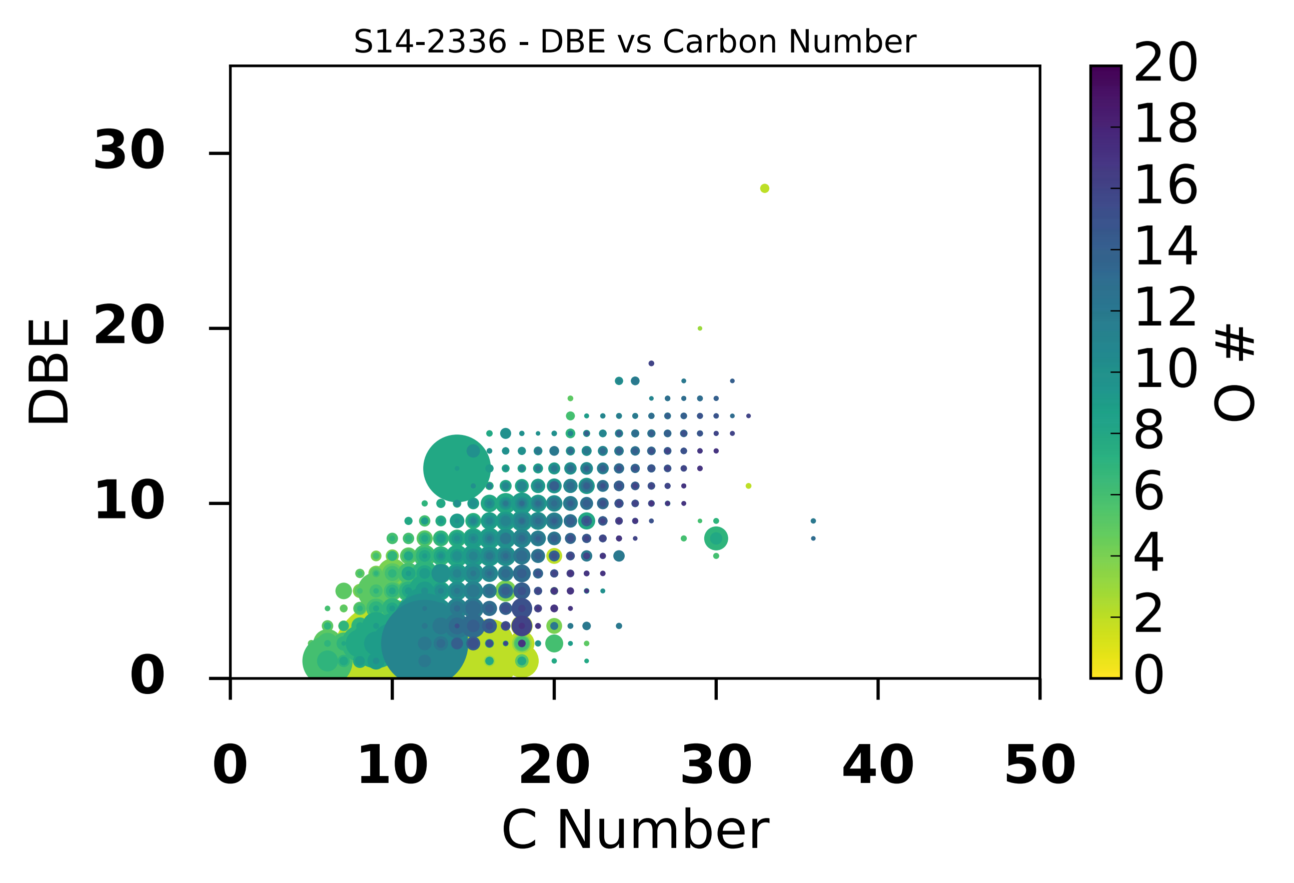
S14-2336 - DBE vs Carbon Number
DBE
0
10
20
30
C Number
0
10
20
30
40
50
O #
0
2
4
6
8
10
12
14
16
18
20

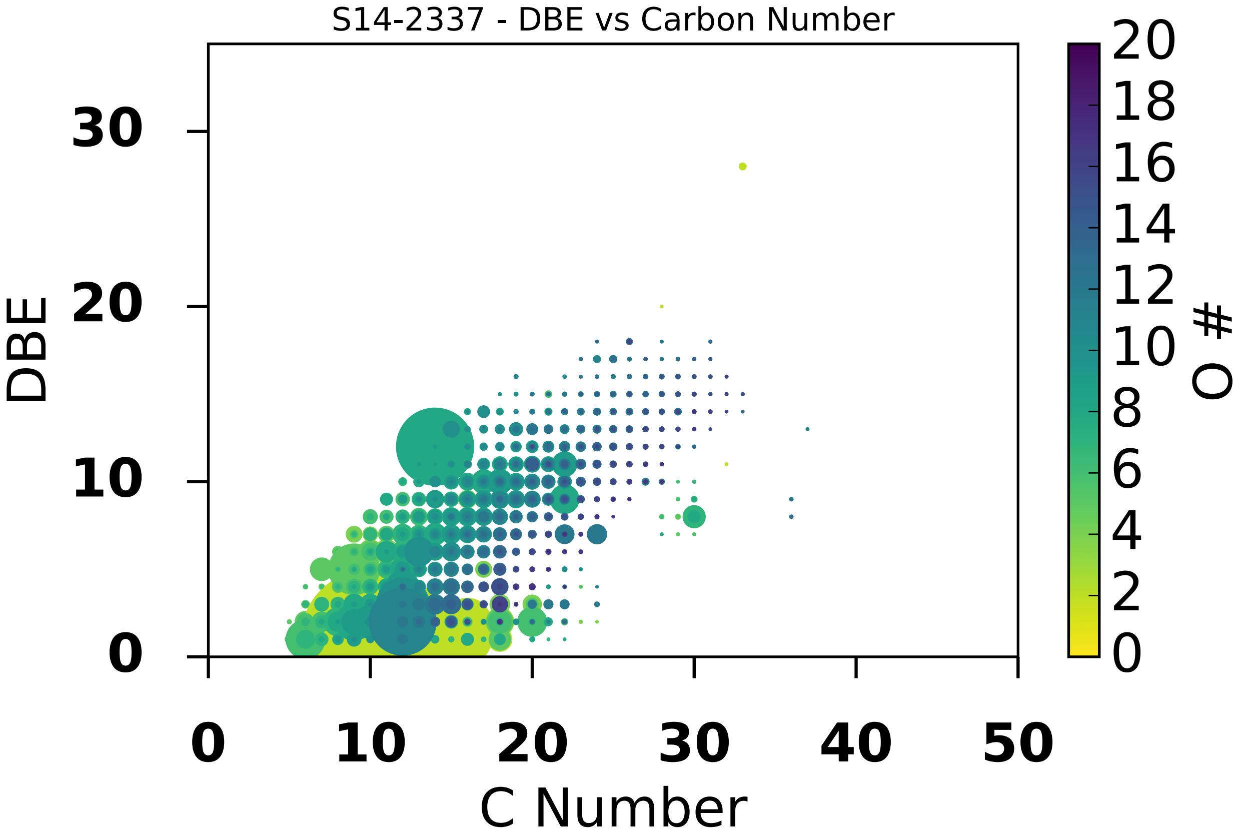

S14-2337 - DBE vs Carbon Number
DBE
0
10
20
30
C Number
0
10
20
30
40
50
O #
0
2
4
6
8
10
12
14
16
18
20

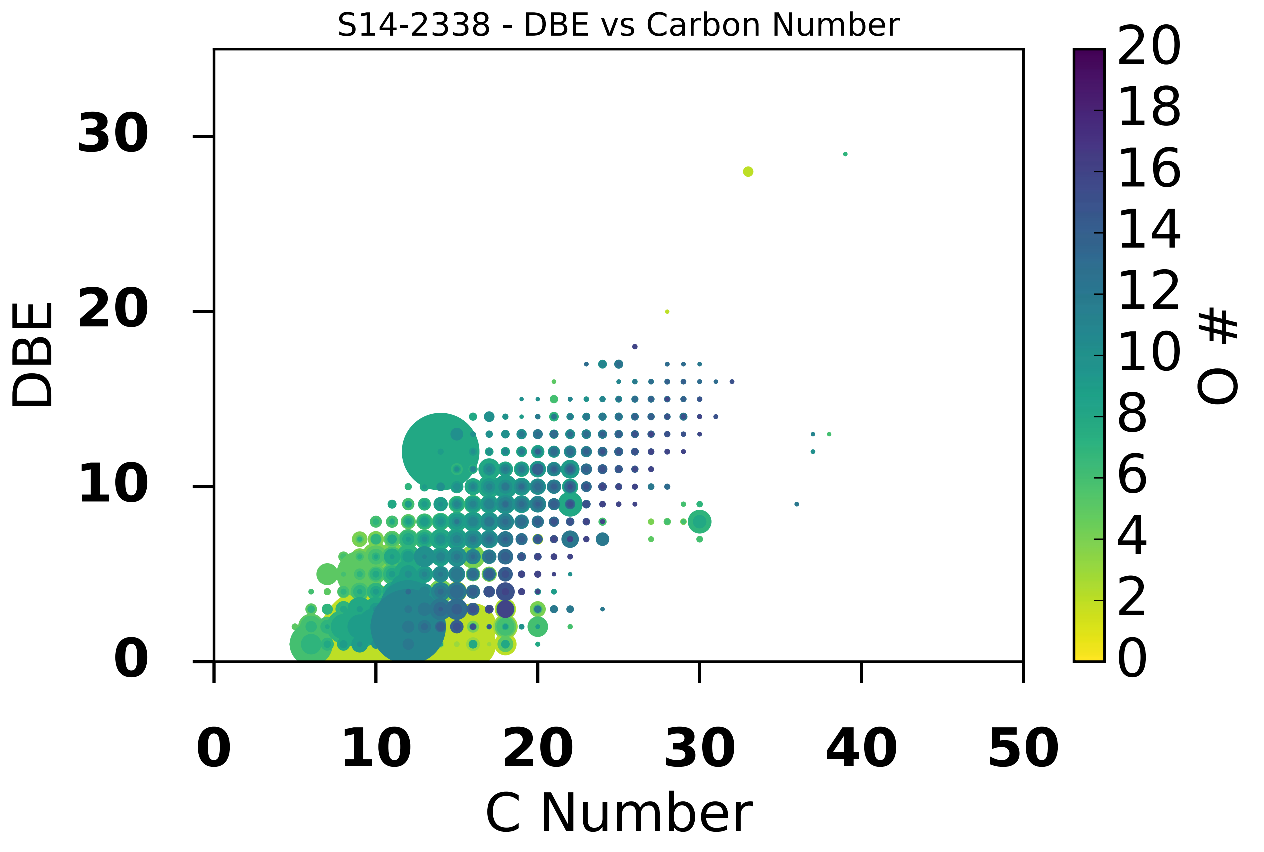

S14-2338 - DBE vs Carbon Number
DBE
0
10
20
30
C Number
0
10
20
30
40
50
O #
0
2
4
6
8
10
12
14
16
18
20

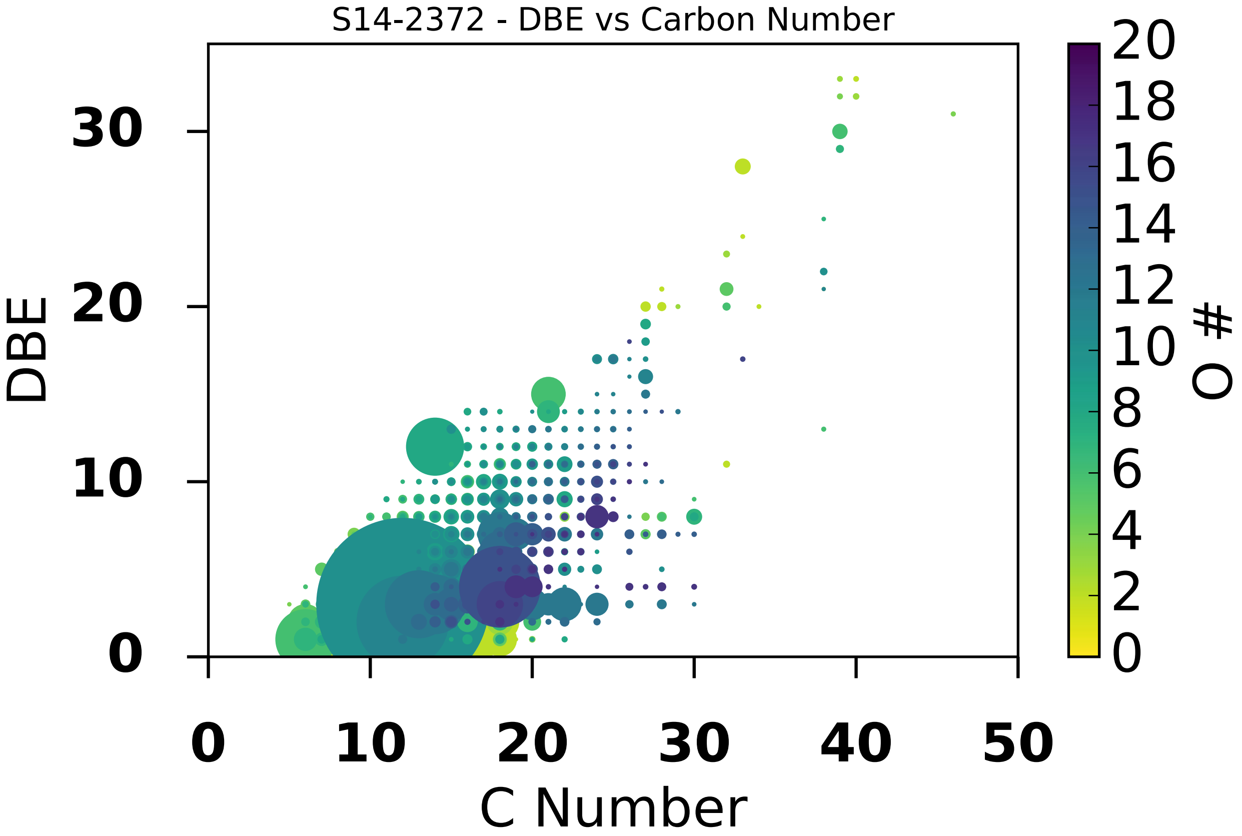

S14-2372 - DBE vs Carbon Number
DBE
C Number
O #
0
10
20
30
0
10
20
30
40
50
0
2
4
6
8
10
12
14
16
18
20

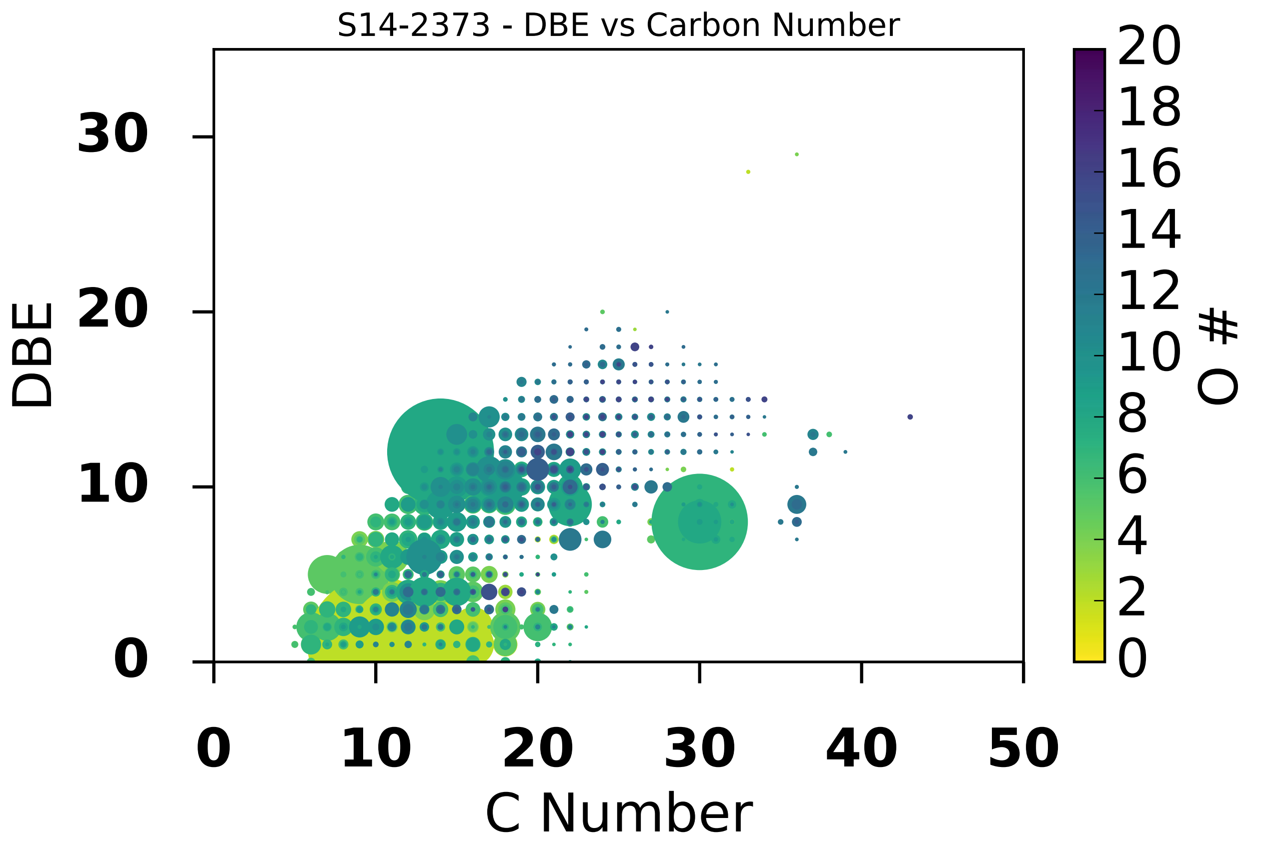

S14-2373 - DBE vs Carbon Number
DBE
0
10
20
30
C Number
0
10
20
30
40
50
O #
0
2
4
6
8
10
12
14
16
18
20

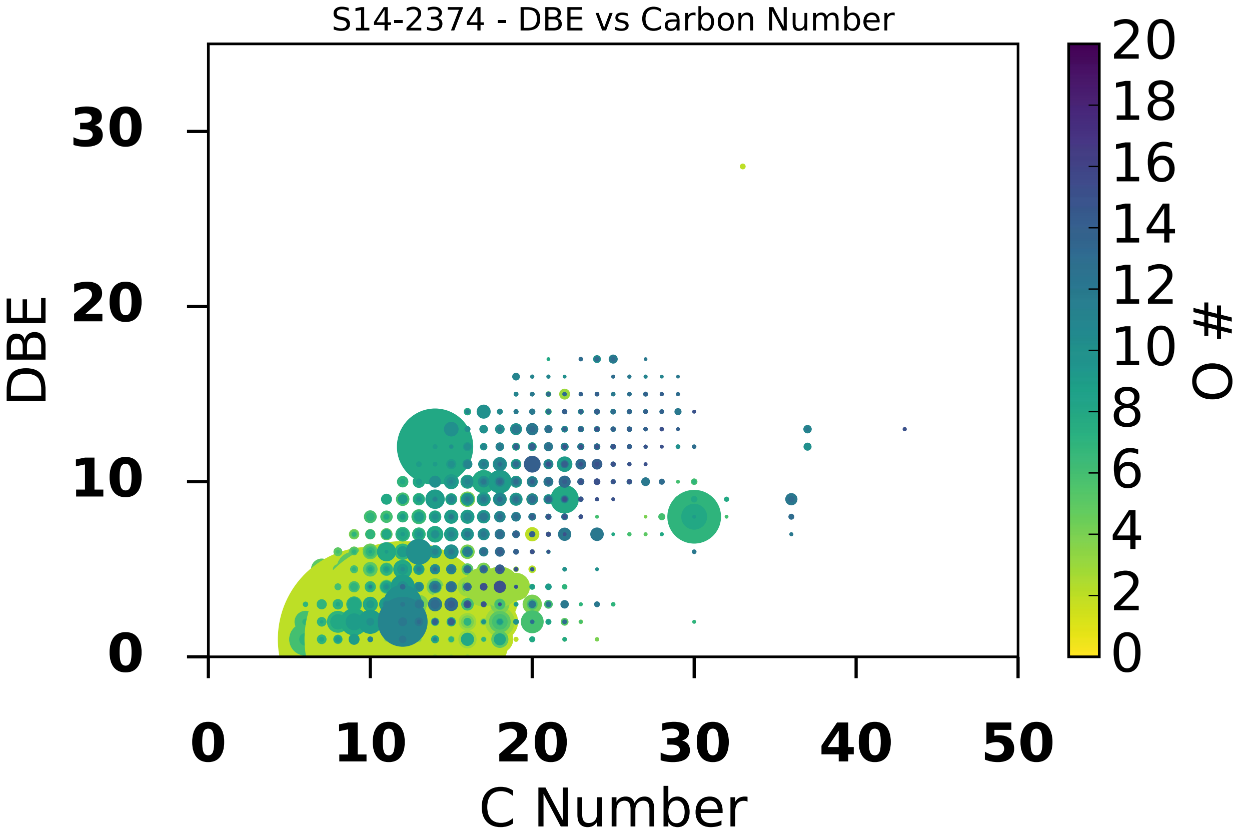
S14-2374 - DBE vs Carbon Number
DBE
C Number
O #
0
10
20
30
40
50
0
2
4
6
8
10
12
14
16
18
20

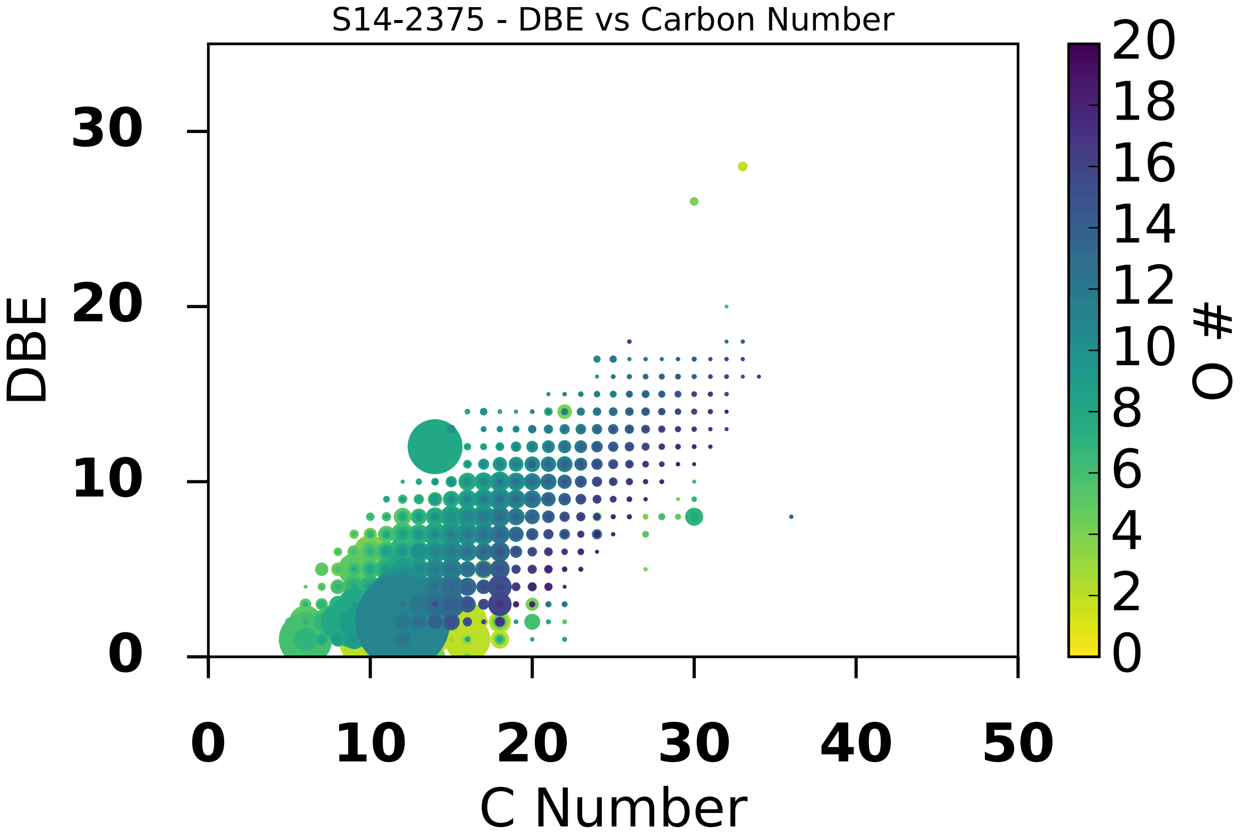
S14-2375 - DBE vs Carbon Number
DBE
30
20
10
0
0
10
20
30
40
50
C Number
O #
20
18
16
14
12
10
8
6
4
2
0

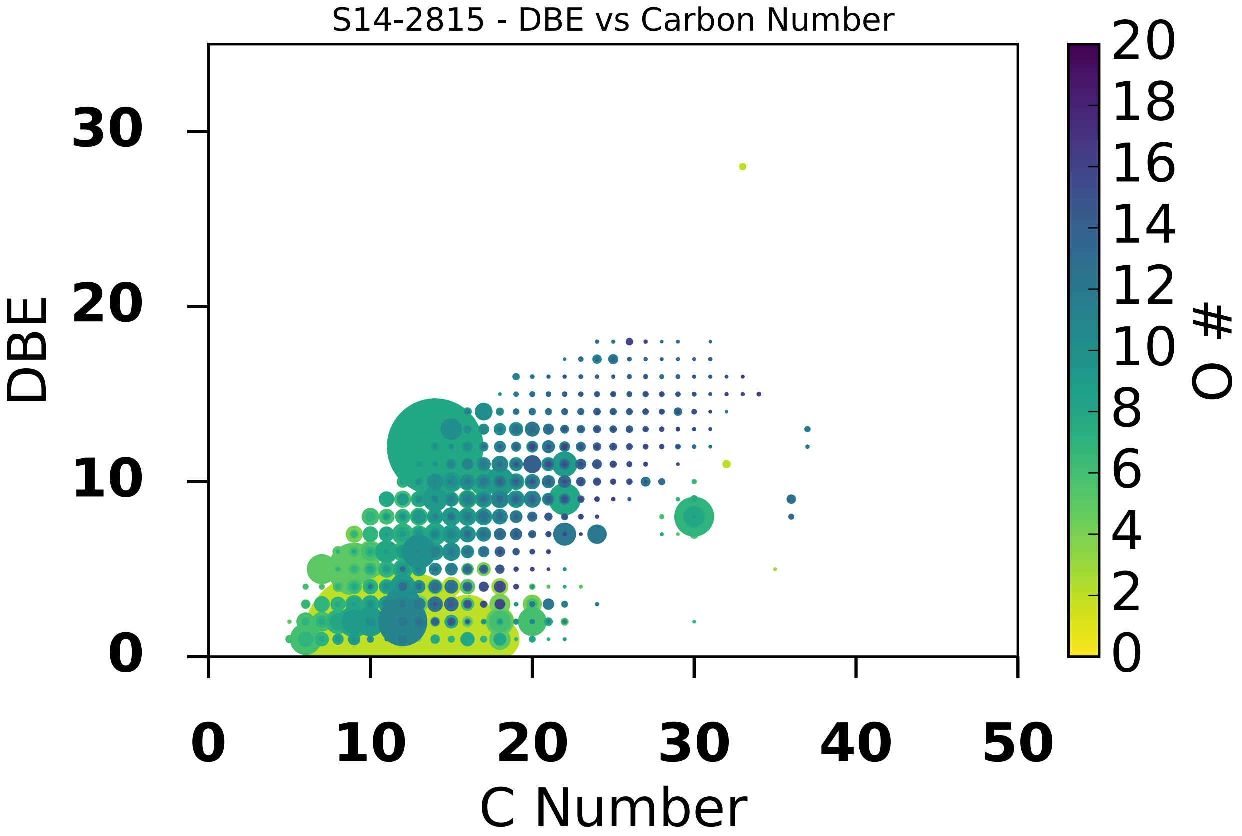
S14-2815 - DBE vs Carbon Number
DBE
30
20
10
0
0
10
20
30
40
50
C Number
20
18
16
14
12
10
8
6
4
2
0
O #

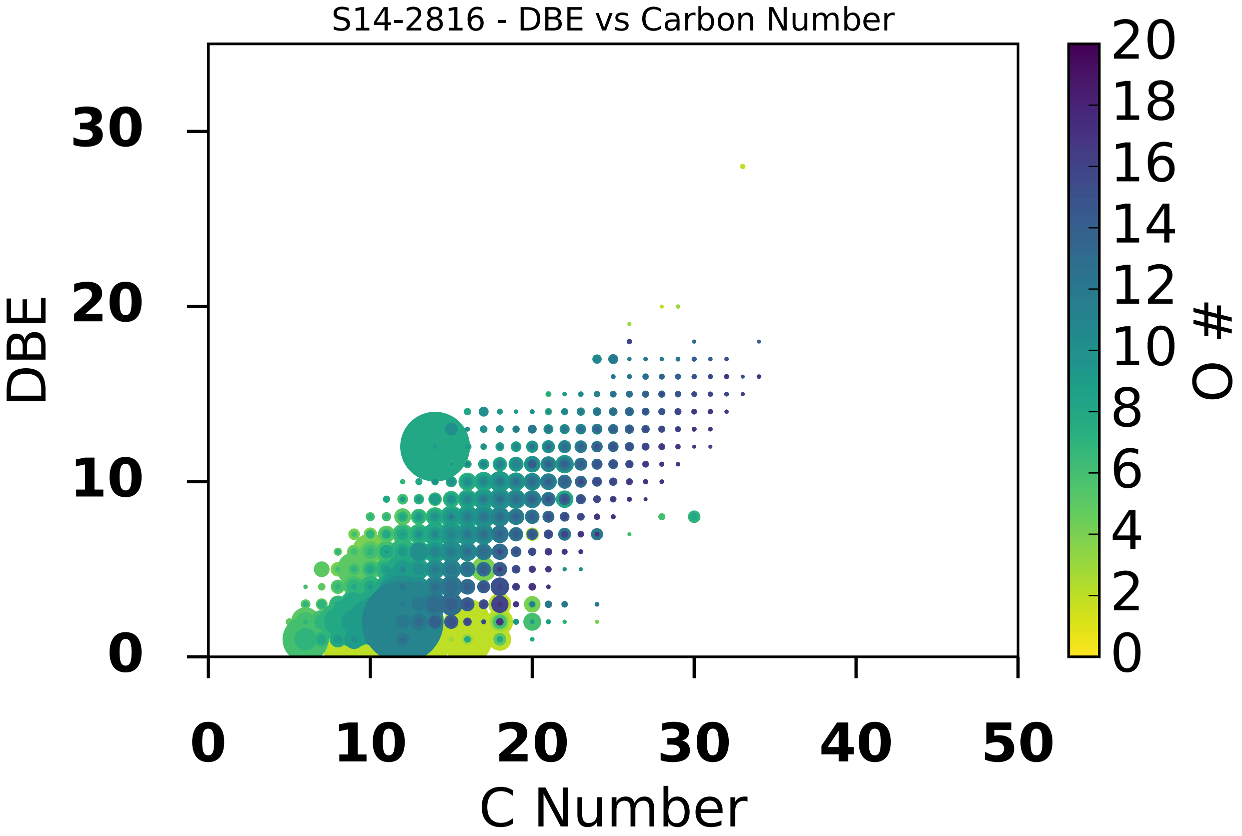
S14-2816 - DBE vs Carbon Number
DBE
30
20
10
0
0
10
20
30
40
50
C Number
20
18
16
14
12
10
8
6
4
2
0
O #

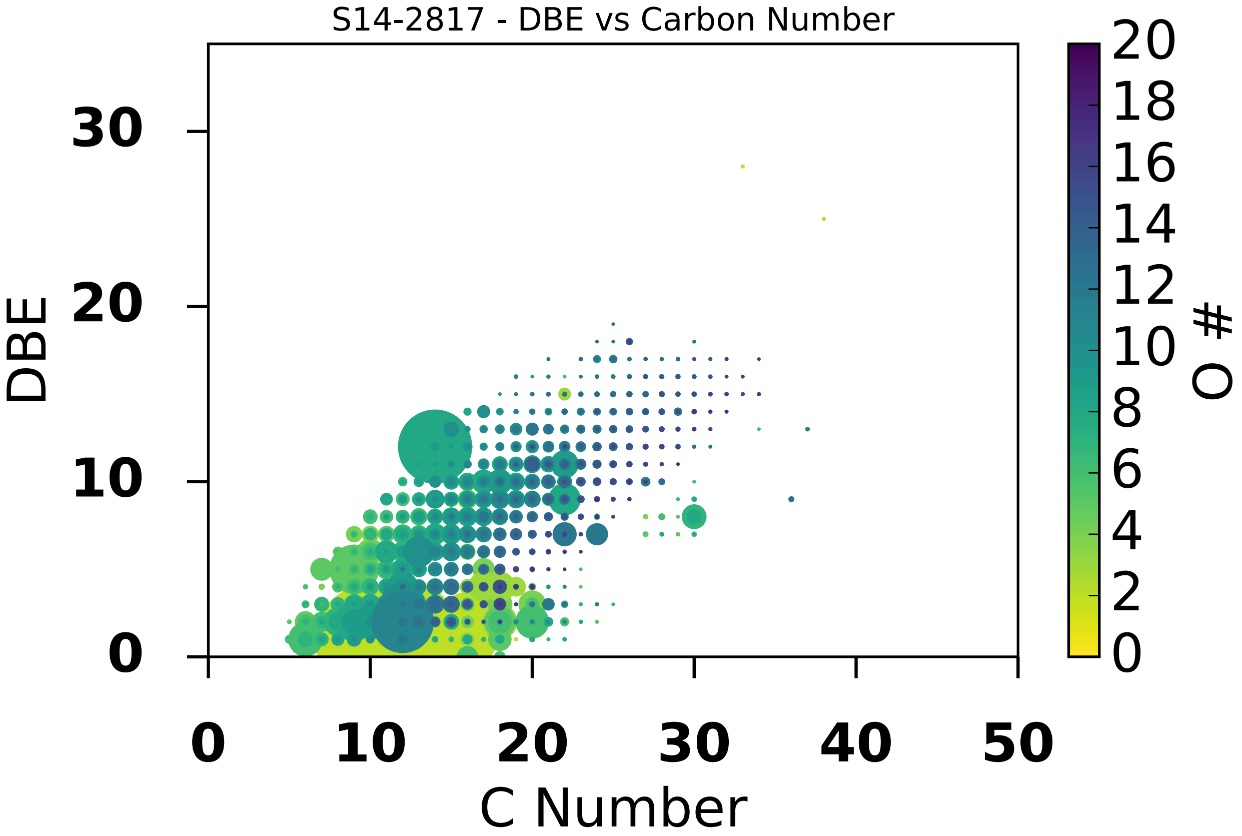

S14-2817 - DBE vs Carbon Number
DBE
30
20
10
0
0
10
20
30
40
50
C Number
O #
20
18
16
14
12
10
8
6
4
2
0

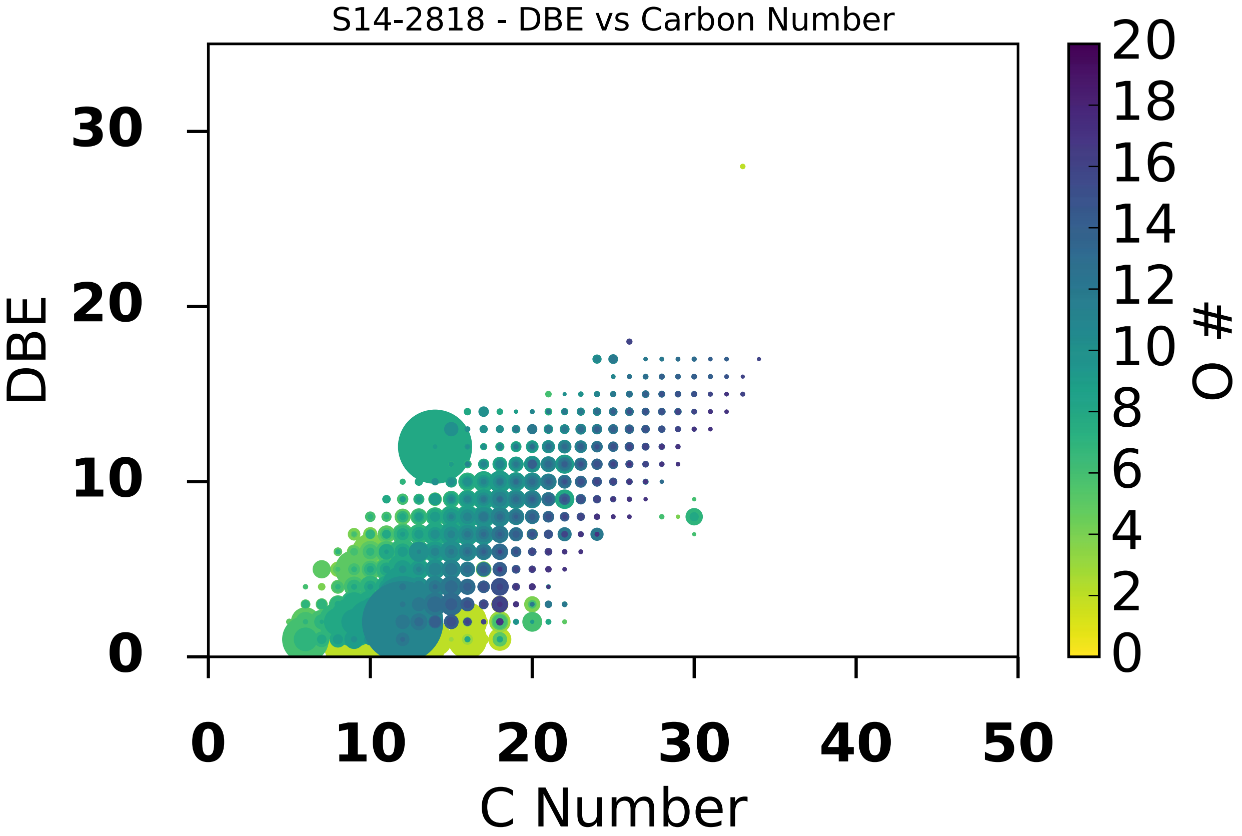

S14-2818 - DBE vs Carbon Number
DBE
0
10
20
30
C Number
0
10
20
30
40
50
O #
0
2
4
6
8
10
12
14
16
18
20

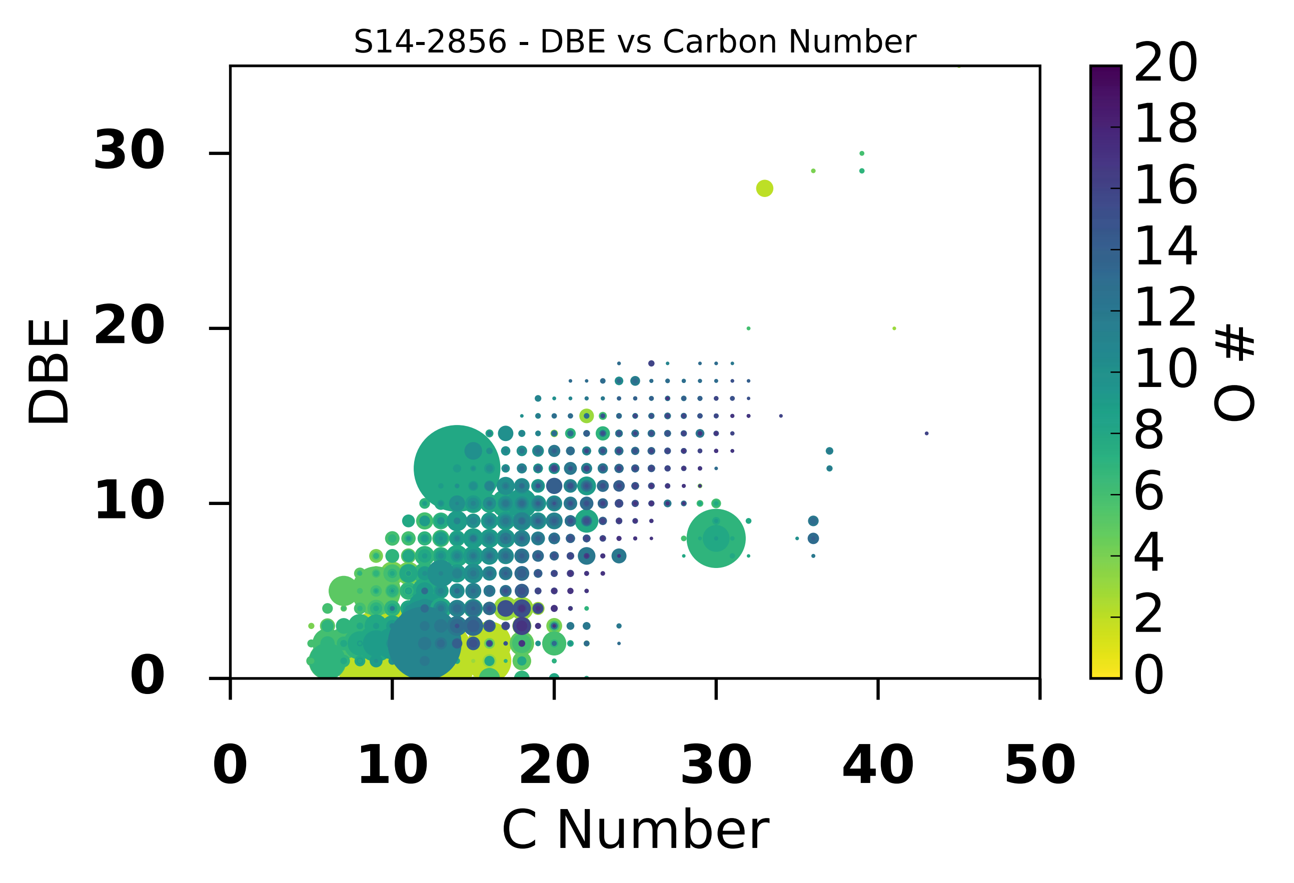
S14-2856 - DBE vs Carbon Number
DBE
30
20
10
0
0
10
20
30
40
50
C Number
O #
20
18
16
14
12
10
8
6
4
2
0

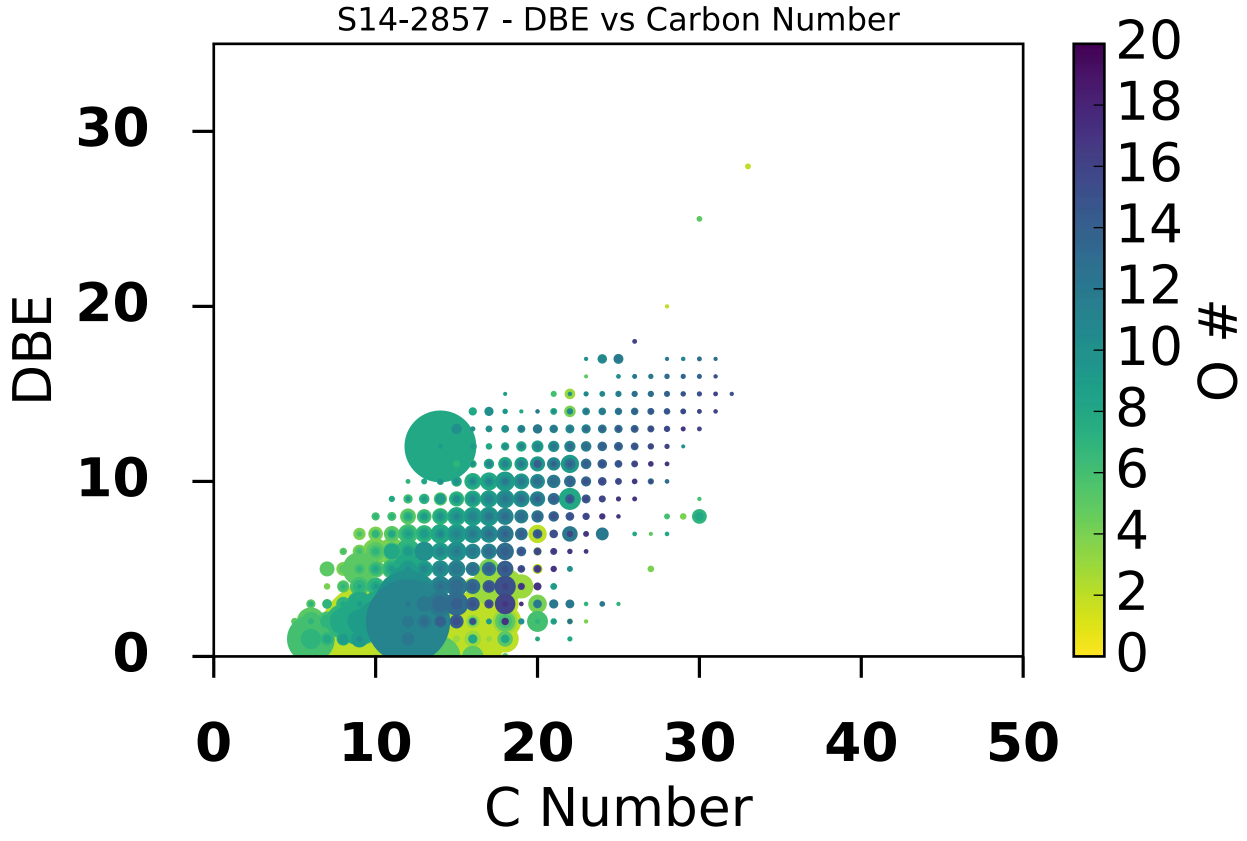

S14-2857 - DBE vs Carbon Number
DBE
0
10
20
30
C Number
0
10
20
30
40
50
O #
0
2
4
6
8
10
12
14
16
18
20

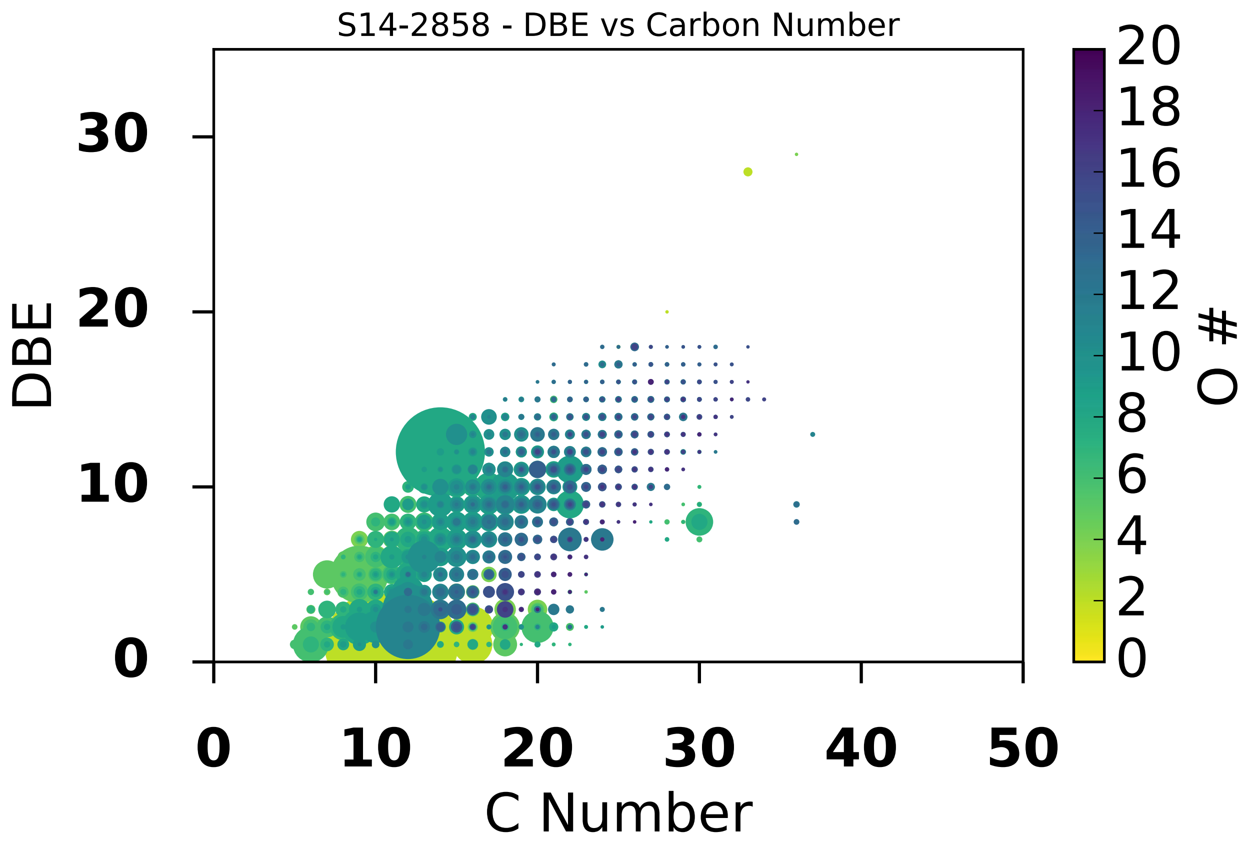
S14-2858 - DBE vs Carbon Number
DBE
0
10
20
30
C Number
0
10
20
30
40
50
O #
0
2
4
6
8
10
12
14
16
18
20
